# Design and synthesis of photoswitchable desloratadine ligands for histamine H_1_ receptor photopharmacology

**DOI:** 10.1039/d5md00589b

**Published:** 2025-08-13

**Authors:** Lars C. P. Binkhorst, Ivana Josimovic, Daan de Vetten, Tyrone J. Nijman, Niels J. Hauwert, Sufyan Ahmad, Oscar P. J. van Linden, Iwan J. P. de Esch, Henry F. Vischer, Maikel Wijtmans, Rob Leurs

**Affiliations:** a Amsterdam Institute of Molecular and Life Sciences, Division of Medicinal Chemistry, Faculty of Science, Vrije Universiteit Amsterdam De Boelelaan 1083 1081 HV Amsterdam The Netherlands m.wijtmans@vu.nl r.leurs@vu.nl

## Abstract

Despite the pharmacological relevance of the histamine H_1_ receptor (H_1_R), the second most therapeutically targeted G protein-coupled receptor (GPCR), an effective photoswitchable ligand to optically control this receptor remains elusive. In this work, we aimed to identify a suitable photoswitchable H_1_R ligand by performing an ‘azoscan’ on the H_1_R antagonist desloratadine. Taking advantage of the synthetic toolbox available for the desloratadine scaffold, aniline groups were regioselectively installed on the aromatic positions of this scaffold to enable the synthesis of azobenzene analogs targeting the orthosteric binding pocket of H_1_R. Additionally, we functionalized the piperidine ring of desloratadine with azobenzene moieties. These two strategies resulted in a total of nine photoswitchable compounds, displaying efficient *trans* to *cis* isomerization (PSS_*cis*_ > 87%) and a broad range of thermal relaxation half-lives. Pharmacological evaluation revealed the 2-position (10a) to be most suitable for accommodation of a photoswitchable group, as it exhibits the most balanced profile in absolute affinity (*K*_i_*trans* = 2 nM) and a 3.2-fold light-induced affinity shift. Computational docking studies provide a rationale, with the binding pose of the *trans* and *cis* isomer in the H_1_R binding pocket potentially being inverted. While the development of effective photoswitchable ligands for H_1_R remains challenging, this study provides promising opportunities for future optimization to achieve optical control of this GPCR.

## Abstract 2

Despite the pharmacological relevance of the histamine H_1_ receptor (H_1_R), the second most therapeutically targeted G protein-coupled receptor (GPCR), an effective photoswitchable ligand to optically control this receptor remains elusive.

## Graphical abstract

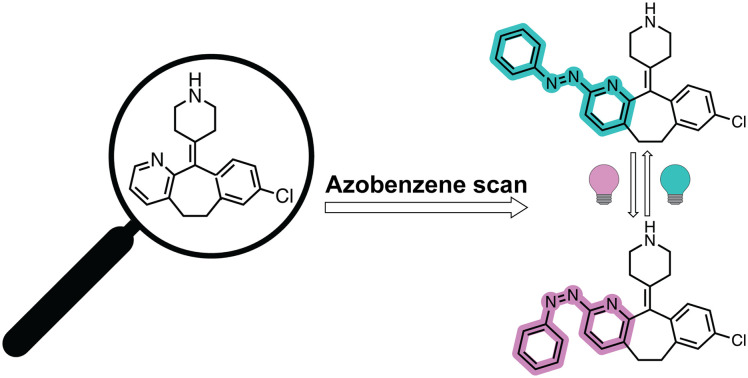

## Introduction

Photopharmacology enables precise spatiotemporal control of protein function using light, offering powerful tools to investigate dynamic signaling processes.^1^ Photopharmacology uses photoresponsive ligands,^2^ employing one of two main strategies: (i) photocaging, which uses ligands with photocleavable protecting groups^3^ or (ii) photoswitching, which uses small-molecule ligands that undergo light-induced isomerization.^1,4,5^ Successful design of photoswitchable ligands requires incorporation of a photoswitchable moiety (often an azobenzene) in such a way that the two isomers have different pharmacological properties. This can be achieved by (i) azologization, where a bioisoster (azosteres) in the core of the template ligand is replaced by an azobenzene or (ii) azoextension, where the template ligand is expanded with a photoswitchable unit.^5,6^

**Fig. 1:**
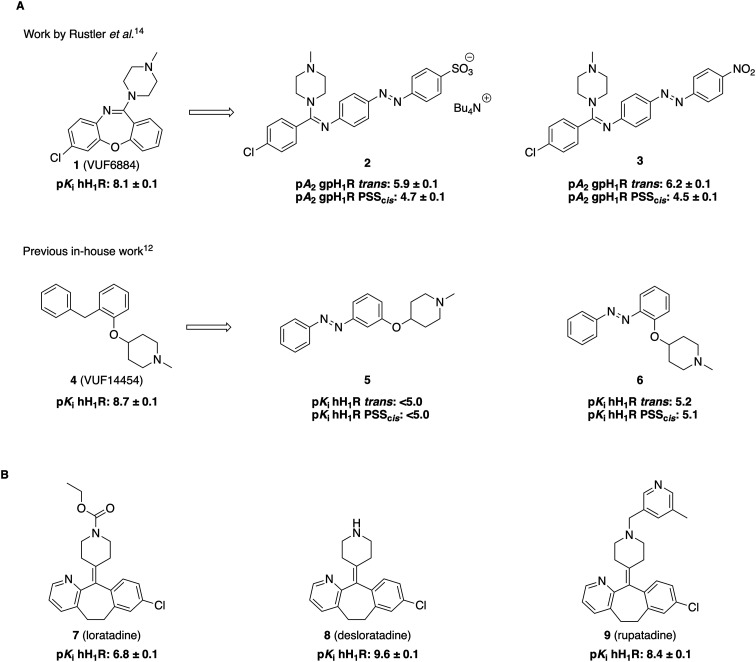
Structures and pharmacological data of key compounds. Except for 2 and 3, in-house affinity data and associated references are provided. (A) Previously reported photoswitchable ligands targeting gpH_1_R^14^ based on 1 (VUF6884)^15^ or targeting hH_1_R^12^ based on 4 (VUF14454)^16^ (B) structures of loratadine,^23^ desloratadine (value from Table 2) and rupatadine.^24^

Photoswitchable ligands have been developed for a broad range of biological targets, including ion channels, enzymes and G protein-coupled receptors (GPCRs).^6,7^ GPCRs represent one of the most pharmacologically relevant protein families, with approximately 36% of the approved drugs targeting GPCRs.^8^ After the dopamine D_2_ receptor, the histamine H_1_ receptor (H_1_R) is the second most frequent GPCR targeted by approved drugs, with 59 drugs targeting this receptor.^8^ H_1_R is widely distributed throughout the body, in, for example, smooth muscle cells, endothelial cells and the central nervous system. H_1_R antagonists, also known as antihistamines, are a class of drugs used to treat allergic diseases like allergic rhinitis, allergic conjunctivitis, and urticaria.^9^ These drugs alleviate symptoms such as itching, swelling, and redness by blocking the action of histamine on the H_1_R. Despite significant progress in the GPCR photopharmacology field,^10–13^ and the high number of drugs targeting H_1_R,^8^ it has proven remarkably difficult to develop a photoswitchable ligand that effectively modulates this receptor. Rustler *et al.* previously published photoswitchable ligands targeting guinea pig H_1_R (gpH_1_R)^14^ based on a clozapine derivative (1, Fig. 1A).^15^ However, the resulting photoswitchable compounds 2 and 3 exhibit low affinity for gpH_1_R and no data on human H_1_R (hH_1_R) were disclosed. Previous in-house efforts with photoswitchable molecules 5 and 6 based on VUF14454 (4)^16^ were also unsuccessful with the ligands having low H_1_R affinity and no appreciable affinity shift between isomers.^12^ Likewise, the desmethyl analog^16^ of these compounds or substitution of the nitrogen atom with an acidic moiety connected through a linker,^17^ were ineffective (unpublished data). Thus, an effective photoswitchable hH_1_R ligand remains elusive to date. We reasoned that the 11-(piperidin-4-ylidene)-6,11-dihydro-5*H*-benzo[5,6]cyclohepta[1,2-*b*]pyridine core, as in antihistamines loratadine (7, Fig. 1B) and desloratadine (8), provides a promising scaffold. Both are well-characterized compounds that are frequently used in exemplification for late-stage aromatic functionalization.^18–22^ In the current work, we capitalized on this synthetic accessibility by performing an ‘azoscan’ on desloratidine, a unique approach in which the azobenzene moieties are systematically installed on different positions of the template scaffold to identify a suitable position for placement of a photoswitchable moiety. These include aromatic vectors, but also *N*-substitution on the piperidine ring, generating analogs of rupatadine (9).

## Results and discussion

### Design

**Fig. 2:**
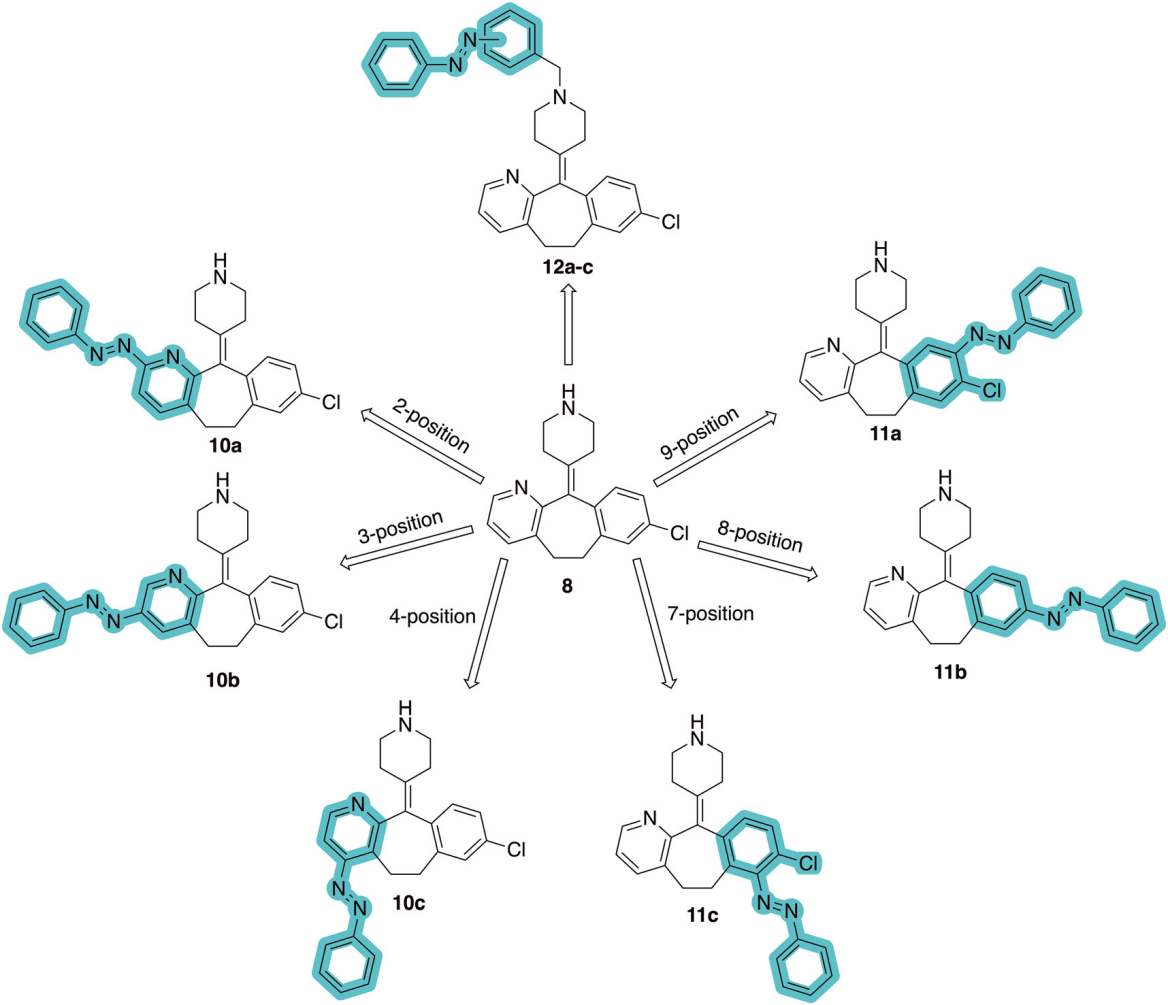
Design strategy towards a photoswitchable hH_1_R ligand by performing an ‘azoscan’ on desloratadine (8).

The design of new photoswitchable ligands targeting hH_1_R (Fig. 2) is based on the second-generation antihistamine desloratadine (8) as the template. It exhibits an approximately 600-fold higher affinity for hH_1_R than loratadine (7, Fig. 1B).^23,24^ The cryo-EM structure of desloratadine bound to hH_1_R, published by Wang *et al.*,^25^ has revealed that it engages in key hydrogen bond interactions with D107^3.32^ and Y431^6.52^ within the orthosteric hH_1_R pocket. The structure suggests that there is space for growth on desloratadine, particularly on the side of the pyridine ring (Fig. S1). Notably, Wang *et al.* also found that the ligand-binding pocket of H_1_R shows significant conformational flexibility based on the ligand bound, offering additional opportunities to potentially accommodate a photoswitchable ligand in the orthosteric pocket.^25^

The 11-(piperidin-4-ylidene)-6,11-dihydro-5*H*-benzo[5,6]cyclohepta[1,2-*b*]pyridine core of desloratadine has proven compatible with several late-stage synthetic functionalization approaches.^18–22^ We hypothesized that the use of such literature approaches would enable the installation of aniline moieties on the pyridine and phenyl rings, thereby providing synthetic vectors for the subsequent formation of an azo bond. Leveraging this synthetic accessibility, we conducted a systematic ‘azoscan’. By introducing azobenzene moieties at various positions of desloratadine, we sought to identify a photoswitchable ligand where one isomer has a favorable conformation within the binding site, while the other isomer induces steric clashes and/or unfavorable interactions to induce an affinity shift. In total, three series were explored (Fig. 2). The first two series involve introduction of an azobenzene on the pyridine ring on the 2-, 3-, and 4-positions (10a–c) and at the phenyl ring on the 7-, 8-, and 9-positions (11a–c) to target the orthosteric binding pocket. Substitution of the 8-position required removal of the chlorine of desloratadine. Importantly, Lall *et al.* have shown that replacement of the chlorine atom of loratadine with a hydrogen atom results in an equipotent compound.^26^ Furthermore, we did not pursue the 10-position, located on the phenyl ring, due to the significant synthetic challenges expected with this sterically hindered position. The third series we explored is substitution at the piperidine ring (12a–c), *i.e.*, based on rupatadine as a template.

### Synthesis

**Scheme 1:**
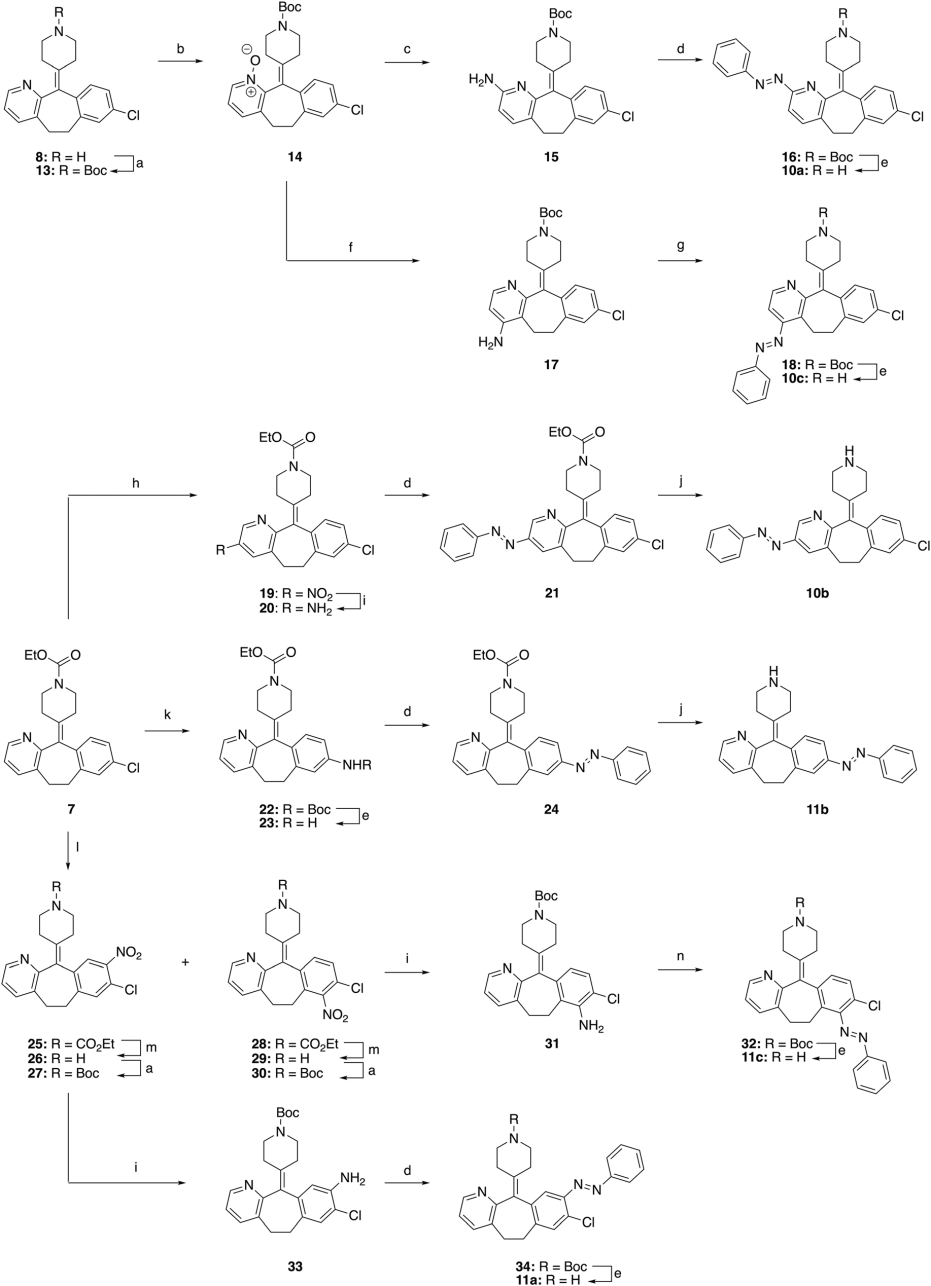
Synthesis of 10a–c and 11a–c. Reagents and conditions: (a) Boc_2_O, Et_3_N, DCM, rt, 2–16 h, 82–95%; (b) *m*-CPBA, DCM, rt, 50 min, 61%; (c) (i) potassium phthalimide, TsCl, Et_3_N, DCM, rt, 20 h; (ii) H_2_NNH_2_·H_2_O, H_2_O, 63%; (d) PhNO, AcOH, PhMe, 18 h–14 d, 75–90 °C, 8–36%; (e) 4 M HCl in 1,4-dioxane, MeOH, rt, 16–22 h, 16–98%; (f) (i) 4-cyanopyridine, Tf_2_O, DCM, MeCN, 0 °C to rt; (ii) Aq. NH_4_OH, rt, 16 h, 25%; (g) PhNO, NaH, THF, rt, 72 h, 23%; (h) Bu_4_N^+^ NO_3_^−^, TFAA, DCM, rt, 66 h, 21%; (i) Fe, NH_4_Cl, 1,4-dioxane, EtOH, H_2_O, 80 °C, 2–3 h, 81–89%; (j) KOH, EtOH, H_2_O, 80 °C, 72–100 h, 33–61%; (k) XPhos, Pd(OAc)_2_, BocNH_2_, Cs_2_CO_3_, 1,4-dioxane, 95 °C, 2 h, 82%; (l) Conc. H_2_SO_4_, KNO_3_, −10 °C – rt, 16 h, 81% of 25 and 9% of 28; (m) Conc. HCl, 80 °C, 24 h, 82–89%; (n) (i) BF_3_·Et_2_O, *t*-BuNO_2_, THF, rt, 2 h; (ii) PhMgBr, THF, −70 °C, 18 h, 14%.

The synthesis routes for the first two series of photoswitchable desloratadine analogs (10a–c, 11a–c) were designed to regio-selectively install aniline groups on the aromatic rings (Scheme 1), enabling subsequent azobenzene formation. For the preparation of the 2-isomer (10a), desloratadine (8) was first protected to give Boc-protected intermediate 13, which was oxidized with *m*-CPBA to *N*-oxide 14. *ortho*-Amination, following the method of Verbeet *et al.*,^27^ yielded aminopyridine 15. Subsequent formation of the azobenzene using PhNO under acidic Mills conditions afforded azobenzene 16, which was deprotected with HCl to yield 10a. Intermediate 14 was also used for the preparation of the 4-isomer (10c). Substitution of the hydrogen atom through a triflate-intermediate, based on a procedure by Choi *et al.*,^28^ gave intermediate 17. Due to poor reactivity of this intermediate in the Mills reaction under acidic conditions, the aniline was rendered more nucleophilic by deprotonation successfully providing 18. Deprotection of the Boc group in 18 with HCl afforded 10c. The 3-isomer was synthesized *via* regioselective nitration of loratadine (7).^29,30^ Reduction of the resulting nitro-compound 19*via* a Béchamp reduction provided aniline 20, which was subjected to acidic Mills conditions (affording 21) and carbamate deprotection with KOH to yield 10b.

For installation of the azobenzene at the 8-position (11b), a Buchwald–Hartwig amination of loratadine (7) with *tert*-butylcarbamate yielded 22. Boc-deprotection to 23 followed by the Mills reaction under acidic conditions afforded intermediate 24, which was deprotected using KOH to give 11b. The 7- and 9-analogs were accessed using a method for the nitration^29^ of loratadine that produces the two regioisomers 25 (major) and 28 (minor). For the synthesis of 11c from 28, the ethyl-carbamate protecting group was replaced with a Boc protecting group (through intermediacy of 29) to avoid the formation of side products observed in deprotection attempts of the ethyl-carbamate in the last step (data not shown). Reduction of the nitro group of resulting intermediate 30 gave aniline 31, which was converted to 32*via in situ* diazotation and reaction with PhMgBr, based on a procedure of Barbero *et al.*^31^ This alternative strategy was chosen because both acidic and basic Mills conditions on 31 were ineffective. Similarly, to obtain 11a, compound 25 was converted to Boc-protected intermediate 27*via*26. After reduction of the nitro group to 33 and a Mills reaction to 34, Boc group deprotection afforded 11a.

**Scheme 2:**
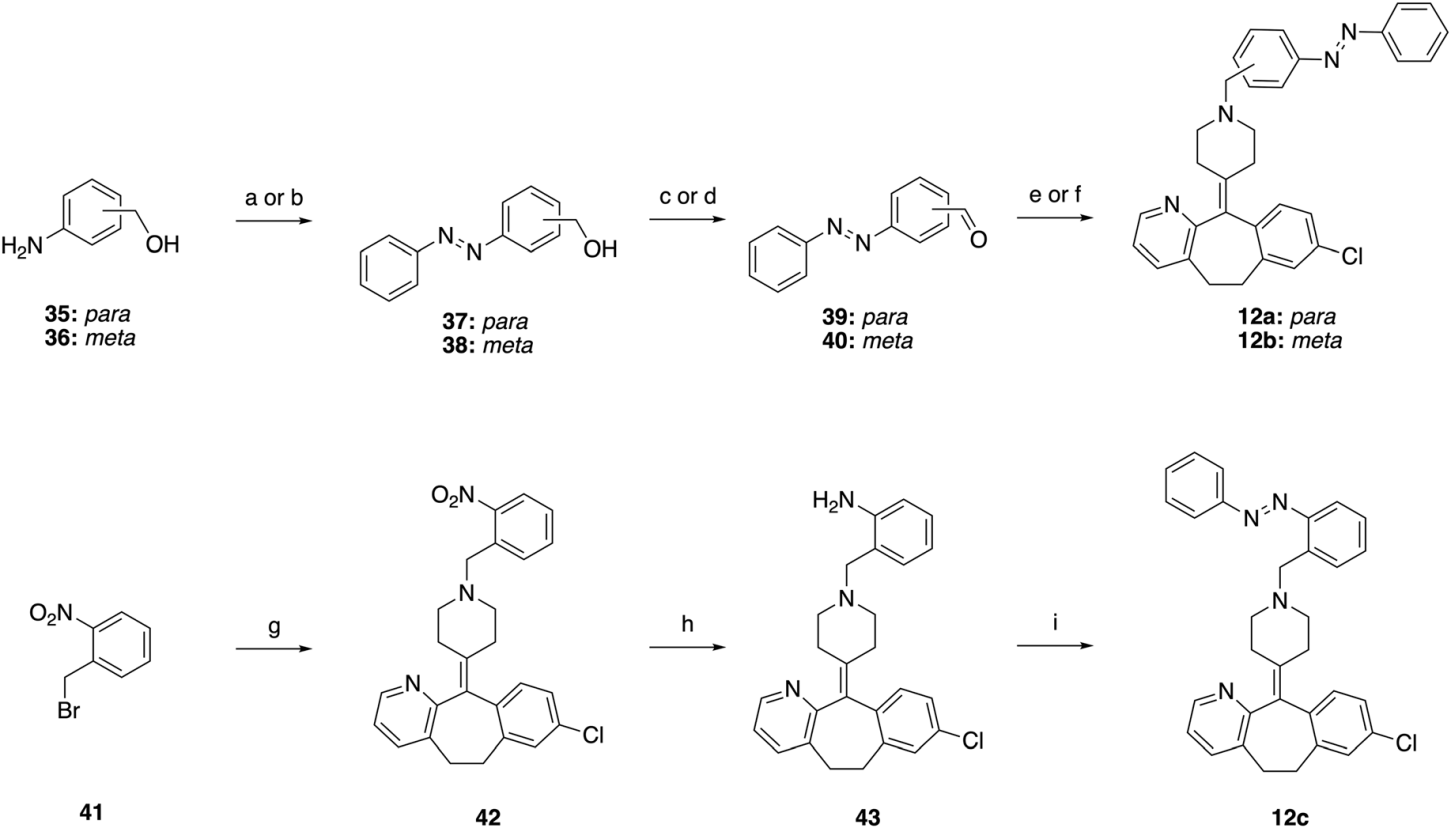
Synthesis of 12a–c. Reagents and conditions: (a) for 37: PhNO, AcOH, rt, 66 h, 12%; (b) for 38: PhNO, DCM, AcOH, rt, 18 h, 70%; (c) for 39: DMP, DCM, rt, 1.5 h, 89%; (d) for 40: MnO_2_, DCM, rt, 2 h, 75%; (e) for 12a: 8, NaBH(OAc)_3_, AcOH, DCE, rt, 16 h, 26%; (f) for 12b: 8, NaBH(OAc)_3_, AcOH, DCM, rt, 2 h, 62%; (g) 8, MeCN, K_2_CO_3_, reflux, 3 h, 91%; (h) Fe, NH_4_Cl, 1,4-dioxane, EtOH, H_2_O, 80 °C, 3 h, quantitative; (i) PhNO, PhMe, AcOH, 75 °C, 16 h, 12%.

Rupatadine analogs (12a–c) were synthesized using two different routes (Scheme 2). For analogs 12a (*para*) and 12b (*meta*), the corresponding anilines 35 and 36 were coupled to PhNO *via* the Mills reaction under acidic conditions to afford azobenzenes 37 and 38. These compounds were then oxidized to give aldehydes 39 and 40 using DMP or MnO_2_, respectively. Reductive amination with desloratadine (8) yielded rupatadine analogs 12a and 12b. In contrast, synthesis of 12c (*ortho*) required an alternative approach, as aldehyde formation from the corresponding azobenzene was unsuccessful. Instead, alkylation of desloratadine (8) with alkylbromide 41 gave nitro-compound 42, which was reduced to aniline 43 and subjected to acidic Mills conditions to afford 12c.

### Photochemistry

The photochemical properties of the photoswitchable ligands (Table 1, Fig. S2–S10) were first characterized by UV-vis absorption spectroscopy. Spectra were recorded at a concentration of 25 μM in HBSS buffer containing 50% DMSO (Fig. S2–S10). Most compounds exhibit absorption patterns characteristic for azobenzenes and azopyridines.^32–34^ The absorption maxima (*λ*_max_) of the *trans* isomers are observed around 310–340 nm (π–π* absorption band). Of note is the π–π* transition band of 11c, which is observed as a shoulder around 310 nm. Next to a potential effect of the *ortho*-Cl atom,^35^ the absence of a comparable blue-shift in π–π* band for the related compound 11a could highlight a steric effect on the absorption profile in 11c. The *λ*_max_ values of the *cis* isomers are observed at 419–435 nm (*n*–π* absorption band). Due to the short thermal half-life of 10c and 12c (*vide infra*), determination of the *λ*_max_ value of their *cis* isomer and quantification of their photostationary states (PSS) was not feasible. The *trans* to *cis* isomerization for all other compounds could be quantified upon illumination with 360 nm, resulting in PSS_*cis*_ values ranging from 87–97%. The exception was compound 11c, which only reaches a moderate PSS_*cis*_ value of 67% (*vide supra*, Fig. S7C). Indeed, UV analysis (Fig. S7B) clearly shows that 360 nm is near an isosbestic point of 11c. All compounds show expected PSS_*trans*_ values between 79–85% upon illumination with 434 nm. The approximated dark stabilities of these compounds in buffer vary substantially, with thermal half-lives ranging from seconds (12c) and minutes (10c) to days (11b, 12a) and even months (10a, 10b, 11a, 11c, 12b).

**Table 1:** Photochemical parameters of 10a–c, 11a–c and 12a–c

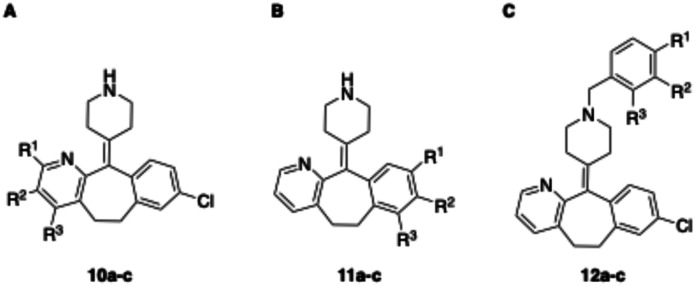									
Compound	Structure	R^1^	R^2^	R^3^	*λ*_max_ *trans* a (nm)	*λ*_max_ *cis* a (nm)	PSS_*cis*_ (area% *cis*)^b^	PSS_*trans*_ (area% *trans)*^b^	Approximate *t*_1/2_^c^
10a	A	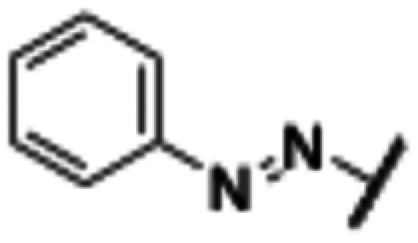	H	H	332	429	91	85	38 d
10b	A	H	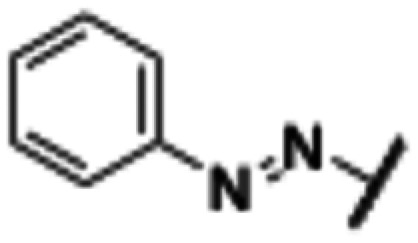	H	335	430	87	79	31 d
10c	A	H	H	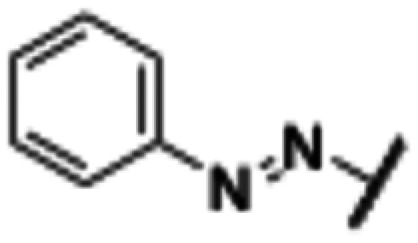	319	419	n.d.^d^	n.d.^d^	15 min
11a	B	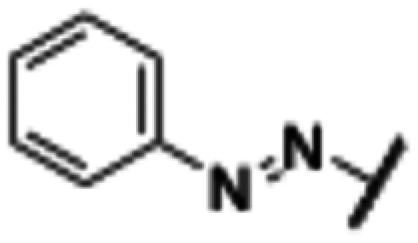	Cl	H	340	429	97	79	78 d
11b	B	H	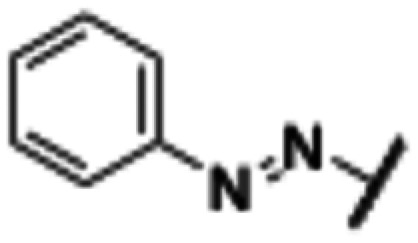	H	338	435	91	80	5 d
11c	B	H	Cl	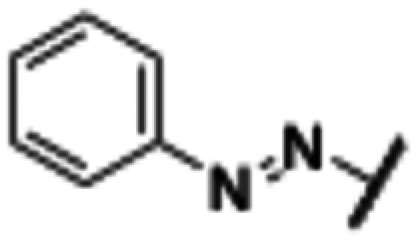	∼310^e^	419	67	82	211 d
12a	C	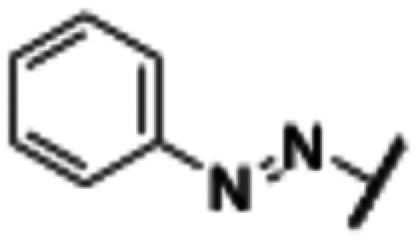	H	H	327	432	93	83	19 d
12b	C	H	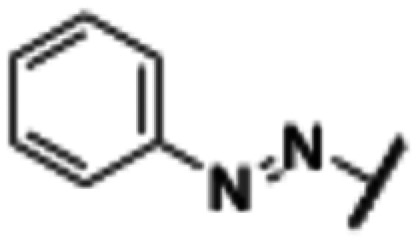	H	325	423	91	81	198 d
12c	C	H	H	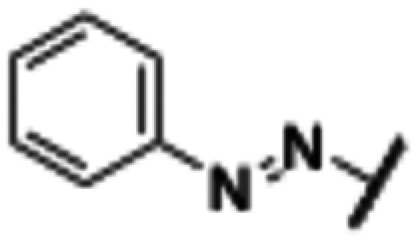	329	—	n.d.^d^	n.d.^d^	45 s

a Determined at 25 μM in HBSS buffer containing 50% DMSO.

b Photostationary state area percentages either after illumination of *trans* isomer with 360 ± 20 nm at 10 mM in DMSO as determined by acidic LC-MS analysis at the isosbestic point, or after illumination of PSS_*cis*_ states with 434 ± 9 nm at 10 mM in DMSO as determined by acidic LC-MS analysis at the isosbestic point.

c Thermal relaxation (*t*_1/2_) of PSS_*cis*_ states in HBSS buffer containing 50% DMSO, as estimated by the method of Ahmed *et al.*^36^ by extrapolating to 20 °C. Arrhenius plots are available in SI.

d Photostationary state area percentages could not be determined by LC-MS analysis due to the small *t*_1/2_ value.

e Approximate value due to overlapping bands.

### Pharmacology

The affinities (p*K*_i_ values) of 10a–c, 11a–c and 12a–c for the hH_1_R were evaluated in competition radioligand-binding experiments with the labelled H_1_R antagonist [^3^H]mepyramine (Table 2, Fig. S11–S13). For 10c and 12c, continuous illumination at 365 nm was used to counteract the short half-lives of their PSS_*cis*_ states, while for the other compounds PSS_*cis*_ states were obtained by pre-illumination with 360 nm. In the first series, featuring substitutions on the pyridine ring (10a–c), substitution on the 2-position (10a) provides a compound with high affinity for the *trans* isomer (p*K*_i_ = 8.3, template 8: p*K*_i_ = 9.6) and a lower affinity in the PSS_*cis*_ state (p*K*_i_ = 7.8), resulting in a significant light-induced affinity shift of −0.5 log unit (*i.e.*, a 3.2-fold shift in affinity). Incorporation of an azobenzene at the 3-position (10b) is also well tolerated (p*K*_i_*trans* = 8.7)*.* However, a reduced light-induced affinity shift was observed between *trans*-10b and 10b-PSS_*cis*_ (−0.3 log unit). Placement of the azobenzene on the 4-position (10c) reduces affinity for the *trans* isomer compared to 10a and 10b, providing high-nM affinities (p*K*_i_10c = 6.8). However, 10c shows no affinity shift upon photoisomerization, indicating that this position is not optimal for azobenzene placement. In the second series (11a–c), which involves substitution on the phenyl ring, *trans*-11a and *trans*-11b have comparable affinity to *trans*-10c (p*K*_i_ = 6.8). Noteworthy, 11b shows a significant affinity shift (−0.6 log unit) between *trans* and PSS_*cis.*_ In contrast, substitution at the 7-position (11c) results in a loss of affinity for hH_1_R for either state (p*K*_i_ < 6.0), indicating that this position is not suitable for azologization. The third series (12a–c), involving rupatadine analogs, provides high affinities for the *trans* isomers (p*K*_i_ = 8.3–8.6, template 9: p*K*_i_ = 8.4, Fig. 1). This is in line with other reports showing that the piperidine of 8 can be substituted without eroding H_1_R affinities.^24,37,38^ However, none of the ligands 12a–c shows an affinity shift upon photoisomerization, indicating that the *N*-substitution of 8 is not a viable strategy to achieve photochemical modulation of hH_1_R. In all, the 10 and 12 series are generally more amenable to appending *trans*-azobenzene moieties while maintaining affinities, with only the 10 series also showing some appreciable affinity shifts upon photoisomerization. The 11 series notably suffers from reduction in H_1_R affinity upon appending a *trans* azobenzene, although some members in this series show affinity shifts upon photoisomerization. In all, 10a and 11b demonstrate the most pronounced light-induced affinity shifts among the three series (−0.5 and −0.6, respectively). Notably, 10a displays an approximately 25-fold higher H_1_R affinity (p*K*_i_*trans* = 8.3) compared to 11b and therefore emerges as the most suitable photopharmacological H_1_R ligand in this study.

**Table 2:** Human histamine H_1_R binding affinity (p*K*_i_) values and affinity shifts of 10a–c, 11a–c and 12a–c

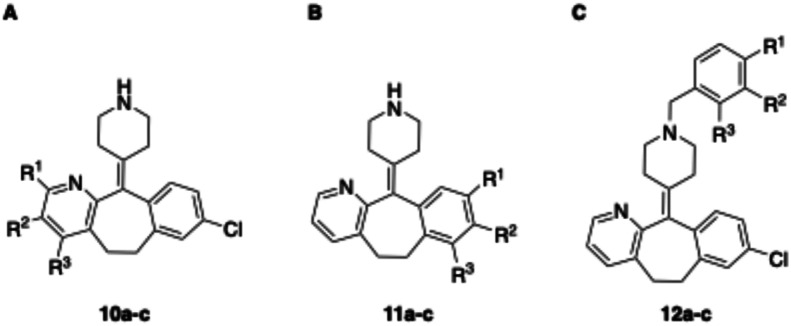							
Compound number	Structure	R^1^	R^2^	R^3^	p*K*_i_*trans*^a^	p*K*_i_ PSS_*cis*_^a^	p*K*_i_ shift^b^
8	A	H	H	H	9.6 ± 0.1	—	—
10a	A	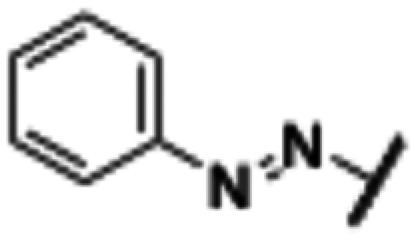	H	H	8.3 ± 0.2	7.8 ± 0.2	−0.5
10b	A	H	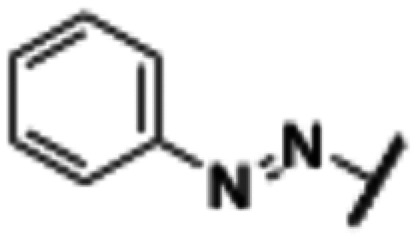	H	8.7 ± 0.1	8.4 ± 0.1	−0.3
10c	A	H	H	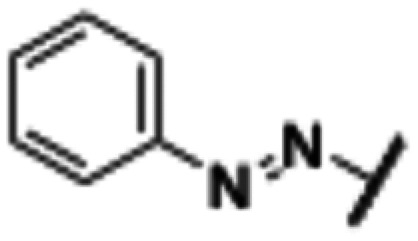	6.8 ± 0.0	6.8 ± 0.0^c^	0.0
11a	B	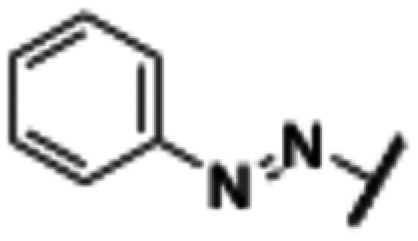	Cl	H	6.8 ± 0.2	6.6 ± 0.1	−0.2
11b	B	H	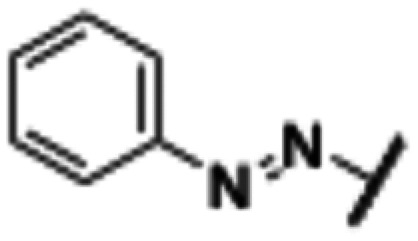	H	6.9 ± 0.0	6.3 ± 0.1	−0.6
11c	B	H	Cl	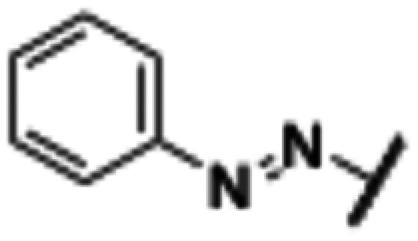	<6.0	<6.0	—
12a	C	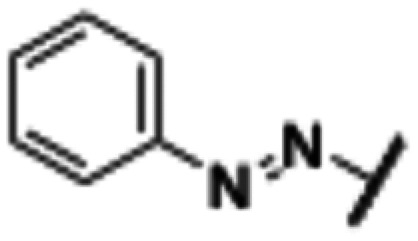	H	H	8.6 ± 0.5	8.5 ± 0.2	−0.1
12b	C	H	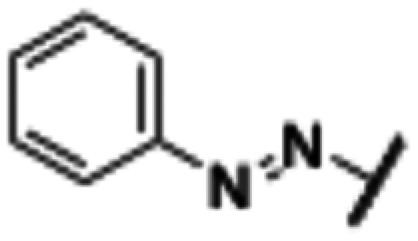	H	8.3 ± 0.1	8.2 ± 0.1	−0.1
12c	C	H	H	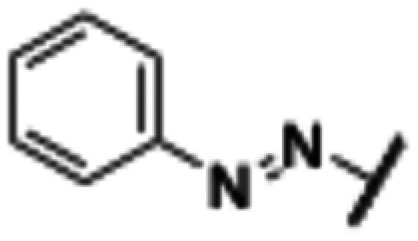	8.4 ± 0.2	8.3 ± 0.2^c^	−0.1

a Affinity (p*K*_i_) values as obtained from radioligand competition experiments with [^3^H]mepyramine. Values are mean ± SEM of *n* = 3 experiments, performed in triplicate. Competition binding curves are available in the SI.

b Affinity shifts between PSS_*cis*_ and *trans* states are defined as p*K*_i_ PSS_*cis*_–p*K*_i_*trans*.

c Continuous illumination at 365 nm was used during 4 h incubation at 25 °C.

### Proposed binding mode of 10a

**Fig. 3:**
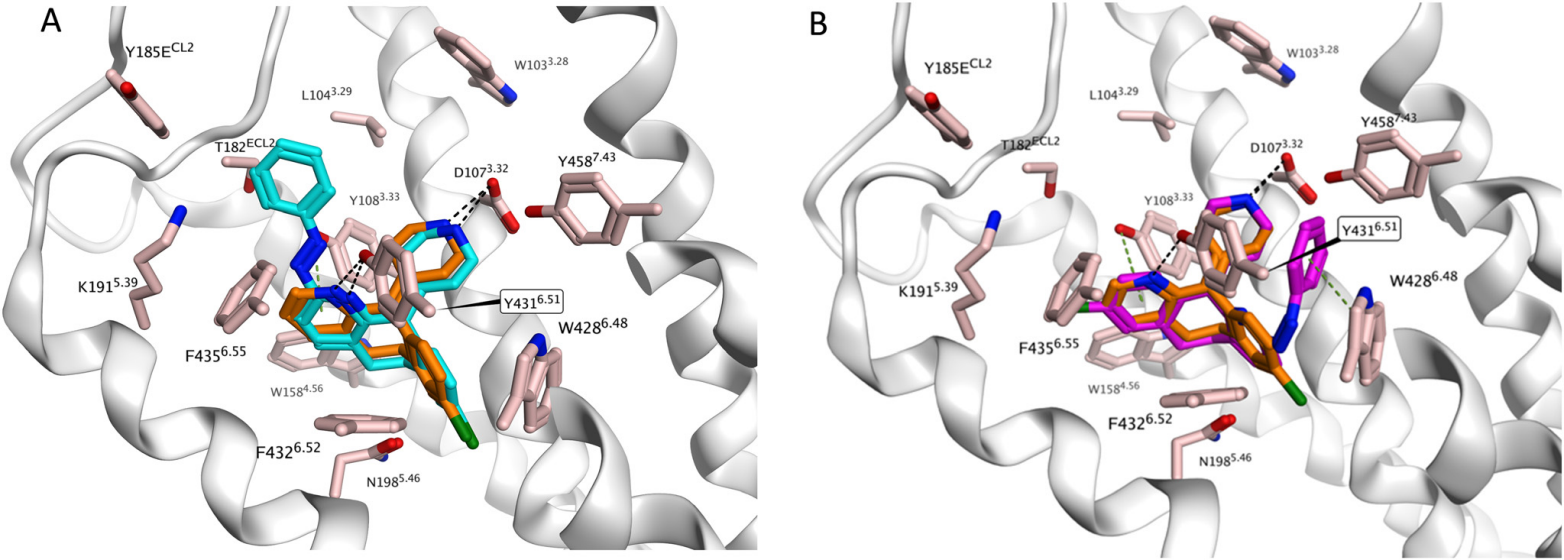
Proposed binding mode of desloratadine (orange) in the H_1_R binding pocket as determined by cryo-EM (PDB ID: 8X64)^25^ in overlay with the docking pose of (A) *trans*-10a (cyan) and (B) *cis*-10a (purple).

Molecular modelling using the recently disclosed cryo-EM structure of H_1_R with 8 (PDB ID: 8X64)^25^ was performed to gain insight in the observed affinities of *trans*- and *cis*-10a. We investigated whether both isomers could bind to H_1_R in a similar fashion as desloratadine. Indeed, *trans*-10a adopts a conformation similar to that of desloratadine (Fig. 3A). In this docking pose, the key interactions of the protonated amine with D107^3.32^ and of the pyridine nitrogen atom with Y431^6.52^ are maintained. The azobenzene moiety is directed towards the solvent-exposed region. In contrast, no comparable docking poses could be identified for *cis*-10a. Instead, for *cis*-10a a binding mode was identified in which the desloratadine core was flipped 180 degrees in the binding pocket. In this binding mode *cis*-10a maintains the key interaction with D107^3.32^*via* its protonated amine but lacks the hydrogen bond interaction with Y431^6.52^. The azobenzene moiety is buried deep in the pocket where it forms a π-stacking interaction with W428^6.48^, while the chloro-substituted ring of the desloratadine core forms an arene-H interaction with Y108^3.33^. These binding modes explain the reduced affinity of *cis*-10a compared to that of *trans*-10a, while also providing a rationale for the still appreciable affinity of *cis*-10a owing to the maintained key ionic interaction with D107^3.32^ and the two newly formed interactions with Y108^3.33^ and W428^6.48^. Based on these findings, computer-aided approaches could help the design of the next generation of desloratadine-based photoswitchable ligands, for example by focusing on increasing the bulk on the peripheral phenyl ring of 10a. These modifications may allow the *trans* isomer to maintain a similar binding mode to *trans*-10a as its azobenzene moiety is directed towards the solvent-exposed region, while the *cis* isomer in its inverted binding mode would experience steric clashes with the protein. This in turn would lower the affinity of the *cis* isomer and therefore improve the affinity shift.

## Conclusion

Finding effective photoswitchable ligands for optical control of the hH_1_R remains challenging, which may be attributed to the intrinsic flexibility of the orthosteric binding pocket of hH_1_R. Here, a total of nine potential photoswitchable ligands for hH_1_R was explored by performing an ‘azoscan’ on the antihistamine desloratadine (8). Late-stage regioselective installation of aniline groups on the aromatic rings of the desloratadine scaffold enabled azobenzene formation and overall an aromatic azoscan. This was supplemented by a concise series of *N*-functionalized derivatives. Most ligands show efficient *trans* to *cis* isomerization (PSS_*cis*_ > 87%) by using an illumination wavelength of 360 nm, except for 11c (PSS_*cis*_ = 67%). Additionally, a wide range of half-lives was observed ranging from seconds to months. Pharmacological evaluation revealed marked differences in effect upon probing azobenzenes in the three regions of 8, and only a few compounds show an appreciable light-induced H_1_R affinity shift upon installation of an azobenzene. Two suitable positions, *i.e.* the 2-position (10a) and the 8-position (11b), were identified with similar H_1_R affinity shifts between the *trans* and PSS_*cis*_ states. Of these, 10a shows the most balanced profile (p*K*_i_*trans* = 8.3, p*K*_i_ PSS_*cis*_ = 7.8). Molecular modeling studies indicate that the docking pose of *trans*-10a in H_1_R shows good overlap with the binding mode of desloratadine, but that, in contrast, *cis*-10a adopts a flipped binding mode. Building on these findings, a light-induced H_1_R affinity shift could potentially be improved by decorating the peripheral phenyl ring of the azobenzene of 10a. Thus, photoswitchable ligand 10a may provide a promising starting point for future development of improved hH_1_R photoswitchable ligands.

## Methods

Molecular modeling, synthetic chemistry, photochemistry, pharmacology and chemical analyses can be found in the SI.

## Author contributions

LCPB: conceptualization, investigation, methodology, formal analysis, visualization, writing – original draft; IJ: conceptualization, investigation, methodology, formal analysis, writing – review & editing; DdV: investigation, formal analysis; TJN: investigation, formal analysis; NJH: conceptualization, investigation, formal analysis, writing – review & editing; SA: investigation, formal analysis; OPJL: visualization, supervision, writing – review & editing; IJPdE: supervision, writing – review & editing; HFV: supervision, funding acquisition, writing – review & editing; MW: supervision, funding acquisition, writing – original draft; RL: supervision, funding acquisition, writing – review & editing.

## Conflicts of interest

There are no conflicts of interest to declare.

## Supplementary Material

MD-OLF-D5MD00589B-s001

## Data Availability

Supplementary Information available: Detailed photochemical characterization, synthesis, chemical analyses, molecular modeling and pharmacological characterization. See DOI: https://doi.org/10.1039/D5MD00589B. The data supporting this article have been included as part of the SI.
