# Supplementary material for: Design and synthesis of photoswitchable desloratadine ligands for histamine H1 receptor photopharmacology
Source: RSC Med Chem. 2025 Aug 13;16(10):5041–51. doi: 10.1039/d5md00589b (PMC12378361; doi:10.1039/d5md00589b)
Supplement: MD-016-D5MD00589B-s001 [file MD-016-D5MD00589B-s001.pdf]

Supplementary Information (SI) for RSC Medicinal Chemistry.
This journal is © The Royal Society of Chemistry 2025

Supporting information

Design and synthesis of photoswitchable desloratadine ligands for histamine H_1 receptor photopharmacology

Lars C.P. Binkhorst,† Ivana Josimovic,† Daan de Vetten, Tyrone J. Nijman, Niels J. Hauwert, Sufyan Ahmad, Oscar P.J. van Linden, Iwan J.P. de Esch, Henry F. Vischer, Maikel Wijtmans* and Rob Leurs*

Amsterdam Institute of Molecular and Life Sciences, Division of Medicinal Chemistry, Faculty of Science, Vrije Universiteit Amsterdam, De Boelelaan 1083, 1081 HV Amsterdam, the Netherlands.

† Authors contributed equally to this manuscript.

* Corresponding author: m.wijtmans@vu.nl (Maikel Wijtmans) & r.leurs@vu.nl (Rob Leurs).

Table of contents

Binding mode of desloratadine (Fig. S1)	S1
Photochemical analysis of compounds (Fig. S2-S10)	S2-S10
Radioligand binding of compounds (Fig. S11-S13)	S11-S13
Experimental procedures – Pharmacology	S14
Experimental procedures – Computational chemistry	S15
Experimental procedures – Photochemistry	S16
Experimental procedures – Synthesis	S17-S32
Chemical analyses	S33-S115
References	S116

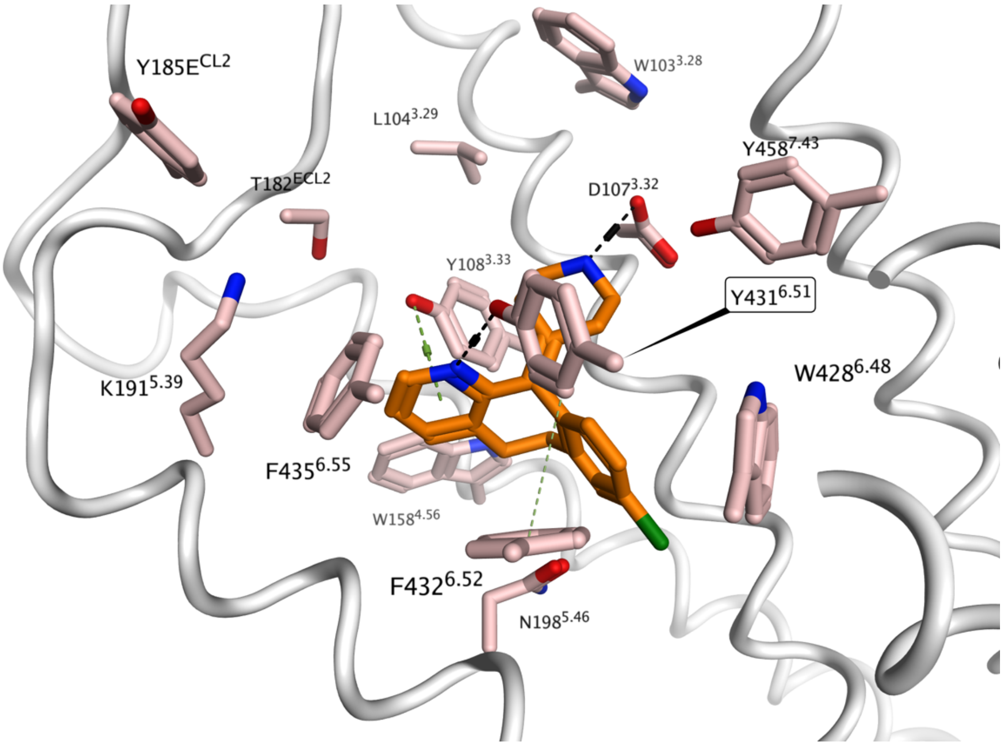

Figure S1. The cryo-EM structure of desloratadine bound to histamine H_1R (PDB:8X63)[1] visualized with MOE. Key interactions with $D107^{3.32}$ and $Y431^{6.52}$ are shown in black dashed lines.

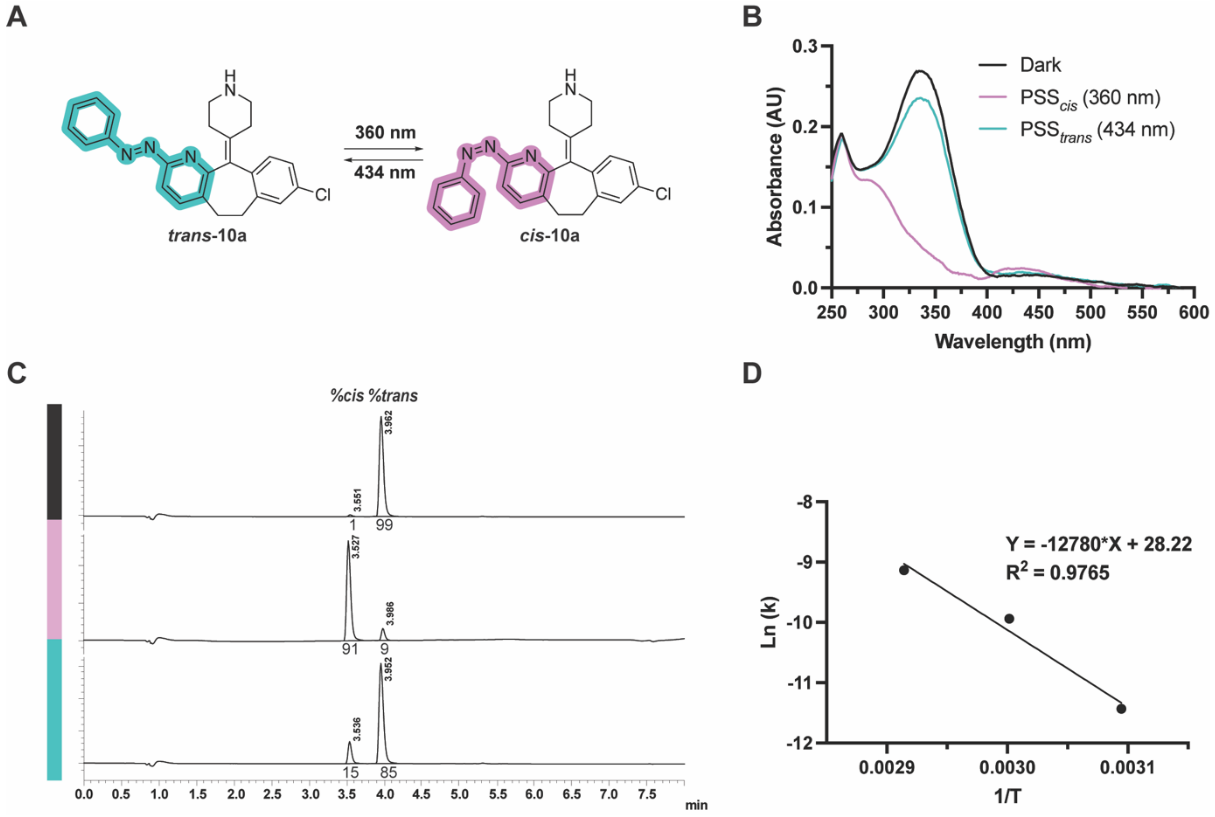

Figure S2. (A) Chemical structures of the *trans* and *cis* isomers of **10a** (B) UV-vis spectra of 25 µM of **10a** in HBSS buffer containing 50% DMSO as the *trans* isomer (black), after illumination with 360 ± 20 nm for 2 min to PSS$_{cis}$ (magenta) and after subsequent illumination with 434 ± 9 nm for 2 min to PSS$_{trans}$ (cyan). (C) *Trans*-**10a** (upper panel) and photostationary state (PSS) area percentages after illumination with 360 ± 20 nm for 10 min to reach PSS$_{cis}$ (middle panel) and after subsequent illumination with 434 ± 9 nm for 10 min to reach PSS$_{trans}$ (lower panel) at 10 mM in DMSO as determined by LC-MS analysis at the isosbestic point (265 nm). (D) Arrhenius fit for the thermal relaxation of **10a** at three different temperatures (50 °C, 60 °C, 70 °C), as measured at 25 µM in HBSS buffer containing 50% DMSO.

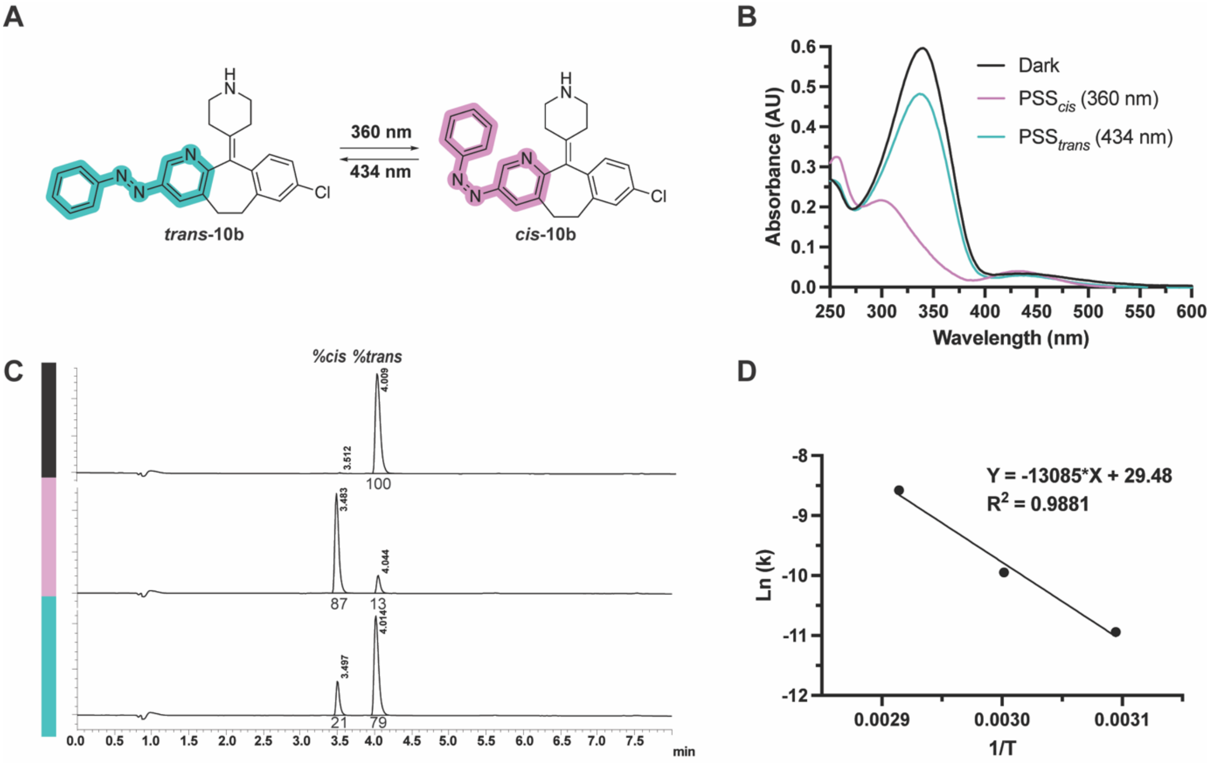

Figure S3. (A) Chemical structures of the *trans* and *cis* isomers of **10b** (B) UV-vis spectra of 25 µM of **10b** in HBSS buffer containing 50% DMSO as the *trans* isomer (black), after illumination with 360 ± 20 nm for 2 min to PSS$_{cis}$ (magenta) and after subsequent illumination with 434 ± 9 nm for 2 min to PSS$_{trans}$ (cyan). (C) *Trans*-**10b** (upper panel) and photostationary state (PSS) area percentages after illumination with 360 ± 20 nm for 20 min to reach PSS$_{cis}$ (middle panel) and after subsequent illumination with 434 ± 9 nm for 10 min to reach PSS$_{trans}$ (lower panel) at 10 mM in DMSO as determined by LC-MS analysis at the isosbestic point (280 nm). (D) Arrhenius fit for the thermal relaxation of **10b** at three different temperatures (50 °C, 60 °C, 70 °C), as measured at 25 µM in HBSS buffer containing 50% DMSO.

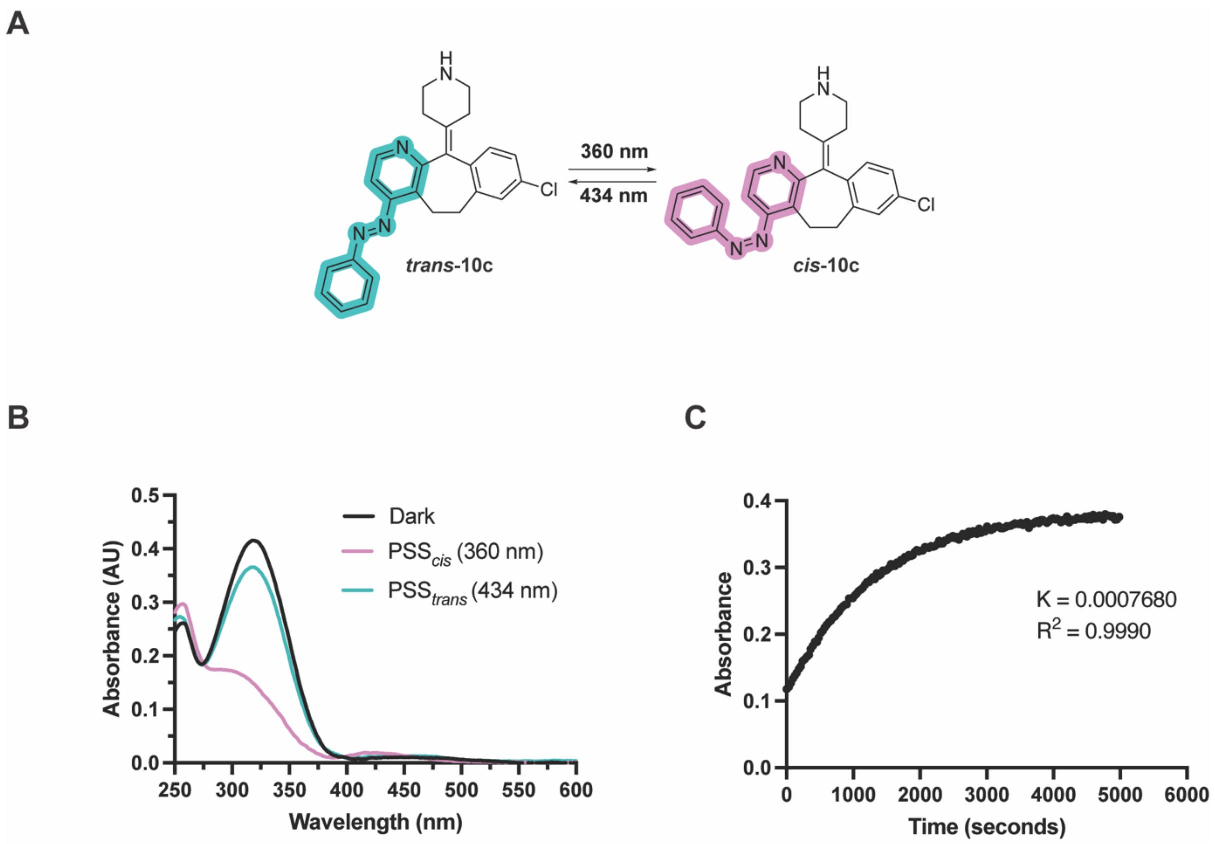

Figure S4. (A) Chemical structures of the *trans* and *cis* isomers of **10c** (B) UV-vis spectra of 25 µM of **10c** in HBSS buffer containing 50% DMSO as the *trans* isomer (black), after illumination with 360 ± 20 nm for 4 min to PSS_{cis} (magenta) and after subsequent illumination with 434 ± 9 nm for 2 min to PSS_{trans} (cyan). (C) Thermal relaxation of *cis*-**10c** at 20°C, as measured at 25 µM in HBSS buffer containing 50% DMSO by UV absorbance at 320 nm.

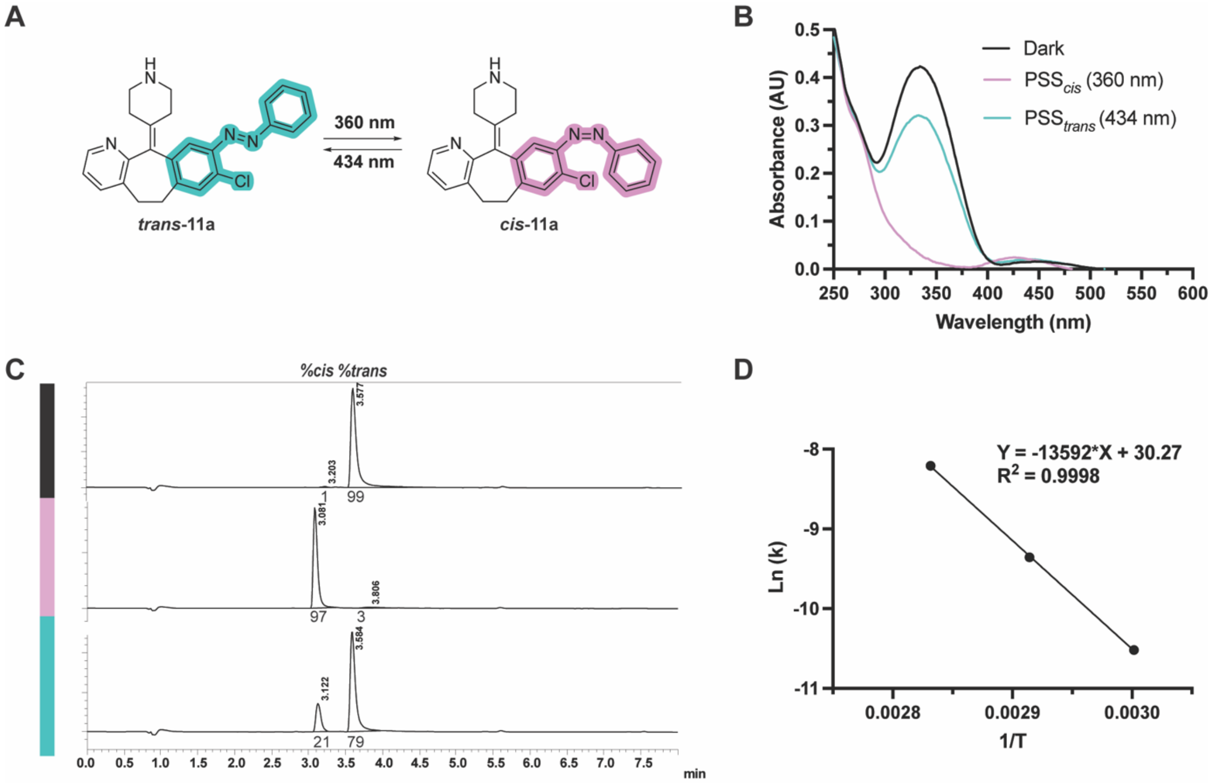

Figure S5. (A) Chemical structures of the *trans* and *cis* isomers of **11a** (B) UV-vis spectra of 25 µM of **11a** in HBSS buffer containing 50% DMSO as the *trans* isomer (black), after illumination with 360 ± 20 nm for 2 min to PSS$_{cis}$ (magenta) and after subsequent illumination with 434 ± 9 nm for 2 min to PSS$_{trans}$ (cyan). (C) *Trans*-**11a** (upper panel) and photostationary state (PSS) area percentages after illumination with 360 ± 20 nm for 5 min to reach PSS$_{cis}$ (middle panel) and after subsequent illumination with 434 ± 9 nm for 10 min to reach PSS$_{trans}$ (lower panel) at 10 mM in DMSO as determined by LC-MS analysis at the isosbestic point (275 nm). (D) Arrhenius fit for the thermal relaxation of **11a** at three different temperatures (60 °C, 70 °C, 80 °C), as measured at 25 µM in HBSS buffer containing 50% DMSO.

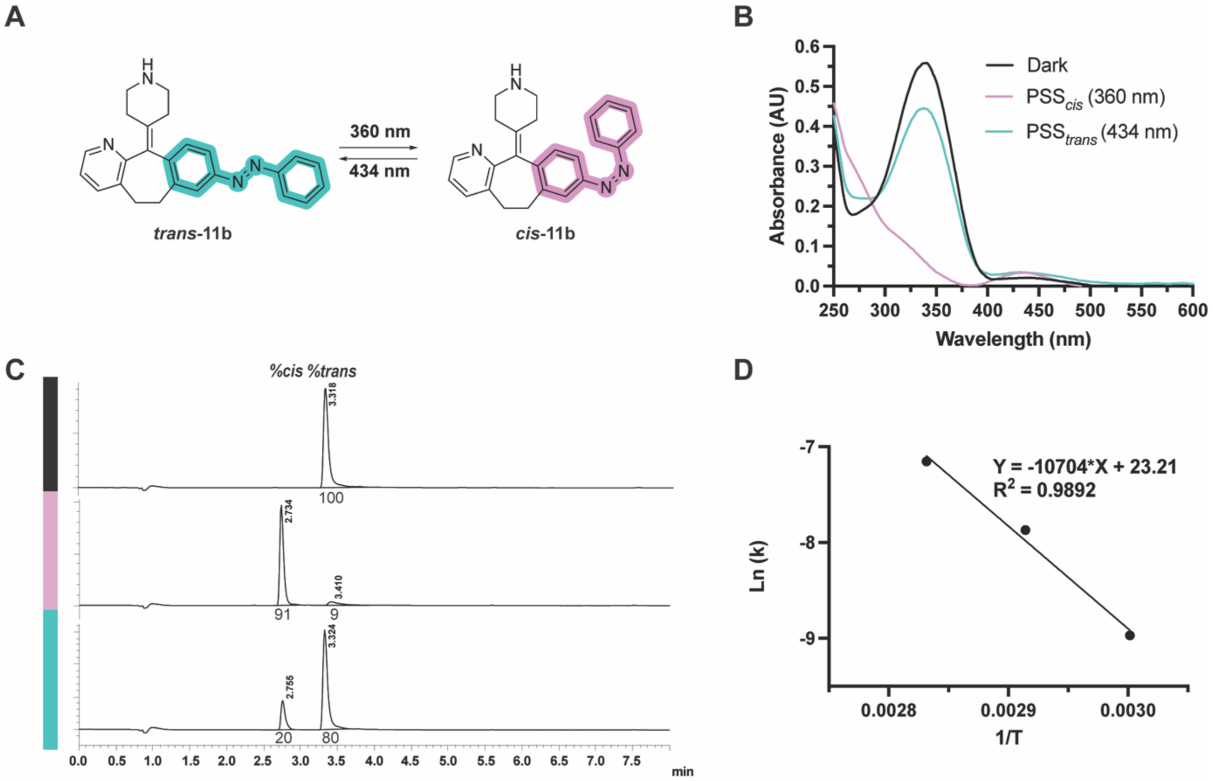

Figure S6. (A) Chemical structures of the *trans* and *cis* isomers of **11b** (B) UV-vis spectra of 25 µM of **11b** in HBSS buffer containing 50% DMSO as the *trans* isomer (black), after illumination with 360 ± 20 nm for 2 min to PSS_{cis} (magenta) and after subsequent illumination with 434 ± 9 nm for 2 min to PSS_{trans} (cyan). (C) *Trans*-**11b** (upper panel) and photostationary state (PSS) area percentages after illumination with 360 ± 20 nm for 5 min to reach PSS_{cis} (middle panel) and after subsequent illumination with 434 ± 9 nm for 10 min to reach PSS_{trans} (lower panel) at 10 mM in DMSO as determined by LC-MS analysis at the isosbestic point (290 nm). (D) Arrhenius fit for the thermal relaxation of **11b** at three different temperatures (60 °C, 70 °C, 80 °C), as measured at 25 µM in HBSS buffer containing 50% DMSO.

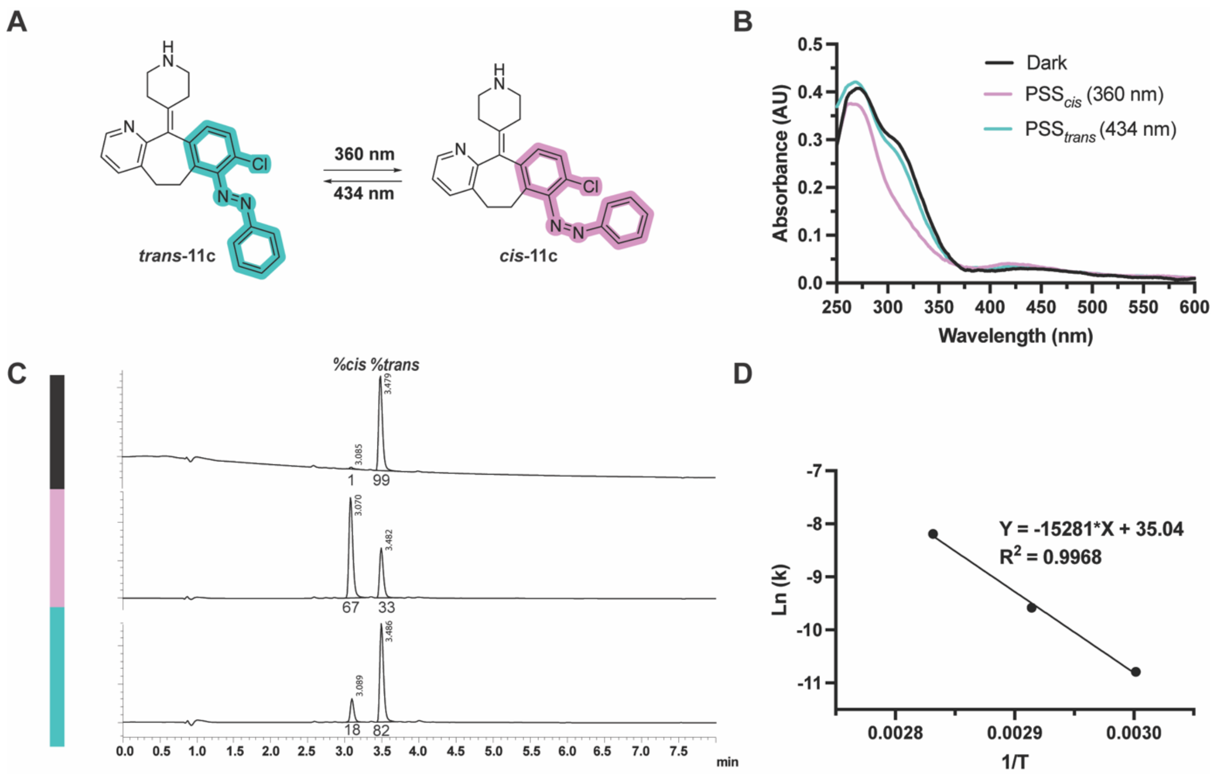

Figure S7. (A) Chemical structures of the *trans* and *cis* isomers of **11c** (B) UV-vis spectra of 25 µM of **11c** in HBSS buffer containing 50% DMSO as the *trans* isomer (black), after illumination with 360 ± 20 nm for 4 min to PSS_{cis} (magenta) and after subsequent illumination with 434 ± 9 nm for 2 min to PSS_{trans} (cyan). (C) *Trans*-**11c** (upper panel) and photostationary state (PSS) area percentages after illumination with 360 ± 20 nm for 10 min to reach PSS_{cis} (middle panel) and after subsequent illumination with 434 ± 9 nm for 10 min to reach PSS_{trans} (lower panel) at 10 mM in DMSO as determined by LC-MS analysis at the isosbestic point (255 nm). (D) Arrhenius fit for the thermal relaxation of **11c** at three different temperatures (60 °C, 70 °C, 80 °C), as measured at 25 µM in HBSS buffer containing 50% DMSO.

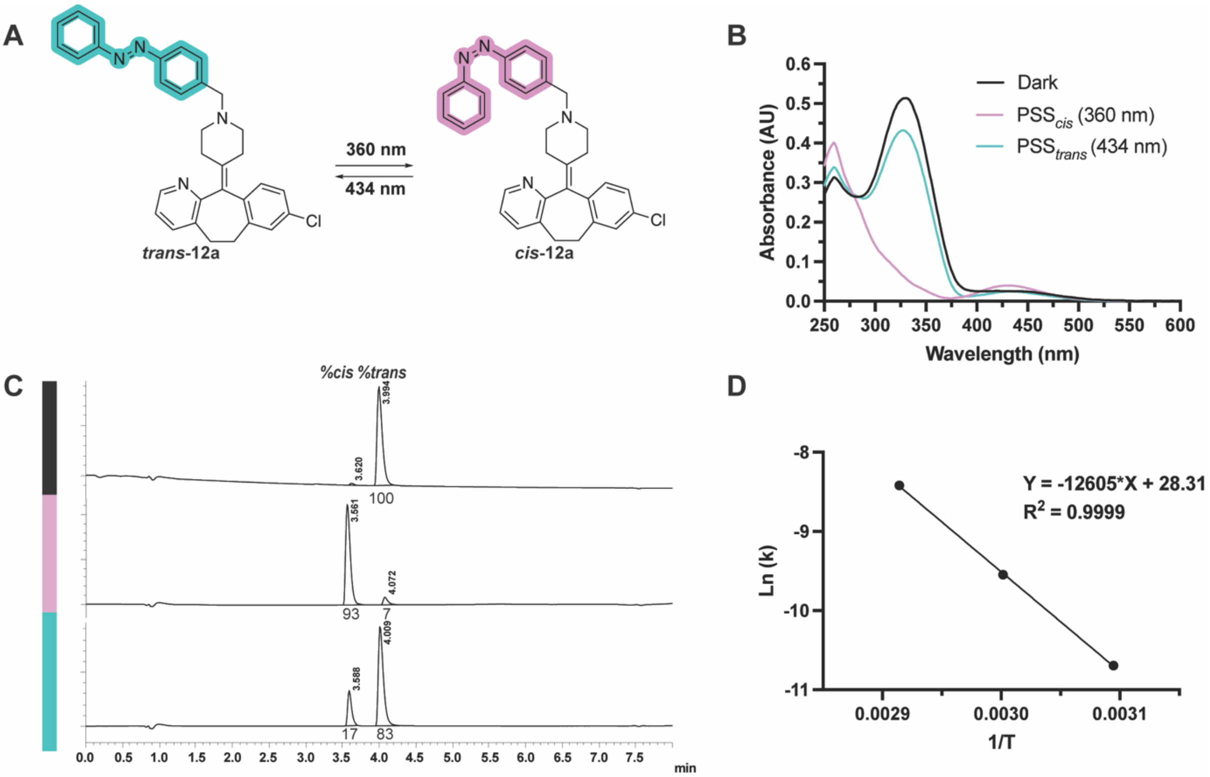

Figure S8. (A) Chemical structures of the *trans* and *cis* isomers of **12a** (B) UV-vis spectra of 25 µM of **12a** in HBSS buffer containing 50% DMSO as the *trans* isomer (black), after illumination with 360 ± 20 nm for 4 min to PSS_{cis} (magenta) and after subsequent illumination with 434 ± 9 nm for 4 min to PSS_{trans} (cyan). (C) *Trans*-**12a** (upper panel) and photostationary state (PSS) area percentages after illumination with 360 ± 20 nm for 5 min to reach PSS_{cis} (middle panel) and after subsequent illumination with 434 ± 9 nm for 10 min to reach PSS_{trans} (lower panel) at 10 mM in DMSO as determined by LC-MS analysis at the isosbestic point (280 nm). (D) Arrhenius fit for the thermal relaxation of **12a** at three different temperatures (50 °C, 60 °C, 70 °C), as measured at 25 µM in HBSS buffer containing 50% DMSO.

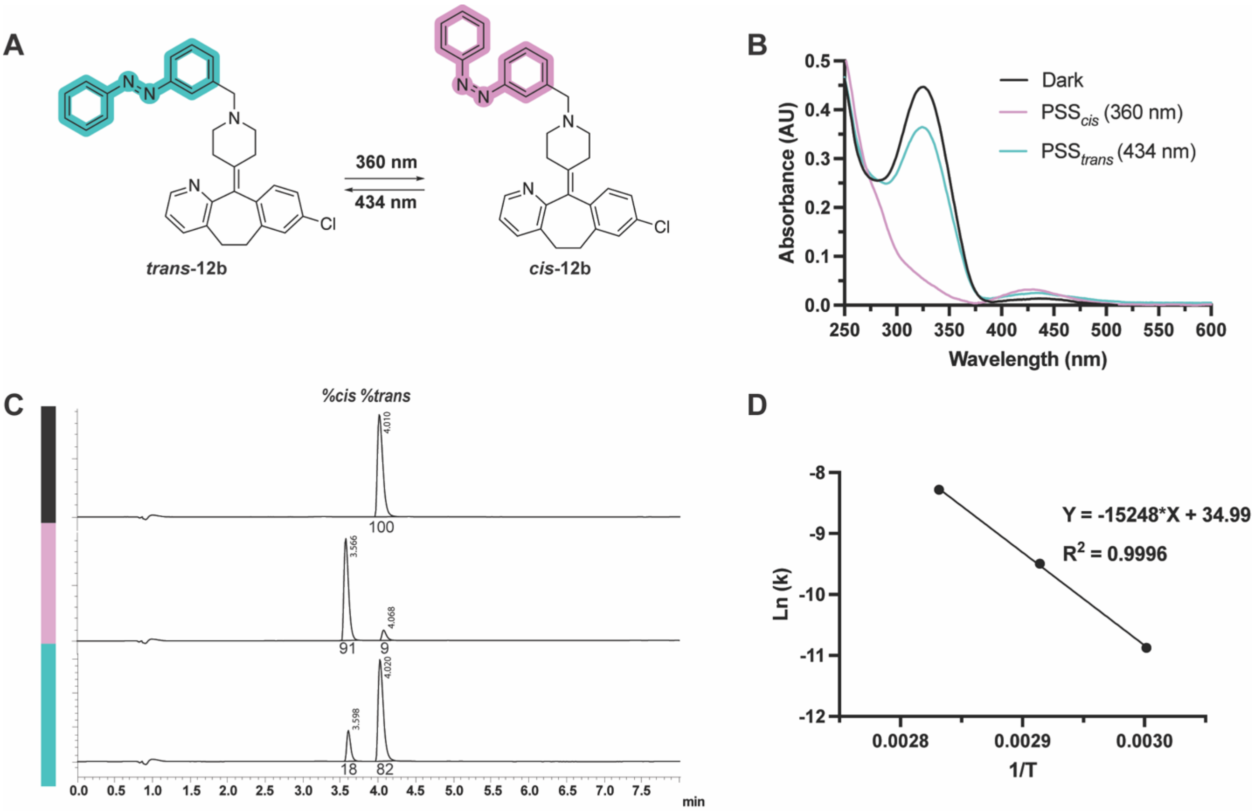

Figure S9. (A) Chemical structures of the *trans* and *cis* isomers of **12b** (B) UV-vis spectra of 25 µM of **12b** in HBSS buffer containing 50% DMSO as the *trans* isomer (black), after illumination with 360 ± 20 nm for 4 min to PSS$_{cis}$ (magenta) and after subsequent illumination with 434 ± 9 nm for 4 min to PSS$_{trans}$ (cyan). (C) *Trans*-**12b** (upper panel) and photostationary state (PSS) area percentages after illumination with 360 ± 20 nm for 5 min to reach PSS$_{cis}$ (middle panel) and after subsequent illumination with 434 ± 9 nm for 10 min to reach PSS$_{trans}$ (lower panel) at 10 mM in DMSO as determined by LC-MS analysis at the isosbestic point (270 nm). (D) Arrhenius fit for the thermal relaxation of **12b** at three different temperatures (60 °C, 70 °C, 80 °C), as measured at 25 µM in HBSS buffer containing 50% DMSO.

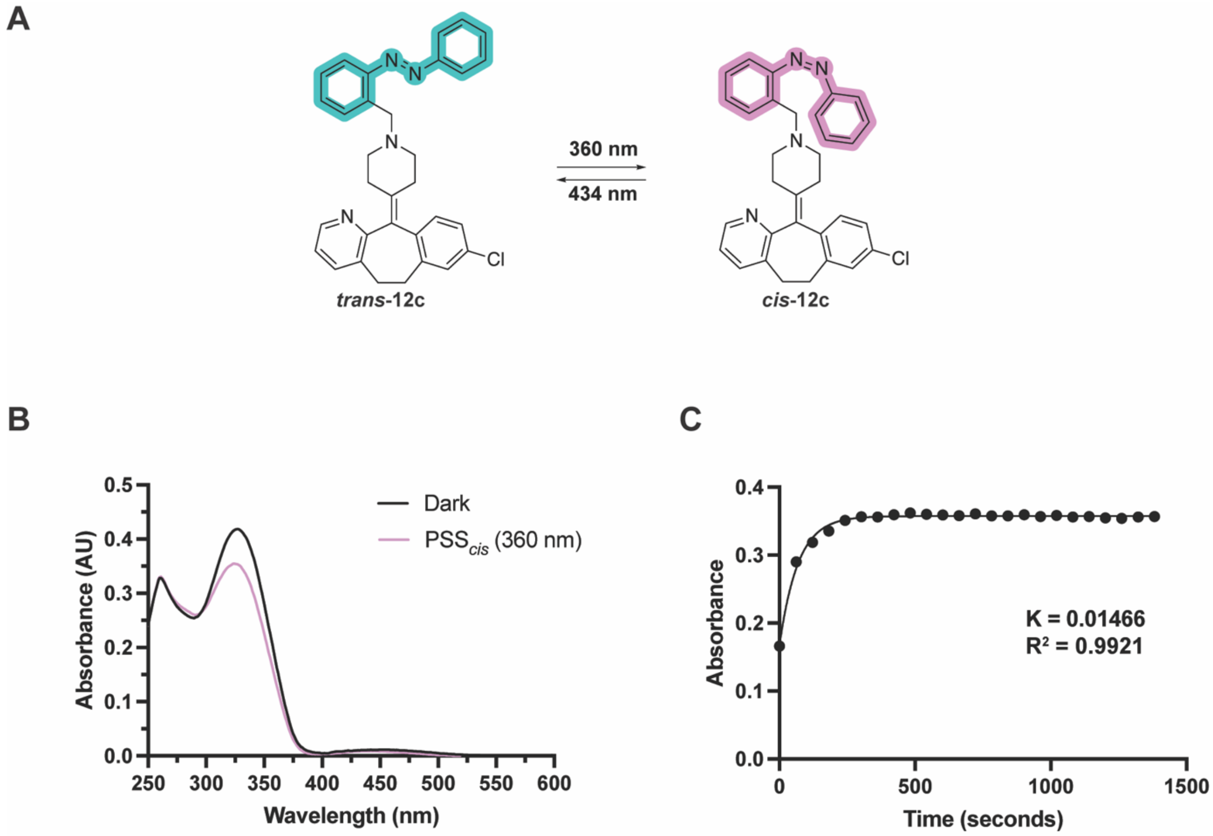

Figure S10. (A) Chemical structures of the *trans* and *cis* isomers of **12c** (B) UV-vis spectra of 25 µM of **12c** in HBSS buffer containing 50% DMSO as the *trans* isomer (black), after illumination with 360 ± 20 nm for 10 min to PSS_{cis} (magenta). (C) Thermal relaxation of *cis*-**12c** at 20°C, as measured at 25 µM in HBSS buffer containing 50% DMSO by UV absorbance at 320 nm.

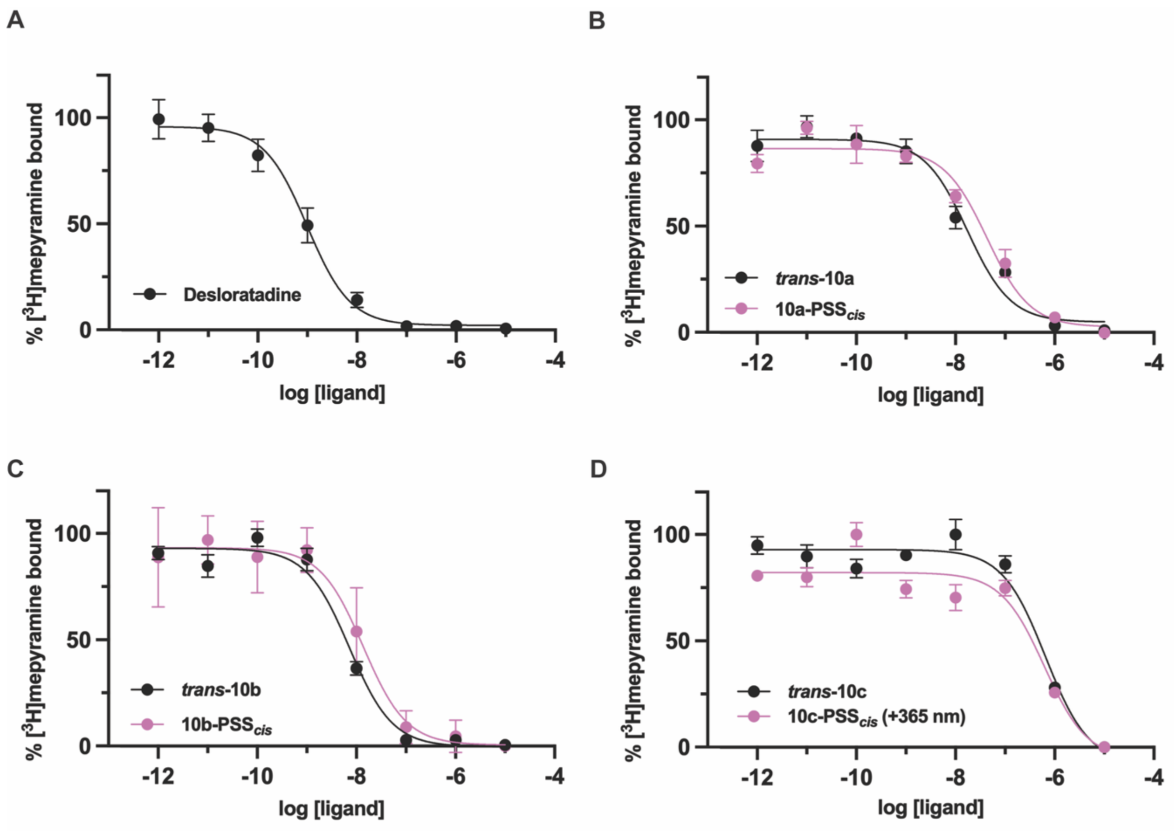

Figure S11. Competition binding curves of (A) desloratadine (**8**) and *trans* and PSS$_{cis}$ states of (B) **10a** (C) **10b** and (D) **10c** in competition with [^{3}H]mepyramine. Pooled data are shown as mean ± SEM of three independent experiments. Due to its short thermal half-life, compound **10c** was continuously illuminated with 365 nm light during incubation.

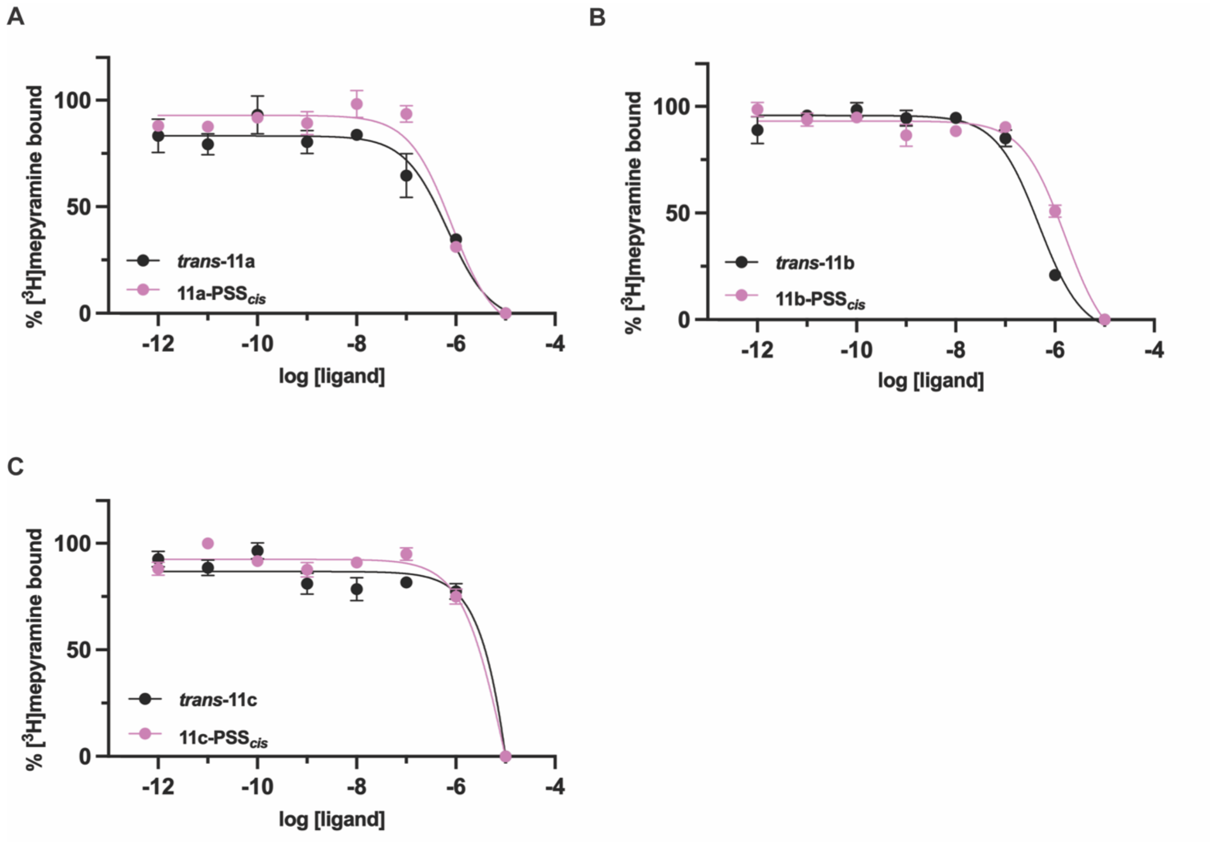

Figure S12. Competition binding curves of *trans* and PSS_{cis} states of (A) **11a** (B) **11b** and (C) **11c** in competition with [^{3}H]mepyramine. Pooled data are shown as mean ± SEM of three independent experiments.

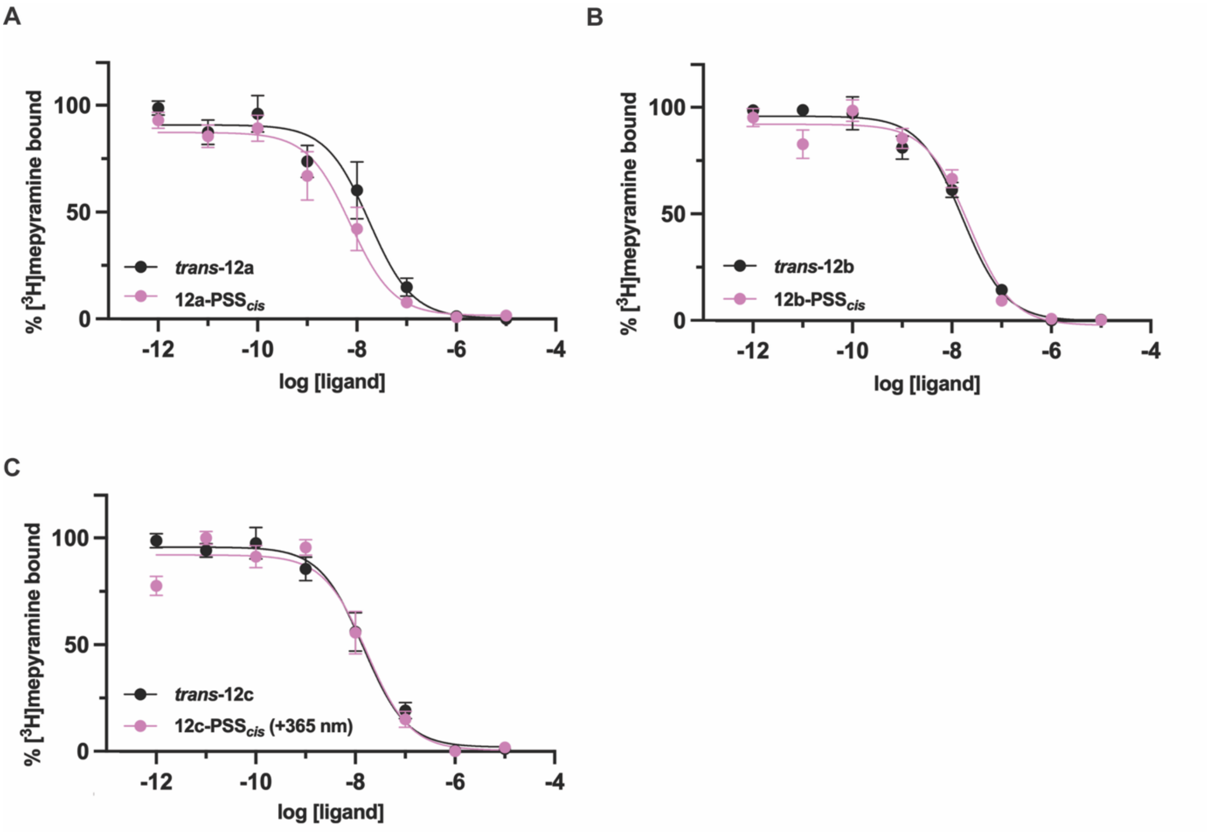

Figure S13. Competition binding curves of *trans* and PSS_{cis} states of (A) **12a** (B) **12b** and (C) **12c** in competition with [^{3}H]mepyramine. Pooled data are shown as mean ± SEM of three independent experiments. Due to its short thermal half-life, compound **12c** was continuously illuminated with 365 nm light during incubation.

Experimental methods – Pharmacology

Radioligand binding experiments were performed in the following manner, as published previously, with minor changes.[2,3] Cell homogenates were made using HEK293T cells, transiently expressing the N-terminally HA-tagged H_1R. The homogenates were stored at −20 °C. For the assay, the HA-H_1R-expressing cell homogenates were resuspended in radioligand binding buffer (Na_2HPO_4 (50 mM) and KH_2PO_4 (50 mM)), pH 7.4, and homogenized with a Branson Sonifier 250 (Branson Ultrasonics, Danbury, CT). Afterwards, the cell homogenates were incubated with the respective (photo)ligands in increasing concentrations (10^{-5}–10^{-12} M) in competition with [^{3}H]mepyramine (2.7–4.9 nM) at 25 °C in black 96 well plates (Greiner). During the incubation time, **10c** and **12c** were continuously illuminated with 365 nm (10 V, 2 mW, constant mode) light (96 LED array plates, LAD-1, LEDA-x, Bio Research Center Co., Ltd. Izumi, Japan) to account for their short-lived *cis* isomers. After a 4-hour incubation time, the binding reaction was stopped using a harvester (PerkinElmer). Upon multiple wash steps through a polyethyleneimine-coated GF/C filter plate (PerkinElmer) receptor-bound radioligand was captured by the GF/C filter plate. After drying the GF/C plate, the filter-bound radioligand was quantified by scintillation counting using MicroScint-O and the Wallac MicroBeta counter (PerkinElmer). IC_{50} values were obtained by analyzing the displacement curves with GraphPad Prism 10 (GraphPad Software, San Diego) and were converted to K_i values using the Cheng–Prusoff equation.[4]

Experimental methods – Computational chemistry

The cryo-EM structure of the H_1R-BRIL/Anti BRIL Fab complex with desloratadine (PDB: 8X64)[1] was prepared using the Structure Preparation module in MOE v2024.06. Hydrogen atoms were added, and partial charges were calculated using the Amber:EHT forcefield. Desloratadine was subsequently extracted from the complex. *Trans*-**10a**, *cis*-**10a** and desloratadine were converted from their SMILES representation into 3D structures using MOE. Each compound was visually inspected and modified to ensure the correct isomeric form. The protonation state of the molecules was checked and corrected as needed during this process.

The docking procedure was validated by redocking of desloratadine into the prepared histamine H_1R structure. MOE's external docking function was applied via the docking user interface, using default settings (Genetic Algorithm placement with PLP and Rigid Receptor refinement). Two hundred docking poses were generated, initially scored using London dG, and then refined. The refined poses were subsequently rescored using the GBVI/WSA dG and twenty poses with the highest score were reported. Docking scores for the desloratadine ranged from -6.5847 kcal/mol to -5.5730 kcal/mol. Interaction fingerprints (IFPs) were used to identify binding modes in which the basic amine of the ligand binds with $Asp107^{3.32}$, a hallmark interaction of aminergic GPCRs. Three out of the twenty reported structures of redocked desloratadine poses exhibited binding modes highly similar to the experimental cryo-EM structure, with RMSD values between 0.8398 and 1.2006 Å.

Trans-**10a** and *cis*-**10a** were docked in the prepared histamine H_1 structure using the same protocol. Docking scores ranged from -7.9736 to -6.5838 kcal/mol for *trans*-**10a** and -8.0865 to -7.0272 kcal/mol for *cis*-**10a**. IFP-based filtering resulted in seven docking poses for *trans*-**10a** with interactions to $Asp107^{3.32}$. The binding mode of *trans*-**10a** that was selected has a docking score of -7.5910 kcal/mol (i.e., the best docking score of the poses that make an interaction with $Asp107^{3.32}$). Applying the same IFP filtering for *cis*-**10a** gave the best docking score of -7.115 kcal/mol.

In most docking poses of *cis*-**10a**, no interaction with $Asp107^{3.32}$ was observed, and a wide range of binding poses was obtained. However, in two poses, an interaction with $Asp107^{3.32}$ was identified. In these cases, the desloratadine core was flipped relative to the experimental structure but could still be adequately overlaid.

Experimental procedures – Photochemistry

UV−Vis spectra were recorded using a Thermo-scientific Evolution 201 PC spectrophotometer equipped with a thermostated cell holder set at 20 °C. Fits of UV−vis spectroscopy data were generated using Prism 10.1.0. Illumination for all photochemical experiments was executed using a Sutter instruments Lambda LS with a 300 W full-spectrum lamp connected to a Sutter instruments Lambda 10-3 optical filter changer equipped with 360 ± 20 nm, 434 ± 9 nm 520 ± 12 nm. The light intensity used for the lambda 10-3 optical filter is 0.93 mW/mm^2 using the 360 ± 20 nm filter, 0.79 mW/mm^2 for the 434 ± 9 nm filter, 0.77 mW/mm^2 for the 494 ± 9 nm filter, and 0.20 mW/mm^2 for the 520 ± 12 nm filter as measured using a Thorlabs PM16−401 power meter. For photochemical analyses, illuminations were performed in Hellma Suprasil quartz 114QS cuvettes. Thermal relaxation experiments and Arrhenius extrapolations were performed according to Priimagi *et al,*[5] using a compound concentration of 25 µM in HBSS +50% DMSO at temperatures of 50, 60, 70, or 80 °C. Illuminations for pharmacological experiments were performed in cylindrical clear glass vials with a volume of 4.5 mL. The typical distance between light source and vial or cuvette was 2 cm.

Experimental procedures - Chemistry

All chemicals and solvents were obtained from commercial suppliers (primarily Sigma-Aldrich, BLD pharma and Combi-Blocks) and used without purification. DCM, DMF, THF and Et_2O were dried by passing through a PureSolv solvent purification system. All reactions were carried out under N_2 atmosphere unless mentioned otherwise. All reactions with photoresponsive compounds were carried out in the dark or under red light. IUPAC names were adapted from Chemdraw Professional 21.0 (PerkinElmer). Reactions were monitored by thin layer chromatography (Merck Silicagel 60 F254) by visualization under 254 nm lamp. Flash column chromatography was performed with Screening Devices 40-63 µm (Biotage) or GraceResolv (Büchi) cartridges on Isolera One with UV-Vis detection (Biotage). Nuclear magnetic resonance (NMR) spectra were determined with a Bruker Avance II 500 MHz or a Bruker Avance III HD 600 MHz spectrometer. Chemical shifts are reported in parts per million (ppm) against the reference compound using the signal of the residual non-deuterated solvent ($CDCl_3$ δ = 7.26 ppm (^1H), δ = 77.16 ppm (^{13}C); DMSO-d_6 δ = 2.50 ppm (^1H), δ = 39.52 ppm (^{13}C)). NMR spectra were processed using MestReNova 14.1 software. The peak multiplicities are defined as follows: s, singlet; d, doublet; t, triplet; q, quartet; dd, doublet of doublets; ddd, doublet of doublets of doublets; dt, doublet of triplets; dq, doublet of quartets; td, triplet of doublets; tt, triplet of triplets; qd, quartet of doublets; p, pentet; dp, doublet of pentets; br, broad signal; m, multiplet. For NMR listings, in addition to specific instructions that are given by the journal in the guidelines for authors the following additional procedures were used: 1) Multiplicity is not solely reported based on peak shapes, but also distinguishes the coupling to all non-equivalent protons that have similar J values; 2) If additional smaller couplings are observed but are too small for accurate quantitation because the precision is smaller than the digital resolution, a symbol $^{\Delta}$ will be used; 3) The notation 'm' is used in case of obscured accurate interpretation as a result of (i) overlapping signals for different protons, or (ii) a result of overlapping signal lines within the same proton signal; 4) For any rotamers or diastereomers, signals will be listed separately; 5) NMR signals that could only be detected with HSQC analysis are denoted with a $^{\#}$ symbol; 6) NMR signals that could only be detected with HMBC analysis are denoted with a * symbol; 7) If one or more signals remain undetected after extensive 1D and 2D NMR analyses, this will be mentioned. 8) Signals for exchangeable proton atoms (such as NH and OH groups) are only listed if clearly visible (excluding e.g. the use of D_2O or CD_3OD) and if confirmed by a D_2O shake and/or HSQC. Analytical HPLC analyses were performed using a Shimadzu LC-20AD liquid chromatography pump system, equipped with a Waters XBridge C18 column 5 µm 4.6 x 50 mm (used at 40 °C), connected to a Shimadzu SPDM20A photodiode array detector. MS detection was performed using Shimadzu LCMS-2010EV mass spectrometer operating in both positive and negative ionization mode. The mobile phase used for acidic mode was a mixture of A = H_2O + 0.1% HCO_2H and B = MeCN + 0.1% HCO_2H. The mobile phase used for basic mode was a mixture of A = H_2O and B = MeCN + 0.4% w/v NH_4HCO_3. The method used is as follows: flow rate: 1.0 mL/min, start 95% A in a linear gradient to 10% A over 4.5 min, hold 1.5 min at 10% A, in 0.5 min in a linear gradient to 95% A, hold 1.5 min at 95% A, total runtime: 8.0 min. Analytical UPLC analyses were performed using a Shimadzu LC-40D liquid chromatograph pump system, equipped with an Xbridge (BEH C18) 2.5 µm column (50 mm, 2.1 mm), connected to a Shimadzu SPD-M40 diode array detector. MS detection was performed using a Shimadzu LCMS-2020 EV mass spectrometer. The mobile phase used for acidic mode was a mixture of A = H_2O + 0.1% HCO_2H and B = MeCN + 0.1% HCO_2H, The method used is as follows: flow rate: flow rate: 0.4 mL/min, start 95% A in a linear gradient to 10% A over 1.75 min, hold 0.25 min at 10% A, in 0.25 min in a linear gradient to 95% A, hold 0.75 min at 95% A, total run time: 3 min. Compound purities were calculated as the percentage peak area of the analysed compound by UV detection at 254 nm. Unless mentioned otherwise, all compounds have a LC purity of >95 %. High-resolution mass

spectra (HRMS) were recorded on a Bruker micrOTOF mass spectrometer using ESI in positive ion mode (HRMS). λ_{max} values provided in the procedures are those extracted from the LC analysis.

General procedure A - Baeyer-Mills reaction

The aniline was dissolved in the indicated volume of PhMe. The solution was purged with N_2 before addition of PhNO (1.1 eq) and glacial AcOH (4.0 eq). The solution was stirred at the indicated temperature until LC-MS indicated full consumption of the aniline (LC-MS). The mixture was diluted with EtOAc and quenched with satd. aq. $NaHCO_3$ solution. The aqueous layer was extracted with three times with EtOAc. The combined organic layers were washed with brine, dried over Na_2SO_4, filtered, and concentrated under reduced pressure. The crude product was purified using column chromatography to give the desired azobenzene.

General procedure B – Deprotection of *N*-Boc moiety

The Boc-protected amine was dissolved in the indicated volume of MeOH. To this was added 4M HCl in 1,4-dioxane (10.0 eq). The solution was stirred at rt until full consumption of the starting material (LC-MS). The solution was poured on ice, and the organic solvent was evaporated under reduced pressure. The aqueous residue was made basic with satd. aq. $NaHCO_3$ solution and was extracted three times with EtOAc. The combined organic layers were washed with brine, dried over Na_2SO_4, and concentrated under reduced pressure. When indicated, the crude product was purified using column chromatography to give the desired product.

General procedure C – Béchamp reduction

The nitro-compound was dissolved in 1,4-dioxane (0.09 M), EtOH (0.11 M) and water (0.17 M). Solid NH_4Cl (3.6 eq) and Fe powder (4.6 eq) were added. The resulting reaction mixture was stirred at 75 °C until full consumption of the starting material (LC-MS). The reaction mixture was diluted with EtOAc, filtered over Celite and washed with EtOAc. The organic solvents were evaporated under reduced pressure and the aqueous residue was extracted three times with EtOAc. The combined organic layers were washed with brine, dried over Na_2SO_4, filtered, and concentrated under reduced pressure to give the desired aniline.

General procedure D – Deprotection of ethylcarbamate moiety

The carbamate (1.0 eq) was dissolved in EtOH:H_2O (4:1, 0.10 M), after which KOH (10.0 eq) was added. The reaction mixture was heated at reflux until full consumption of the starting material (LC-MS). The solvent was evaporated under reduced pressure and the aqueous residue was extracted three times with EtOAc. The combined organic layers were washed with brine, dried over Na_2SO_4, filtered, and concentrated under reduced pressure. The crude product was purified using column chromatography to give the desired product.

tert-butyl 4-(8-chloro-5,6-dihydro-11H-benzo[5,6]cyclohepta[1,2-b]pyridin-11-ylidene)piperidine-1-carboxylate (13)

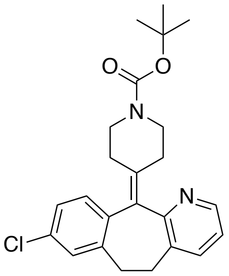

Desloratadine (**8**, 2.00 g, 6.43 mmol, 1.0 eq) was dissolved in DCM (55 mL). To this was added Boc_2O (1.55 g, 7.08 mmol, 1.1 eq) and Et_3N (1.0 mL, 7.19 mmol, 1.1 eq). The solution was stirred at rt for 16 h. The mixture was diluted with DCM (50 mL), and the organic layer was washed with water (3 x 30 mL) and brine. The organic layer was dried over Na_2SO_4, filtered, and concentrated under reduced pressure. This yielded **8** as a white solid (2.50 g, 95%). **^{1}H NMR** (500 MHz, $CDCl_3$) δ 8.39 (dd, *J* = 4.8, 1.6 Hz, 1H), 7.46 – 7.40 (m, 1H), 7.17 – 7.05 (m, 4H), 3.76 (s, 2H), 3.43 – 3.29 (m, 2H), 3.07 (dddd, *J* = 13.2, 9.6, 4.3, 4.3 Hz, 2H), 2.88 – 2.74 (m, 2H), 2.46 (ddd, *J* = 14.2, 9.4, 4.6 Hz, 1H), 2.39 – 2.23 (m, 3H), 1.44 (s, 9H). **^{13}C NMR** (126 MHz, $CDCl_3$) δ 157.3, 154.9, 146.8, 139.7, 137.9, 137.8, 137.6, 134.1, 133.5, 133.0, 130.7, 129.1, 126.3, 122.4, 79.7, 45.4, 44.6, 31.8, 31.6, 30.9, 30.7, 28.6. **LC-MS**: t_r = 4.32 min, purity: > 99%, $[M+H]^+$ m/z calc. 411.15, found 411.18.

11-(1-(tert-butoxycarbonyl)piperidin-4-ylidene)-8-chloro-6,11-dihydro-5H-benzo[5,6]cyclohepta[1,2-b]pyridine 1-oxide (14)

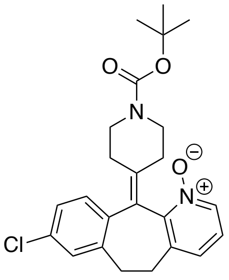

Pyridine **13** (2.50 g, 6.09 mmol, 1.0 eq) was dissolved in DCM (30 mL). To this was added *m*-CPBA (1.58 g, 9.13 mmol, 1.5 eq). The solution was stirred at rt for 50 min. The solution was diluted with DCM and made basic using satd. aq. $NaHCO_3$. The mixture was extracted with DCM (3 x 40 mL). The combined organic layers were washed with brine, dried over Na_2SO_4, filtered, and concentrated under reduced pressure. The crude product was purified using normal-phase column chromatography (0% -> 10% MeOH in EtOAc). This yielded the *N*-oxide **14** as a white solid (1.59 g, 61%). **^{1}H NMR** (500 MHz, CD_3OD) δ 8.17 (dd, *J* = 6.6, 1.2 Hz, 1H), 7.48 (dd, *J* = 7.8, 1.1 Hz, 1H), 7.35 (dd, *J* = 7.8, 6.5 Hz, 1H), 7.32 – 7.27 (m, 1H), 7.20 – 7.14 (m, 2H), 3.71 (dddd, *J* = 17.7, 11.1, 5.1, 5.1 Hz, 2H), 3.48 – 3.22 (m, 4H), 2.99 – 2.86 (m, 2H), 2.48 (ddd, *J* = 13.4, 8.6, 4.5 Hz, 1H), 2.34 (ddd, *J* = 14.2, 6.4, 4.0 Hz, 1H), 2.28 – 2.19 (m, 1H), 1.94 (ddd, *J* = 14.1, 6.3, 3.9 Hz, 1H), 1.46 (s, 9H). **^{13}C NMR** (126 MHz, CD_3OD) δ 156.4, 150.7, 143.0, 140.4, 140.3, 138.5, 134.9, 134.8, 133.6, 131.3, 130.2, 127.0, 126.1, 124.4, 81.3, 45.9, 44.7, 32.8, 31.3, 31.1, 28.7. **LC-MS:** t_r = 4.70 min, purity: > 99%, $[M+H]^+$ m/z calc. 427.20, found 427.17.

tert-butyl 4-(2-amino-8-chloro-5,6-dihydro-11H-benzo[5,6]cyclohepta[1,2-b]pyridin-11-ylidene)piperidine-1-carboxylate (15)

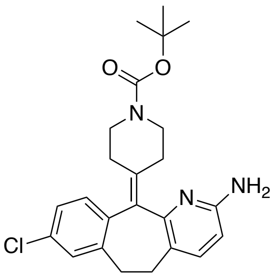

N-oxide **14** (650 mg, 1.52 mmol, 1.0 eq), potassium phthalimide (564 mg, 3.04 mmol, 2.0 eq), and Et_3N (423 µL, 3.04 mmol, 2.0 eq) were dissolved in DCM (1.5 mL). The solution was cooled to 0 ºC. TsCl (580 mg, 3.045 mmol, 2.0 eq) in DCM (6 mL) was added dropwise. The solution was stirred at rt for 20 h. The solution was diluted with water (3 mL) and $H_2NNH_2 \cdot H_2O$ (370 µL, 7.63 mmol, 5.0 eq) was added. The solution was heated at 80 °C for 1.5 h. The mixture was cooled to rt and extracted with DCM (3 x 25 mL). The combined organic layers were washed with 1M NaOH (40 mL). The organic layers were dried over Na_2SO_4, filtered, and concentrated under reduced pressure. The crude product was purified using flash column chromatography (50% -> 80% EtOAc in cHex). This yielded the title compound as a white solid (405 mg, 63%). **^{1}H NMR** (500 MHz, $CDCl_3$) δ 7.18 (d, *J* = 8.2 Hz, 1H), 7.16 – 7.14 (m, 1H), 7.14 – 7.08 (m, 2H), 6.34 (d, *J* = 8.3 Hz, 1H), 4.38 (s, 2H), 3.94 – 3.70 (m, 2H), 3.31 (ddd, *J* = 12.9, 9.0, 4.4 Hz, 1H), 3.23 – 3.13 (m, 1H), 3.04 – 2.92 (m, 2H), 2.78 – 2.64 (m, 2H), 2.50 – 2.39 (m,

2H), 2.32 – 2.26 (m, 2H), 1.45 (s, 9H). **^{13}C NMR** (126 MHz, $CDCl_3$) δ 155.9, 155.0, 154.5, 140.2, 140.0, 138.2, 137.6, 134.1, 132.8, 130.4, 129.0, 126.1, 122.9, 107.7, 79.7, 45.5, 44.4, 32.1, 31.0, 30.8, 28.6. **LC-MS:** t_r = 3.98 min, purity: >99%, $[M+H]^+$ m/z calc. 426.18, found 426.20.

***tert*-butyl (*E*)-4-(8-chloro-2-(phenyldiazenyl)-5,6-dihydro-11*H*-benzo[5,6]cyclohepta[1,2-*b*]pyridin-11-ylidene)piperidine-1-carboxylate (16)**

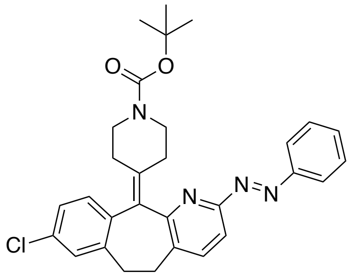

This compound was synthetised according to general procedure **A** using aniline **15** (600 mg, 1.41 mmol, 1.0 eq), PhNO (181 mg, 1.69 mmol, 1.2 eq), AcOH (324 µL, 5.63 mmol, 4.0 eq), PhMe (14.6 mL) and a reaction time of 18h. The crude product was purified using reverse-phase column chromatography (5->95% MeCN + 0.1% H_2COH in H_2O + 0.1% H_2COH). The desired fractions were combined, made basic with satd. aq. $NaHCO_3$ and extracted three times with EtOAc. The combined organic layers were washed with brine, dried over Na_2SO_4, filtered, and concentrated under reduced pressure. This gave **16** as an orange solid (61 mg, 8%). **^{1}H NMR** (600 MHz, $CDCl_3$) δ 8.06 – 8.00 (m, 2H), 7.61 (d, *J* = 8.2 Hz, 1H), 7.58 (d, *J* = 8.1 Hz, 1H), 7.55 – 7.49 (m, 3H), 7.23 – 7.18 (m, 2H), 7.16 (dd, *J* = 8.1, 2.2 Hz, 1H), 3.98 – 3.68 (m, 2H), 3.49 – 3.39 (m, 2H), 3.06 (ddd, *J* = 13.3, 9.9, 3.7 Hz, 2H), 2.96 (ddd, *J* = 16.2, 10.2, 4.5 Hz, 1H), 2.84 (ddd, *J* = 16.1, 8.0, 4.5 Hz, 1H), 2.64 – 2.57 (m, 1H), 2.49 – 2.43 (m, 1H), 2.40 – 2.31 (m, 2H), 1.45 (s, 9H). **^{13}C NMR** (151 MHz, $CDCl_3$) δ 161.4, 156.7, 155.0, 152.4, 140.0, 139.7, 139.0, 137.9, 135.8, 133.3, 133.1, 132.1, 131.0, 129.2, 129.0, 126.4, 123.8, 110.5, 79.7, 31.8, 31.7, 29.8, 28.6. **LC-MS:** t_r = 6.58 min, purity: 97%, λ_{max}: 331 nm, $[M+H]^+$ m/z calc. 515.22, found 515.30.

(*E*)-8-chloro-2-(phenyldiazenyl)-11-(piperidin-4-ylidene)-6,11-dihydro-5*H*-benzo[5,6]cyclohepta[1,2-*b*]pyridine (10a)

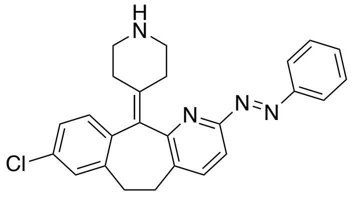

This compound was synthetised according to general procedure **B** using Boc-protected amine **16** (55 mg, 0.11 mmol, 1.0 eq), 4M HCl in 1,4-dioxane (0.27 mL, 1.07 mmol, 10.0 eq), MeOH (1.1 mL) and a reaction time of 17h. The crude product was purified using reverse-phase column chromatography (5->90% MeCN + 0.1% H_2COH in H_2O + 0.1% H_2COH). The desired fractions were combined, made basic with satd. aq. $NaHCO_3$ and extracted three times with EtOAc. The combined organic layers were washed with brine, dried over Na_2SO_4, filtered, and concentrated under reduced pressure. This gave **10a** as an orange solid (7 mg, 16%). **^{1}H NMR** (600 MHz, $(CD_3)_2SO$) δ 7.94 – 7.90 (m, 2H), 7.82 (d, *J* = 8.1 Hz, 1H), 7.64 – 7.59 (m, 3H), 7.55 (d, *J* = 8.0 Hz, 1H), 7.32 (d, *J* = 2.3 Hz, 1H), 7.24 (dd, *J* = 8.2, 2.3 Hz, 1H), 7.15 (d, *J* = 8.2 Hz, 1H), 3.41 – 3.33 (m, 3H), 3.03 – 2.80 (m, 4H), 2.63 – 2.53 (m, 2H), 2.37 – 2.28 (m, 1H), 2.28 – 2.14 (m, 3H). **^{13}C NMR** (151 MHz, $(CD_3)_2SO$) δ 160.6, 156.9, 152.0, 140.3, 140.3, 139.8, 137.9, 136.3, 132.5, 131.8, 131.1, 131.0, 129.8, 129.1, 125.9, 123.1, 111.4, 47.5, 47.4, 32.1, 32.0, 31.0, 30.7. **LC-MS:** t_r = 3.95 min, purity: 97%, λ_{max}: 334 nm, $[M+H]^+$ m/z calc. 415.17, found 415.15. **HRMS**: $[M+H]^+$ calc. for $C_{25}H_{24}ClN_4$: 415.1684, found 415.1694.

tert-butyl 4-(4-amino-8-chloro-5,6-dihydro-11H-benzo[5,6]cyclohepta[1,2-b]pyridin-11-ylidene)piperidine-1-carboxylate (17)

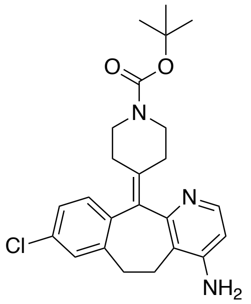

N-oxide **14** (950 mg, 2.31 mmol, 1.0 eq) and 4-cyanopyridine (481 mg, 4.62 mmol, 2.0 eq) were dissolved in MeCN (10 mL). The mixture was cooled to 0 °C. Tf_2O (1M in DCM, 2.3 mL, 2.3 mmol, 1.0 eq) was added. The solution was warmed to rt and stirred for 2 h. Conc. aq. NH_4OH (28-30 wt%) was added (1.0 mL, 15 mmol, 6.4 eq) and the solution was stirred at rt for 16 h. The mixture was diluted with water and the pH was adjusted to 8 using 2M HCl. The reaction mixture was extracted with DCM (3 x 30 mL). The combined organic layers were washed with brine, dried over Na_2SO_4, filtered, and concentrated under reduced pressure. The crude product was purified twice with normal-phase column chromatography (40% -> 75% EtOAc:MeOH:Et_3N (90:5:5) in cHex, followed by 0% -> 10% MeOH in DCM). This yielded **17** (247 mg, 25%) as a brown solid. **^{1}H NMR** (500 MHz, $CDCl_3$) δ 8.03 (d, *J* = 5.5 Hz, 1H), 7.21 (d, *J* = 2.0 Hz, 1H), 7.17 – 7.09 (m, 2H), 6.45 (d, *J* = 5.6 Hz, 1H), 4.28 (s, 2H), 3.92 – 3.65 (m, 2H), 3.44 (ddd, *J* = 14.1, 12.2, 4.2 Hz, 1H), 3.04 (m, 2H), 2.91 (ddd, *J* = 16.1, 4.7, 4.7 Hz, 1H), 2.80 (ddd, *J* = 14.1, 4.8, 4.8 Hz, 1H), 2.58 – 2.45 (m, 2H), 2.40 (ddd, *J* = 14.3, 4.5, 4.5 Hz, 1H), 2.32 – 2.16 (m, 2H), 1.44 (s, 9H). **^{13}C NMR** (126 MHz, $CDCl_3$) δ 155.0, 152.2, 145.7, 140.0, 139.6, 138.4, 132.9, 129.7, 128.1, 126.6, 116.5, 108.7, 80.1, 44.2, 31.0, 30.9, 30.7, 28.6, 26.9. Two missing quaternary carbons **LC-MS**: t_r = 3.69 min, purity: >99%, $[M+H]^+$ m/z calc. 426.19, found 426.15.

tert-butyl (E)-4-(8-chloro-4-(phenyldiazenyl)-5,6-dihydro-11H-benzo[5,6]cyclohepta[1,2-b]pyridin-11-ylidene)piperidine-1-carboxylate (18)

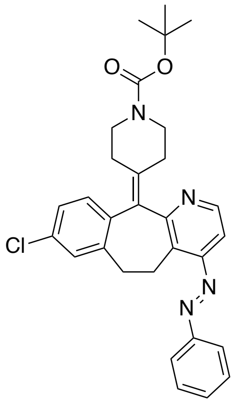

Aniline **17** (247 mg, 0.58 mmol, 1.0 eq) was dissolved in THF (2.5 mL). NaH (60% dispersion in mineral oil, 50 mg, 2.1 mmol, 3.6 eq) and PhNO (124 mg, 1.16 mmol, 2.0 eq) in THF (2.5 mL) were added. The mixture was stirred for 72 h at rt. The crude product was purified using reverse-phase column chromatography (5->90% MeCN + 0.1% H_2COH in H_2O + 0.1% H_2COH). The desired fractions were combined, made basic with satd. aq. $NaHCO_3$ and extracted three times with EtOAc. The combined organic layers were washed with brine, dried over Na_2SO_4, filtered, and concentrated under reduced pressure. This yielded **18** (70 mg, 23%) as an orange oil. **^{1}H NMR** (500 MHz, $CDCl_3$) δ 8.54 (d, *J* = 5.4 Hz, 1H), 7.99 – 7.91 (m, 2H), 7.61 – 7.50 (m, 3H), 7.30 (d, *J* = 5.4 Hz, 1H), 7.20 (d, *J* = 13.0 Hz, 3H), 3.96 – 3.71 (m, 2H), 3.63 (ddd, *J* = 16.8, 9.4, 4.7 Hz, 1H), 3.56 – 3.45 (m, 2H), 3.13 (ddd, *J* = 13.1, 9.4, 3.8 Hz, 2H), 2.93 (ddd, *J* = 15.2, 8.5, 4.7 Hz, 1H), 2.64 – 2.53 (m, 1H), 2.49 – 2.27 (m, 3H), 1.46 (s, 9H). **^{13}C NMR** (126 MHz, $CDCl_3$) δ 157.5*, 154.9, 152.8, 139.5, 136.8, 133.5, 132.9, 132.1*, 131.1, 129.5, 129.3, 126.6, 123.8, 109.0, 79.8, 45.1*, 31.9, 31.0, 28.6, 26.0. Four carbon signals could not be identified despite extensive 2D-NMR analysis (three quaternary carbon atoms, one tertiary aromatic carbon atom). **LC-MS**: t_r = 6.28 min, purity: >99%, λ_{max}: 316 nm, $[M+H]^+$ m/z calc. 515.20, found 515.30.

(E)-8-chloro-4-(phenyldiazenyl)-11-(piperidin-4-ylidene)-6,11-dihydro-5H-benzo[5,6]cyclohepta[1,2-b]pyridine (10c)

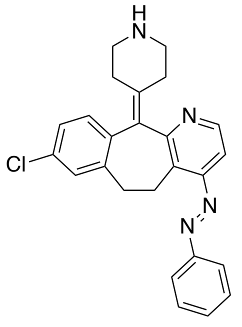

This compound was synthetised according to General procedure **B** using Boc-protected amine **18** (70 mg, 0.14 mmol, 1.0 eq), HCl in 1,4-dioxane (0.34 mL, 1.36 mmol, 10 eq), MeOH (1.4 mL) and a reaction time of 22h. The crude product was purified using flash column chromatography (3%-> 6% MeOH + NH_3 in DCM). Compound **10c** was obtained as an orange solid (35 mg, 62%) **^{1}H NMR** (500 MHz, $CDCl_3$) δ 8.51 (d, *J* = 5.2 Hz, 1H), 7.98 – 7.88 (m, 2H), 7.59 – 7.49 (m, 3H), 7.21 (d, *J* = 5.3 Hz, 1H), 7.19 – 7.16 (m, 2H), 7.14 (dd, *J* = 7.8, 2.1 Hz, 1H), 3.70 – 3.60 (m, 1H), 3.55 – 3.44 (m, 2H), 3.13 – 3.05 (m, 2H), 2.93 (ddd, *J* = 15.4, 9.6, 4.6 Hz, 1H), 2.75 (dddd, *J* = 15.6, 12.2, 9.5, 3.7 Hz, 2H), 2.51 – 2.32 (m, 4H). **^{13}C NMR** (126 MHz, $CDCl_3$) δ 160.8, 155.9, 152.8, 147.8, 139.7, 139.5, 137.3, 133.0, 132.7, 132.4, 131.1, 131.0, 129.4, 129.4, 126.2, 123.6, 108.4, 48.2, 48.1, 32.6, 31.9, 25.7. **LC-MS:** t_r = 3.94 min, purity 99%, λ_{max}: 317 nm, $[M+H]^+$ m/z calc. 415.16, found 415.15. **HRMS**: $[M+H]^+$ calc. for $C_{25}H_{24}ClN_4O_2$: 415.1684, found 415.1688.

Ethyl 4-(8-chloro-3-nitro-5,6-dihydro-11H-benzo[5,6]cyclohepta[1,2-b]pyridin-11-ylidene)piperidine-1-carboxylate (19)

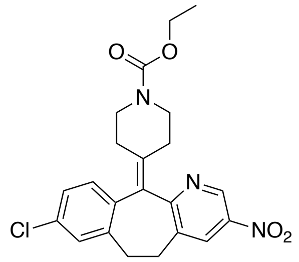

Loratadine (**7**, 2.00 g, 5.22 mmol, 1.0 eq) was dissolved in DCM (15 mL). The solution was cooled to -5 °C. A solution of $Bu_4N^+NO_3^-$ (1.84 g, 6.04 mmol, 1.16 eq) and TFAA (0.85 mL, 6.0 mmol, 1.2 eq) in DCM (8 mL) was added dropwise, keeping the temperature below 0 °C. The solution was stirred for 2 h at 0 °C, after which the solution was warmed to rt and stirred for 66 h. The mixture was quenched with satd. aq. $NaHCO_3$ and the aqueous layer was extracted three times with DCM. The combined organic layers were washed with brine, dried over Na_2SO_4, filtered, and concentrated under reduced pressure. The crude product was purified using normal-phase column chromatography (10% -> 30% EtOAc in cHex). This yielded **19** (473 mg, 21%) as a yellow solid. **^{1}H NMR** (600 MHz, $CDCl_3$) δ 9.21 (d, *J* = 2.5 Hz, 1H), 8.24 (d, *J* = 2.4 Hz, 1H), 7.20 (d^{Δ}, *J* = 2.1 Hz, 1H), 7.17 (dd, *J* = 8.1, 2.2 Hz, 1H), 7.10 (d^{Δ}, *J* = 8.1 Hz, 1H), 4.14 (q, *J* = 7.1 Hz, 2H), 3.85 – 3.66 (m, 2H), 3.49 – 3.40 (m, 2H), 3.22 (dddd, *J* = 12.8, 8.8, 8.8, 3.9 Hz, 2H), 3.01 (ddd, *J* = 16.9, 9.9, 4.7 Hz, 1H), 2.85 (ddd, *J* = 15.2, 8.2, 4.6 Hz, 1H), 2.53 – 2.45 (m, 1H), 2.43 – 2.35 (m, 1H), 2.35 – 2.28 (m, 1H), 2.28 – 2.21 (m, 1H), 1.25 (t, *J* = 7.1 Hz, 3H). **^{13}C NMR** (151 MHz, $CDCl_3$) δ 162.9, 155.6, 143.0, 142.1, 140.4, 139.1, 136.5, 134.7, 133.8, 132.9, 132.6, 130.9, 129.2, 126.8, 61.6, 44.9, 44.8, 31.8, 31.2, 14.8. **LC-MS:** t_r = 5.38 min, purity >99%, $[M+H]^+$ m/z calc. 428.13, found 428.15.

Ethyl 4-(3-amino-8-chloro-5,6-dihydro-11H-benzo[5,6]cyclohepta[1,2-b]pyridin-11-ylidene)piperidine-1-carboxylate (20)

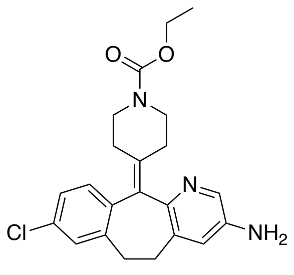

This compound was synthetised according to general procedure **C** using nitro-compound **19** (473 mg, 1.11 mmol, 1.0 eq), Fe (278 mg, 4.97 mmol, 4.5 eq), NH_4Cl (213 mg, 3.98 mmol, 3.6 eq), 1,4-dioxane (13 mL), EtOH (8 mL), water (6.5 mL), and a reaction time of 3h. This yielded the title compound as a brown oil (387 mg, 88%). **^{1}H NMR** (500 MHz, $(CD_3)_2SO$) δ 7.71 (d, *J* = 2.6 Hz, 1H), 7.29 (d, *J* = 2.3 Hz, 1H), 7.19 (dd, *J* = 8.1, 2.3 Hz, 1H), 7.02 (d^{Δ}, *J* = 8.1 Hz, 1H), 6.66 (d, *J* = 2.6 Hz, 1H), 4.03 (qd, *J* = 7.1, 1.7 Hz, 2H), 3.60 (ddd, *J* = 12.7, 5.2, 5.2 Hz, 2H), 3.31 – 3.08 (m, 4H), 2.78 – 2.61 (m, 2H), 2.36 – 2.08 (m, 4H), 1.22 – 1.12 (m, 3H). **LC-MS:** t_r = 3.59 min, purity >99%, $[M+H]^+$ m/z calc. 398.16, found 398.10.

Ethyl (E)-4-(8-chloro-3-(phenyldiazenyl)-5,6-dihydro-11H-benzo[5,6]cyclohepta[1,2-b]pyridin-11-ylidene)piperidine-1-carboxylate (21)

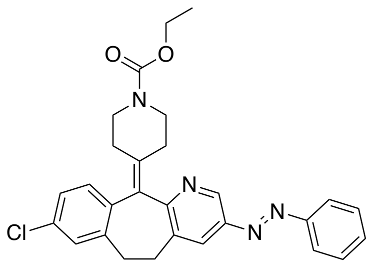

This compound was synthetised according to general procedure **A** using aminopyridine **20** (0.387 g, 0.973 mmol, 1.0 eq), AcOH (0.22 mL, 3.9 mmol, 4.0 eq), PhNO (104 mg, 0.97 mmol, 1.0 eq), PhMe (10 mL) and a reaction time of 25h. The crude product was purified using flash column chromatography (10% -> 30% EtOAc in cHex). This yielded **21** as an orange solid (152 mg, 32%). **^{1}H NMR** (600 MHz, $CDCl_3$) δ 9.01 (d, *J* = 2.2 Hz, 1H), 7.98 – 7.94 (m, 1H), 7.93 – 7.89 (m, 2H), 7.57 – 7.48 (m, 3H), 7.21 – 7.19 (m, 1H), 7.19 – 7.16 (m, 2H), 4.14 (q, *J* = 7.1 Hz, 2H), 3.94 – 3.74 (m, 2H), 3.53 – 3.37 (m, 2H), 3.17 (m, 2H), 3.00 (ddd, *J* = 15.7, 9.6, 4.6 Hz, 1H), 2.87 (ddd, *J* = 15.2, 8.4, 4.6 Hz, 1H), 2.63 – 2.51 (m, 1H), 2.46 – 2.31 (m, 3H), 1.26 (t, *J* = 7.1 Hz, 3H). **^{13}C NMR** (151 MHz, $CDCl_3$) δ 159.3, 155.6, 152.6, 147.0, 144.2, 139.4, 139.1, 137.1, 134.7, 133.4, 131.9, 130.9, 129.4, 129.2, 128.5, 126.6, 123.2, 61.5, 44.9, 31.8, 31.6, 31.0, 14.8. One carbon signal is not visible (quaternary carbon atom). **LC-MS:** t_r = 6.29 min, purity 96%, λ_{max}: 339 nm, $[M+H]^+$ m/z calc. 487.18, found 487.20.

(E)-8-chloro-3-(phenyldiazenyl)-11-(piperidin-4-ylidene)-6,11-dihydro-5H-benzo[5,6]cyclohepta[1,2-b]pyridine (10b)

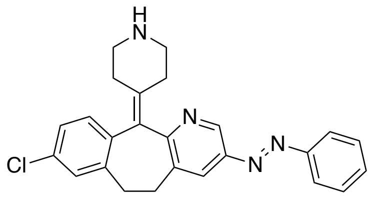

This compound was synthetised according to General procedure **D** using protected amine **21** (60 mg, 0.12 mmol, 1 eq), KOH (270 mg, 4.81 mmol, 39.0 eq), EtOH (4 mL), water (1 mL) and a reaction time of 4d. The crude product was purified using reverse-phase column chromatography (5->90% MeCN + 0.1% H_2COH in H_2O + 0.1% H_2COH). The desired fractions were combined, made basic with satd. aq. $NaHCO_3$ and extracted three times with EtOAc. The combined organic layers were washed with brine, dried over Na_2SO_4, filtered, and concentrated under reduced pressure. This gave **10b** as an orange solid (17 mg, 33%). **^{1}H NMR** (600 MHz, $(CD_3)_2SO$) δ 8.91 (d, *J* = 2.3 Hz, 1H), 8.00 (d, *J* = 2.3 Hz, 1H), 7.91 – 7.88 (m, 2H), 7.63 – 7.57 (m, 3H), 7.32 (d^{Δ}, *J* = 2.3 Hz, 1H), 7.23 (dd, *J* = 8.2, 2.3 Hz, 1H), 7.11 (d^{Δ}, *J* = 8.2 Hz, 1H), 3.44 – 3.35 (m, 2H), 2.99 (ddd, *J* = 16.7, 9.1, 4.7 Hz, 1H), 2.93 – 2.85 (m, 3H), 2.60 (dddd, *J* = 15.0, 12.2, 8.9, 3.5 Hz, 2H), 2.33 – 2.21 (m, 2H), 2.20 – 2.12 (m, 2H). **^{13}C NMR** (151 MHz, $(CD_3)_2SO$) δ 160.2, 152.0, 146.2, 143.8, 140.4, 140.1, 137.3, 134.6, 132.0, 131.6, 131.1, 131.0, 129.6, 129.1, 127.4, 125.8, 122.7, 47.7, 47.6, 32.3, 32.2, 30.9, 30.6. **LC-MS:** t_r = 4.02 min, purity 98%, λ_{max}: 337 nm, $[M+H]^+$ m/z calc. 415.16, found 415.15. **HRMS**: $[M+H]^+$ calc. for $C_{25}H_{24}ClN_4$: 415.1684, found 415.1689.

ethyl 4-(8-((tert-butoxycarbonyl)amino)-5,6-dihydro-11H-benzo[5,6]cyclohepta[1,2-b]pyridin-11-ylidene)piperidine-1-carboxylate (22)

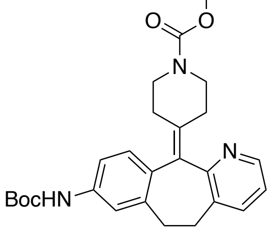

Loratadine (**7**, 500 mg, 1.31 mmol, 1.0 eq) was dissolved in 1,4-dioxane (12 mL). XPhos (224 mg, 0.47 mmol, 0.36 eq), $Pd(OAc)_2$ (35 mg, 0.16 mmol, 0.12 eq), $BocNH_2$ (184 mg, 1.57 mmol, 1.2 eq) and Cs_2CO_3 (596 mg, 1.83 mmol, 1.4 eq) were added. The reaction mixture was stirred at 95 °C for 2 h, after which it was cooled to rt. The reaction mixture was diluted with EtOAc and filtered over Celite. The filtrate was diluted with water and extracted three times with EtOAc. The combined organic layers were washed with brine, dried over Na_2SO_4, filtered, and concentrated under reduced pressure. The crude product was purified using

normal-phase column chromatography (30 -> 60 % EtOAc:MeOH:Et_3N (90:5:5) in cHex). This yielded **22** as an off-white solid (499 mg, 82%). **^{1}H NMR** (500 MHz, $CDCl_3$) δ 8.38 (dd, *J* = 4.8, 1.7 Hz, 1H), 7.43 (dd, *J* = 7.6, 1.7 Hz, 1H), 7.39 – 7.33 (m, 1H), 7.12 – 7.05 (m, 2H), 6.98 (dd, *J* = 8.2, 2.3 Hz, 1H), 6.48 (s, 1H), 4.13 (q, *J* = 7.1 Hz, 2H), 3.88 – 3.72 (m, 2H), 3.44 – 3.28 (m, 2H), 3.16 – 3.05 (m, 2H), 2.90 – 2.76 (m, 2H), 2.47 (ddd, *J* = 14.2, 9.5, 4.6 Hz, 1H), 2.40 – 2.24 (m, 3H), 1.50 (s, 9H), 1.24 (t, *J* = 7.1 Hz, 3H). **^{13}C NMR** (126 MHz, $CDCl_3$) δ 157.8, 155.7, 152.9, 146.5, 138.6, 137.7, 137.6, 136.9, 134.1, 133.9, 130.0, 122.2, 119.2, 116.4, 80.7, 61.4, 45.0, 45.0, 32.2, 31.7, 30.9, 30.7, 28.5, 14.8. **LC-MS**: t_r = 3.74 min, purity: >99%, [M+H]+ m/z calc. 464.30, found 464.25.

ethyl 4-(8-amino-5,6-dihydro-11H-benzo[5,6]cyclohepta[1,2-b]pyridin-11-ylidene)piperidine-1-carboxylate (23)

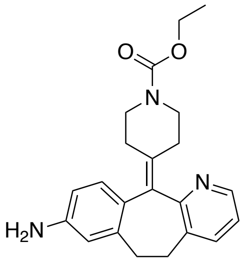

This compound was synthetised according to general procedure **B** using protected amine **22** (486 mg, 1.05 mmol, 1.0 eq), HCl in dioxane (2.6 mL, 10.50 mmol, 10.0 eq), MeOH (15 mL) and a reaction time of 17h. This gave **23** (375 mg, 98%) as a yellow powder. **^{1}H NMR** (500 MHz, $CDCl_3$) δ 8.38 (dd, *J* = 4.8, 1.7 Hz, 1H), 7.44 (dd, *J* = 7.7, 1.7 Hz, 1H), 7.07 (dd, *J* = 7.6, 4.8 Hz, 1H), 7.00 – 6.95 (m, 1H), 6.52 – 6.47 (m, 2H), 4.13 (q, *J* = 7.1 Hz, 2H), 3.91 – 3.68 (m, 2H), 3.38 – 3.28 (m, 2H), 3.17 – 3.04 (m, 2H), 2.86 – 2.67 (m, 2H), 2.50 – 2.33 (m, 3H), 2.27 (ddd, *J* = 14.6, 4.5 Hz, 1H), 1.24 (t, *J* = 7.1 Hz, 3H). **^{13}C NMR** (126 MHz, $CDCl_3$) δ 158.5, 155.7, 146.3, 145.8, 138.6, 137.4, 134.0, 130.7, 129.3, 122.1, 115.6, 113.0, 61.4, 45.1, 45.0, 32.3, 31.7, 30.8, 30.7, 14.8. Two carbon signal nots are not visible (quaternary carbon atoms). **LC-MS**: t_r = 2.58 min, purity: >99%, $[M+H]^+$ m/z calc. 364.19, found 364.15.

ethyl (E)-4-(8-(phenyldiazenyl)-5,6-dihydro-11H-benzo[5,6]cyclohepta[1,2-b]pyridin-11-ylidene)piperidine-1-carboxylate (24)

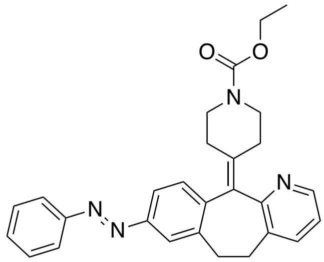

This compound was synthetised according to general procedure **A** using aniline **23** (375 mg, 1.03 mmol, 1.0 eq), PhNO (173 mg, 1.62 mmol, 1.6 eq), AcOH (0.24 mL, 4.1 mmol, 4.0 eq), PhMe (10 mL) and a reaction time of 72h. The crude product was purified using normal-phase column chromatography (5% -> 40% EtOAc:MeOH:Et_3N (90:5:5) in cHex). This gave **24** as an orange solid (170 mg, 36%). **^{1}H NMR** (600 MHz, $CDCl_3$) δ 8.43 (dd, *J* = 4.8, 1.6 Hz, 1H), 7.91 – 7.85 (m, 2H), 7.77 – 7.71 (m, 2H), 7.54 – 7.42 (m, 4H), 7.36 (d^{Δ}, *J* = 8.6 Hz, 1H), 7.13 (dd, *J* = 7.7, 4.8 Hz, 1H), 4.14 (q, *J* = 7.1 Hz, 2H), 3.92 – 3.76 (m, 2H), 3.56 – 3.48 (m, 1H), 3.41 (ddd, *J* = 15.9, 8.6, 4.6 Hz, 1H), 3.17 (ddd, *J* = 13.1, 9.0, 3.7 Hz, 2H), 3.01 – 2.89 (m, 2H), 2.54 (ddd, *J* = 14.0, 9.4, 4.6 Hz, 1H), 2.47 – 2.31 (m, 3H), 1.25 (t, *J* = 7.1 Hz, 3H). **^{13}C NMR** (151 MHz, $CDCl_3$) δ 156.9, 155.7, 152.8, 152.1, 146.7, 142.4, 138.9, 138.2, 138.0, 134.6, 133.8, 131.1, 130.3, 129.2, 123.2, 122.9, 122.5, 121.2, 61.5, 45.0, 44.9, 32.0, 31.9, 31.0, 30.8, 14.8. **LC-MS**: t_r = 4.48 min, purity: 98%, $[M+H]^+$ m/z calc. 453.22 found 453.25, λ_{max}: 329 nm. **HRMS**: $[M+H]^+$ calc. for $C_{25}H_{25}N_4$: 381.2074, found 381.2076.

(E)-8-(phenyldiazenyl)-11-(piperidin-4-ylidene)-6,11-dihydro-5H-benzo[5,6]cyclohepta[1,2-b]pyridine (11b)

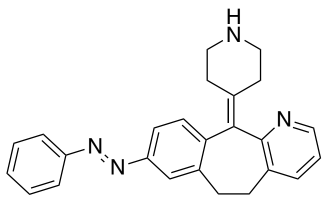

This compound was synthetised according to general procedure **D** using protected amine **24** (113 mg, 0.25 mmol, 1.0 eq), KOH (140 mg, 2.50 mmol, 10.0 eq), EtOH (8 mL), water (2 mL) and a reaction time of 100h. The crude product was purified using normal-phase column chromatography (3.5% -> 6.5% MeOH + NH_3 in DCM). This yielded **11b**

as an orange solid (58 mg, 61%). **^{1}H NMR** (500 MHz, $CDCl_3$) δ 8.41 (dd, *J* = 4.8, 1.7 Hz, 1H), 7.91 – 7.85 (m, 2H), 7.76 – 7.69 (m, 2H), 7.50 (ddd, *J* = 8.3, 6.4, 1.8 Hz, 2H), 7.49 – 7.42 (m, 3H), 7.38 – 7.32 (m, 1H), 7.09 (dd, *J* = 7.7, 4.8 Hz, 1H), 3.54 (ddd, *J* = 13.2, 8.7, 4.5 Hz, 1H), 3.43 (ddd, *J* = 15.6, 7.0, 4.3 Hz, 1H), 3.10 (dddd, *J* = 16.4, 12.0, 4.8, 4.7 Hz, 2H), 3.01 – 2.86 (m, 2H), 2.75 (dddd, *J* = 13.0, 9.4, 4.2, 4.2 Hz, 2H), 2.53 – 2.34 (m, 4H). **^{13}C NMR** (126 MHz, $CDCl_3$) δ 157.4, 152.9, 152.0, 146.8, 142.6, 139.1, 138.9, 137.6, 133.8, 133.6, 131.0, 130.4, 129.2, 123.3, 122.9, 122.3, 121.0, 48.1, 48.1, 32.6, 32.3, 32.1, 31.8. **LC-MS**: t_r = 3.29 min, purity > 99%, $[M+H]^+$ calculated 381.21, found 381.20, λ_{max}: 329 nm. **HRMS**: $[M+H]^+$ calc. for $C_{28}H_{29}N_4$: 381.2079, found 381.2076.

ethyl 4-(8-chloro-9-nitro-5,6-dihydro-11*H*-benzo[5,6]cyclohepta[1,2-*b*]pyridin-11-ylidene)piperidine-1-carboxylate (25) and ethyl 4-(8-chloro-7-nitro-5,6-dihydro-11*H*-benzo[5,6]cyclohepta[1,2-*b*]pyridin-11-ylidene)piperidine-1-carboxylate (28)

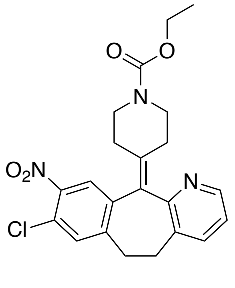

25
major

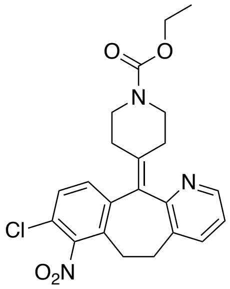

28
minor

Loratadine (**7**, 6.28 g, 16.4 mmol, 1.0 eq) was dissolved in conc. H_2SO_4 (30 mL). The solution was cooled between -10 °C and -5 °C. To this solution was slowly added solid KNO_3 (1.66 g, 16.4 mmol, 1.0 eq), while keeping the temperature between -10 °C and -5 °C. The mixture was stirred for 3 h in this temperature range, after which it was warmed to rt and stirred for 17 h at rt. The mixture was poured onto crushed ice and made basic using aq. NH_4OH (28-30 wt%). The resulting mixture was extracted three times with DCM. The combined organic layers were washed with brine, dried over Na_2SO_4, filtered, and concentrated under reduced pressure. The two products were separated using flash column chromatography (20% -> 100% EtOAc in cHex). Compound **25** (5.665 g, 81%) was obtained as a white solid and compound **28** (640 mg, 9%) as a yellow solid.

Compound **25** (major): **^{1}H NMR** (500 MHz, $CDCl_3$) δ 8.43 (dd, *J* = 4.8, 1.7 Hz, 1H), 7.79 – 7.73 (m, 1H), 7.48 (dd, *J* = 7.7, 1.7 Hz, 1H), 7.40 – 7.34 (m, 1H), 7.15 (dd, *J* = 7.7, 4.8 Hz, 1H), 4.14 (q, *J* = 7.1 Hz, 2H), 3.89 – 3.75 (m, 2H), 3.55 – 3.32 (m, 2H), 3.17 (dddd, *J* = 18.5, 13.3, 9.3, 3.9 Hz, 2H), 2.88 (dddd, *J* = 14.0, 10.5, 8.4, 5.4 Hz, 2H), 2.52 (ddd, *J* = 14.1, 9.4, 4.7 Hz, 1H), 2.42 (ddd, *J* = 14.0, 9.3, 4.7 Hz, 1H), 2.35 – 2.24 (m, 2H), 1.25 (t, *J* = 7.1 Hz, 3H).**^{13}C NMR** (126 MHz, $CDCl_3$) δ 156.1, 155.6, 147.2, 145.6, 144.5, 140.0, 138.9, 137.8, 133.1, 132.5, 132.2, 126.6, 125.8, 122.9, 61.6, 44.7, 31.8, 31.0, 30.9, 30.7, 14.8. **LC-MS:** t_r = 4.00 min, purity 95%, $[M+H]^+$ m/z calc. 428.13, found 428.10.

Compound **28** (minor): **^{1}H NMR** (500 MHz, $CDCl_3$) δ 8.43 (dd, *J* = 4.7, 1.7 Hz, 1H), 7.47 (d^{Δ}, *J* = 7.5 Hz, 1H), 7.33 (d, *J* = 8.3 Hz, 1H), 7.27 (d, *J* = 8.3 Hz, 1H), 7.16 (dd, *J* = 7.7, 4.8 Hz, 1H), 4.14 (q, *J* = 7.1 Hz, 2H), 3.89 – 3.76 (m, 2H), 3.40 – 3.28 (m, 2H), 3.18 (dddd, *J* = 13.1, 9.3, 6.8, 3.9 Hz, 2H), 2.96 (ddd, *J* = 13.8, 13.1, 5.5 Hz, 1H), 2.76 (ddd, *J* = 15.1, 7.6, 4.4 Hz, 1H), 2.53 (ddd, *J* = 14.1, 9.4, 4.6 Hz, 1H), 2.42 – 2.23 (m, 3H), 1.25 (t, *J* = 7.1 Hz, 3H). **^{13}C NMR** (126 MHz, $CDCl_3$) δ 155.6, 155.4, 149.8, 147.0, 140.6, 138.4, 132.9, 131.6, 131.1, 128.3, 123.7, 122.9, 61.6, 44.8, 31.1, 30.8, 27.0, 14.8. A signal for one quaternary carbon is missing. **LC-MS:** t_r = 4.14 min, purity 99%, $[M+H]^+$ m/z calc. 428.13, found 428.10.

8-chloro-9-nitro-11-(piperidin-4-ylidene)-6,11-dihydro-5H-benzo[5,6]cyclohepta[1,2-b]pyridine (26)

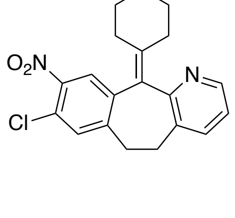

Protected amine **25** (3.00 g, 7.01 mmol, 1.0 eq) was dissolved in conc. HCl (12.0 mL, 139 mmol, 19.9 eq). The solution was heated at 80 °C for 24 h. The mixture was made basic with aq. NH_4OH (28-30 wt%). The resulting mixture was extracted three times with DCM. The combined organic layers were washed with brine, dried over Na_2SO_4, filtered, and concentrated under reduced pressure.

This yielded **26** as a yellow solid (2.23 g, 89%). **^{1}H NMR** (500 MHz, CD_3OD) δ 8.36 (dd, J = 4.9, 1.6 Hz, 1H), 7.75 – 7.71 (m, 1H), 7.69 (dd, J = 7.8, 1.6 Hz, 1H), 7.53 – 7.49 (m, 1H), 7.29 (dd, J = 7.7, 4.9 Hz, 1H), 3.54 – 3.40 (m, 2H), 3.06 – 2.88 (m, 4H), 2.69 (dddd, J = 33.3, 12.7, 9.4, 3.7 Hz, 2H), 2.47 – 2.28 (m, 3H), 2.24 – 2.15 (m, 1H). **^{13}C NMR** (126 MHz, CD_3OD) δ 157.9, 147.5, 146.9, 146.1, 139.8, 139.6, 135.7, 133.9, 131.5, 127.3, 126.0, 124.5, 48.2, 48.0, 32.9, 32.8, 32.7, 31.3. **LC-MS:** t_r = 2.86 min, purity 98%, $[M+H]^+$ m/z calc. 356.11, found 356.10.

tert-butyl 4-(8-chloro-9-nitro-5,6-dihydro-11H-benzo[5,6]cyclohepta[1,2-b]pyridin-11-ylidene)piperidine-1-carboxylate (27)

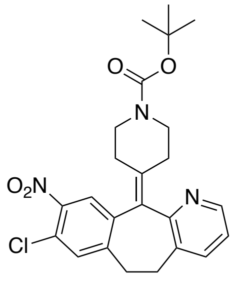

Amine **26** (1.87 g, 5.26 mmol, 1.0 eq) was dissolved in DCM (48 mL). Et_3N (0.80 mL, 5.75 mmol, 1.1 eq) and Boc_2O (1.36 g, 6.24 mmol, 1.2 eq) were added. The solution was stirred at rt for 2 h. The solution was diluted with DCM and washed with water (3 x 50 mL). The organic layer was washed with brine, dried over Na_2SO_4, filtered, and concentrated under reduced pressure. This gave **27** as a yellow solid (2.25 g, 94%). **^{1}H NMR** (500 MHz, $CDCl_3$) δ 8.43 (dd, *J* = 4.8, 1.7 Hz, 1H), 7.78 – 7.74 (m, 1H), 7.47 (dd, *J* = 7.7, 1.7 Hz, 1H), 7.37 – 7.33 (m, 1H), 7.14 (dd, *J* = 7.7, 4.8 Hz, 1H), 3.83 – 3.74 (m, 2H), 3.50 – 3.34 (m, 2H), 3.10 (dddd, *J* = 13.2, 13.2, 10.4, 3.6 Hz, 2H), 2.94 – 2.82 (m, 2H), 2.45 (m, 2H), 2.30 (dddd, *J* = 14.5, 5.2 Hz, 2H), 1.45 (s, 9H). **^{13}C NMR** (126 MHz, $CDCl_3$) δ 156.3, 154.8, 147.3, 145.6, 144.5, 140.3, 139.0, 137.7, 133.0, 132.5, 132.0, 126.7, 125.8, 122.9, 79.9, 45.9, 31.9, 31.0, 31.0, 30.8, 28.5. **LC-MS:** t_r = 4.54 min, purity 97 %, $[M+H]^+$ m/z calc. 456.16 found 456.20.

tert-butyl 4-(9-amino-8-chloro-5,6-dihydro-11H-benzo[5,6]cyclohepta[1,2-b]pyridin-11-ylidene)piperidine-1-carboxylate (33)

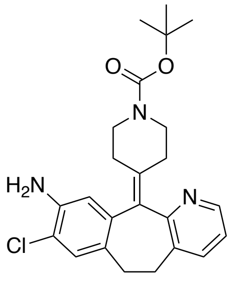

This compound was synthetised according to general procedure **C** using nitro-compound **27** (2.00 g, 4.39 mmol, 1.0 eq), Fe (980 mg, 17.55 mmol, 4.0 eq), NH_4Cl (821 mg, 15.4 mmol, 3.5 eq), 1,4-dioxane (24 mL), EtOH (20 mL), water (20 mL) and a reaction time of 3h. This gave **33** as a white solid (1.67 g, 89%). **^{1}H NMR** (500 MHz, $CDCl_3$) δ 8.38 (dd, *J* = 4.8, 1.7 Hz, 1H), 7.42 (dd, *J* = 7.7, 1.7 Hz, 1H), 7.08 (dd, *J* = 7.7, 4.8 Hz, 1H), 7.05 (s, 1H), 6.61 (s, 1H), 3.91 (s, 2H), 3.77 (s, 2H), 3.34 – 3.23 (m, 2H), 3.05 (dddd, *J* = 13.4, 9.8, 9.7, 4.0 Hz, 2H), 2.81 (ddd, *J* = 16.8, 9.5, 4.7 Hz, 1H), 2.73 – 2.64 (m, 1H), 2.48 – 2.30 (m, 3H), 2.26 (ddd, *J* = 14.2, 4.6 Hz, 1H), 1.45 (s, 9H). **^{13}C NMR** (126 MHz, $CDCl_3$) δ 157.2, 154.9, 146.5, 140.9, 139.0, 137.9, 137.5, 134.3, 133.8, 129.6, 128.6, 122.3, 118.3, 116.4, 79.7, 45.3, 32.1, 30.9, 30.8, 30.7, 28.6. **LC-MS:** t_r = 3.86 min, purity: 95%, $[M+H]^+$ m/z calc. 426.19, found 426.20.

tert-butyl (E)-4-(8-chloro-9-(phenyldiazenyl)-5,6-dihydro-11H-benzo[5,6]cyclohepta[1,2-b]pyridin-11-ylidene)piperidine-1-carboxylate (34)

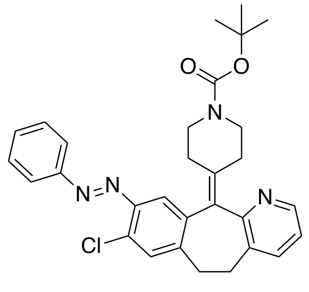

This compound was synthetised according to general procedure **A** using aniline **33** (300 mg, 0.70 mmol, 1.0 eq), PhNO (83 mg, 0.78 mmol, 1.1 eq) and AcOH (0.16 mL, 2.8 mmol, 4.0 eq) and PhMe (7 mL). The mixture was stirred for 14 d at 80 °C, after which approximately 40% consumption of the starting material was achieved. The reaction mixture was made basic using satd. aq. $NaHCO_3$ (40 mL). The resulting solution was extracted with EtOAc (3 x 40 mL). The combined organic layers were washed with brine, dried over Na_2SO_4, filtered, and concentrated under reduced pressure. The crude product was purified using normal-phase column chromatography (10% -> 35% EtOAc in cHex).

This gave **34** as an orange solid (90 mg, 25%).**^{1}H NMR** (500 MHz, $CDCl_3$) δ 8.43 (dd, *J* = 4.9, 1.7 Hz, 1H), 7.95 – 7.89 (m, 2H), 7.63 (m, 1H), 7.56 – 7.43 (m, 4H), 7.38 (m, 1H), 7.12 (dd, *J* = 7.7, 4.8 Hz, 1H), 3.78 (m, 2H), 3.51 – 3.35 (m, 2H), 3.12 (ddd, *J* = 13.0, 9.2, 4.0 Hz, 2H), 2.94 – 2.84 (m, 2H), 2.53 (ddd, *J* = 14.0, 9.3, 4.6 Hz, 1H), 2.44 – 2.28 (m, 3H), 1.45 (s, 9H). **^{13}C NMR** (126 MHz, $CDCl_3$) δ 154.8, 152.7, 146.7, 146.7, 142.2, 138.5, 137.5, 134.1, 133.3, 131.4, 130.9, 129.1, 123.3, 122.4, 118.2, 79.6, 45.2, 31.6, 31.5, 30.9, 30.7, 28.4. The signals for three quaternary carbon atoms are missing. **LC-MS:** t_r = 5.41 min, purity 98%, λ_{max}: 325 nm, $[M+H]^+$ m/z calc. 515.22, found 515.30.

(E)-8-chloro-9-(phenyldiazenyl)-11-(piperidin-4-ylidene)-6,11-dihydro-5H-benzo[5,6]cyclohepta[1,2-b]pyridine (11a)

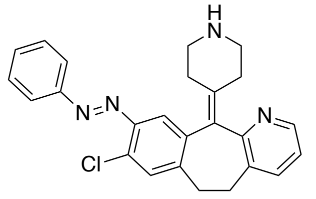

This compound was synthetised according to general procedure **B** using protected amine **34** (65 mg, 0.13 mmol, 1.0 eq), HCl in 1,4-dioxane (0.32 mL, 1.3 mmol, 10.0 eq), MeOH (1.3 mL). The mixture was stirred at rt for 20 h. This gave **11a** as an orange solid (37 mg, 71%). **^{1}H NMR** (600 MHz, $CDCl_3$) δ 8.41 (dd, *J* = 4.8, 1.7 Hz, 1H), 7.94 – 7.89 (m, 2H), 7.64 – 7.61 (m, 1H), 7.54 – 7.46 (m, 3H), 7.44 (dd, *J* = 7.7, 1.7 Hz, 1H), 7.39 – 7.35 (m, 1H), 7.09 (dd, *J* = 7.7, 4.7 Hz, 1H), 3.52 – 3.45 (m, 1H), 3.45 – 3.37 (m, 1H), 3.10 (dddd, *J* = 17.3, 11.8, 4.3, 4.3 Hz, 2H), 2.92 – 2.84 (m, 2H), 2.81 – 2.71 (m, 2H), 2.49 (ddd, *J* = 13.9, 9.6, 4.4 Hz, 1H), 2.43 – 2.34 (m, 3H). **^{13}C NMR** (151 MHz, $CDCl_3$) δ 157.2, 152.8, 147.0, 146.7, 142.5, 139.4, 138.8, 137.6, 134.1, 133.4, 132.6, 131.5, 131.0, 129.2, 123.4, 122.3, 118.4, 48.0, 47.9, 32.4, 32.2, 31.8, 31.6. **LC-MS:** t_r = 3.54 min, purity 98%, λ_{max}: 330 nm, $[M+H]^+$ m/z calc. 415.16, found 415.15. **HRMS**: $[M+H]^+$ calc. for $C_{25}H_{24}ClN_4$: 415.1684, found 415.1685.

8-chloro-7-nitro-11-(piperidin-4-ylidene)-6,11-dihydro-5H-benzo[5,6]cyclohepta[1,2-b]pyridine (29)

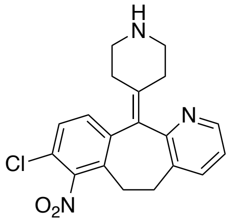

Protected amine **28** (640 mg, 1.50 mmol, 1.0 eq) was dissolved in conc. HCl (2.5 mL, 29.0 mmol, 19 eq). The solution was heated at 80 °C for 22 h. The pH was adjusted to 8 using aq. NH_4OH (28-30 wt%), after which the reaction mixture was diluted with water. The mixture was extracted with DCM (3 x 30 mL). The combined organic layers were washed with brine, dried over Na_2SO_4, filtered, and concentrated under reduced pressure. This yielded **29** as a yellow solid (475 mg, 89%). **^{1}H NMR** (500 MHz, $CDCl_3$) δ 8.42 (dd, *J* = 4.7, 1.7 Hz, 1H), 7.44 (dd, *J* = 7.8, 1.7 Hz, 1H), 7.31 (d, *J* = 8.3 Hz, 1H), 7.27 (d, *J* = 8.5 Hz, 1H), 7.12 (dd, *J* = 7.7, 4.7 Hz, 1H), 3.41 – 3.30 (m, 2H), 3.09 (ddd, *J* = 16.8, 8.5, 4.8 Hz, 2H), 3.00 – 2.87 (m, 1H), 2.75 (dddd, *J* = 12.3, 10.8, 9.2, 4.9 Hz, 3H), 2.49 (ddd, *J* = 14.1, 9.6, 4.5 Hz, 1H), 2.42 – 2.27 (m, 3H). **^{13}C NMR** (126 MHz, $CDCl_3$) δ 155.9, 149.8, 147.2, 141.1, 140.8, 138.0, 132.8, 131.7, 131.6, 131.2, 128.1, 123.4, 122.7, 47.9, 47.9, 32.5, 32.2, 30.7, 27.0. **LC-MS**: t_r = 2.99 min, purity 98%, $[M+H]^+$ m/z calc. 356.10, found 356.11.

tert-butyl 4-(8-chloro-7-nitro-5,6-dihydro-11H-benzo[5,6]cyclohepta[1,2-b]pyridin-11-ylidene)piperidine-1-carboxylate (30)

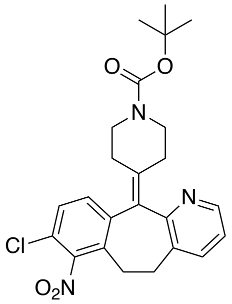

Amine **29** (459 mg, 1.29 mmol, 1.0 eq) was dissolved in DCM (11.5 mL). To this solution were added Et_3N (0.20 mL, 1.4 mmol, 1.1 eq) and Boc_2O (310 mg, 1.42 mmol, 1.1 eq). The solution was stirred at rt for 2 h. The mixture was diluted with DCM and washed with water (3 x 25 mL). The organic layer was washed with brine, dried over Na_2SO_4, filtered, and concentrated under reduced pressure. This yielded **30** as a light brown solid (484 mg, 82%). **^{1}H NMR** (500 MHz, $CDCl_3$) δ 8.42 (dd, *J* = 4.8, 1.7 Hz, 1H), 7.46 (dd, *J* = 7.8, 1.7 Hz, 1H), 7.32 (d, *J* = 8.2 Hz, 1H), 7.27 (d, *J* = 8.0 Hz, 1H), 7.14 (dd, *J* = 7.7, 4.8 Hz, 1H), 3.86 – 3.73 (m, 2H), 3.40 –

3.28 (m, 2H), 3.16 – 3.06 (m, 2H), 2.99 – 2.90 (m, 1H), 2.76 (ddd, *J* = 15.1, 7.6, 4.3 Hz, 1H), 2.51 (ddd, *J* = 14.2, 9.4, 4.7 Hz, 1H), 2.40 – 2.22 (m, 3H), 1.45 (s, 9H). **^{13}C NMR** (126 MHz, $CDCl_3$) δ 155.4, 154.7, 149.7, 146.9, 140.5, 140.2, 138.1, 132.7, 132.4, 131.5, 131.0, 128.1, 123.5, 122.7, 79.8, 45.8, 31.0, 30.6, 28.4, 26.9. **LC-MS**: t_r = 4.69 min, purity: 98%, $[M+H]^+$ m/z calc. 456.16, found 456.15.

tert-butyl 4-(7-amino-8-chloro-5,6-dihydro-11H-benzo[5,6]cyclohepta[1,2-b]pyridin-11-ylidene)piperidine-1-carboxylate (31)

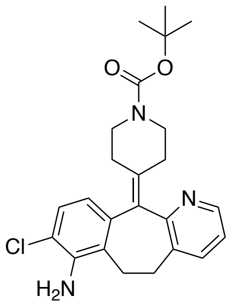

This compound was synthetised according to general procedure **C** using nitro-compound **30** (463 mg, 1.02 mmol, 1.0 eq), Fe (199 mg, 3.57 mmol, 3.5 eq), NH_4Cl (217 mg, 4.08 mol, 4.0 eq), 1,4-dioxane (11 mL), EtOH (9 mL), water (6 mL) and a reaction time of 2h. This yielded **31** as a yellow solid (350 mg, 81%). **^{1}H NMR** (500 MHz, $CDCl_3$) δ 8.42 (dd, *J* = 4.9, 1.7 Hz, 1H), 7.53 (dd, *J* = 7.6, 1.6 Hz, 1H), 7.18 – 7.10 (m, 2H), 6.64 (d, *J* = 8.3 Hz, 1H), 4.06 (s, 2H), 3.76 (s, 2H), 3.46 (ddd, *J* = 15.1, 11.3, 4.3 Hz, 1H), 3.15 (ddd, *J* = 13.1, 8.5, 4.7 Hz, 1H), 3.09 (ddd, *J* = 8.7, 3.2, 3.2 Hz, 2H), 2.89 (ddd, *J* = 14.6, 6.2, 4.5 Hz, 1H), 2.63 (ddd, *J* = 16.0, 11.3, 4.5 Hz, 1H), 2.52 – 2.35 (m, 3H), 2.22 – 2.13 (m, 1H), 1.47 (s, 9H). **^{13}C NMR** (126 MHz, $CDCl_3$) δ 159.4, 154.9, 146.9, 141.1, 137.0, 136.4, 134.6, 133.7, 126.7, 122.5, 122.4, 120.8, 118.8, 79.7, 45.2, 30.8, 30.2, 28.6, 28.4. One signal for a quaternary carbon atom is missing. **LC-MS**: t_r = 3.73 min, purity: 97%, [M+H]+ m/z calc. 426.19, found 426.30.

tert-butyl (E)-4-(8-chloro-7-(phenyldiazenyl)-5,6-dihydro-11H-benzo[5,6]cyclohepta[1,2-b]pyridin-11-ylidene)piperidine-1-carboxylate (32)

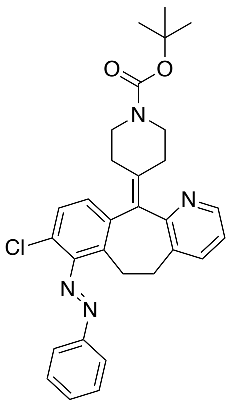

Aniline **31** (200 mg, 0.47 mmol, 1.0 eq) was dissolved in THF (2.4 mL). To this solution was added BF_3•Et_2O (87 µL, 0.70 mmol, 1.5 eq). The mixture was stirred at 0 °C for 15 min. Subsequently, *t*BuONO (67 µL, 0.56 mmol, 1.2 eq) was added and the solution was stirred at rt for 2 h. The solution was cooled to -70 °C and PhMgBr (172 µL, 0.52 mmol, 1.1 eq) was added. The reaction mixture was warmed to rt and stirred for 18 h. The reaction mixture was diluted with water and extracted with EtOAc (3 x 40 mL). The combined organic layers were washed with brine, dried over Na_2SO_4, filtered, and concentrated under reduced pressure. The crude product was purified using reverse-phase column chromatography (5->90% MeCN + 0.1% H_2COH in H_2O + 0.1% H_2COH). The desired fractions were combined, made basic with satd. aq. $NaHCO_3$ and extracted three times with EtOAc. The combined organic layers were washed with brine, dried over Na_2SO_4, filtered, and concentrated under reduced pressure. This yielded **32** as an orange oil (35 mg, 14%). **^{1}H NMR** (600 MHz, $CDCl_3$) δ 8.41 (dd, J = 4.8, 1.6 Hz, 1H), 7.97 – 7.92 (m, 2H), 7.62 – 7.52 (m, 3H), 7.47 – 7.40 (m, 1H), 7.31 (d, J = 8.2 Hz, 1H), 7.16 – 7.07 (m, 2H), 3.92 – 3.66 (m, 2H), 3.30 (ddd, J = 16.2, 6.8, 4.3 Hz, 1H), 3.21 – 3.06 (m, 3H), 2.96 – 2.79 (m, 2H), 2.52 (ddd, J = 14.1, 9.2, 4.6 Hz, 1H), 2.44 – 2.32 (m, 3H), 1.46 (s, 9H). **^{13}C NMR** (151 MHz, $CDCl_3$) δ 156.3, 154.9, 152.5, 150.2, 146.6, 140.6, 138.7, 138.2, 133.9, 133.6, 132.2, 130.9, 129.4, 129.1, 128.2, 123.1, 122.5, 79.8, 45.3, 44.5, 31.5, 31.1, 30.8, 28.6, 26.7. **LC-MS**: t_r = 5.15 min, purity: 95%, $[M+H]^+$ m/z calc. 515.30, found 515.30, λ_{max}: 277 nm.

(E)-8-chloro-7-(phenyldiazenyl)-11-(piperidin-4-ylidene)-6,11-dihydro-5H-benzo[5,6]cyclohepta[1,2-b]pyridine (11c)

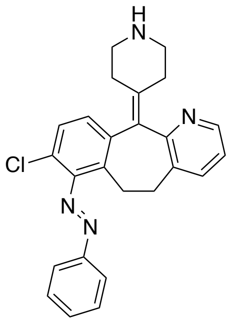

This compound was synthetised according to general procedure **B** using protected amine **32** (35 mg, 0.067 mmol, 1.0 eq), HCl in dioxane (0.17 mL, 0.68 mmol, 10.0 eq), MeOH (0.7 mL) and a reaction time of 22h. The crude product was purified using flash column chromatography (4% -> 6% MeOH + NH_3 in DCM). This yielded **11c** as an orange solid (16 mg, 57%). **^{1}H NMR** (600 MHz, $(CD_3)_2SO$) δ 8.53 – 8.50 (m, 1H), 8.38 – 8.32 (m, 1H), 7.91 (dd, *J* = 6.6, 3.0 Hz, 2H), 7.65 (dd, *J* = 5.2, 1.9 Hz, 3H), 7.54 (dd, *J* = 7.7, 1.7 Hz, 1H), 7.42 (d, *J* = 8.2 Hz, 1H), 7.18 (dd, *J* = 7.7, 4.7 Hz, 1H), 7.11 (d, *J* = 8.2 Hz, 1H), 3.32 – 3.24 (m, 1H), 3.11 (ddd, *J* = 14.9, 10.0, 4.5 Hz, 1H), 2.91 – 2.78 (m, 3H), 2.66 (ddd, *J* = 15.1, 7.5, 4.5 Hz, 1H), 2.58 (m, 2H), 2.31 – 2.09 (m, 4H). **^{13}C NMR** (151 MHz, $(CD_3)_2SO$) δ 156.7, 151.9, 149.5, 146.4, 140.8, 140.1, 137.6, 132.9, 132.6, 131.2, 130.7, 129.7, 129.4, 127.5, 122.8, 122.6, 122.2, 47.9, 47.8, 32.7, 32.4, 30.5, 26.6. **LC-MS**: t_r = 3.49 min, purity: 95%, $[M+H]^+$ m/z calc. 415.17, found 415.15, λ_{max}: 278 nm (not the π-π* band). **HRMS**: $[M+H]^+$ calc. for $C_{25}H_{24}ClN_4$: 415.1684, found 415.1691.

(E)-(4-(phenyldiazenyl)phenyl)methanol (37)

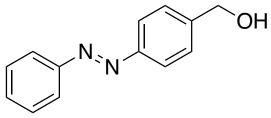

4-Aminobenzylalcohol (**35**, 2.58 g, 20.9 mmol, 1.0 eq) was dissolved in glacial AcOH (100 mL). To this solution was added PhNO (2.55 g, 23.8 mmol, 1.1 eq). The solution was stirred for 66 h at rt. The solution was concentrated under reduced pressure. The crude product was dissolved in EtOAc. The mixture was made basic using satd. aq. $NaHCO_3$ and extracted three times with EtOAc. The combined organic layers were washed with brine, dried over Na_2SO_4, filtered, and concentrated under reduced pressure. The crude product was purified using flash column chromatography (100% DCM) to afford alcohol **37** as an orange solid (530 mg, 12%). **^{1}H NMR** (600 MHz, $CDCl_3$) δ 7.95 – 7.90 (m, 4H), 7.55 – 7.50 (m, 4H), 7.50 – 7.46 (m, 1H), 4.81 – 4.78 (m, 2H), 1.80 (t, *J* = 5.5 Hz, 1H). **^{13}C NMR** (151 MHz, $CDCl_3$) δ 152.75, 152.23, 143.94, 131.16, 129.24, 127.58, 123.23, 122.98, 65.04. **LC-MS:** t_r = 4.42 min, purity >99%, λ_{max}: 325 nm, $[M+H]^+$ m/z calc. 213.10, found 213.00.

(E)-4-(phenyldiazenyl)benzaldehyde (39)

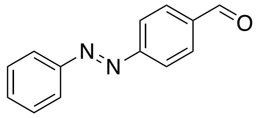

Alcohol **37** (530 mg, 2.50 mmol, 1.0 eq) was dissolved in DCM (45 mL). To this mixture was added DMP (2.457 g, 5.79 mmol, 2.3 eq). The solution was stirred at rt for 1.5 h. The reaction mixture was diluted with DCM, made basic using satd. aq. $NaHCO_3$ and extracted three times with DCM. The combined organic layers were washed with brine, dried over Na_2SO_4, filtered, and concentrated under reduced pressure. The crude product was purified using normal-phase column chromatography (0 -> 10% EtOAc in cHex). This yielded the aldehyde **30** as an orange solid (469 mg, 89%). **^{1}H NMR** (600 MHz, $CDCl_3$) δ 10.11 (s, 1H), 8.06 – 8.03 (m, 4H), 7.99 – 7.95 (m, 2H), 7.58 – 7.51 (m, 3H). **^{13}C NMR** (151 MHz, $CDCl_3$) δ 191.8, 156.0, 152.7, 137.6, 132.1, 130.9, 129.4, 123.5, 123.4. **LC-MS:** t_r = 5.13 min, purity > 99%, λ_{max}: 327 nm, $[M+H]^+$ m/z calc. 211.08, found 210.80.

(*E*)-8-chloro-11-(1-(4-(phenyldiazenyl)benzyl)piperidin-4-ylidene)-6,11-dihydro-5*H*-benzo[5,6]cyclohepta[1,2-*b*]pyridine (12a)

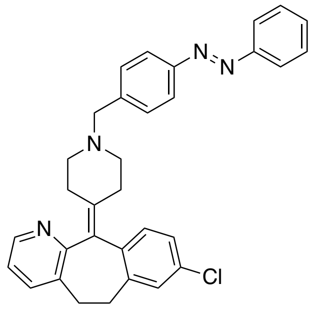

Aldehyde **39** (245 mg, 1.17 mmol, 1.7 eq) and desloratadine (**8**, 210 mg, 0.68 mmol, 1.0 eq) were dissolved in DCE (4.5 mL). The solution was purged with N_2 before the addition of $NaBH(OAc)_3$ (286 mg, 1.35 mmol, 2.0 eq) and a catalytic amount of AcOH (3 drops). The solution was stirred at rt for 16 h. The solution was made basic using satd. aq. $NaHCO_3$ and extracted with DCM thrice. The combined organic layers were washed with brine, dried over Na_2SO_4, filtered, and concentrated under reduced pressure. The crude product was purified using normal-phase column chromatography (0% to 10% MeOH in EtOAc). The title compound **4a** was obtained as an orange solid (87 mg, 26%). **^{1}H NMR** (600 MHz, $CDCl_3$) δ 8.4 (dd, *J* = 4.9, 1.7 Hz, 1H), 7.9 – 7.9 (m, 2H), 7.9 – 7.8 (m, 2H), 7.5 – 7.5 (m, 2H), 7.5 – 7.5 (m, 2H), 7.5 – 7.4 (m, 1H), 7.4 (dd, *J* = 7.7, 1.7 Hz, 1H), 7.2 – 7.1 (m, 2H), 7.1 (dd, *J* = 8.2, 2.0 Hz, 1H), 7.1 (dd, *J* = 7.6, 4.8 Hz, 1H), 3.6 (s, 2H), 3.5 – 3.3 (m, 2H), 2.9 – 2.8 (m, 2H), 2.8 – 2.7 (m, 2H), 2.6 (ddd, *J* = 14.1, 9.9, 4.2 Hz, 1H), 2.5 (ddd, *J* = 13.9, 9.7, 4.2 Hz, 1H), 2.4 – 2.3 (m, 2H), 2.2 – 2.1 (m, 2H). **^{13}C NMR** (151 MHz, $CDCl_3$) δ 157.7, 152.8, 152.0, 146.8, 141.9, 139.6, 139.0, 138.0, 137.4, 133.5, 132.8, 131.0, 131.0, 129.9, 129.2, 129.1, 126.1, 122.9, 122.9, 122.2, 62.6, 55.0, 54.9, 32.0, 31.6, 31.1, 30.9. One missing carbon. **LC-MS:** t_r = 4.00 min, purity 98%, λ_{max}: 318 nm, $[M+H]^+$ m/z calc. 505.21, found 505.20. **HRMS**: $[M+H]^+$ calc. for $C_{32}H_{30}ClN_4$: 505.2154, found 505.2165.

(*E*)-(3-(phenyldiazenyl)phenyl)methanol (38)

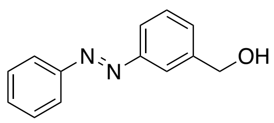

A solution of (3-aminophenyl)methanol (**36**, 3.00 g, 24.4 mmol) and PhNO (2.49 g, 23.2 mmol) in DCM (58 mL) and AcOH (58 mL) was stirred for 18 h at rt. The resulting mixture was concentrated and purified using normal-phase column chromatography (0 -> 10% EtOAc in cHex) to give the title compound **38** (3.420 g, 67%). **^{1}H NMR** (500 MHz, $CDCl_3$) δ 7.95 – 7.89 (m, 3H), 7.86 (ddd, *J* = 7.5, 1.8, 1.8 Hz, 1H), 7.57 – 7.45 (m, 5H), 4.89 – 4.76 (m, 2H), 1.98 (d, *J* = 5.1 Hz, 1H). **^{13}C NMR** (126 MHz, $CDCl_3$) δ 153.0, 152.7, 142.2, 131.2, 129.5, 129.4, 129.2, 123.0, 122.8, 120.7, 65.1. **LC-MS**: t_r = 4.40 min, purity: 97%, $[M+H]^+$ m/z calc. 213.10, found 213.00.

(*E*)-3-(phenyldiazenyl)benzaldehyde (40)

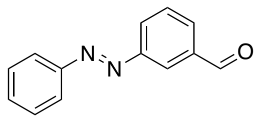

A mixture of alcohol **38** (3.40 g, 16.0 mmol) and MnO_2 (8.36 g, 96 mmol) in DCM (100 mL) was stirred at rt for 2 h. The reaction mixture was filtered over Celite and the filtrate was concentrated *in vacuo* to give the title compound **40** (2.508 g, 75%). **^{1}H NMR** (500 MHz, $CDCl_3$) δ 10.14 (s, 1H), 8.41 (dd, *J* = 1.8, 1.8 Hz, 1H), 8.19 (ddd, *J* = 7.9, 2.1, 1.3 Hz, 1H), 8.01 (ddd, *J* = 7.6, 1.4, 1.4 Hz, 1H), 7.99 – 7.93 (m, 2H), 7.70 (dd, *J* = 7.7, 7.7 Hz, 1H), 7.59 – 7.49 (m, 3H). **^{13}C NMR** (126 MHz, $CDCl_3$) δ 191.9, 153.1, 152.5, 137.5, 131.8, 131.2, 130.0, 129.4, 128.9, 124.0, 123.2. **LC-MS**: t_r = 5.08 min, purity: >99%, $[M+H]^+$ m/z calc. 211.09, found 211.00.

(*E*)-8-chloro-11-(1-(3-(phenyldiazenyl)benzyl)piperidin-4-ylidene)-6,11-dihydro-5*H*-benzo[5,6]cyclohepta[1,2-*b*]pyridine (12b)

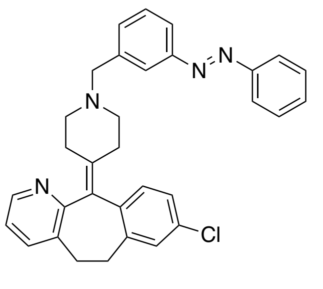

To a solution of aldehyde **40** (304 mg, 1.45 mmol), desloratadine (**8**, 150 mg, 0.48 mmol) and AcOH (221 µL) in DCM (4.8 mL) was added $NaBH(OAc)_3$. The reaction mixture was stirred for 2 h at rt. Satd. aq. $NaHCO_3$ (20 mL) was added. The organic layer was separated, dried over Na_2SO_4 and concentrated. The resulting crude compound was purified using normal-phase column chromatography (0 -> 40% EtOAc in cHex) to give the title compound **12b** as an orange solid (150 mg, 62%). **^{1}H NMR** (600 MHz, $CDCl_3$) δ 8.38 (dd, *J* = 4.8, 1.6 Hz, 1H), 7.93 – 7.89 (m, 2H), 7.87 – 7.84 (m, 1H), 7.82 – 7.77 (m, 1H), 7.54 – 7.49 (m, 2H), 7.49 – 7.44 (m, 3H), 7.42 (dd, *J* = 7.6, 1.7 Hz, 1H), 7.15 – 7.09 (m, 3H), 7.07 (dd, *J* = 7.6, 4.8 Hz, 1H), 3.61 (s, 2H), 3.45 – 3.33 (m, 2H), 2.87 – 2.73 (m, 4H), 2.55 (ddd, *J* = 14.2, 10.0, 4.3 Hz, 1H), 2.45 (ddd, *J* = 14.0, 9.9, 4.3 Hz, 1H), 2.40 – 2.30 (m, 2H), 2.25 – 2.13 (m, 2H). **^{13}C NMR** (151 MHz, $CDCl_3$) δ 157.8, 152.8, 152.8, 146.8, 139.8, 139.6, 139.2, 138.0, 137.3, 133.5, 132.8, 131.8, 131.1, 131.0, 129.2, 129.1, 126.1, 123.6, 123.0, 122.2, 121.8, 62.7, 55.0, 54.9, 32.0, 31.6, 31.2, 30.9. **LC-MS:** t_r = 3.92 min, purity >99%, λ_{max}: 316 nm, $[M+H]^+$ m/z calc. 505.21, found 505.25. **HRMS**: $[M+H]^+$ calc. for $C_{32}H_{30}ClN_4$: 505.2154, found 505.2159.

8-chloro-11-(1-(2-nitrobenzyl)piperidin-4-ylidene)-6,11-dihydro-5*H*-benzo[5,6]cyclohepta[1,2-*b*]pyridine (42)

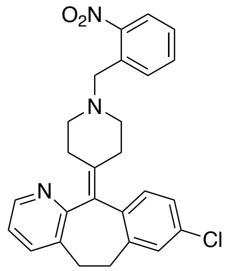

Desloratadine (**8**, 515 mg, 1.66 mmol, 1.0 eq) was dissolved in MeCN (5.5 mL). To this were added 2-nitrobenzyl bromide (391 mg, 1.81 mmol, 1.1 eq) and K_2CO_3 (313 mg, 2.27 mmol, 1.4 eq). The mixture was heated at reflux for 3 h. The mixture was concentrated under reduced pressure and the residue was dissolved in EtOAc. The mixture was washed with satd. aq. $NaHCO_3$ (30 mL) and brine (25 mL). The organic layer was dried over Na_2SO_4, filtered, and concentrated under reduced pressure. The crude product was purified with normal-phase column chromatography (60% -> 100% EtOAc in cHex). Product **42** was obtained as a dark brown solid (669 mg, 91%). ^{1}H NMR (600 MHz, $CDCl_3$) δ 8.38 (dd, *J* = 4.8, 1.7 Hz, 1H), 7.81 (dd, *J* = 8.1, 1.3 Hz, 1H), 7.68 (d, *J* = 7.7 Hz, 1H), 7.53 (ddd, *J* = 7.6, 7.6, 1.3 Hz, 1H), 7.42 (dd, *J* = 7.6, 1.7 Hz, 1H), 7.40 – 7.34 (m, 1H), 7.18 – 7.09 (m, 3H), 7.07 (dd, *J* = 7.6, 4.8 Hz, 1H), 3.78 (s, 2H), 3.44 – 3.30 (m, 2H), 2.88 – 2.74 (m, 3H), 2.73 – 2.66 (m, 2H), 2.49 (ddd, *J* = 14.1, 9.9, 4.3 Hz, 1H), 2.40 (ddd, *J* = 14.0, 9.8, 4.3 Hz, 1H), 2.36 – 2.25 (m, 2H), 2.25 – 2.12 (m, 2H).**^{13}C NMR** (151 MHz, $CDCl_3$) δ 157.7 , 149.9, 146.7, 139.7, 138.7$^\#$, 137.9, 137.4, 134.3$^\#$, 133.6, 132.9, 132.8, 132.6, 131.0, 130.9, 129.1, 127.9, 126.1, 124.5, 122.2, 58.8, 55.0, 54.9, 31.9, 31.6, 31.1, 30.9.**LC-MS**: t_r = 3.31 min, purity: >99%, $[M+H]^+$ m/z calc. 446.16, found 446.15.

2-((4-(8-chloro-5,6-dihydro-11*H*-benzo[5,6]cyclohepta[1,2-*b*]pyridin-11-ylidene)piperidin-1-yl)methyl)aniline (43)

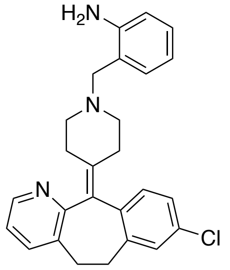

This compound was synthetised according to general procedure **C**, using nitro-compound **42** (649 mg, 1.46 mmol, 1.0 eq), Fe (460 mg, 8.24 mmol, 5.6 eq), NH_4Cl (308 mg, 5.76 mmol, 4.0 eq), 1,4-dioxane (24 mL), EtOH (14 mL), water (8.6 mL), and a reaction time of 3h. This yielded aniline **43** as a brown oil (620 mg, quant.). **^{1}H NMR** (500 MHz, $CDCl_3$) δ 8.37 (dd, *J* = 4.8, 1.7 Hz, 1H), 7.41 (dd, *J* = 7.6, 1.7 Hz, 1H), 7.15 (d, *J* = 1.3 Hz, 1H), 7.11 (d, *J* = 1.3 Hz, 2H), 7.09 – 7.05 (m, 2H), 6.96 – 6.91 (m, 1H), 6.67 – 6.60 (m, 2H), 4.98 (br s, 2H), 3.56 (s, 2H), 3.46 – 3.28 (m, 2H), 2.92 – 2.71 (m, 4H), 2.61 – 2.09 (m, 6H). **^{13}C NMR** (126 MHz, $CDCl_3$) δ 157.4, 147.2, 146.7, 139.6, 137.8, 137.5, 133.5, 132.9, 130.9, 130.7, 129.1, 128.8, 126.1, 122.3, 117.7, 115.9, 61.5,

54.5, 54.4, 31.8, 31.5, 30.7, 30.6. The signals for three quaternary carbon atoms are missing. **UPLC-MS:** t_r = 1.45 min, purity > 99%, $[M+H]^+$ m/z calc. 416.18, found 416.25.

(*E*)-8-chloro-11-(1-(2-(phenyldiazenyl)benzyl)piperidin-4-ylidene)-6,11-dihydro-5*H*-benzo[5,6]cyclohepta[1,2-*b*]pyridine (12c)

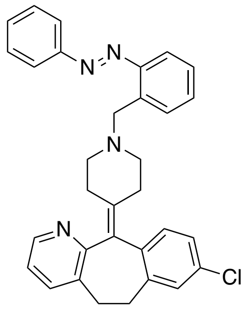

This compound was synthetised according to general procedure **A** with aniline **43** (200 mg, 0.481 mmol, 1.0 eq), PhNO (58 mg, 0.54 mmol, 1.1 eq), AcOH (0.11 mL, 1.9 mmol, 4.0 eq), PhMe (5 mL), and a reaction time of 16h. The crude product was purified using flash column chromatography (19:1 EtOAc:Et_3N). This yielded product **12c** as an orange solid (30 mg, 12%). **^{1}H NMR** (600 MHz, $CDCl_3$) δ 8.37 (dd, *J* = 4.8, 1.6 Hz, 1H), 7.92 – 7.86 (m, 2H), 7.66 – 7.60 (m, 2H), 7.52 (dd, *J* = 8.3, 6.5 Hz, 2H), 7.50 – 7.47 (m, 1H), 7.45 (ddd, *J* = 8.8, 7.6, 2.0 Hz, 1H), 7.41 (dd, *J* = 7.7, 1.7 Hz, 1H), 7.34 (dd^{Δ}, *J* = 7.6, 7.5 Hz, 1H), 7.13 – 7.10 (m, 2H), 7.09 (dd, *J* = 8.1, 2.0 Hz, 1H), 7.06 (dd, *J* = 7.7, 4.7 Hz, 1H), 4.14 (s, 2H), 3.43 – 3.28 (m, 2H), 2.89 – 2.81 (m, 2H), 2.81 – 2.73 (m, 2H), 2.53 (ddd, *J* = 14.2, 10.0, 4.3 Hz, 1H), 2.43 (ddd, *J* = 14.0, 9.8, 4.2 Hz, 1H), 2.37 – 2.29 (m, 2H), 2.29 – 2.18 (m, 2H). **^{13}C NMR** (151 MHz, $CDCl_3$) δ 157.8, 153.0, 151.0, 146.7, 139.6, 139.0, 138.0, 137.4, 137.3, 133.5, 132.7, 132.5, 131.1, 131.0, 131.0, 130.9, 129.2, 129.1, 127.8, 126.1, 123.1, 122.2, 115.4, 56.6, 54.8, 54.7, 32.0, 31.6, 31.2, 31.0. **LC-MS**: t_r = 3.87 min, purity 98%, λ_{max}: 324 nm, $[M+H]^+$ m/z calculated 505.21, found 505.20. **HRMS**: $[M+H]^+$ calc. for $C_{32}H_{30}ClN_4$: 505.2154, found 505.2168.

^{1}H NMR (500 MHz, $CDCl_3$) δ 8.39 (dd, *J* = 4.8, 1.6 Hz, 1H), 7.46 – 7.40 (m, 1H), 7.17 – 7.05 (m, 4H), 3.76 (s, 2H), 3.43 – 3.29 (m, 2H), 3.07 (dddd, *J* = 13.2, 9.6, 4.3, 4.3 Hz, 2H), 2.88 – 2.74 (m, 2H), 2.46 (ddd, *J* = 14.2, 9.4, 4.6 Hz, 1H), 2.39 – 2.23 (m, 3H), 1.44 (s, 9H).

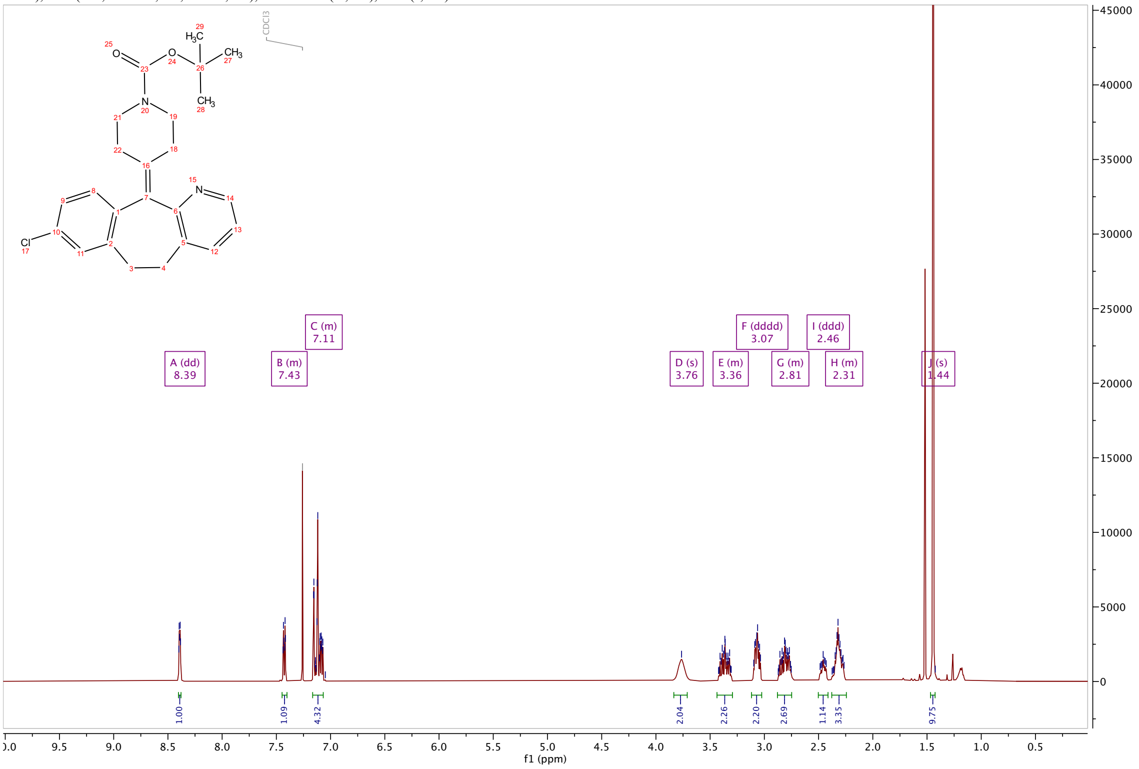

Figure S14. ^{1}H NMR spectrum of **13**.

^{13}C NMR (126 MHz, $CDCl_3$) δ 157.3, 154.9, 146.8, 139.7, 137.9, 137.8, 137.6, 134.1, 133.5, 133.0, 130.7, 129.1, 126.3, 122.4, 79.7, 45.4, 44.6, 31.8, 31.6, 30.9, 30.7, 28.6, 27.5.

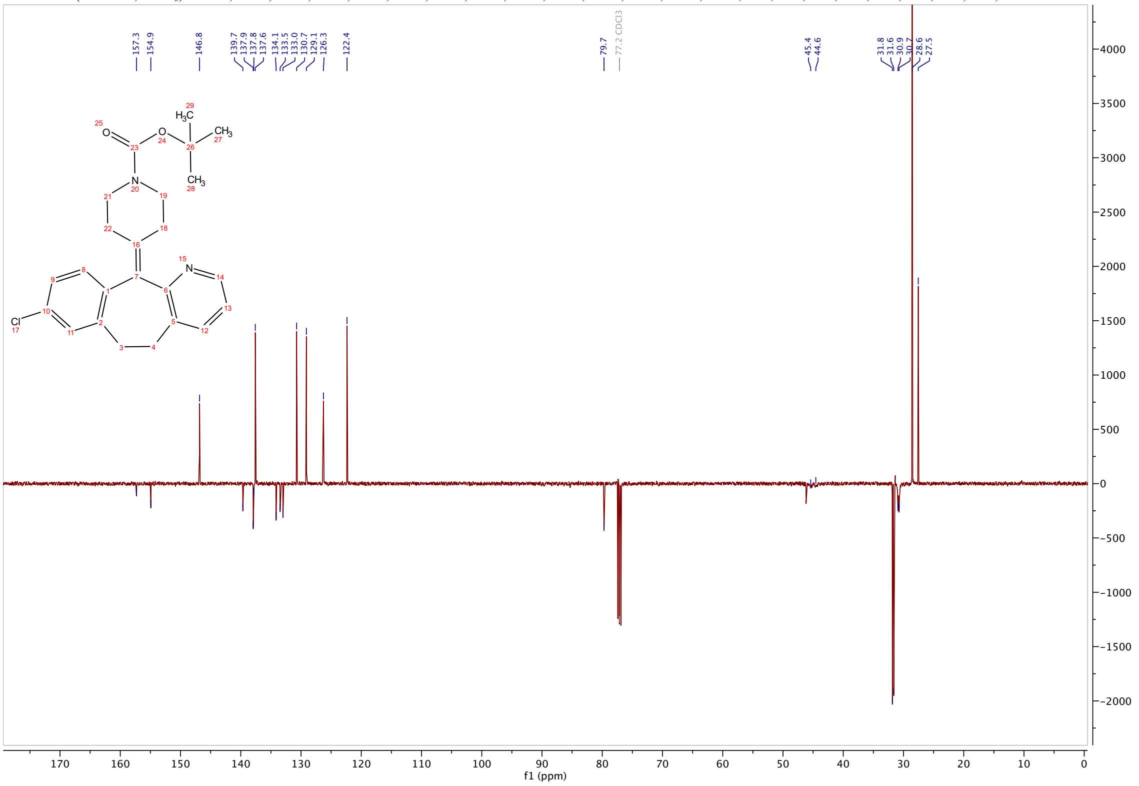

Figure S15. ^{13}C NMR spectrum of **13**.

Acquired by	: Admin
Date Acquired	: 10/4/2024 9:28:01 AM
Sample Name	: DAVE01-086-1
Sample ID	:
Tray#	: 1
Vial#	: 2
Injection Volume	: 1
Data File	: C:\LabSolutions\Data\2024\2024-wk40\DAVE01-086-1.lcd
Background File	: blanco 04102024.lcd
Method File	: Method SCAN ACID standard.lcm
Report Format	: DefaultLCMS.lcr
Tuning File	: C:\LabSolutions\Tuning File\Tuning-ESI-pos-neg01072015.lct
Processed by	: Admin
Modified Date	: 10/4/2024 9:39:13 AM

Chromatogram
DAVE01-086-1 C:\LabSolutions\Data\2024\2024-wk40\DAVE01-086-1.lcd

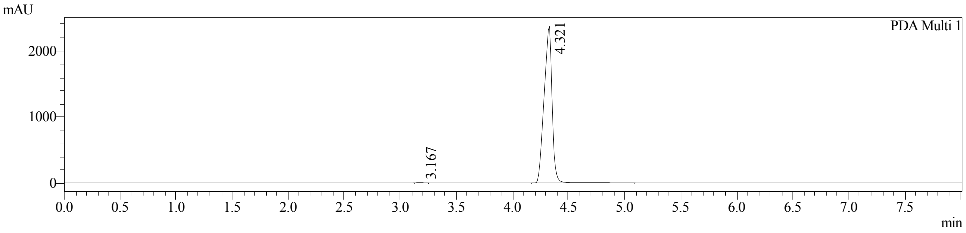

1 PDA Multi 1 / 254nm 4nm

PeakTable

PDA Ch1 254nm 4nm

Peak#	Ret. Time	Area	Height	Name	Area %
1	3.167	4823	1475		0.042
2	4.321	11566513	2370058		99.958
Total		11571336	2371533		100.000

PeakTable

PDA Ch2

MS Chromatogram
DAVE01-086-1 C:\LabSolutions\Data\2024\2024-wk40\DAVE01-086-1.lcd

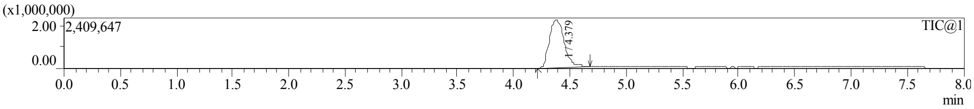

MS Spectrum Graph

#:1 Ret.Time:Averaged 4.370-4.390(Scan#:438-440)
BG Mode:Calc 4.220<->4.680(423<->469)
Mass Peaks:15 Base Peak:411.15(1000979) Polarity:Pos Segment1 - Event1

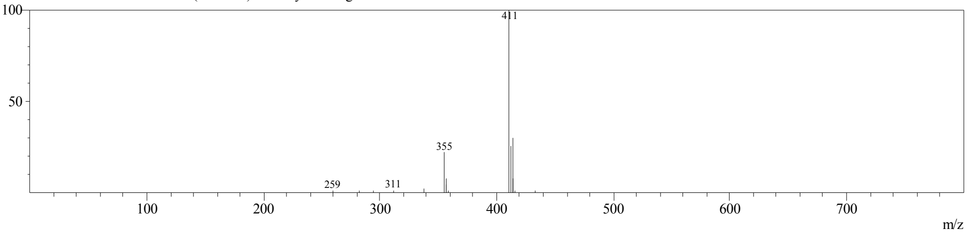

MS Spectrum Table

#:1 Ret.Time:
BG Mode:Calc 4.220<->4.680(423<->469)
Mass Peaks:15 Base Peak:411.15(1000979) Polarity:Pos Segment1 - Event1

#	m/z	Abs.Inten.	Rel.Inten.	Charge	Polarity	Monoisotopic
1	259.10	16379	1.64			
2	282.15	12430	1.24			
3	294.00	14994	1.50			
4	311.15	16839	1.68			
5	337.10	23233	2.32			
6	355.10	222550	22.23			
7	356.10	51643	5.16			
8	357.10	78156	7.81			
9	358.10	17284	1.73			
10	411.15	1000979	100.00			
11	412.15	261610	26.14			
12	413.15	300889	30.06			
13	414.20	78522	7.84			
14	415.25	10799	1.08			
15	433.05	11763	1.18			

Figure S16. LC-MS chromatogram of **13**.

^{1}H NMR (500 MHz, MeOD) δ 8.17 (dd, J = 6.6, 1.2 Hz, 1H), 7.48 (dd, J = 7.8, 1.1 Hz, 1H), 7.35 (dd, J = 7.8, 6.5 Hz, 1H), 7.32 – 7.27 (m, 1H), 7.20 – 7.14 (m, 2H), 3.71 (dddd, J = 17.7, 11.1, 5.1, 5.1 Hz, 2H), 3.48 – 3.22 (m, 4H), 2.99 – 2.86 (m, 2H), 2.48 (ddd, J = 13.4, 8.6, 4.5 Hz, 1H), 2.34 (ddd, J = 14.2, 6.4, 4.0 Hz, 1H), 2.28 – 2.19 (m, 1H), 1.94 (ddd, J = 14.1, 6.3, 3.9 Hz, 1H), 1.46 (s, 9H).

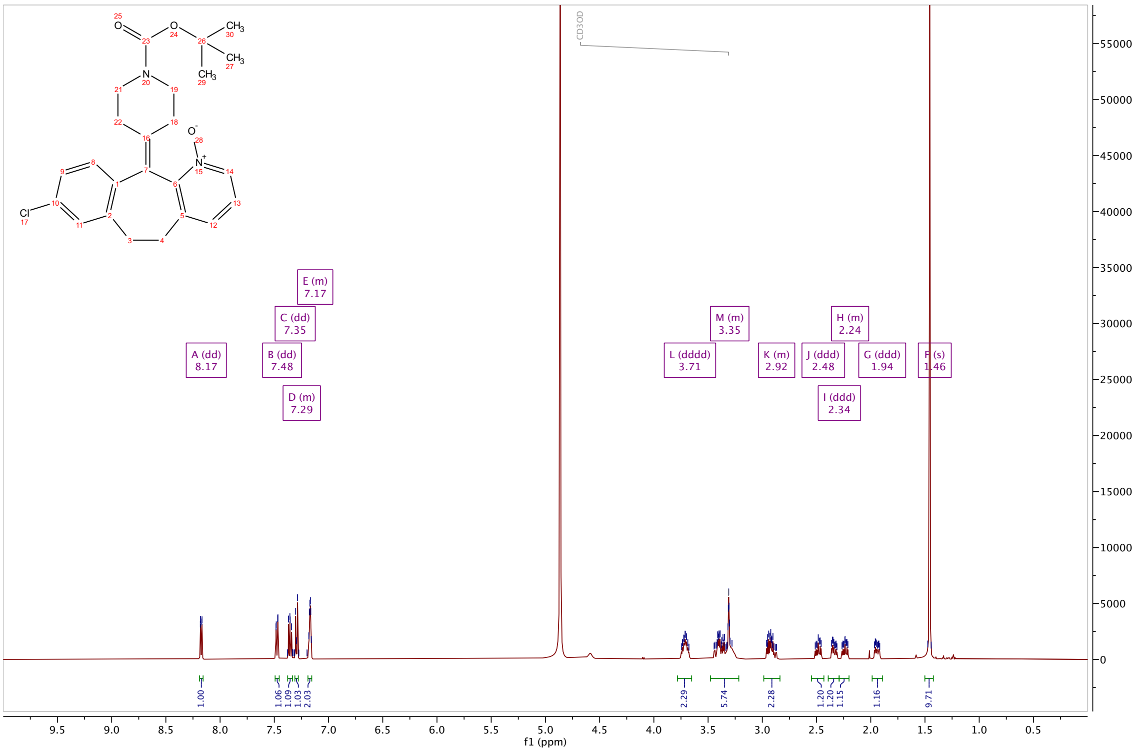

Figure S17. ^{1}H NMR spectrum of **14**.

^{13}C NMR (126 MHz, MeOD) δ 156.4, 150.7, 143.0, 140.4, 140.3, 138.5, 134.9, 134.8, 133.6, 131.3, 130.2, 127.0, 126.1, 124.4, 81.3, 45.9, 44.7, 32.8, 31.3, 31.1, 28.7.

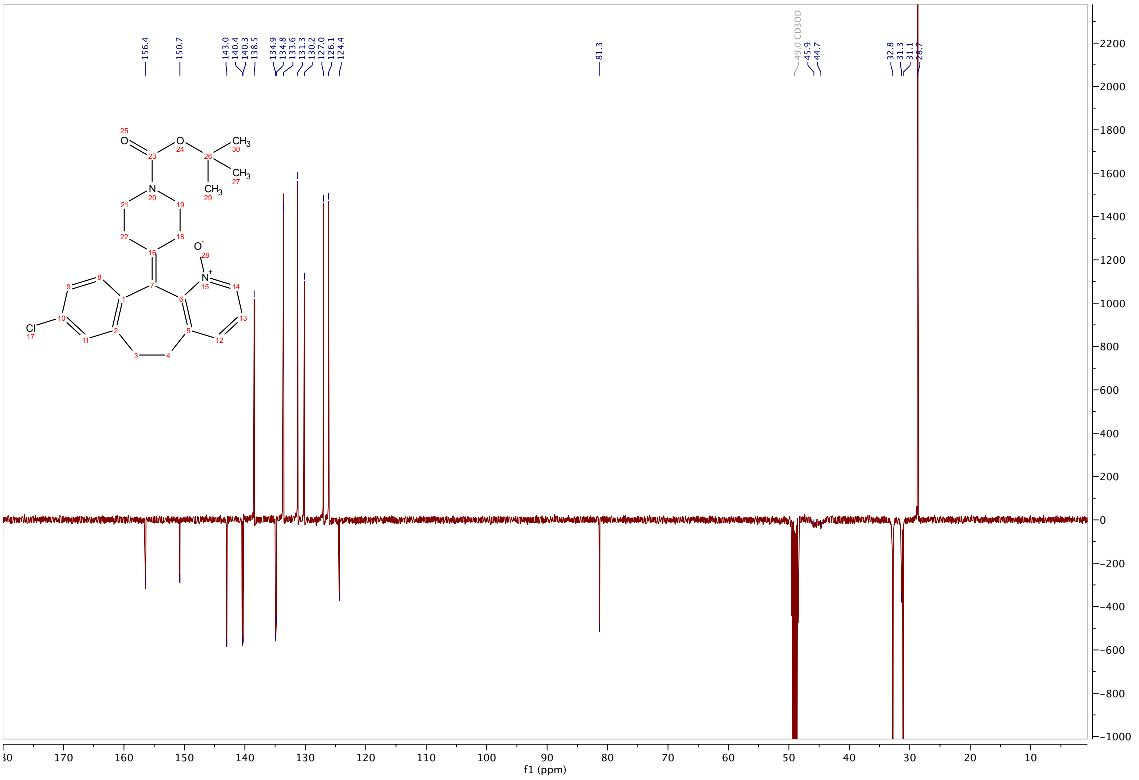

Figure S18. ^{13}C NMR spectrum of **14**.

Acquired by : Admin
Date Acquired : 10/7/2024 3:59:21 PM
Sample Name : DAVE01-087-1
Sample ID :
Tray# : 1
Vial# : 6
Injection Volume : 4
Data File : C:\LabSolutions\Data\2024\2024-wk41\DAVE01-087-2.lcd
Background File : blanco 08102024.lcd
Method File : Method SCAN ACID standard.lcm
Report Format : DefaultLCMS.lcr
Tuning File : C:\LabSolutions\Tuning File\Tuning-ESI-pos-neg01072015.lct
Processed by : Admin
Modified Date : 10/8/2024 2:23:30 PM

Chromatogram
DAVE01-087-1 C:\LabSolutions\Data\2024\2024-wk41\DAVE01-087-2.lcd

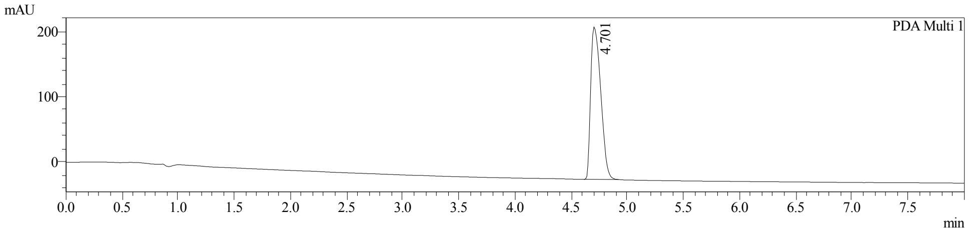

1 PDA Multi 1 / 254nm 4nm

PeakTable

PDA Ch1 254nm 4nm

Peak#	Ret. Time	Area	Height	Name	Area %
1	4.701	1447826	233467		100.000
Total		1447826	233467		100.000

MS Chromatogram
DAVE01-087-1 C:\LabSolutions\Data\2024\2024-wk41\DAVE01-087-2.lcd

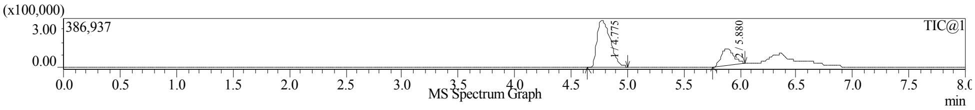

MS Spectrum Graph

#:1 Ret.Time:Averaged 4.760-4.780(Scan#:477-479)
BG Mode:Calc 4.650<->5.000(466<->501)
Mass Peaks:38 Base Peak:427.20(91533) Polarity:Pos Segment1 - Event1

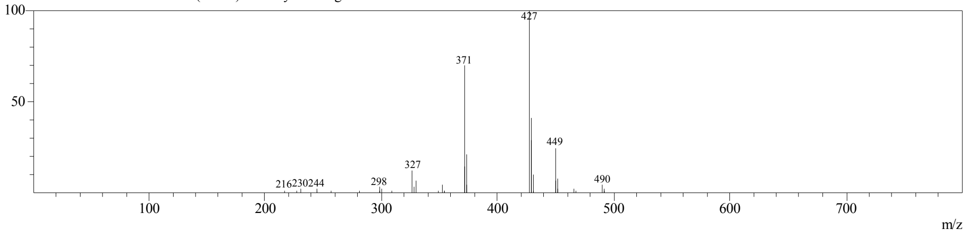

MS Spectrum Table

#:1 Ret.Time:
BG Mode:Calc 4.650<->5.000(466<->501)
Mass Peaks:38 Base Peak:427.20(91533) Polarity:Pos Segment1 - Event1

#	m/z	Abs.Inten.	Rel.Inten.	Charge	Polarity	Monoisotopic	#	m/z	Abs.Inten.	Rel.Inten.	Charge	Polarity	Monoisotopic
1	215.90	1490	1.63				8	297.80	2883	3.15			
2	227.90	1273	1.39				9	298.80	928	1.01			
3	229.90	1964	2.15				10	299.80	1869	2.04			
4	243.90	2048	2.24				11	309.80	1438	1.57			
5	244.90	1507	1.65				12	327.00	11437	12.49			
6	255.85	1283	1.40				13	327.95	2933	3.20			
7	280.85	1125	1.23				14	329.15	5915	6.46			

Figure S19. LC-MS chromatogram of **14**.

^{1}H NMR (500 MHz, $CDCl_3$) δ 7.18 (d, J = 8.2 Hz, 1H), 7.16 – 7.14 (m, 1H), 7.14 – 7.08 (m, 2H), 6.34 (d, J = 8.3 Hz, 1H), 4.38 (s, 2H), 3.94 – 3.70 (m, 2H), 3.31 (ddd, J = 12.9, 9.0, 4.4 Hz, 1H), 3.23 – 3.13 (m, 1H), 3.04 – 2.92 (m, 2H), 2.78 – 2.64 (m, 2H), 2.50 – 2.39 (m, 2H), 2.32 – 2.26 (m, 2H), 1.45 (s, 9H).

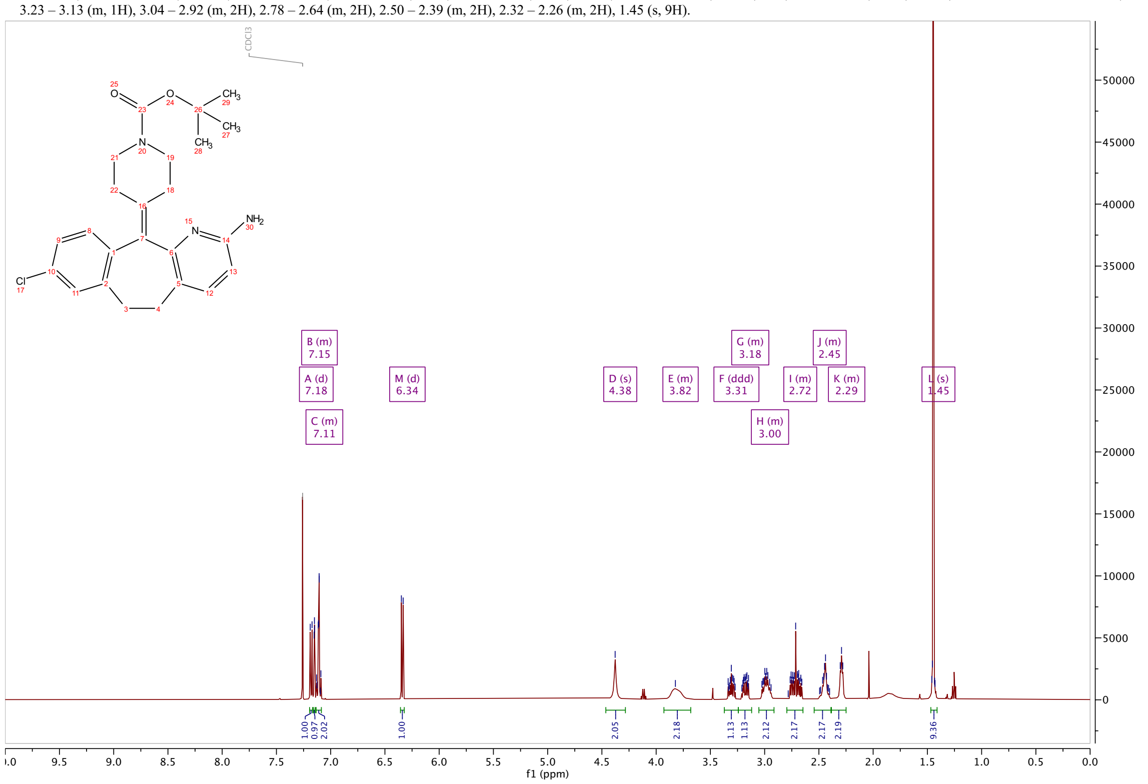

Figure S20. ^{1}H NMR spectrum of **15**.

^{13}C NMR (126 MHz, $CDCl_3$) δ 155.9, 155.0, 154.5, 140.2, 140.0, 138.2, 137.6, 134.1, 132.8, 130.4, 129.0, 126.1, 122.9, 107.7, 79.7, 45.5, 44.4, 32.1, 31.0, 30.8, 28.6.

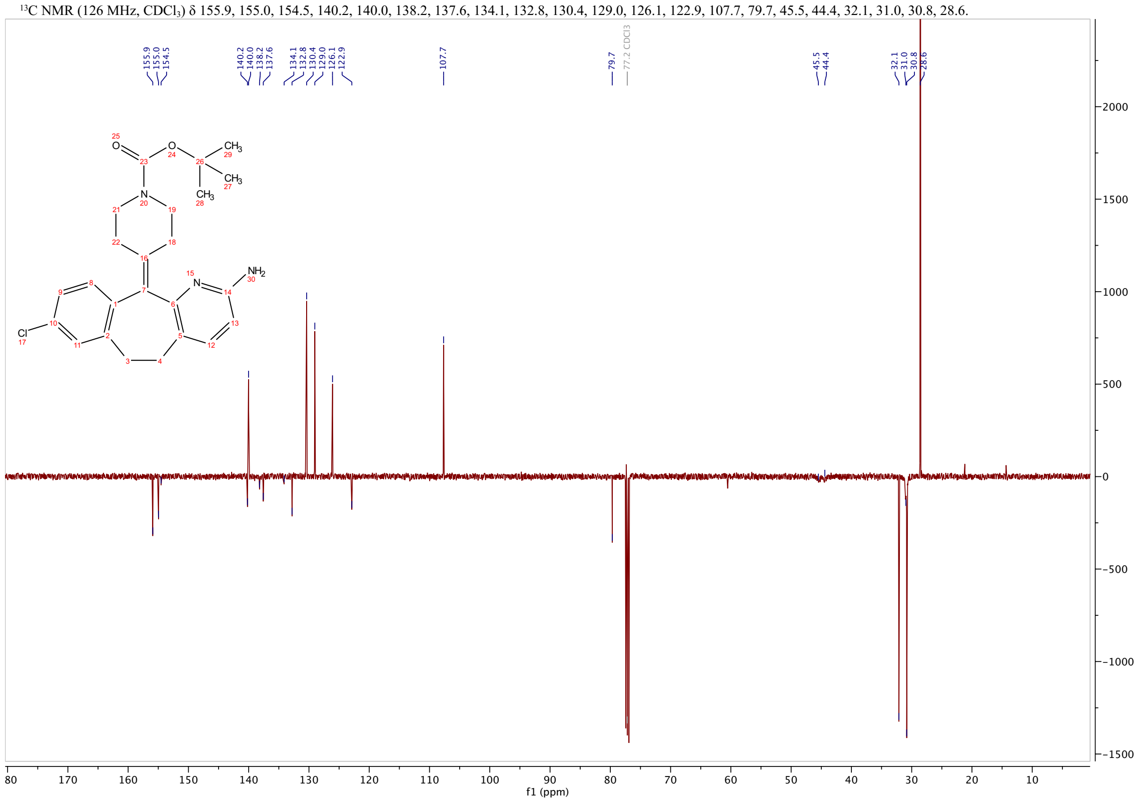

Figure S21. ^{13}C NMR spectrum of **15**.

Acquired by	: Admin
Date Acquired	: 10/9/2024 2:06:09 PM
Sample Name	: DAVE01-088-3
Sample ID	:
Tray#	: 1
Vial#	: 6
Injection Volume	: 1
Data File	: C:\LabSolutions\Data\2024\2024-wk41\DAVE01-088-3.lcd
Background File	: blanco 09102024.lcd
Method File	: Method SCAN ACID standard.lcm
Report Format	: DefaultLCMS.lcr
Tuning File	: C:\LabSolutions\Tuning File\Tuning-ESI-pos-neg01072015.lct
Processed by	: Admin
Modified Date	: 10/9/2024 2:18:55 PM

Chromatogram
DAVE01-088-3 C:\LabSolutions\Data\2024\2024-wk41\DAVE01-088-3.lcd

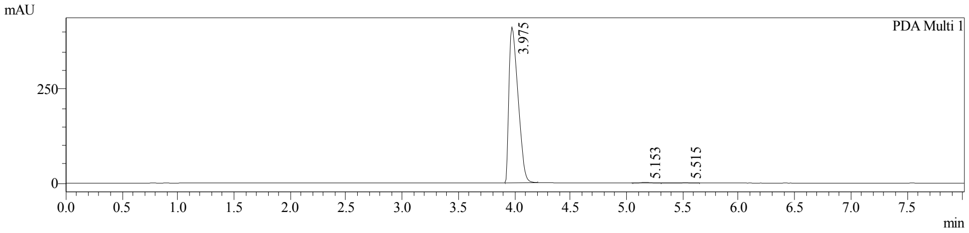

1 PDA Multi 1 / 254nm 4nm

PeakTable

PDA Ch1 254nm 4nm

Peak#	Ret. Time	Area	Height	Name	Area %
1	3.975	2316907	415267		99.367
2	5.153	10667	1847		0.457
3	5.515	4089	700		0.175
Total		2331663	417813		100.000

MS Chromatogram
DAVE01-088-3 C:\LabSolutions\Data\2024\2024-wk41\DAVE01-088-3.lcd

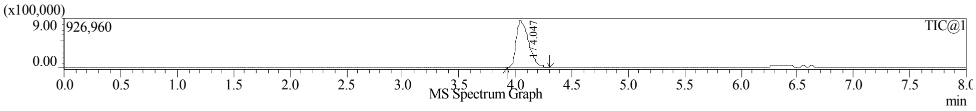

MS Spectrum Graph

#:1 Ret.Time:Averaged 4.040-4.060(Scan#:405-407)
BG Mode:Calc 3.920<->4.310(393<->432)
Mass Peaks:12 Base Peak:426.20(412249) Polarity:Pos Segment1 - Event1

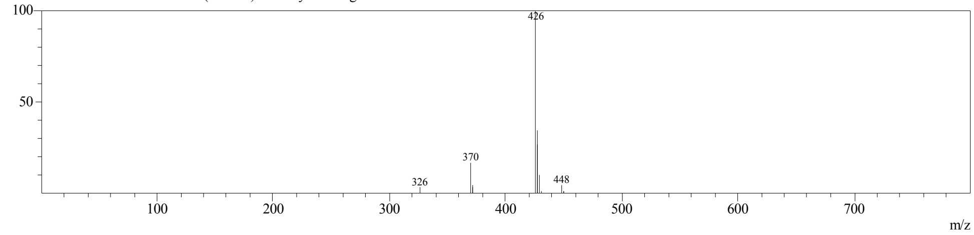

MS Spectrum Table

#:1 Ret.Time:
BG Mode:Calc 3.920<->4.310(393<->432)
Mass Peaks:12 Base Peak:426.20(412249) Polarity:Pos Segment1 - Event1

#	m/z	Abs.Inten.	Rel.Inten.	Charge	Polarity	Monoisotopic	#	m/z	Abs.Inten.	Rel.Inten.	Charge	Polarity	Monoisotopic
1	326.10	12651	3.07				7	428.20	143728	34.86			
2	370.10	69693	16.91				8	429.20	39293	9.53			
3	371.10	13556	3.29				9	430.20	5647	1.37			
4	372.10	21000	5.09				10	448.15	17904	4.34			
5	426.20	412249	100.00				11	449.20	4141	1.00			
6	427.20	110228	26.74				12	450.20	5643	1.37			

Figure S22. LC-MS chromatogram of **15**.

^{1}H NMR (600 MHz, $CDCl_3$) δ 8.06 – 8.00 (m, 2H), 7.61 (d, *J* = 8.2 Hz, 1H), 7.58 (d, *J* = 8.1 Hz, 1H), 7.55 – 7.49 (m, 3H), 7.23 – 7.18 (m, 2H), 7.16 (dd, *J* = 8.1, 2.2 Hz, 1H), 3.98 – 3.68 (m, 2H), 3.49 – 3.39 (m, 2H), 3.06 (ddd, *J* = 13.3, 9.9, 3.7 Hz, 2H), 2.96 (ddd, *J* = 16.2, 10.2, 4.5 Hz, 1H), 2.84 (ddd, *J* = 16.1, 8.0, 4.5 Hz, 1H), 2.64 – 2.57 (m, 1H), 2.49 – 2.43 (m, 1H), 2.40 – 2.31 (m, 2H), 1.45 (s, 9H).

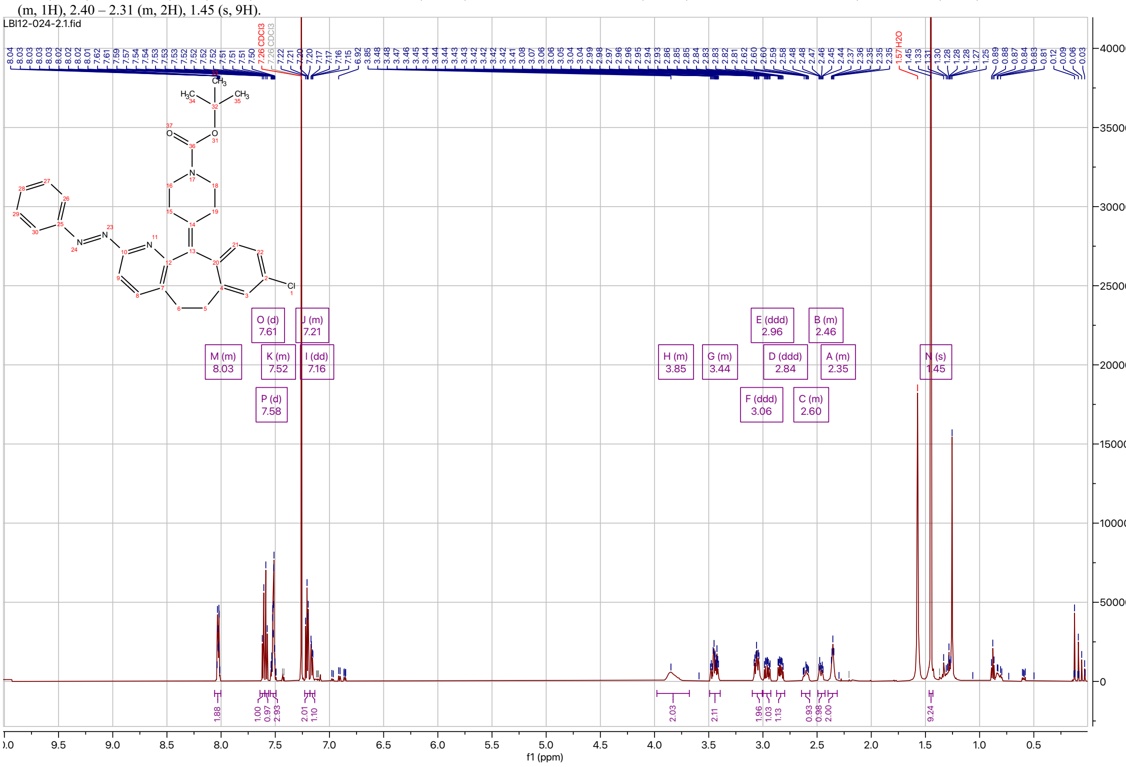

Figure S23. ^{1}H NMR spectrum of **16**.

^{13}C NMR (151 MHz, $CDCl_3$) δ 161.4, 156.7, 155.0, 152.4, 140.0, 139.7, 139.0, 137.9, 135.8, 133.3, 133.1, 132.1, 131.0, 129.2, 129.0, 126.4, 123.8, 110.5, 79.7, 31.8, 31.7, 29.8, 28.6.

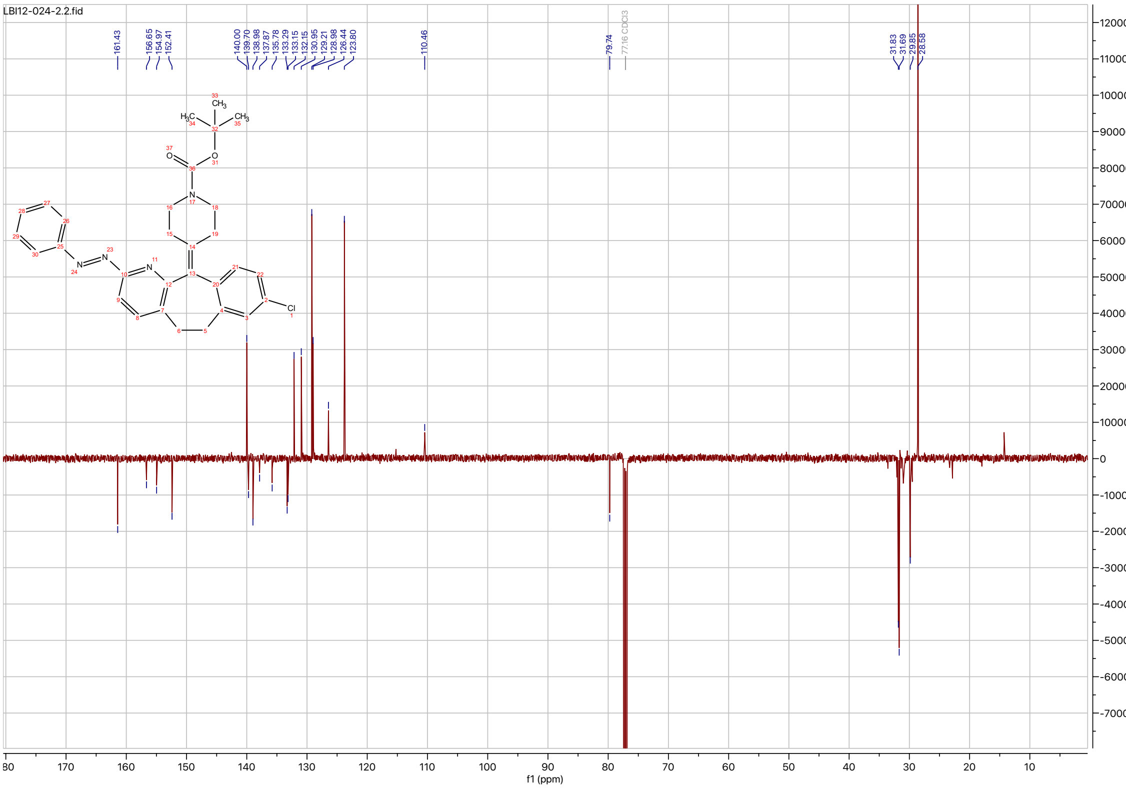

Figure S24. ^{13}C NMR spectrum of **16**.

Acquired by	: Admin
Date Acquired	: 4/23/2025 2:23:16 PM
Sample Name	: LBI12-024-2
Sample ID	:
Tray#	: 1
Vial#	: 7
Injection Volume	: 5
Data File	: C:\LabSolutions\Data\2025\2025-wk17\LBI12-024-2.lcd
Background File	: azoblanco 23042025.lcd
Method File	: Method SCAN ACID standard azo.lcm
Report Format	: DefaultLCMS.lcr
Tuning File	: C:\LabSolutions\Tuning File\Tuning-ESI-pos-neg01072015.lct
Processed by	: Admin
Modified Date	: 4/23/2025 2:36:44 PM

Chromatogram
LBI12-024-2 C:\LabSolutions\Data\2025\2025-wk17\LBI12-024-2.lcd

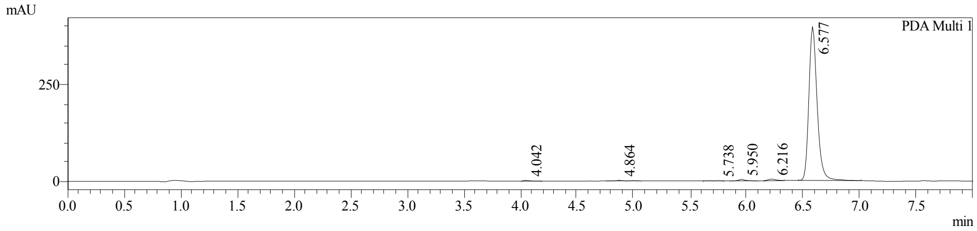

1 PDA Multi 1 / 254nm 4nm

PeakTable

PDA Ch1 254nm 4nm

Peak#	Ret. Time	Area	Height	Name	Area %
1	4.042	5431	1743		0.265
2	4.864	6445	1379		0.314
3	5.738	1821	301		0.089
4	5.950	14203	3653		0.692
5	6.216	19655	4166		0.958
6	6.577	2004255	397092		97.682
Total		2051811	408334		100.000

MS Chromatogram
LBI12-024-2 C:\LabSolutions\Data\2025\2025-wk17\LBI12-024-2.lcd

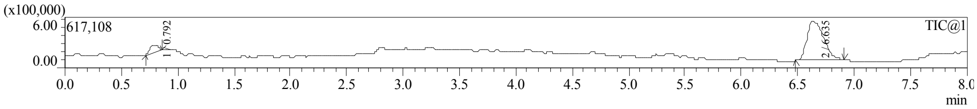

MS Spectrum Graph

#:1 Ret.Time:Averaged 6.620-6.640(Scan#:663-665)
BG Mode:Calc 6.490<->6.920(650<->693)
Mass Peaks:18 Base Peak:515.30(191944) Polarity:Pos Segment1 - Event1

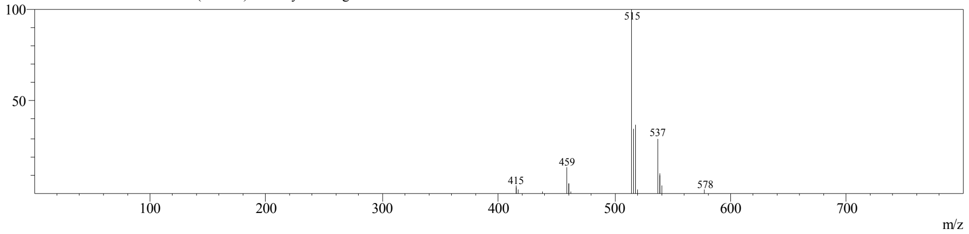

Figure S25. LC-MS chromatogram of **16**.

^{1}H NMR (600 MHz, DMSO) δ 7.94 – 7.90 (m, 2H), 7.82 (d, *J* = 8.1 Hz, 1H), 7.64 – 7.59 (m, 3H), 7.55 (d, *J* = 8.0 Hz, 1H), 7.32 (d, *J* = 2.3 Hz, 1H), 7.24 (dd, *J* = 8.2, 2.3 Hz, 1H), 7.15 (d, *J* = 8.2 Hz, 1H), 3.41 – 3.33 (m, 3H), 3.03 – 2.80 (m, 4H), 2.63 – 2.53 (m, 2H), 2.37 – 2.28 (m, 1H), 2.28 – 2.14 (m, 3H).

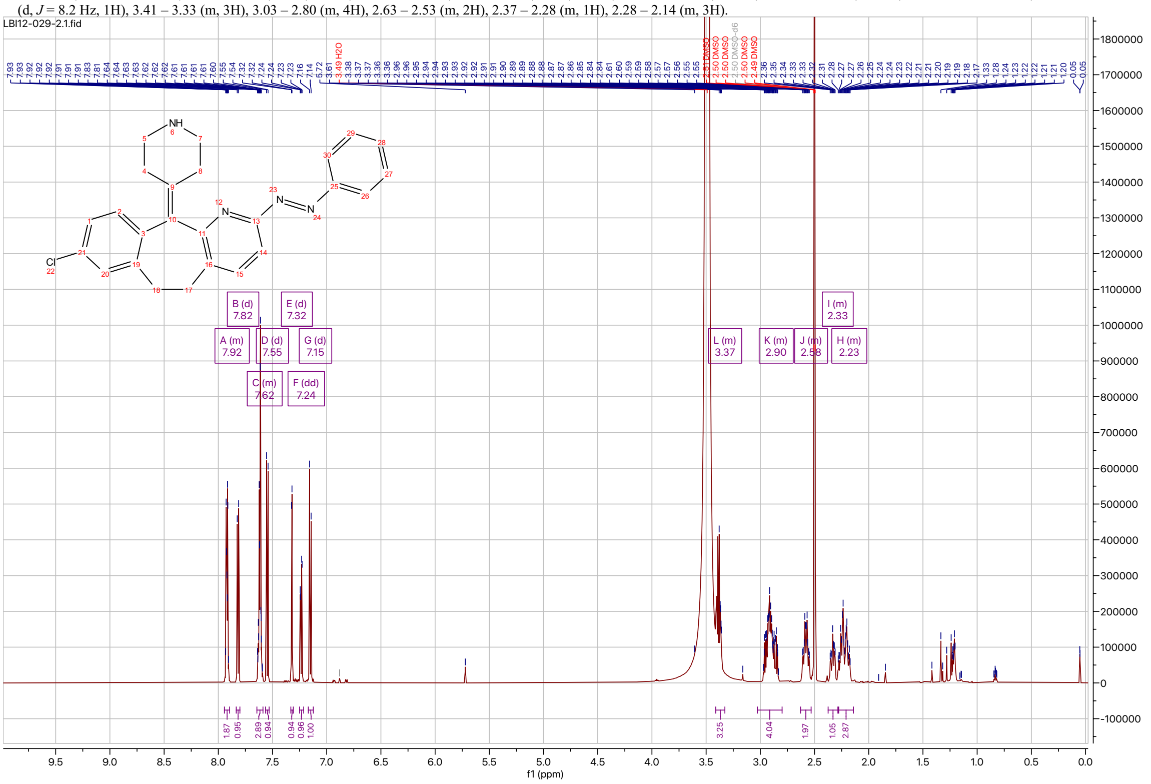

Figure S26. ^{1}H NMR spectrum of **10a**.

^{13}C NMR (151 MHz, DMSO) δ 160.6, 156.9, 152.0, 140.3, 140.3, 139.8, 137.9, 136.3, 132.5, 131.8, 131.1, 131.0, 129.8, 129.1, 125.9, 123.1, 111.4, 47.5, 47.4, 32.1, 32.0, 31.0, 30.7.

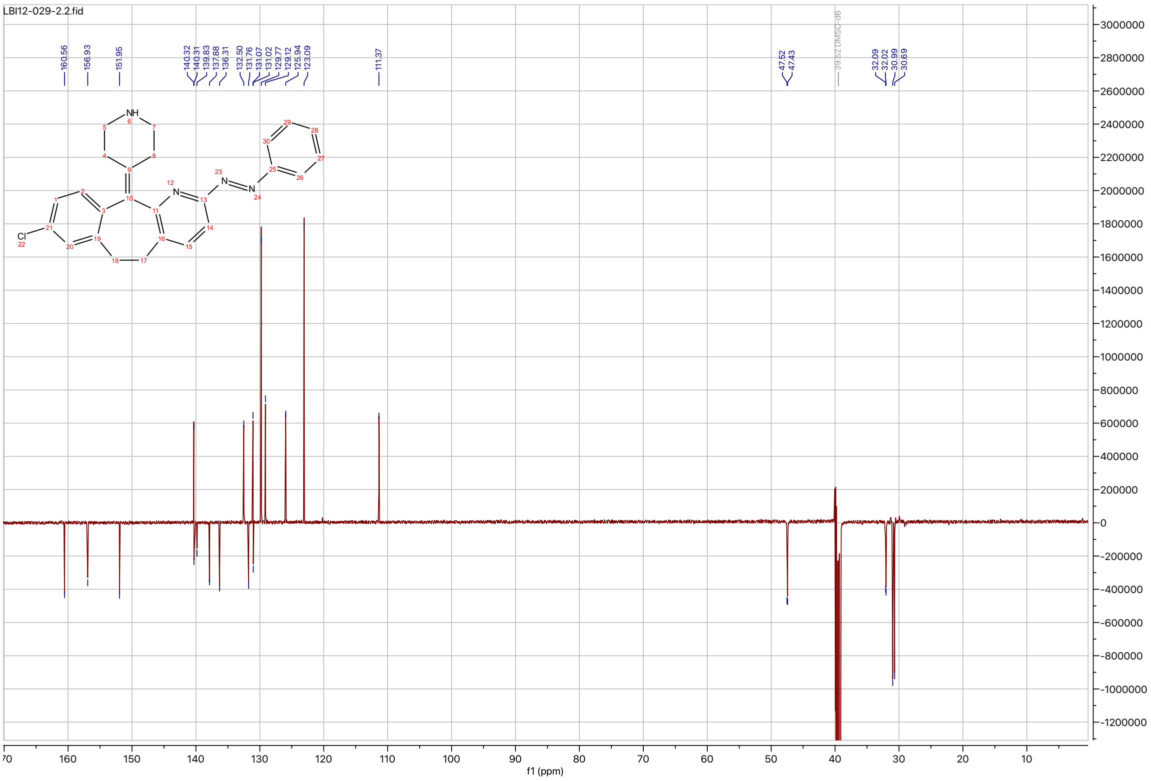

Figure S27. ^{13}C NMR spectrum of **10a**.

Acquired by	: Admin
Date Acquired	: 4/30/2025 2:40:10 PM
Sample Name	: LBI12-029-2
Sample ID	:
Tray#	: 1
Vial#	: 21
Injection Volume	: 10
Data File	: C:\LabSolutions\Data\2025\2025-wk18\LBI12-029-2.lcd
Background File	: azoblanco 30042025.lcd
Method File	: Method SCAN ACID standard azo.lcm
Report Format	: DefaultLCMS.lcr
Tuning File	: C:\LabSolutions\Tuning File\Tuning-ESI-pos-neg01072015.lct
Processed by	: Admin
Modified Date	: 5/1/2025 1:30:55 PM

Chromatogram
LBI12-029-2 C:\LabSolutions\Data\2025\2025-wk18\LBI12-029-2.lcd

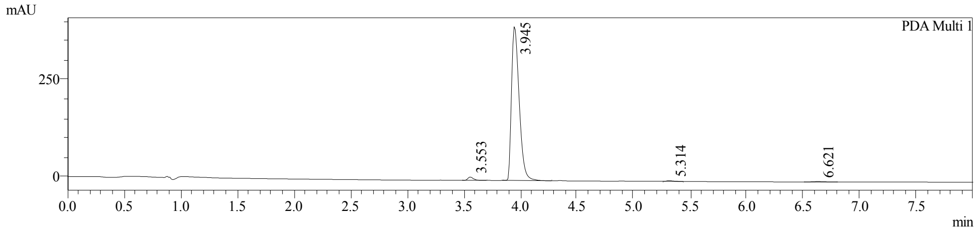

1 PDA Multi 1 / 254nm 4nm

PeakTable

PDA Ch1 254nm 4nm

Peak#	Ret. Time	Area	Height	Name	Area %
1	3.553	27846	8489		1.464
2	3.945	1860721	396315		97.793
3	5.314	7985	1804		0.420
4	6.621	6156	1059		0.324
Total		1902708	407667		100.000

MS Chromatogram
LBI12-029-2 C:\LabSolutions\Data\2025\2025-wk18\LBI12-029-2.lcd

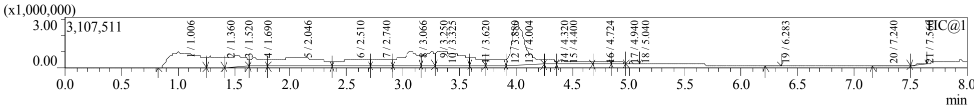

MS Spectrum Graph

#:1 Ret.Time:Averaged 3.990-4.010(Scan#:400-402)
BG Mode:Calc 3.910<->4.250(392<->426)
Mass Peaks:7 Base Peak:415.15(1364982) Polarity:Pos Segment1 - Event1

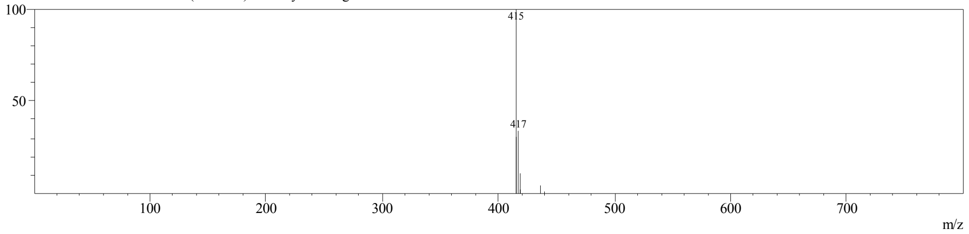

MS Spectrum Table

#:1 Ret.Time:
BG Mode:Calc 3.910<->4.250(392<->426)
Mass Peaks:7 Base Peak:415.15(1364982) Polarity:Pos Segment1 - Event1

#	m/z	Abs.Inten.	Rel.Inten.	Charge	Polarity	Monoisotopic
1	415.15	1364982	100.00			
2	416.15	410985	30.11			
3	417.15	471477	34.54			
4	418.20	140972	10.33			

#	m/z	Abs.Inten.	Rel.Inten.	Charge	Polarity	Monoisotopic
5	419.20	30716	2.25			
6	437.15	60645	4.44			
7	439.30	17363	1.27			

Figure S28. LC-MS chromatogram of **10a**.

Generic Display Report

Analysis Info

Analysis Name	D:\Data\ServiceMS\Hans\2025-wk20\LBI12-029_5-13-2025_09-16-37_100-1200mzrange1.d	Acquisition Date	5/13/2025 9:17:20 AM
Method	100-1200mz range1.m	Operator	Demo User
Sample Name	LBI12-029	Instrument	impact II
Comment			

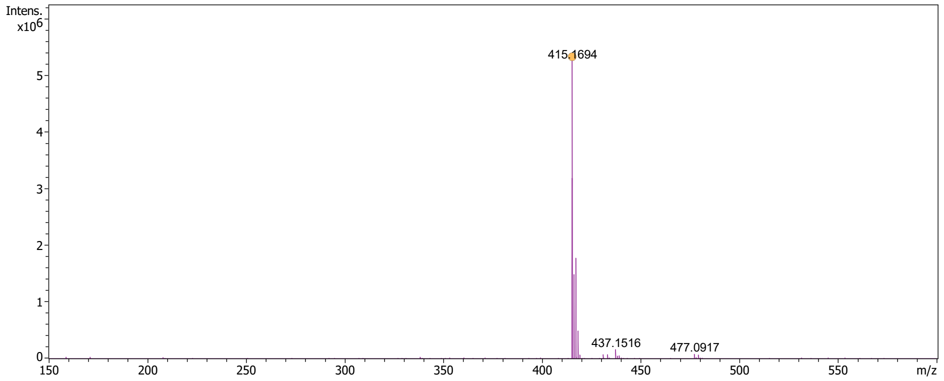

Meas. m/z	#	Ion Formula	m/z	err [ppm]	mSigma	#mSigma	Score	rdb	e⁻ Conf	N-Rule
415.1694	1	C25H24ClN4	415.1684	-2.5	10.0	1	100.00	19.0	even	ok

Bruker Compass DataAnalysis 6.0 printed: 5/13/2025 9:44:14 AM by: demo Page 1 of 1

Figure S29. HRMS spectrum of **10a**.

^{1}H NMR (500 MHz, CDCl$_3$) δ 8.03 (d, J = 5.5 Hz, 1H), 7.21 (d, J = 2.0 Hz, 1H), 7.17 – 7.09 (m, 2H), 6.45 (d, J = 5.6 Hz, 1H), 4.28 (s, 2H), 3.92 – 3.65 (m, 2H), 3.44 (ddd, J = 14.1, 12.2, 4.2 Hz, 1H), 3.04 (dddd, J = 29.0, 13.1, 9.5, 3.8 Hz, 2H), 2.91 (ddd, J = 16.1, 4.7, 4.7 Hz, 1H), 2.80 (ddd, J = 14.1, 4.8, 4.8 Hz, 1H), 2.58 – 2.45 (m, 2H), 2.40 (ddd, J = 14.3, 4.5, 4.5 Hz, 1H), 2.32 – 2.16 (m, 2H), 1.44 (s, 9H).

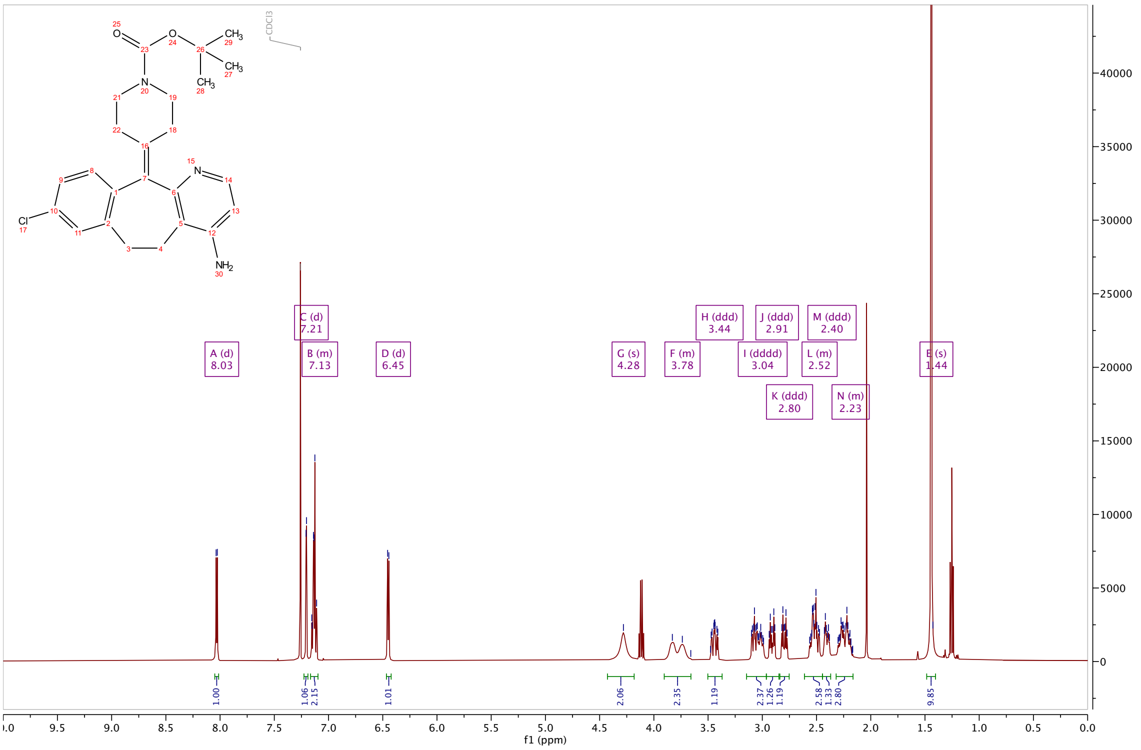

Figure S30. ^{1}H NMR spectrum of **17**.

^{13}C NMR (126 MHz, CDCl$_3$) δ 155.0, 152.2, 145.7, 140.0, 139.6, 138.4, 132.9, 129.7, 128.1, 126.6, 116.5, 108.7, 80.1, 44.2, 31.0, 30.9, 30.7, 28.6, 26.9.

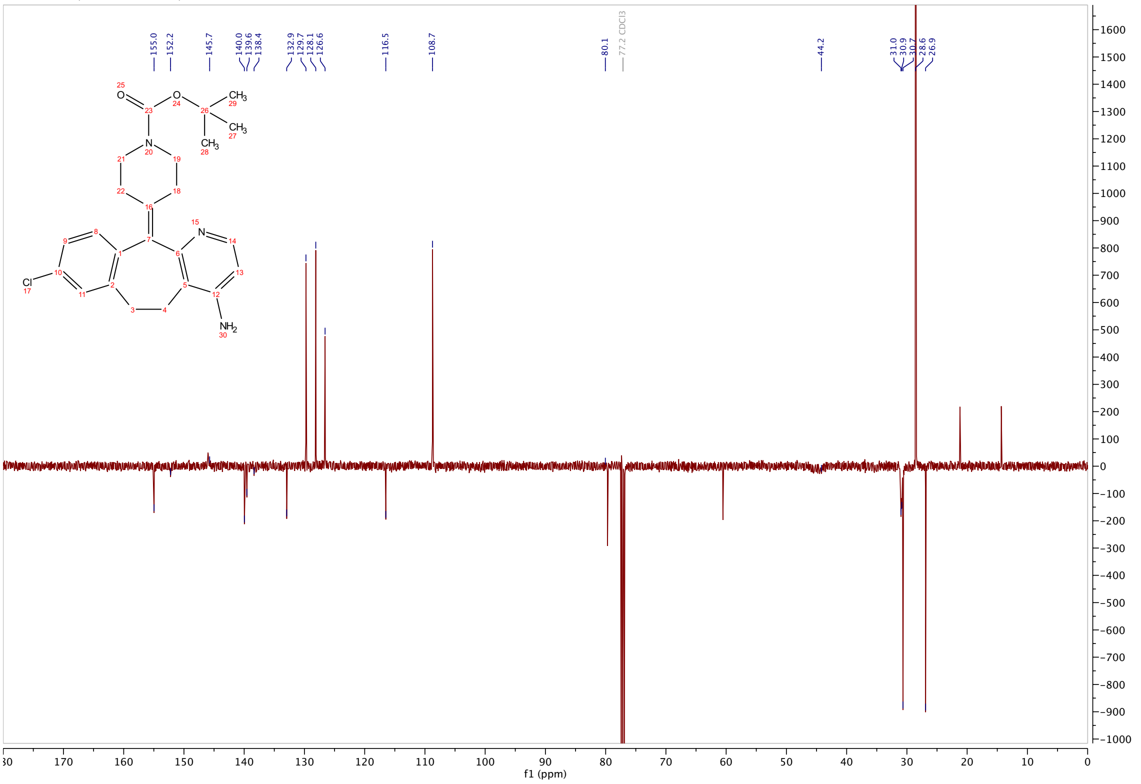

Figure S31. ^{13}C NMR spectrum of **17**.

Acquired by	: Admin
Date Acquired	: 10/15/2024 10:40:28 AM
Sample Name	: DAVE01-089-3
Sample ID	:
Tray#	: 1
Vial#	: 6
Injection Volume	: 2
Data File	: C:\LabSolutions\Data\2024\2024-wk42\DAVE01-089-3.lcd
Background File	: blanco 15102024.lcd
Method File	: Method SCAN ACID standard.lcm
Report Format	: DefaultLCMS.lcr
Tuning File	: C:\LabSolutions\Tuning File\Tuning-ESI-pos-neg01072015.lct
Processed by	: Admin
Modified Date	: 10/15/2024 10:57:10 AM

Chromatogram
DAVE01-089-3 C:\LabSolutions\Data\2024\2024-wk42\DAVE01-089-3.lcd

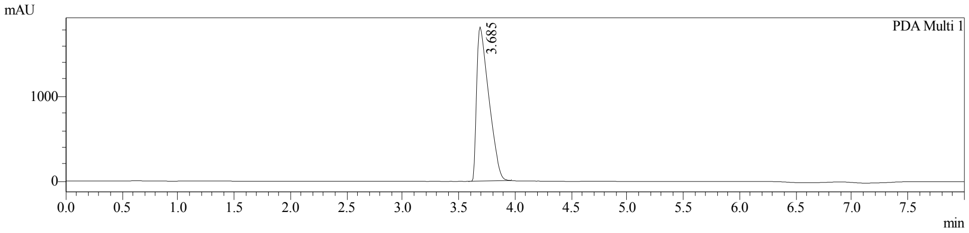

1 PDA Multi 1 / 254nm 4nm

PeakTable

PDA Ch1 254nm 4nm

Peak#	Ret. Time	Area	Height	Name	Area %
1	3.685	13894825	1827905		100.000
Total		13894825	1827905		100.000

MS Chromatogram
DAVE01-089-3 C:\LabSolutions\Data\2024\2024-wk42\DAVE01-089-3.lcd

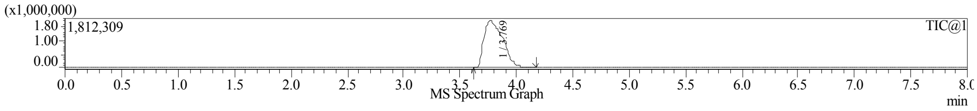

MS Spectrum Graph

#:1 Ret.Time:Averaged 3.760-3.780(Scan#:377-379)
BG Mode:Calc 3.620<->4.170(363<->418)
Mass Peaks:14 Base Peak:426.15(774618) Polarity:Pos Segment1 - Event1

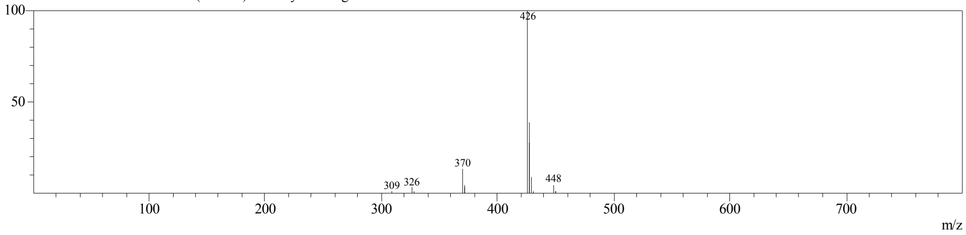

MS Spectrum Table

#:1 Ret.Time:
BG Mode:Calc 3.620<->4.170(363<->418)
Mass Peaks:14 Base Peak:426.15(774618) Polarity:Pos Segment1 - Event1

#	m/z	Abs.Inten.	Rel.Inten.	Charge	Polarity	Monoisotopic	#	m/z	Abs.Inten.	Rel.Inten.	Charge	Polarity	Monoisotopic
1	309.05	9626	1.24				8	427.20	219214	28.30			
2	326.05	23353	3.01				9	428.20	301152	38.88			
3	328.05	10390	1.34				10	429.20	65746	8.49			
4	370.10	104600	13.50				11	430.15	9091	1.17			
5	371.15	22369	2.89				12	448.20	38394	4.96			
6	372.10	34892	4.50				13	449.25	11168	1.44			
7	426.15	774618	100.00				14	450.20	12842	1.66			

Figure S32. LC-MS chromatogram of **17**.

^{1}H NMR (500 MHz, $CDCl_3$) δ 8.54 (d, J = 5.4 Hz, 1H), 7.99 – 7.91 (m, 2H), 7.61 – 7.50 (m, 3H), 7.30 (d, J = 5.4 Hz, 1H), 7.20 (d, J = 13.0 Hz, 3H), 3.96 – 3.71 (m, 2H), 3.63 (ddd, J = 16.8, 9.4, 4.7 Hz, 1H), 3.56 – 3.45 (m, 2H), 3.13 (ddd, J = 13.1, 9.4, 3.8 Hz, 2H), 2.93 (ddd, J = 15.2, 8.5, 4.7 Hz, 1H), 2.64 – 2.53 (m, 1H), 2.49 – 2.27 (m, 3H), 1.46 (s, 9H).

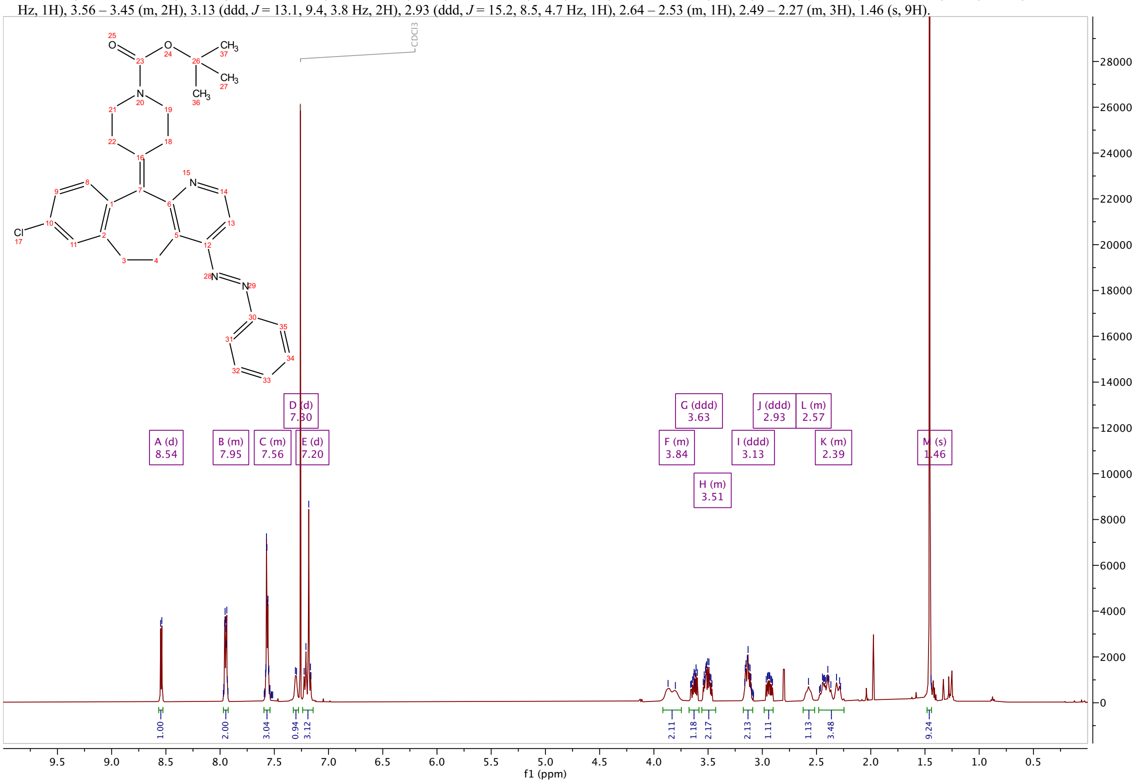

Figure S33. ^{1}H NMR spectrum of **18**.

^{13}C NMR (126 MHz, $CDCl_3$) δ 157.5*, 154.9, 152.8, 139.5, 136.8, 133.5, 132.9, 132.1*, 131.1, 129.5, 129.3, 126.6, 123.8, 109.0, 79.8, 45.1*, 31.9, 31.0, 28.6, 26.0.

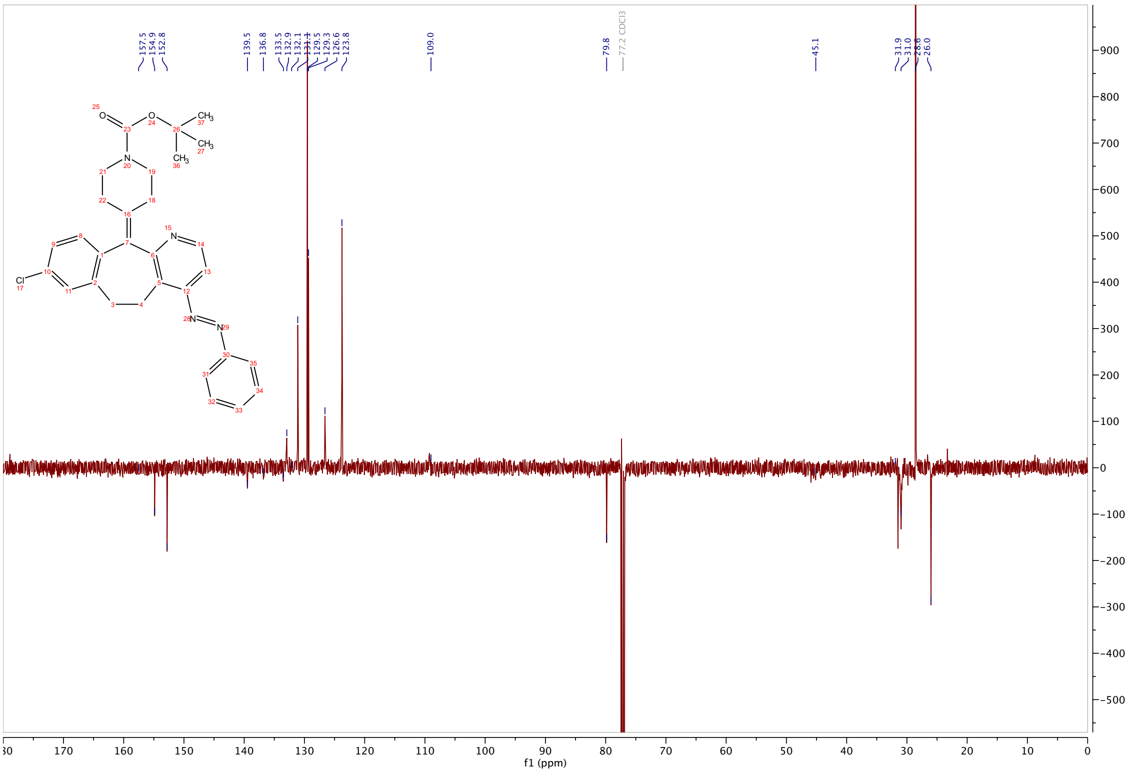

Figure S34. ^{13}C NMR spectrum of **18**.

Acquired by	: Admin
Date Acquired	: 10/21/2024 9:18:00 AM
Sample Name	: DAVE01-097-2
Sample ID	:
Tray#	: 1
Vial#	: 2
Injection Volume	: 5
Data File	: C:\LabSolutions\Data\2024\2024-wk43\DAVE01-097-2.lcd
Background File	: azoblanco 21102024.lcd
Method File	: Method SCAN ACID standard azo.lcm
Report Format	: DefaultLCMS.lcr
Tuning File	: C:\LabSolutions\Tuning File\Tuning-ESI-pos-neg01072015.lct
Processed by	: Admin
Modified Date	: 10/22/2024 1:54:18 PM

Chromatogram
DAVE01-097-2 C:\LabSolutions\Data\2024\2024-wk43\DAVE01-097-2.lcd

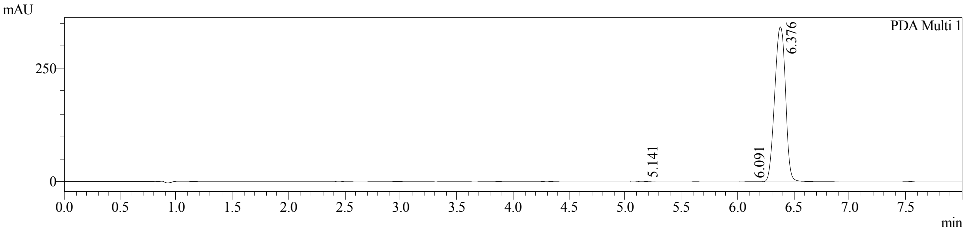

1 PDA Multi 1 / 254nm 4nm

PeakTable

PDA Ch1 254nm 4nm

Peak#	Ret. Time	Area	Height	Name	Area %
1	5.141	7374	1364		0.317
2	6.091	2306	275		0.099
3	6.376	2318591	343597		99.584
Total		2328271	345236		100.000

MS Chromatogram
DAVE01-097-2 C:\LabSolutions\Data\2024\2024-wk43\DAVE01-097-2.lcd

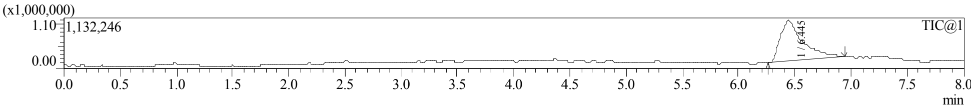

MS Spectrum Graph

#:1 Ret.Time:Averaged 6.430-6.450(Scan#:644-646)
BG Mode:Calc 6.260<->6.940(627<->695)
Mass Peaks:15 Base Peak:515.30(448847) Polarity:Pos Segment1 - Event1

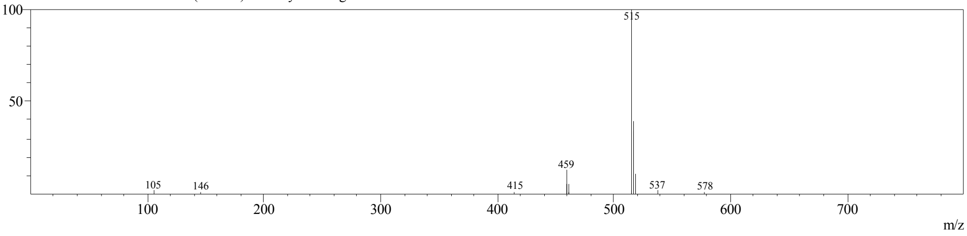

MS Spectrum Table

#:1 Ret.Time:
BG Mode:Calc 6.260<->6.940(627<->695)
Mass Peaks:15 Base Peak:515.30(448847) Polarity:Pos Segment1 - Event1

#	m/z	Abs.Inten.	Rel.Inten.	Charge	Polarity	Monoisotopic
1	105.00	7097	1.58			
2	145.90	4673	1.04			
3	415.30	5387	1.20			
4	459.20	56695	12.63			
5	460.25	24489	5.46			
6	461.15	21243	4.73			
7	462.15	4870	1.09			
8	514.50	6844	1.52			
9	515.30	448847	100.00			
10	516.30	152781	34.04			
11	517.30	175445	39.09			
12	518.25	50574	11.27			
13	519.20	9604	2.14			
14	537.25	7299	1.63			
15	578.10	4721	1.05			

Figure S35. LC-MS chromatogram of **18**.

^{1}H NMR (500 MHz, $CDCl_3$) δ 8.51 (d, *J* = 5.2 Hz, 1H), 7.98 – 7.88 (m, 2H), 7.59 – 7.49 (m, 3H), 7.21 (d, *J* = 5.3 Hz, 1H), 7.19 – 7.16 (m, 2H), 7.14 (dd, *J* = 7.8, 2.1 Hz, 1H), 3.70 – 3.60 (m, 1H), 3.55 – 3.44 (m, 2H), 3.13 – 3.05 (m, 2H), 2.93 (ddd, *J* = 15.4, 9.6, 4.6 Hz, 1H), 2.75 (dddd, *J* = 15.6, 12.2, 9.5, 3.7 Hz, 2H), 2.51 – 2.32 (m, 4H).

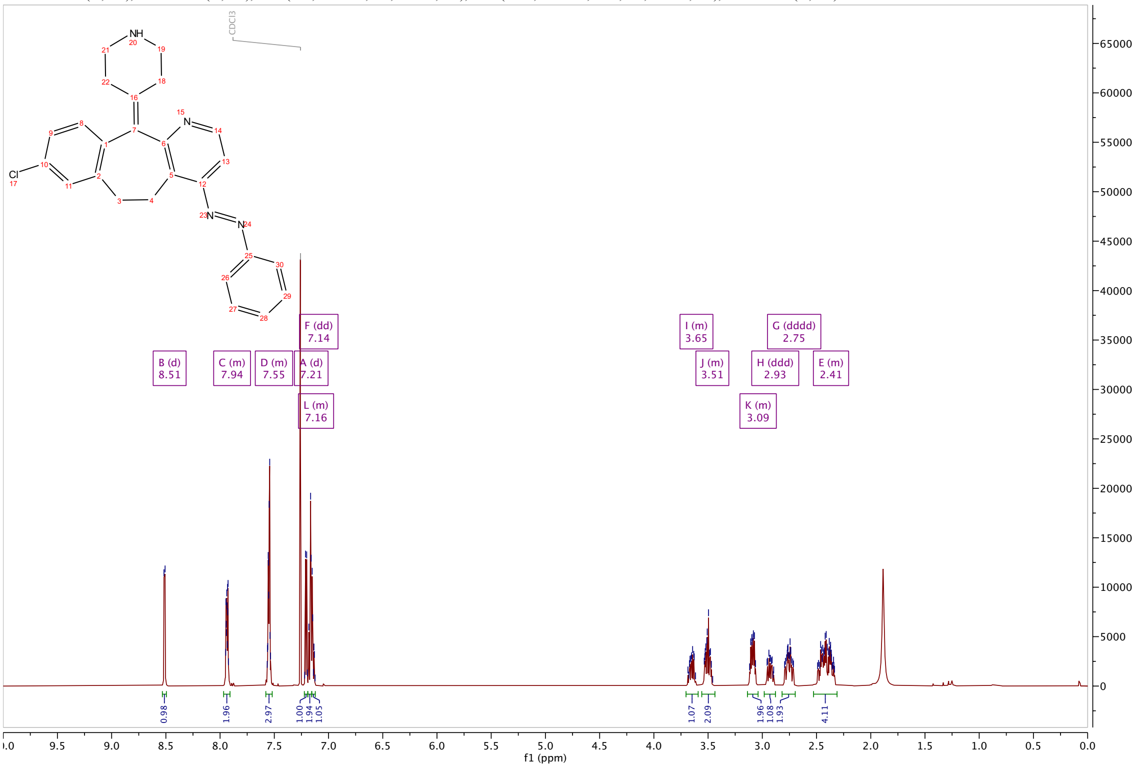

Figure S36. ^{1}H NMR spectrum of **10c.**

^{13}C NMR (126 MHz, $CDCl_3$) δ 160.8, 155.9, 152.8, 147.8, 139.7, 139.5, 137.3, 133.0, 132.7, 132.4, 131.1, 131.0, 129.4, 129.4, 126.2, 123.6, 108.4, 48.2, 48.1, 32.6, 31.9, 25.7.

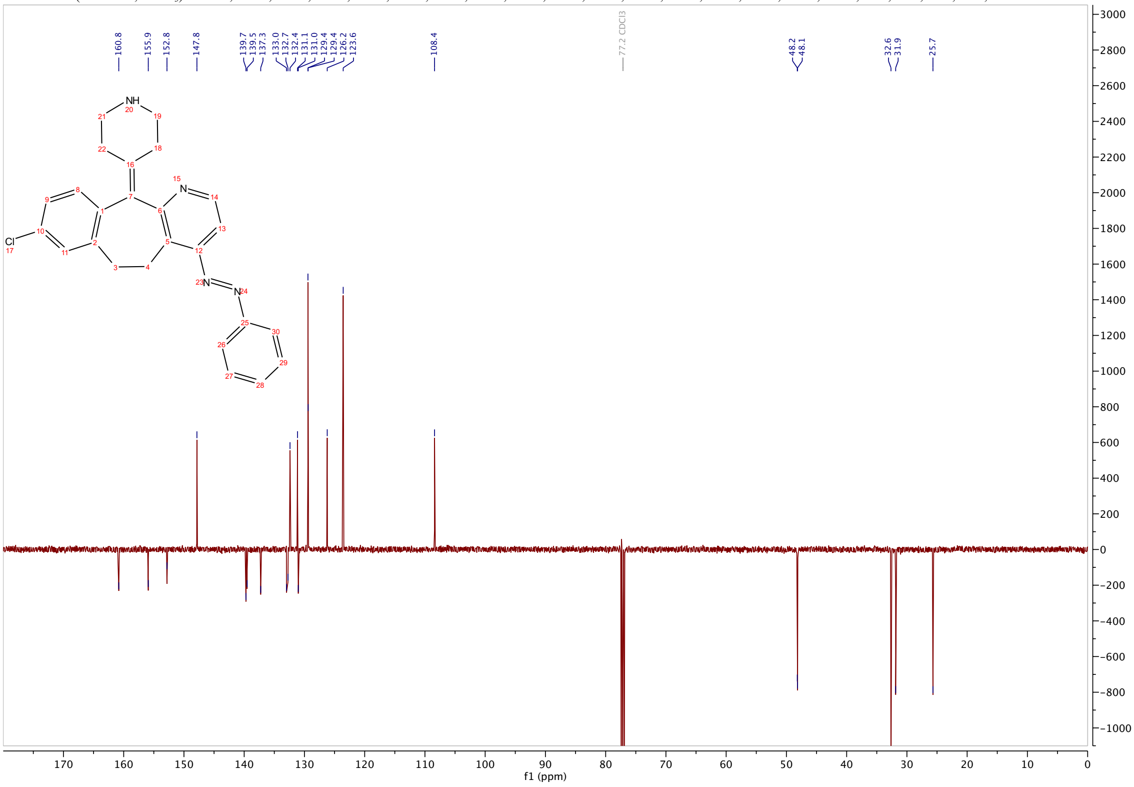

Figure S37. ^{13}C NMR spectrum of **10c**.

Acquired by : Admin
Date Acquired : 10/24/2024 4:11:41 PM
Sample Name : DAVE01-101-3
Sample ID :
Tray# : 1
Vial# : 9
Injection Volume : 5
Data File : C:\LabSolutions\Data\2024\2024-wk43\DAVE01-101-3.lcd
Background File : azoblanco 25102024.lcd
Method File : Method SCAN ACID standard azo.lcm
Report Format : DefaultLCMS.lcr
Tuning File : C:\LabSolutions\Tuning File\Tuning-ESI-pos-neg01072015.lct
Processed by : Admin
Modified Date : 10/25/2024 10:17:03 AM

Chromatogram
DAVE01-101-3 C:\LabSolutions\Data\2024\2024-wk43\DAVE01-101-3.lcd

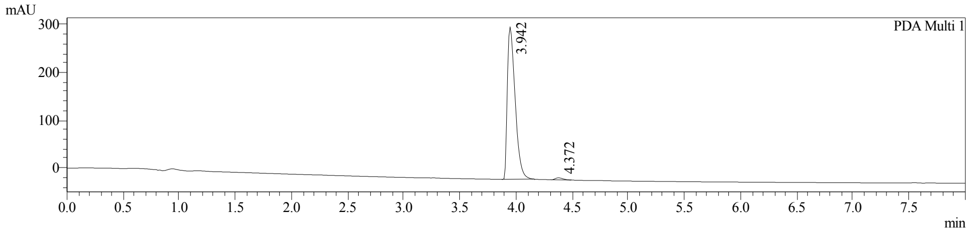

1 PDA Multi 1 / 254nm 4nm

PeakTable

PDA Ch1 254nm 4nm

Peak#	Ret. Time	Area	Height	Name	Area %
1	3.942	1535800	318992		98.662
2	4.372	20828	4288		1.338
Total		1556628	323280		100.000

MS Chromatogram
DAVE01-101-3 C:\LabSolutions\Data\2024\2024-wk43\DAVE01-101-3.lcd

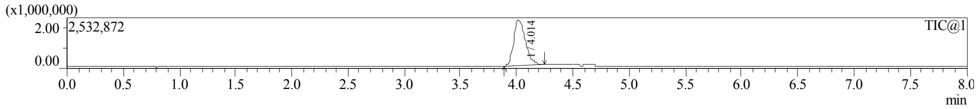

MS Spectrum Graph

#:1 Ret.Time:Averaged 4.000-4.020(Scan#:401-403)
BG Mode:Calc 3.890<->4.250(390<->426)
Mass Peaks:19 Base Peak:415.15(676169) Polarity:Pos Segment1 - Event1

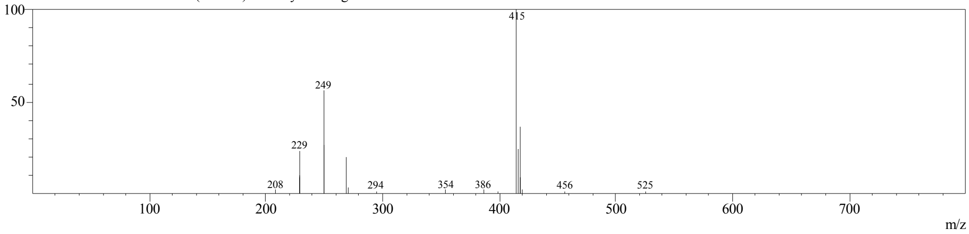

MS Spectrum Table

#:1 Ret.Time:
BG Mode:Calc 3.890<->4.250(390<->426)
Mass Peaks:19 Base Peak:415.15(676169) Polarity:Pos Segment1 - Event1

#	m/z	Abs.Inten.	Rel.Inten.	Charge	Polarity	Monoisotopic
1	208.15	10556	1.56			
2	228.55	67823	10.03			
3	228.75	154920	22.91			
4	229.75	59008	8.73			
5	249.15	376319	55.65			
6	250.05	176082	26.04			
7	269.75	131808	19.49			
8	270.80	18359	2.72			
9	294.05	8355	1.24			
10	354.20	10573	1.56			
11	386.00	10407	1.54			
12	398.05	7147	1.06			
13	415.15	676169	100.00			
14	416.20	164852	24.38			
15	417.20	248071	36.69			
16	418.20	56072	8.29			
17	419.25	9912	1.47			
18	456.25	7225	1.07			
19	525.10	7453	1.10			

Figure S38. LC-MS chromatogram of **10c.**

HRMS MedChem

Analysis Info

Analysis Name	D:\Data\ServiceMS\Hans\2024-wk45\DAVE VUF26843_11-5-2024_09-37-02_ServiceMs Hystar.d	Acquisition Date	11/5/2024 9:37:58 AM
Method	ServiceMs Hystar.m	Operator	Demo User
Sample Name	DAVE VUF26843	Instrument	impact II
Comment			

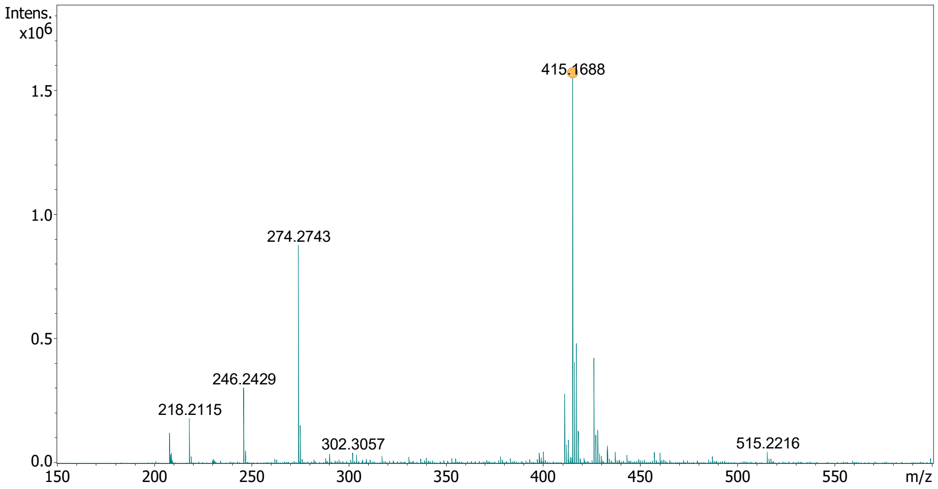

Meas. m/z	#	Ion Formula	m/z	err [ppm]	mSigma	#mSigma	Score	rdb	e^- Conf	N-Rule
415.1688	1	C25H24ClN4	415.1684	-0.9	24.7	1	100.00	19.0	even	ok

Bruker Compass DataAnalysis 6.0 printed: 11/5/2024 9:56:44 AM by: demo Page 1 of 1

Figure S39. HRMS spectrum of **10c**.

^{1}H NMR (600 MHz, $CDCl_3$) δ 9.21 (d, J = 2.5 Hz, 1H), 8.24 (d, J = 2.4 Hz, 1H), 7.20 (dΔ, J = 2.1 Hz, 1H), 7.17 (dd, J = 8.1, 2.2 Hz, 1H), 7.10 (dΔ, J = 8.1 Hz, 1H), 4.14 (q, J = 7.1 Hz, 2H), 3.85 – 3.66 (m, 2H), 3.49 – 3.40 (m, 2H), 3.22 (dddd, J = 12.8, 8.8, 8.8, 3.9 Hz, 2H), 3.01 (ddd, J = 16.9, 9.9, 4.7 Hz, 1H), 2.85 (ddd, J = 15.2, 8.2, 4.6 Hz, 1H), 2.53 – 2.45 (m, 1H), 2.43 – 2.35 (m, 1H), 2.35 – 2.28 (m, 1H), 2.28 – 2.21 (m, 1H), 1.25 (t, J = 7.1 Hz, 3H).

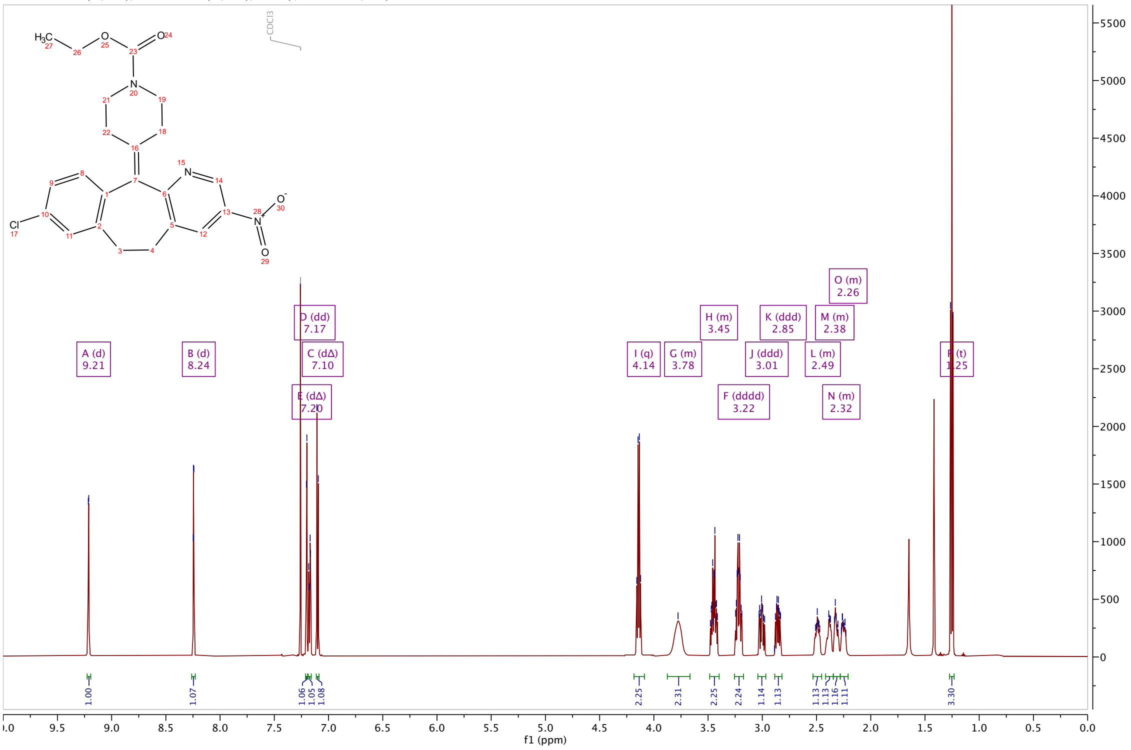

Figure S40. ^{1}H NMR spectrum of **19**.

^{13}C NMR (151 MHz, $CDCl_3$) δ 162.9, 155.6, 143.0, 142.1, 140.4, 139.1, 136.5, 134.7, 133.8, 132.9, 132.6, 130.9, 129.2, 126.8, 61.6, 44.9, 44.8, 31.8, 31.2, 14.8.

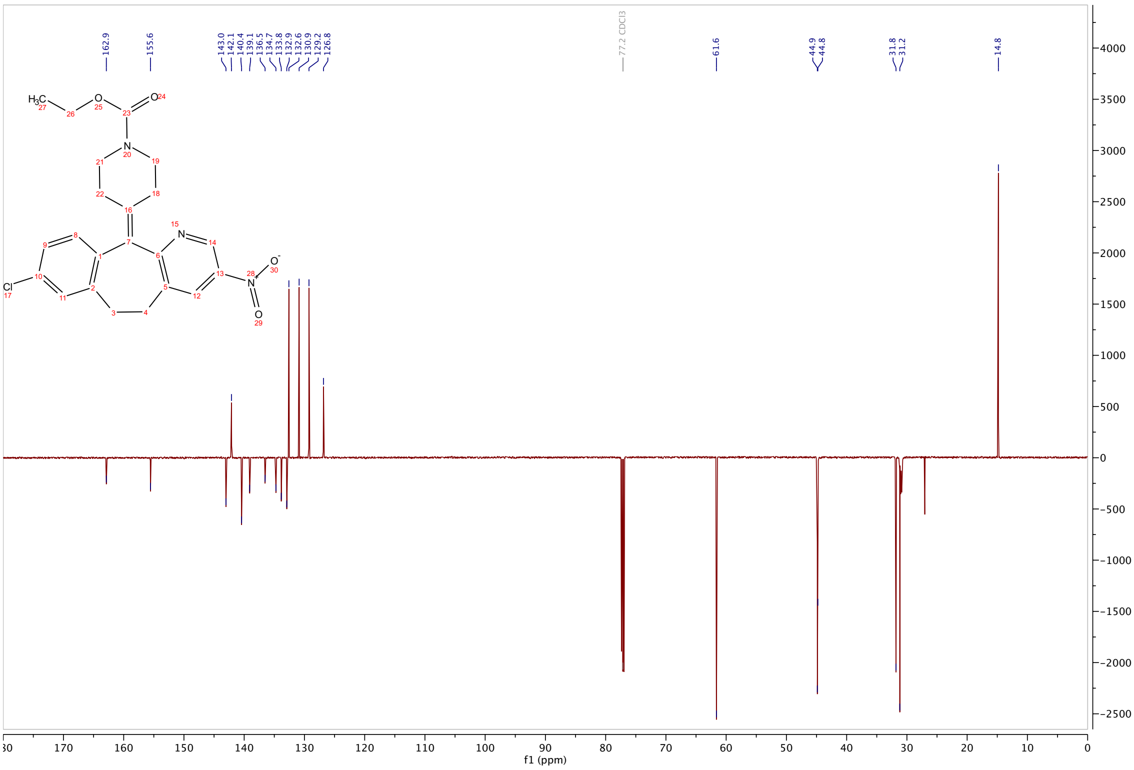

Figure S41. ^{13}C NMR spectrum of **19**.

Acquired by	: Admin
Date Acquired	: 4/30/2024 9:28:10 AM
Sample Name	: DAVE01-011-2
Sample ID	:
Tray#	: 1
Vial#	: 2
Injection Volume	: 1
Data File	: C:\LabSolutions\Data\2024\2024-wk18\DAVE01-011-2.lcd
Background File	: blanco 30042024.lcd
Method File	: Method SCAN ACID standard.lcm
Report Format	: DefaultLCMS.lcr
Tuning File	: C:\LabSolutions\Tuning File\Tuning-ESI-pos-neg01072015.lct
Processed by	: Admin
Modified Date	: 4/30/2024 9:40:15 AM

Chromatogram
DAVE01-011-2 C:\LabSolutions\Data\2024\2024-wk18\DAVE01-011-2.lcd

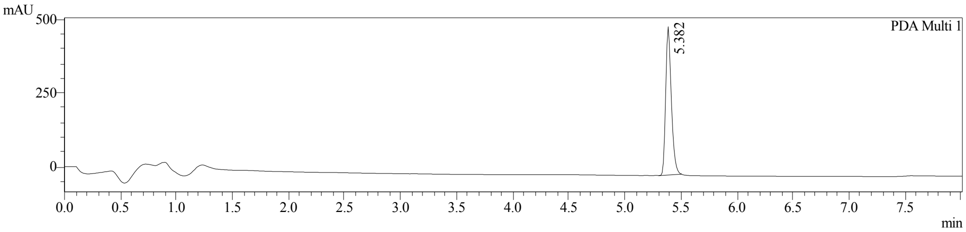

1 PDA Multi 1 / 230nm 4nm

PeakTable

PDA Ch1 230nm 4nm

Peak#	Ret. Time	Area	Height	Name	Area %
1	5.382	1790572	503347		100.000
Total		1790572	503347		100.000

MS Chromatogram
DAVE01-011-2 C:\LabSolutions\Data\2024\2024-wk18\DAVE01-011-2.lcd

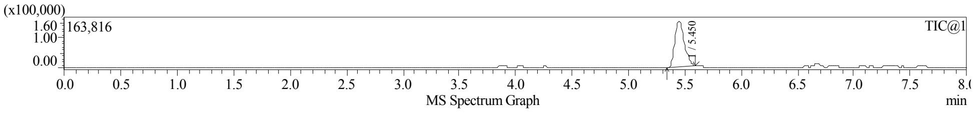

MS Spectrum Graph

#:1 Ret.Time:Averaged 5.430-5.470(Scan#:544-548)
BG Mode:None
Mass Peaks:11 Base Peak:428.15(79560) Polarity:Pos Segment1 - Event1

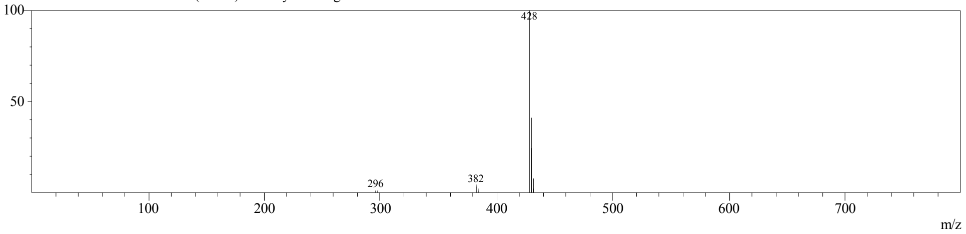

MS Spectrum Table

#:1 Ret.Time:
BG Mode:None
Mass Peaks:11 Base Peak:428.15(79560) Polarity:Pos Segment1 - Event1

#	m/z	Abs.Inten.	Rel.Inten.	Charge	Polarity	Monoisotopic
1	295.80	1520	1.91			
2	296.80	1458	1.83			
3	382.10	3734	4.69			
4	383.00	2503	3.15			
5	384.15	1779	2.24			
6	385.05	1102	1.39			
7	428.15	79560	100.00			
8	429.15	19625	24.67			
9	430.15	32726	41.13			
10	431.15	6367	8.00			
11	432.05	2032	2.55			

Figure S42. LC-MS chromatogram of **19**.

^{1}H NMR (500 MHz, DMSO) δ 7.71 (d, J = 2.6 Hz, 1H), 7.29 (d, J = 2.3 Hz, 1H), 7.19 (dd, J = 8.1, 2.3 Hz, 1H), 7.02 (dΔ, J = 8.1 Hz, 1H), 6.66 (d, J = 2.6 Hz, 1H), 4.03 (qd, J = 7.1, 1.7 Hz, 2H), 3.60 (dt, J = 12.7, 5.2 Hz, 2H), 3.31 – 3.08 (m, 4H), 2.78 – 2.61 (m, 2H), 2.36 – 2.08 (m, 4H), 1.22 – 1.12 (m, 3H).

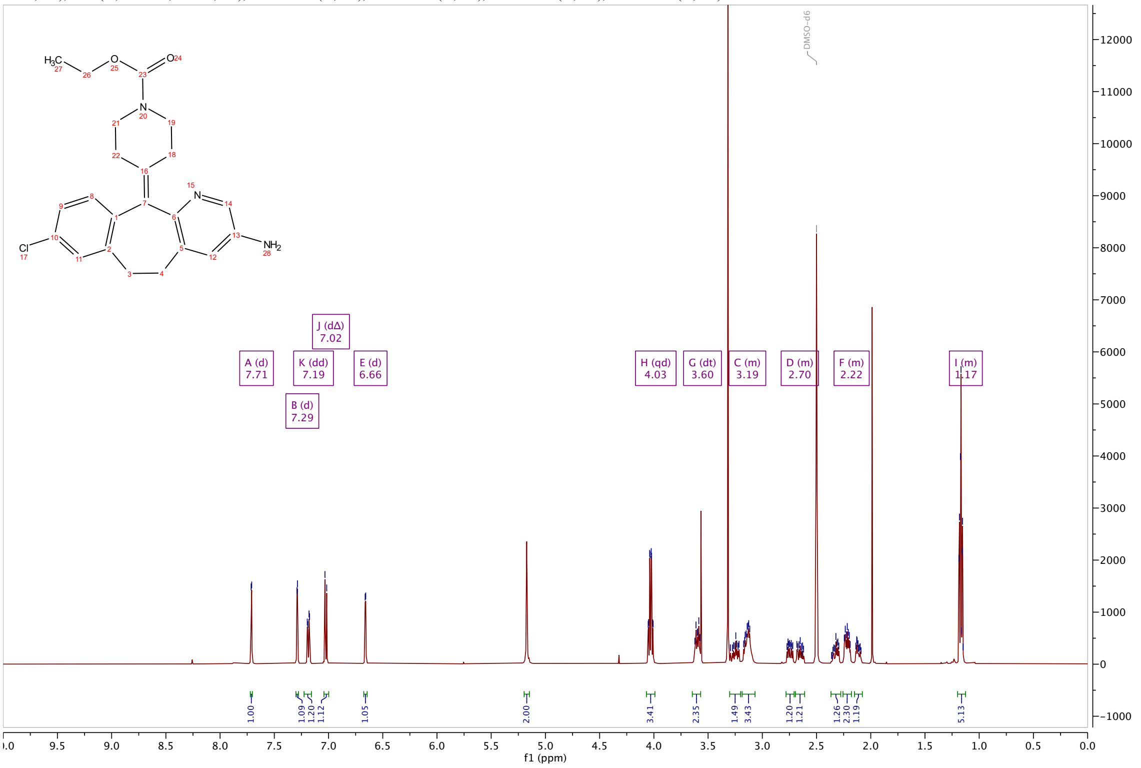

Figure S43. ^{1}H NMR spectrum of **20**.

Acquired by	: Admin
Date Acquired	: 5/2/2024 9:34:03 AM
Sample Name	: DAVE01-012-1
Sample ID	:
Tray#	: 1
Vial#	: 24
Injection Volume	: 1
Data File	: C:\LabSolutions\Data\2024\2024-wk18\DAVE01-012-1.lcd
Background File	: blanco 02052024.lcd
Method File	: Method SCAN ACID standard.lcm
Report Format	: DefaultLCMS.lcr
Tuning File	: C:\LabSolutions\Tuning File\Tuning-ESI-pos-neg01072015.lct
Processed by	: Admin
Modified Date	: 5/2/2024 9:52:12 AM

Chromatogram
DAVE01-012-1 C:\LabSolutions\Data\2024\2024-wk18\DAVE01-012-1.lcd

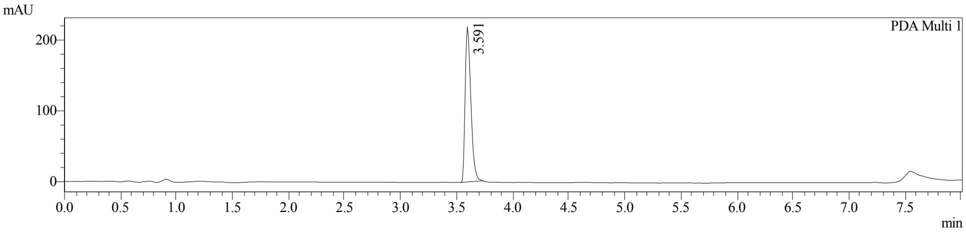

1 PDA Multi 1 / 230nm 4nm

PeakTable

PDA Ch1 230nm 4nm

Peak#	Ret. Time	Area	Height	Name	Area %
1	3.591	769836	218726		100.000
Total		769836	218726		100.000

MS Chromatogram
DAVE01-012-1 C:\LabSolutions\Data\2024\2024-wk18\DAVE01-012-1.lcd

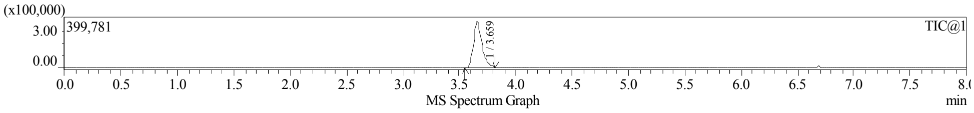

MS Spectrum Graph

#:1 Ret.Time:Averaged 3.650-3.690(Scan#:366-370)
BG Mode:None
Mass Peaks:5 Base Peak:398.10(213796) Polarity:Pos Segment1 - Event1

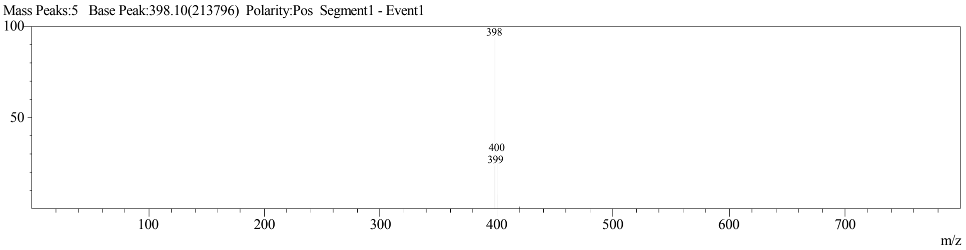

MS Spectrum Table

#:1 Ret.Time:
BG Mode:None
Mass Peaks:5 Base Peak:398.10(213796) Polarity:Pos Segment1 - Event1

#	m/z	Abs.Inten.	Rel.Inten.	Charge	Polarity	Monoisotopic
1	398.10	213796	100.00			
2	399.10	50658	23.69			
3	400.15	64902	30.36			
4	401.15	16961	7.93			
5	420.15	2420	1.13			

Figure S44. LC-MS chromatogram of **20**.

^{1}H NMR (600 MHz, $CDCl_3$) δ 9.01 (d, *J* = 2.2 Hz, 1H), 7.98 – 7.94 (m, 1H), 7.93 – 7.89 (m, 2H), 7.57 – 7.48 (m, 3H), 7.21 – 7.19 (m, 1H), 7.19 – 7.16 (m, 2H), 4.14 (q, *J* = 7.1 Hz, 2H), 3.94 – 3.74 (m, 2H), 3.53 – 3.37 (m, 2H), 3.17 (dddd, *J* = 13.1, 9.4, 3.9 Hz, 2H), 3.00 (ddd, *J* = 15.7, 9.6, 4.6 Hz, 1H), 2.87 (ddd, *J* = 15.2, 8.4, 4.6 Hz, 1H), 2.63 – 2.51 (m, 1H), 2.46 – 2.31 (m, 3H), 1.26 (t, *J* = 7.1 Hz, 3H).

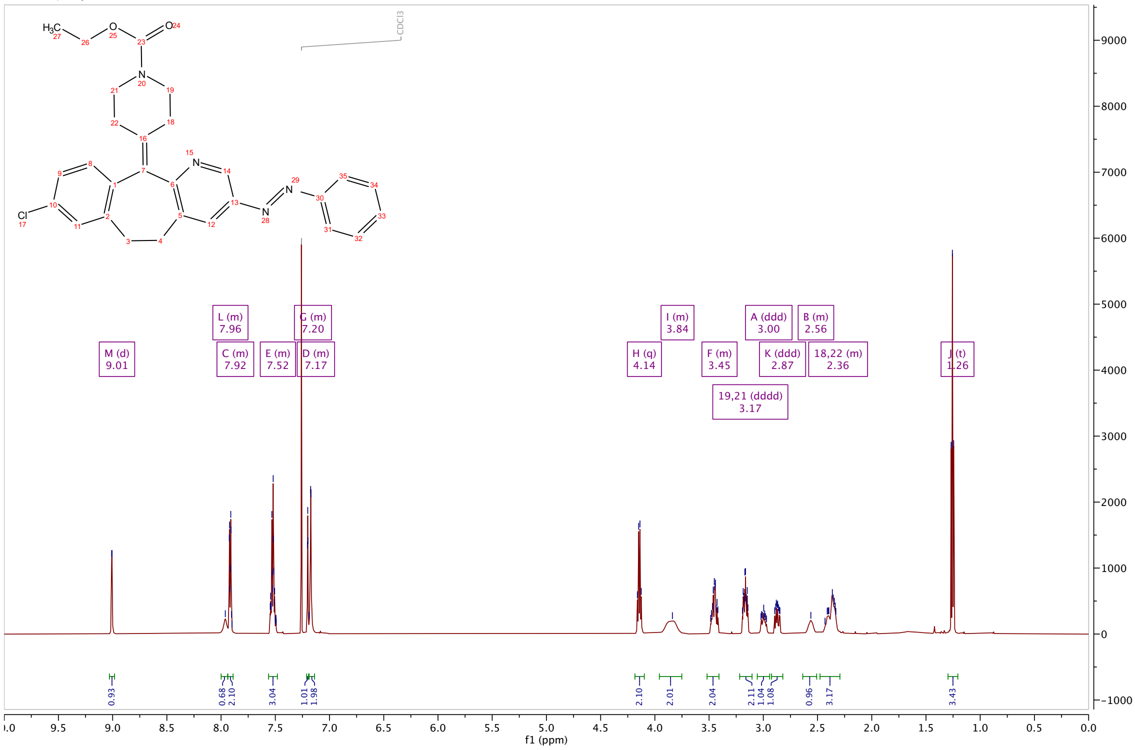

Figure S45. ^{1}H NMR spectrum of **21**.

^{13}C NMR (151 MHz, $CDCl_3$) δ 159.3, 155.6, 152.6, 147.0, 144.2, 139.4, 139.1, 137.1, 134.7, 133.4, 131.9, 130.9, 129.4, 129.2, 128.5, 126.6, 123.2, 61.5, 44.9, 31.8, 31.6, 31.0, 14.8.

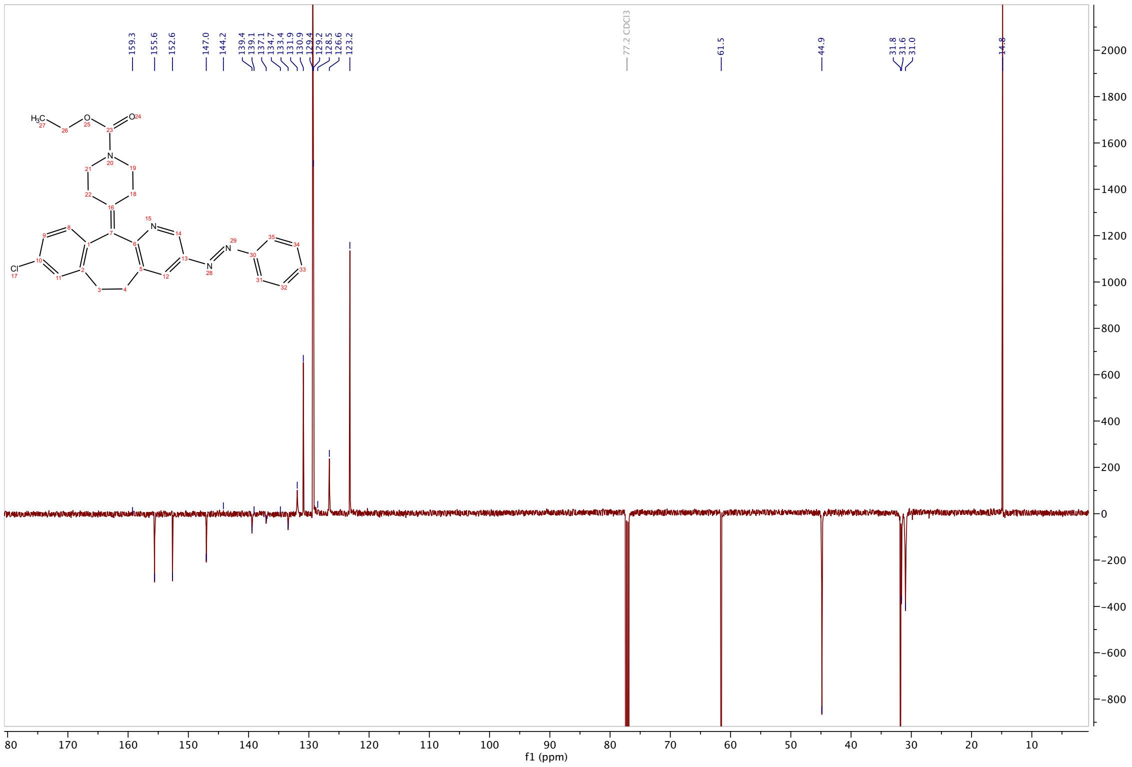

Figure S46. ^{13}C NMR spectrum of **21**.

Acquired by	: Admin
Date Acquired	: 5/7/2024 2:18:30 PM
Sample Name	: DAVE01-014-4
Sample ID	:
Tray#	: 1
Vial#	: 15
Injection Volume	: 1
Data File	: C:\LabSolutions\Data\2024\2024-wk19\DAVE01-014-4.lcd
Background File	: azoblanco 07052024.lcd
Method File	: Method SCAN ACID standard azo.lcm
Report Format	: DefaultLCMS.lcr
Tuning File	: C:\LabSolutions\Tuning File\Tuning-ESI-pos-neg01072015.lct
Processed by	: Admin
Modified Date	: 5/7/2024 3:25:14 PM

Chromatogram
DAVE01-014-4 C:\LabSolutions\Data\2024\2024-wk19\DAVE01-014-4.lcd

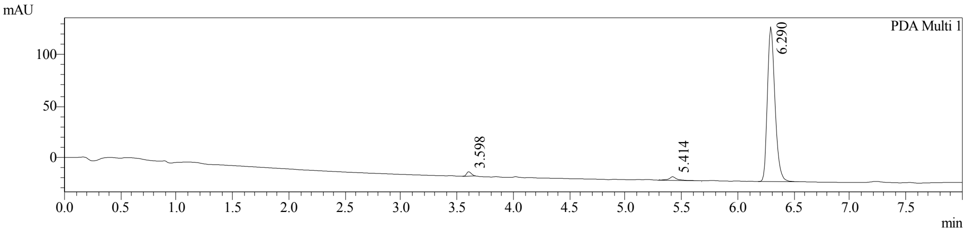

1 PDA Multi 1 / 254nm 4nm

PeakTable

PDA Ch1 254nm 4nm

Peak#	Ret. Time	Area	Height	Name	Area %
1	3.598	11642	4233		1.600
2	5.414	16745	3519		2.301
3	6.290	699433	150367		96.100
Total		727821	158118		100.000

MS Chromatogram
DAVE01-014-4 C:\LabSolutions\Data\2024\2024-wk19\DAVE01-014-4.lcd

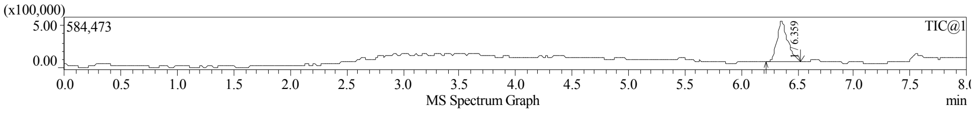

MS Spectrum Graph

#:1 Ret.Time:Averaged 6.340-6.370(Scan#:635-638)
BG Mode:None
Mass Peaks:9 Base Peak:487.20(245668) Polarity:Pos Segment1 - Event1

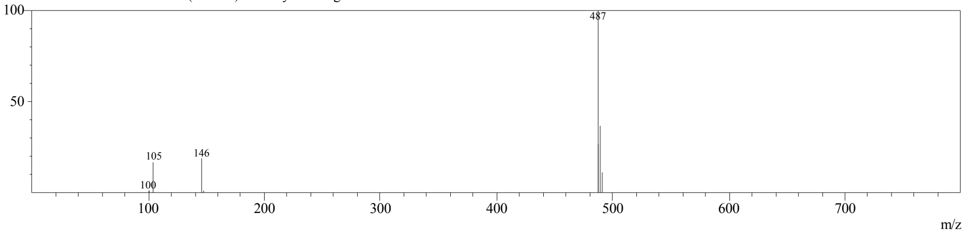

MS Spectrum Table

#:1 Ret.Time:
BG Mode:None
Mass Peaks:9 Base Peak:487.20(245668) Polarity:Pos Segment1 - Event1

#	m/z	Abs.Inten.	Rel.Inten.	Charge	Polarity	Monoisotopic
1	100.00	3023	1.23			
2	101.00	3818	1.55			
3	104.95	41752	17.00			
4	145.95	45604	18.56			
5	147.10	4536	1.85			
6	487.20	245668	100.00			
7	488.20	66098	26.91			
8	489.20	89100	36.27			
9	490.20	27154	11.05			

Figure S47. LC-MS chromatogram of **21**.

^{1}H NMR (600 MHz, DMSO) δ 8.91 (d, J = 2.3 Hz, 1H), 8.00 (d, J = 2.3 Hz, 1H), 7.91 – 7.88 (m, 2H), 7.63 – 7.57 (m, 3H), 7.32 (dΔ, J = 2.3 Hz, 1H), 7.23 (dd, J = 8.2, 2.3 Hz, 1H), 7.11 (dΔ, J = 8.2 Hz, 1H), 3.44 – 3.35 (m, 2H), 2.99 (ddd, J = 16.7, 9.1, 4.7 Hz, 1H), 2.93 – 2.85 (m, 3H), 2.60 (dddd, J = 15.0, 12.2, 8.9, 3.5 Hz, 2H), 2.33 – 2.21 (m, 2H), 2.20 – 2.12 (m, 2H).

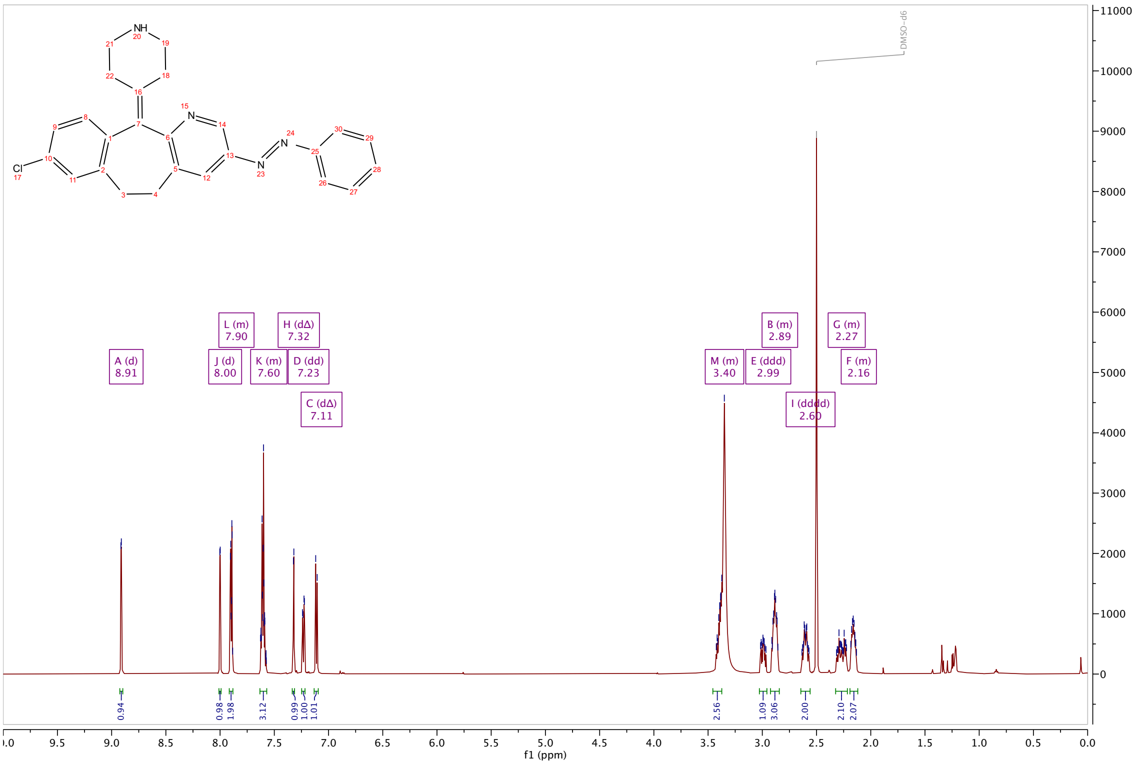

Figure S48. ^{1}H NMR spectrum of **10b**.

^{13}C NMR (151 MHz, DMSO) δ 160.2, 152.0, 146.2, 143.8, 140.4, 140.1, 137.3, 134.6, 132.0, 131.6, 131.1, 131.0, 129.6, 129.1, 127.4, 125.8, 122.7, 47.7, 47.6, 32.3, 32.2, 30.9, 30.6.

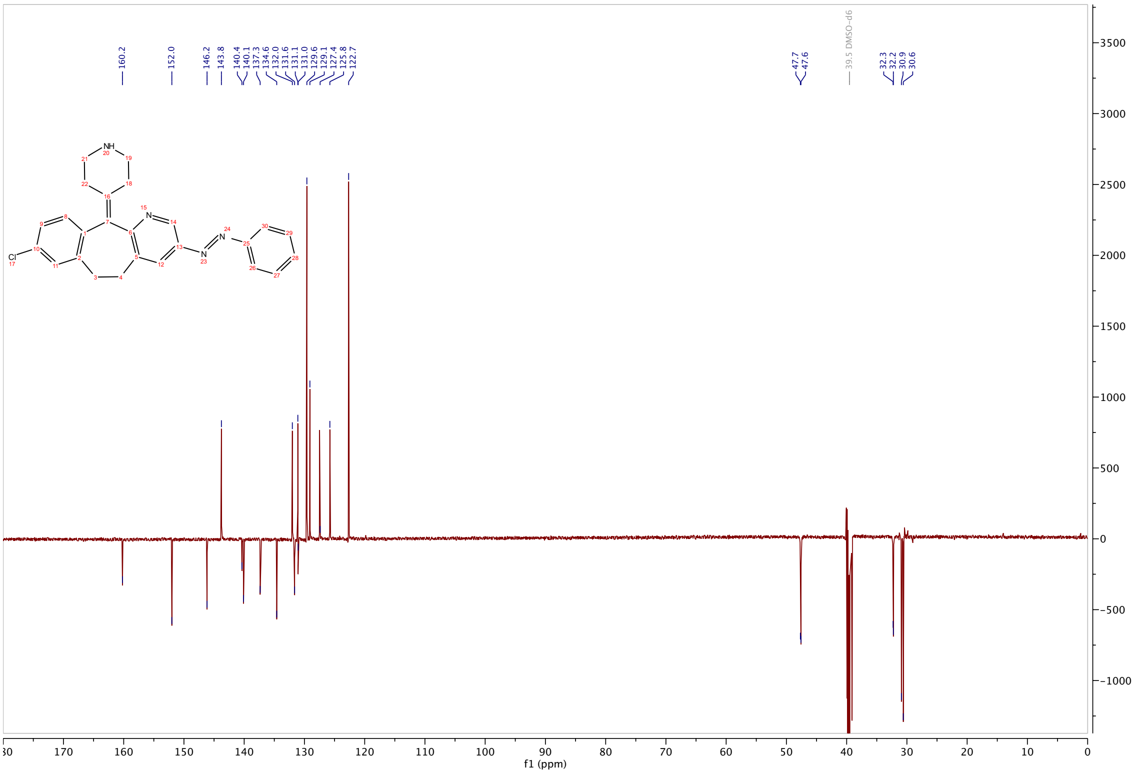

Figure S49. ^{13}C NMR spectrum of **10b**.

Acquired by	: Admin
Date Acquired	: 6/6/2024 12:05:38 PM
Sample Name	: DAVE01-021-5
Sample ID	:
Tray#	: 1
Vial#	: 10
Injection Volume	: 10
Data File	: C:\LabSolutions\Data\2024\2024-wk23\DAVE01-021-5.lcd
Background File	: azoblanco 06062024.lcd
Method File	: Method SCAN ACID standard azo.lcm
Report Format	: DefaultLCMS.lcr
Tuning File	: C:\LabSolutions\Tuning File\Tuning-ESI-pos-neg01072015.lct
Processed by	: Admin
Modified Date	: 6/6/2024 12:46:31 PM

vrije Universiteit

Chromatogram
DAVE01-021-5 C:\LabSolutions\Data\2024\2024-wk23\DAVE01-021-5.lcd

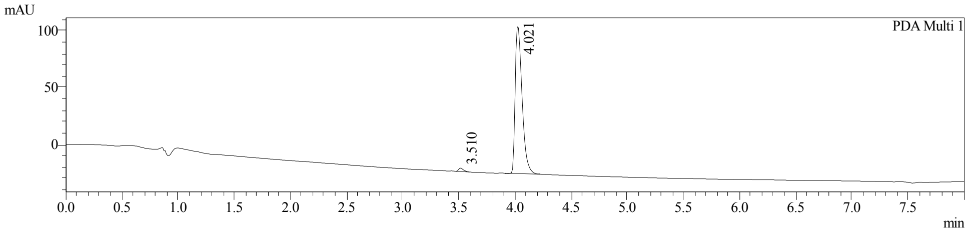

1 PDA Multi 1 / 254nm 4nm

PeakTable

PDA Ch1 254nm 4nm

Peak#	Ret. Time	Area	Height	Name	Area %
1	3.510	8754	2980		1.550
2	4.021	556116	128849		98.450
Total		564870	131829		100.000

MS Chromatogram
DAVE01-021-5 C:\LabSolutions\Data\2024\2024-wk23\DAVE01-021-5.lcd

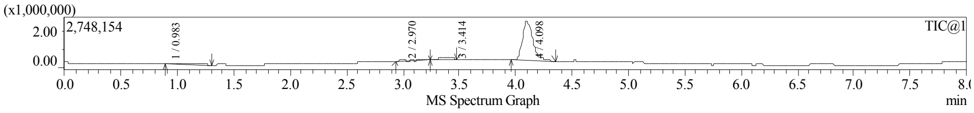

MS Spectrum Graph

#:1 Ret.Time:Averaged 4.090-4.110(Scan#:410-412)
BG Mode:Calc 3.960<->4.350(397<->436)
Mass Peaks:14 Base Peak:415.15(1110146) Polarity:Pos Segment1 - Event1

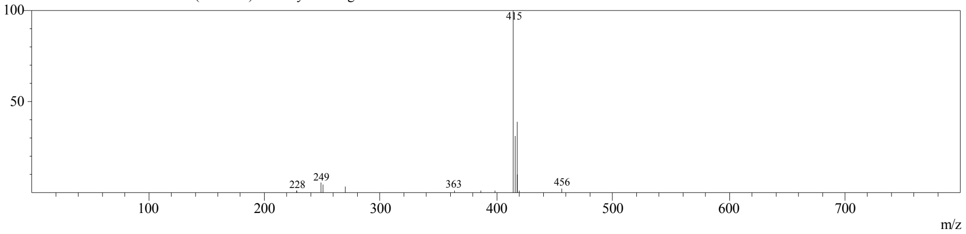

MS Spectrum Table

#:1 Ret.Time:
BG Mode:Calc 3.960<->4.350(397<->436)
Mass Peaks:14 Base Peak:415.15(1110146) Polarity:Pos Segment1 - Event1

#	m/z	Abs.Inten.	Rel.Inten.	Charge	Polarity	Monoisotopic
1	228.45	14859	1.34			
2	228.75	16842	1.52			
3	249.10	59950	5.40			
4	250.05	46654	4.20			
5	269.95	41329	3.72			
6	363.10	18531	1.67			
7	386.15	13124	1.18			

#	m/z	Abs.Inten.	Rel.Inten.	Charge	Polarity	Monoisotopic
8	398.10	15452	1.39			
9	415.15	1110146	100.00			
10	416.15	351491	31.66			
11	417.15	433500	39.05			
12	418.15	116314	10.48			
13	419.15	12753	1.15			
14	456.25	29888	2.69			

Figure S50. LC-MS chromatogram of **10b**.

HRMS MedChem

**Analysis Info**		Acquisition Date	7/31/2024 10:01:54 AM
Analysis Name	D:\Data\ServiceMS\Hans\2024-wk31\DAVE VUF26788_7-31-2024_10-00-58_ServiceMs Hystar.d		
Method	ServiceMs Hystar.m	Operator	Demo User
Sample Name	DAVE VUF26788	Instrument	impact II
Comment			

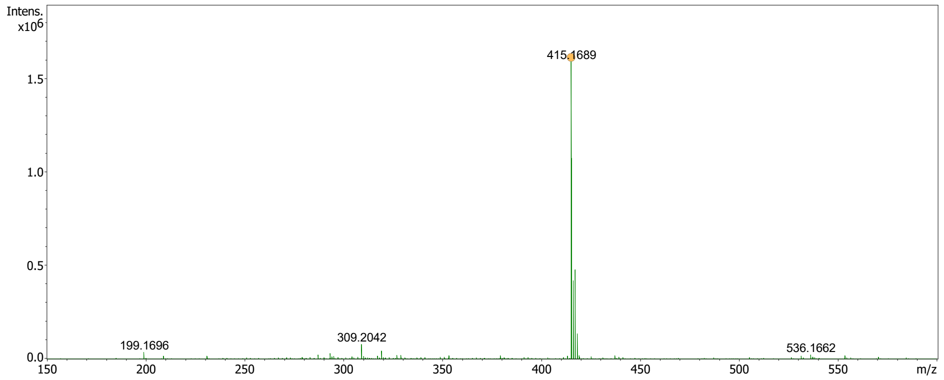

Meas. m/z	#	Ion Formula	m/z	err [ppm]	mSigma	#mSigma	Score	rdb	e⁻ Conf	N-Rule
415.1689	1	C25H24ClN4	415.1684	-1.3	28.8	1	100.00	19.0	even	ok

Bruker Compass DataAnalysis 6.0 printed: 7/31/2024 10:08:41 AM by: demo Page 1 of 1

Figure S51. HRMS spectrum of **10b**.

^{1}H NMR (500 MHz, $CDCl_3$) δ 8.38 (dd, *J* = 4.8, 1.7 Hz, 1H), 7.43 (dd, *J* = 7.6, 1.7 Hz, 1H), 7.39 – 7.33 (m, 1H), 7.12 – 7.05 (m, 2H), 6.98 (dd, *J* = 8.2, 2.3 Hz, 1H), 6.48 (s, 1H), 4.13 (q, *J* = 7.1 Hz, 2H), 3.88 – 3.72 (m, 2H), 3.44 – 3.28 (m, 2H), 3.16 – 3.05 (m, 2H), 2.90 – 2.76 (m, 2H), 2.47 (ddd, *J* = 14.2, 9.5, 4.6 Hz, 1H), 2.40 – 2.24 (m, 3H), 1.50 (s, 9H), 1.24 (t, *J* = 7.1 Hz, 3H).

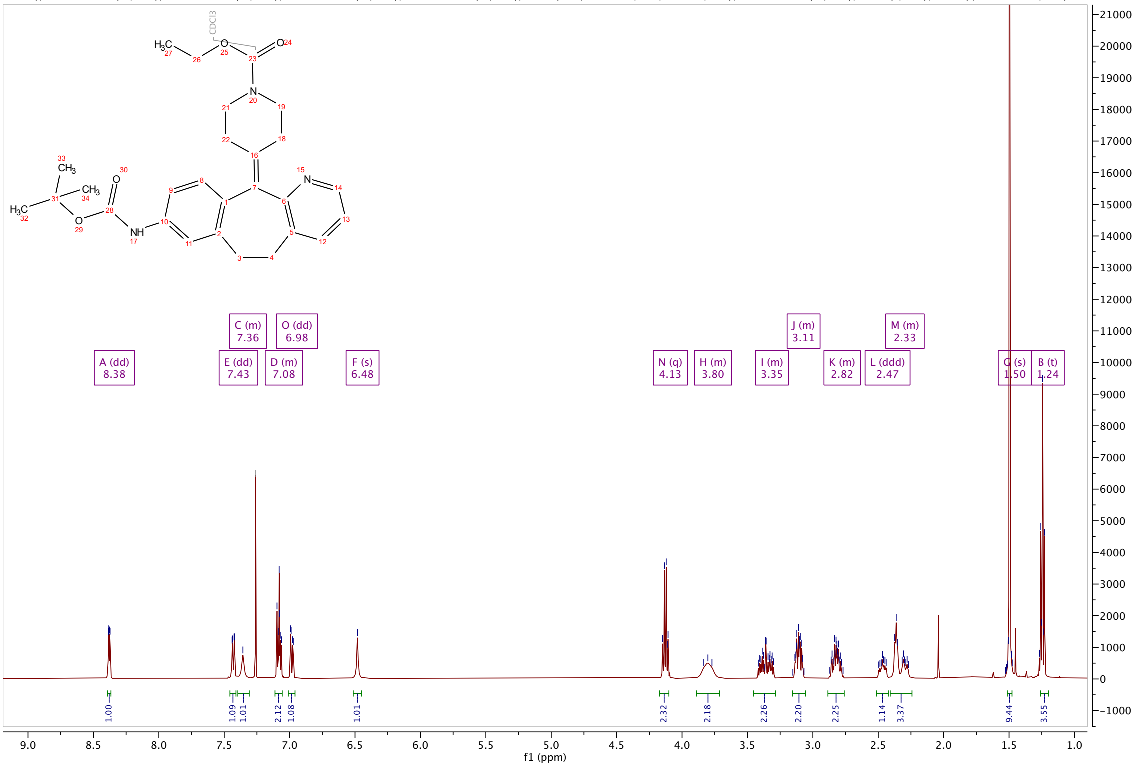

Figure S52. ^{1}H NMR spectrum of **22**.

^{13}C NMR (126 MHz, $CDCl_3$) δ 157.8, 155.7, 152.9, 146.5, 138.6, 137.7, 137.6, 136.9, 134.1, 133.9, 130.0, 122.2, 119.2, 116.4, 80.7, 61.4, 45.0, 45.0, 32.2, 31.7, 30.9, 30.7, 28.5, 14.8.

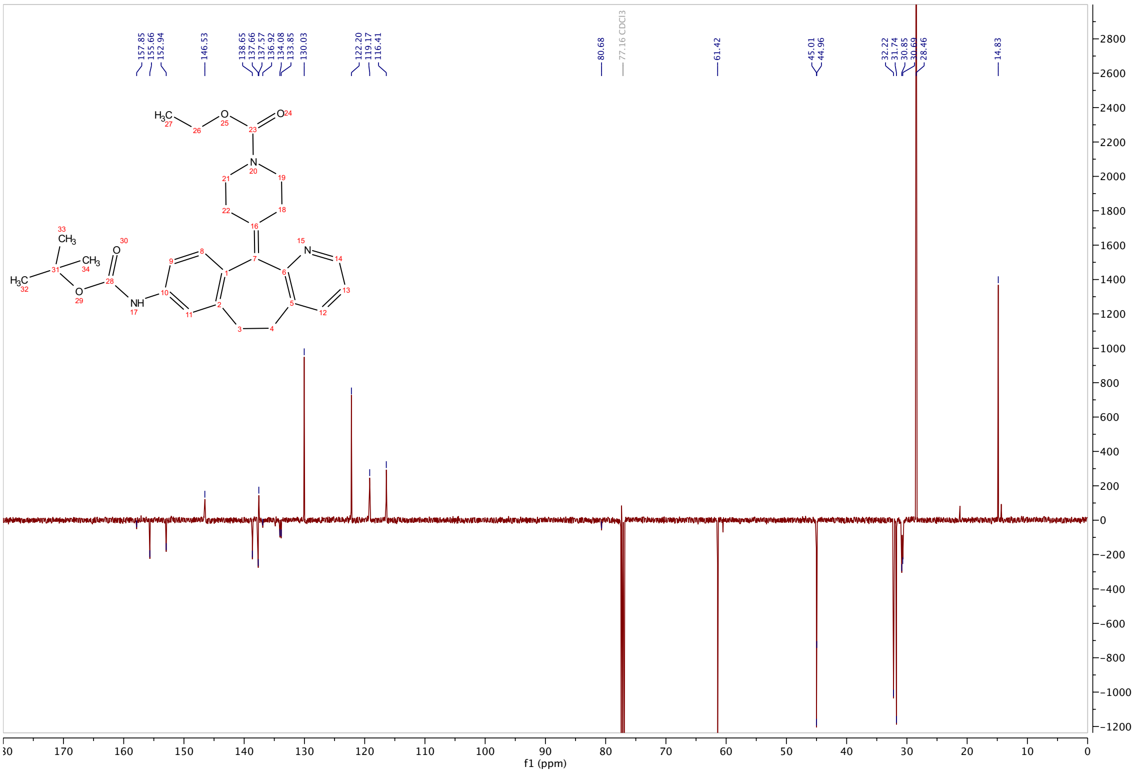

Figure S53. ^{13}C NMR spectrum of **22**.

Acquired by	: Admin
Date Acquired	: 6/21/2024 3:45:10 PM
Sample Name	: DAVE01-031-2
Sample ID	:
Tray#	: 1
Vial#	: 31
Injection Volume	: 1
Data File	: C:\LabSolutions\Data\2024\2024-wk25\DAVE01-031-2.lcd
Background File	: azoblanco 21062024.lcd
Method File	: Method SCAN ACID standard.lcm
Report Format	: DefaultLCMS.lcr
Tuning File	: C:\LabSolutions\Tuning File\Tuning-ESI-pos-neg01072015.lct
Processed by	: Admin
Modified Date	: 6/21/2024 4:04:12 PM

Chromatogram
DAVE01-031-2 C:\LabSolutions\Data\2024\2024-wk25\DAVE01-031-2.lcd

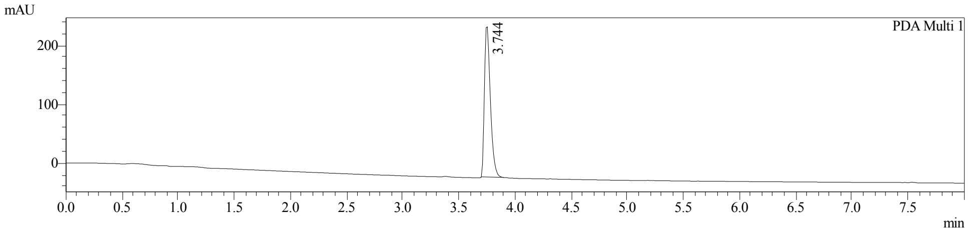

1 PDA Multi 1 / 254nm 4nm

PeakTable

PDA Ch1 254nm 4nm

Peak#	Ret. Time	Area	Height	Name	Area %
1	3.744	931944	253822		100.000
Total		931944	253822		100.000

MS Chromatogram
DAVE01-031-2 C:\LabSolutions\Data\2024\2024-wk25\DAVE01-031-2.lcd

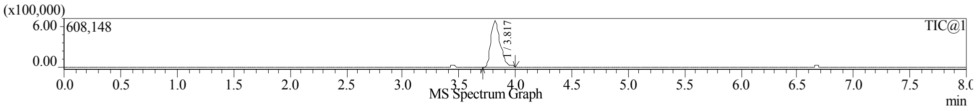

MS Spectrum Graph

#:1 Ret.Time:Averaged 3.810-3.830(Scan#:382-384)
BG Mode:Calc 3.710<->4.000(372<->401)
Mass Peaks:8 Base Peak:464.30(366392) Polarity:Pos Segment1 - Event1

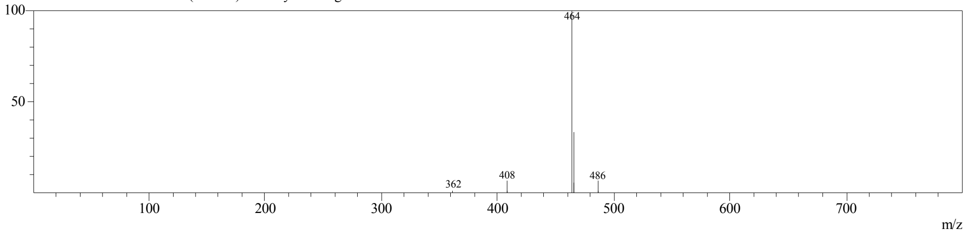

MS Spectrum Table

#:1 Ret.Time:
BG Mode:Calc 3.710<->4.000(372<->401)
Mass Peaks:8 Base Peak:464.30(366392) Polarity:Pos Segment1 - Event1

#	m/z	Abs.Inten.	Rel.Inten.	Charge	Polarity	Monoisotopic
1	362.10	5723	1.56			
2	408.20	24220	6.61			
3	409.15	6122	1.67			
4	464.30	366392	100.00			
5	465.30	120614	32.92			
6	466.30	20103	5.49			
7	486.25	23004	6.28			
8	487.30	5728	1.56			

Figure S54. LC-MS chromatogram of **22**.

^{1}H NMR (500 MHz, $CDCl_3$) δ 8.38 (dd, J = 4.8, 1.7 Hz, 1H), 7.44 (dd, J = 7.7, 1.7 Hz, 1H), 7.07 (dd, J = 7.6, 4.8 Hz, 1H), 7.00 – 6.95 (m, 1H), 6.52 – 6.47 (m, 2H), 4.13 (q, J = 7.1 Hz, 2H), 3.91 – 3.68 (m, 2H), 3.38 – 3.28 (m, 2H), 3.17 – 3.04 (m, 2H), 2.86 – 2.67 (m, 2H), 2.50 – 2.33 (m, 3H), 2.27 (ddd, J = 14.6, 4.5 Hz, 1H), 1.24 (t, J = 7.1 Hz, 3H).

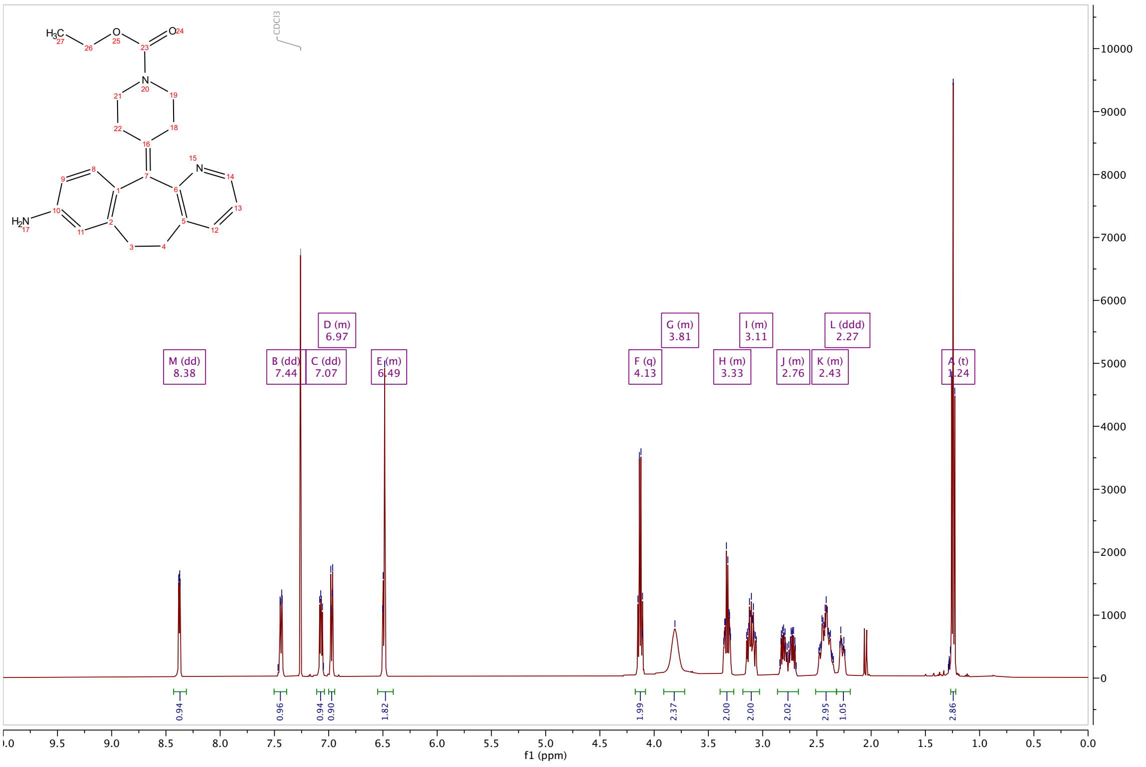

Figure S55. ^{1}H NMR spectrum of **23**.

^{13}C NMR (126 MHz, $CDCl_3$) δ 158.5, 155.7, 146.3, 145.8, 138.6, 137.4, 134.0, 130.7, 129.3, 122.1, 115.6, 113.0, 61.4, 45.1, 45.0, 32.3, 31.7, 30.8, 30.7, 14.8.

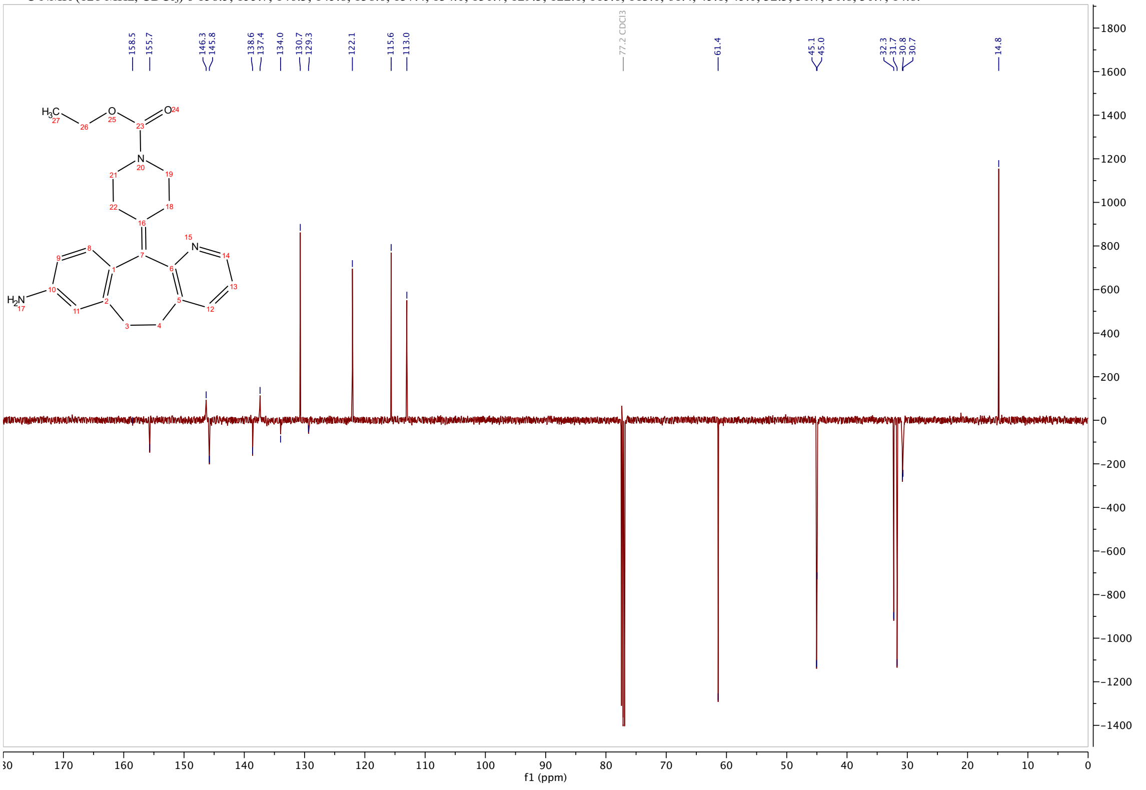

Figure S56. ^{13}C NMR spectrum of **23**.

Acquired by	: Admin
Date Acquired	: 6/28/2024 10:38:45 AM
Sample Name	: DAVE01-032-2
Sample ID	:
Tray#	: 1
Vial#	: 3
Injection Volume	: 1
Data File	: C:\LabSolutions\Data\2024\2024-wk26\DAVE01-032-2.lcd
Background File	: blanco 28062024.lcd
Method File	: Method SCAN ACID standard.lcm
Report Format	: DefaultLCMS.lcr
Tuning File	: C:\LabSolutions\Tuning File\Tuning-ESI-pos-neg01072015.lct
Processed by	: Admin
Modified Date	: 6/28/2024 10:48:03 AM

Chromatogram
DAVE01-032-2 C:\LabSolutions\Data\2024\2024-wk26\DAVE01-032-2.lcd

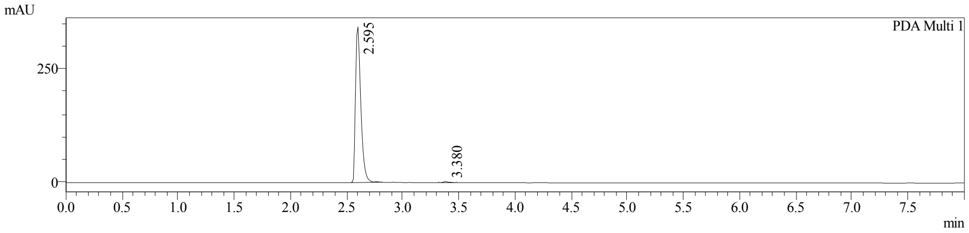

1 PDA Multi 1 / 254nm 4nm

PeakTable

PDA Ch1 254nm 4nm

Peak#	Ret. Time	Area	Height	Name	Area %
1	2.595	1125128	342317		99.268
2	3.380	8297	2421		0.732
Total		1133424	344738		100.000

MS Chromatogram
DAVE01-032-2 C:\LabSolutions\Data\2024\2024-wk26\DAVE01-032-2.lcd

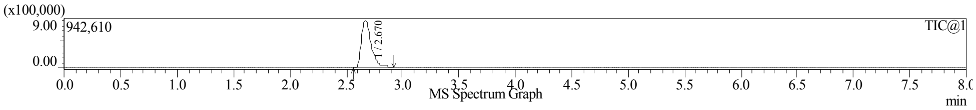

MS Spectrum Graph

#:1 Ret.Time:Averaged 2.660-2.680(Scan#:267-269)
BG Mode:Calc 2.560<->2.920(257<->293)
Mass Peaks:7 Base Peak:364.15(670726) Polarity:Pos Segment1 - Event1

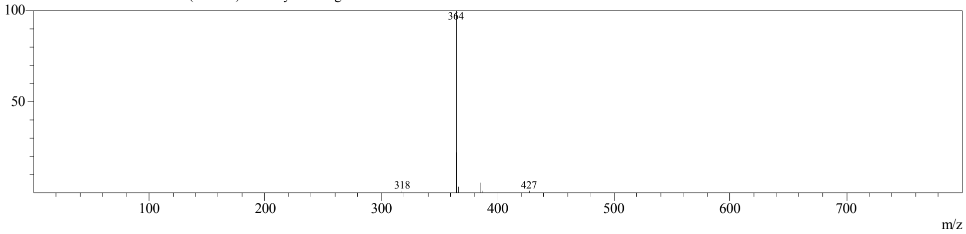

MS Spectrum Table

#:1 Ret.Time:
BG Mode:Calc 2.560<->2.920(257<->293)
Mass Peaks:7 Base Peak:364.15(670726) Polarity:Pos Segment1 - Event1

#	m/z	Abs.Inten.	Rel.Inten.	Charge	Polarity	Monoisotopic
1	318.00	7235	1.08			
2	364.15	670726	100.00			
3	365.15	151709	22.62			
4	366.10	24497	3.65			
5	386.15	39185	5.84			
6	387.10	9940	1.48			
7	427.15	7719	1.15			

Figure S57. LC-MS chromatogram of **23**.

^{1}H NMR (600 MHz, $CDCl_3$) δ 8.43 (dd, *J* = 4.8, 1.6 Hz, 1H), 7.91 – 7.85 (m, 2H), 7.77 – 7.71 (m, 2H), 7.54 – 7.42 (m, 4H), 7.36 (dΔ, *J* = 8.6 Hz, 1H), 7.13 (dd, *J* = 7.7, 4.8 Hz, 1H), 4.14 (q, *J* = 7.1 Hz, 2H), 3.92 – 3.76 (m, 2H), 3.56 – 3.48 (m, 1H), 3.41 (ddd, *J* = 15.9, 8.6, 4.6 Hz, 1H), 3.17 (ddd, *J* = 13.1, 9.0, 3.7 Hz, 2H), 3.01 – 2.89 (m, 2H), 2.54 (ddd, *J* = 14.0, 9.4, 4.6 Hz, 1H), 2.47 – 2.31 (m, 3H), 1.25 (t, *J* = 7.1 Hz, 3H).

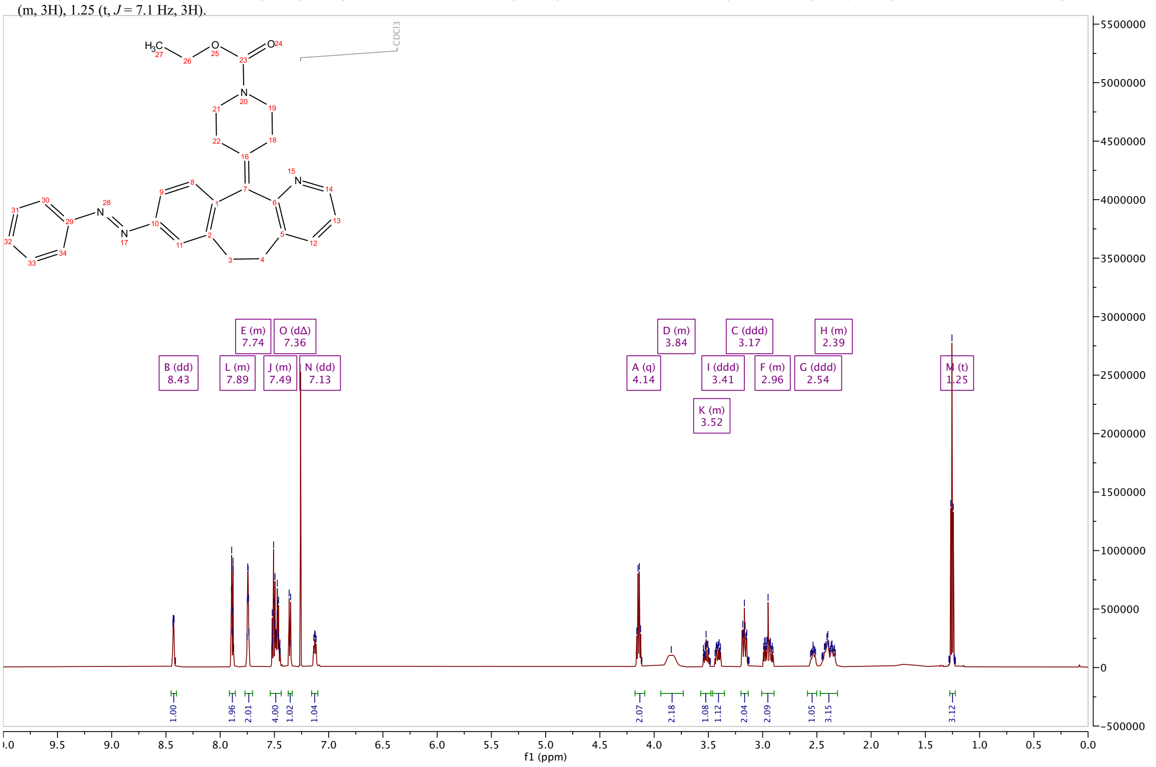

Figure S58. ^{1}H NMR spectrum of **24**.

^{13}C NMR (151 MHz, $CDCl_3$) δ 156.9, 155.7, 152.8, 152.1, 146.7, 142.4, 138.9, 138.2, 138.0, 134.6, 133.8, 131.1, 130.3, 129.2, 123.2, 122.9, 122.5, 121.2, 61.5, 45.0, 44.9, 32.0, 31.9, 31.0, 30.8, 14.8.

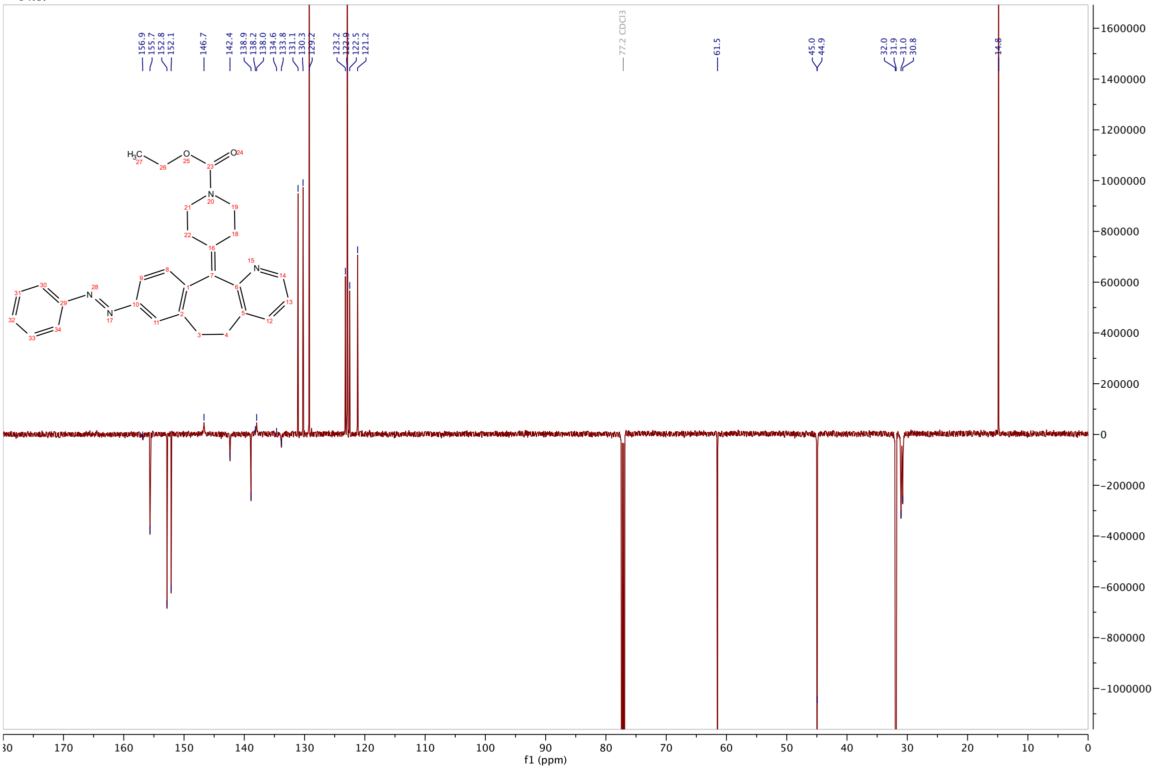

Figure S59. ^{13}C NMR spectrum of **214**.

Acquired by	: Admin
Date Acquired	: 7/3/2024 11:21:24 AM
Sample Name	: DAVE01-034-3
Sample ID	:
Tray#	: 1
Vial#	: 15
Injection Volume	: 4
Data File	: C:\LabSolutions\Data\2024\2024-wk27\DAVE01-034-3.lcd
Background File	: azoblanco 03072024.lcd
Method File	: Method SCAN ACID standard azo.lcm
Report Format	: DefaultLCMS.lcr
Tuning File	: C:\LabSolutions\Tuning File\Tuning-ESI-pos-neg01072015.lct
Processed by	: Admin
Modified Date	: 7/3/2024 11:39:13 AM

Chromatogram
DAVE01-034-3 C:\LabSolutions\Data\2024\2024-wk27\DAVE01-034-3.lcd

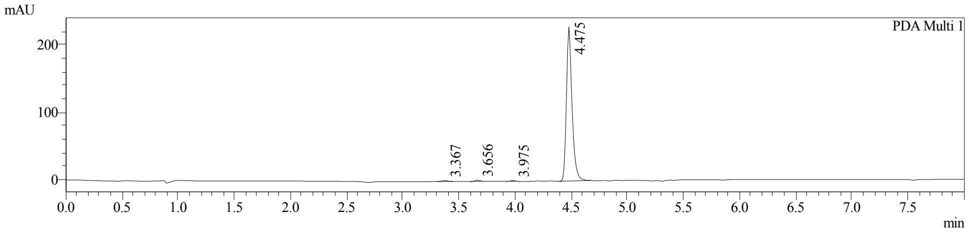

1 PDA Multi 1 / 254nm 4nm

PeakTable

PDA Ch1 254nm 4nm

Peak#	Ret. Time	Area	Height	Name	Area %
1	3.367	4533	1564		0.555
2	3.656	4810	1726		0.589
3	3.975	2720	909		0.333
4	4.475	804538	228264		98.523
Total		816601	232463		100.000

MS Chromatogram
DAVE01-034-3 C:\LabSolutions\Data\2024\2024-wk27\DAVE01-034-3.lcd

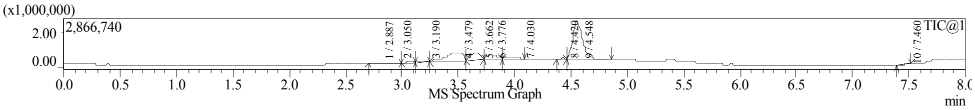

MS Spectrum Graph

#:1 Ret.Time:Averaged 4.540-4.560(Scan#:455-457)
BG Mode:Calc 4.460<->4.860(447<->487)
Mass Peaks:7 Base Peak:453.25(1706808) Polarity:Pos Segment1 - Event1

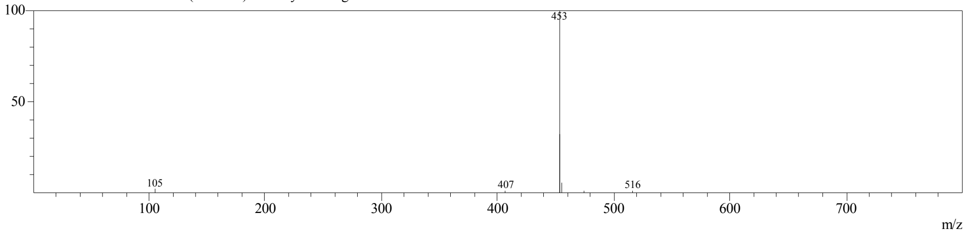

MS Spectrum Table

#:1 Ret.Time:
BG Mode:Calc 4.460<->4.860(447<->487)
Mass Peaks:7 Base Peak:453.25(1706808) Polarity:Pos Segment1 - Event1

#	m/z	Abs.Inten.	Rel.Inten.	Charge	Polarity	Monoisotopic
1	104.95	35555	2.08			
2	407.15	23172	1.36			
3	453.25	1706808	100.00			
4	454.20	551248	32.30			
5	455.25	92100	5.40			
6	475.15	26719	1.57			
7	516.30	24575	1.44			

Figure S60. LC-MS chromatogram of **24**.

^{1}H NMR (500 MHz, $CDCl_3$) δ 8.41 (dd, J = 4.8, 1.7 Hz, 1H), 7.91 – 7.85 (m, 2H), 7.76 – 7.69 (m, 2H), 7.50 (ddd, J = 8.3, 6.4, 1.8 Hz, 2H), 7.49 – 7.42 (m, 3H), 7.38 – 7.32 (m, 1H), 7.09 (dd, J = 7.7, 4.8 Hz, 1H), 3.54 (ddd, J = 13.2, 8.7, 4.5 Hz, 1H), 3.43 (ddd, J = 15.6, 7.0, 4.3 Hz, 1H), 3.10 (dddd, J = 16.4, 12.0, 4.8, 4.7 Hz, 2H), 3.01 – 2.86 (m, 2H), 2.75 (dddd, J = 13.0, 9.4, 4.2, 4.2 Hz, 2H), 2.53 – 2.34 (m, 4H).

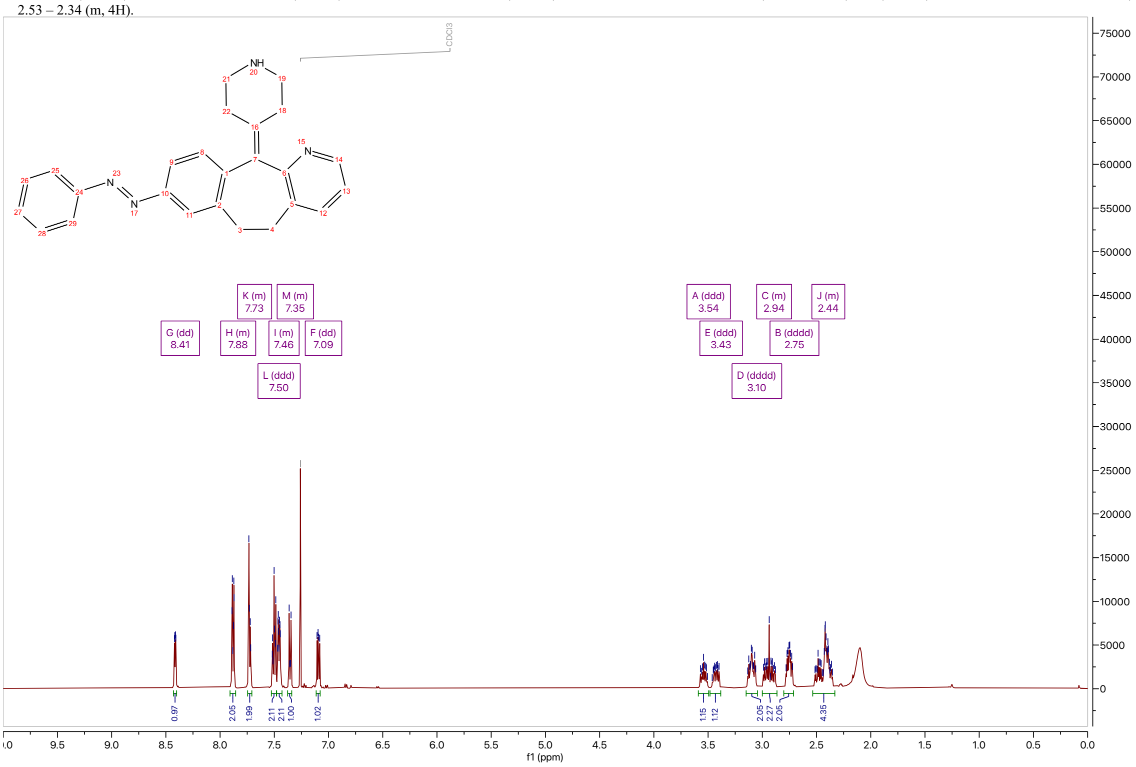

Figure S61. ^{1}H NMR spectrum of **11b**.

^{13}C NMR (126 MHz, $CDCl_3$) δ 157.4, 152.9, 152.0, 146.8, 142.6, 139.1, 138.9, 137.6, 133.8, 133.6, 131.0, 130.4, 129.2, 123.3, 122.9, 122.3, 121.0, 48.1, 48.1, 32.6, 32.3, 32.1, 31.8.

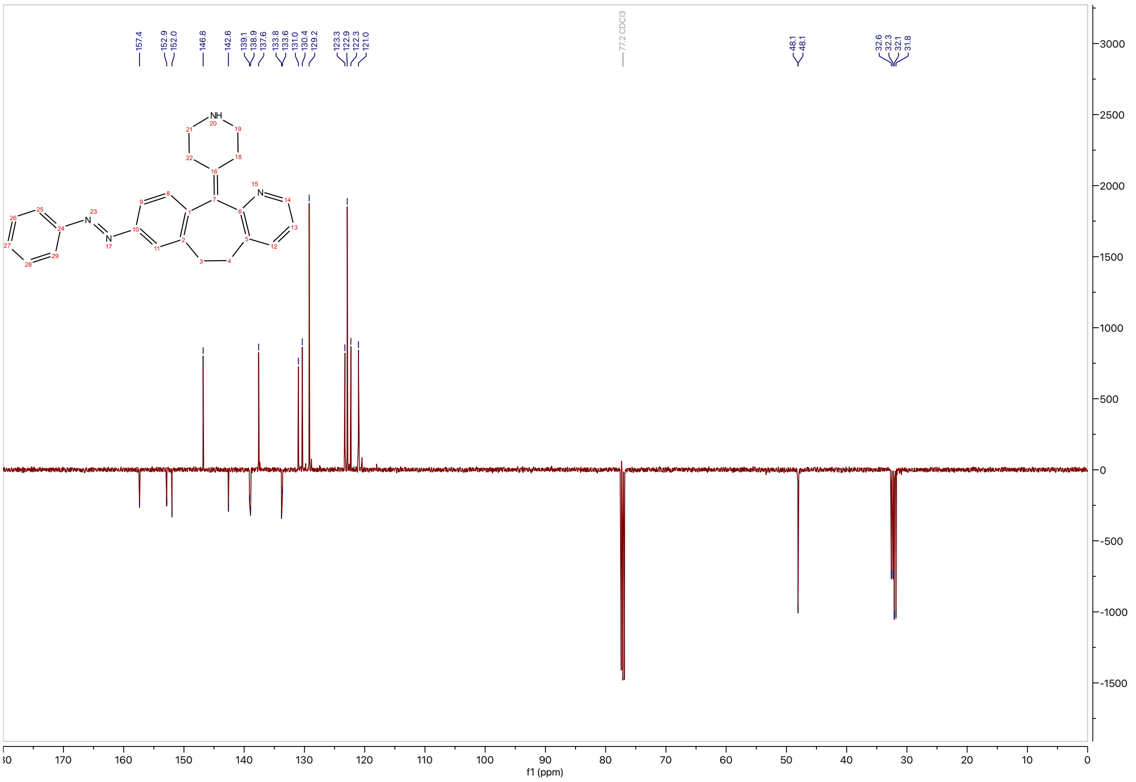

Figure S62. ^{13}C NMR spectrum of **11b**.

Acquired by	: Admin
Date Acquired	: 7/18/2024 12:23:24 PM
Sample Name	: DAVE01-036-8
Sample ID	:
Tray#	: 1
Vial#	: 12
Injection Volume	: 2
Data File	: C:\LabSolutions\Data\2024\2024-wk29\DAVE01-036-8.lcd
Background File	: azoblanco MeOH 18072024.lcd
Method File	: Method SCAN ACID standard azo.lcm
Report Format	: DefaultLCMS.lcr
Tuning File	: C:\LabSolutions\Tuning File\Tuning-ESI-pos-neg01072015.lct
Processed by	: Admin
Modified Date	: 7/18/2024 12:41:01 PM

Chromatogram

DAVE01-036-8 C:\LabSolutions\Data\2024\2024-wk29\DAVE01-036-8.lcd

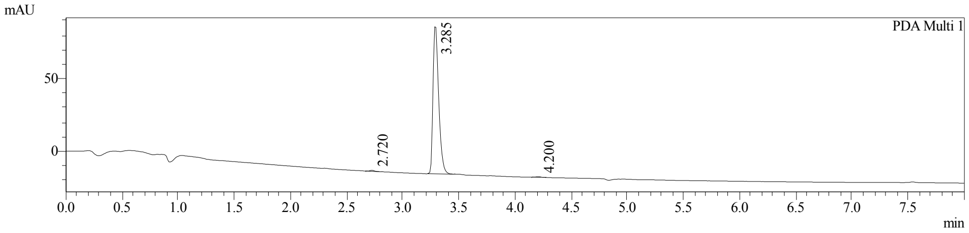

1 PDA Multi 1 / 254nm 4nm

PeakTable

PDA Ch1 254nm 4nm

Peak#	Ret. Time	Area	Height	Name	Area %
1	2.720	1987	746		0.556
2	3.285	354535	101287		99.168
3	4.200	988	344		0.276
Total		357509	102377		100.000

MS Chromatogram

DAVE01-036-8 C:\LabSolutions\Data\2024\2024-wk29\DAVE01-036-8.lcd

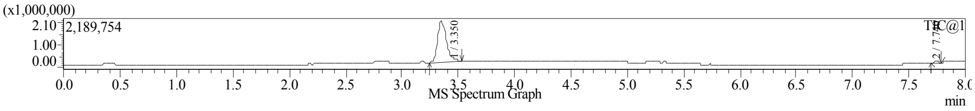

MS Spectrum Graph

#:1 Ret.Time:Averaged 3.340-3.360(Scan#:335-337)

BG Mode:Calc 3.240<->3.530(325<->354)

Mass Peaks:13 Base Peak:381.20(1210367) Polarity:Pos Segment1 - Event1

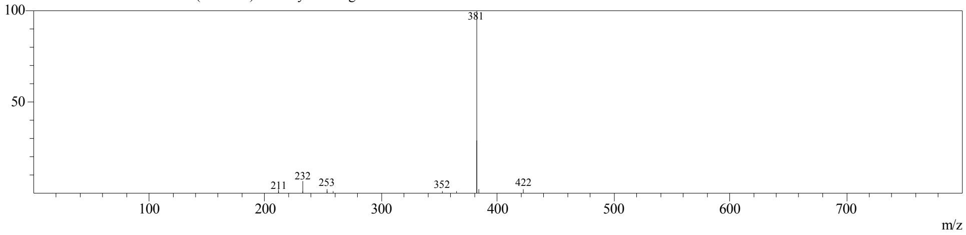

MS Spectrum Table

#:1 Ret.Time:

BG Mode:Calc 3.240<->3.530(325<->354)

Mass Peaks:13 Base Peak:381.20(1210367) Polarity:Pos Segment1 - Event1

#	m/z	Abs.Inten.	Rel.Inten.	Charge	Polarity	Monoisotopic	#	m/z	Abs.Inten.	Rel.Inten.	Charge	Polarity	Monoisotopic
1	211.50	13785	1.14				8	352.10	19875	1.64			
2	211.70	25412	2.10				9	364.25	16683	1.38			
3	232.15	77389	6.39				10	381.20	1210367	100.00			
4	232.65	16976	1.40				11	382.20	347472	28.71			
5	252.55	14106	1.17				12	383.20	32486	2.68			
6	253.00	34061	2.81				13	422.20	33605	2.78			
7	259.10	15883	1.31										

Figure S63. LC-MS chromatogram of **11b**.

Generic Display Report

Analysis Info

Analysis Name	D:\Data\ServiceMS\Hans\2024-wk40\DAVE DAVE01-036_10-1-2024_12-09-09_ServiceMs Hystar.d	Acquisition Date	10/1/2024 12:09:57 PM
Method	ServiceMs Hystar.m	Operator	Demo User
Sample Name	DAVE DAVE01-036	Instrument	impact II
Comment			

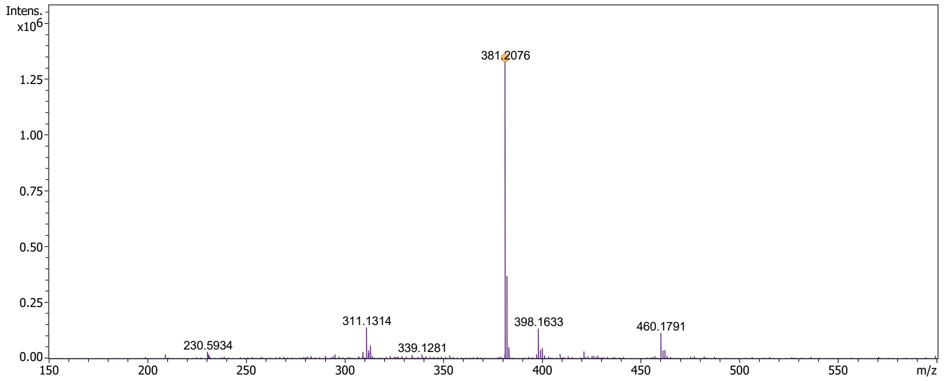

Meas. m/z	#	Ion Formula	m/z	err [ppm]	mSigma	#mSigma	Score	rdb	e⁻ Conf	N-Rule
381.2076	1	C25H25N4	381.2074	-0.5	5.9	1	100.00	16.0	even	ok

Bruker Compass DataAnalysis 6.0 printed: 10/1/2024 12:17:06 PM by: demo Page 1 of 1

Figure S64. HRMS spectrum of **11b**.

^{1}H NMR (500 MHz, $CDCl_3$) δ 8.43 (dd, J = 4.8, 1.7 Hz, 1H), 7.79 – 7.73 (m, 1H), 7.48 (dd, J = 7.7, 1.7 Hz, 1H), 7.40 – 7.34 (m, 1H), 7.15 (dd, J = 7.7, 4.8 Hz, 1H), 4.14 (q, J = 7.1 Hz, 2H), 3.89 – 3.75 (m, 2H), 3.55 – 3.32 (m, 2H), 3.17 (dddd, J = 18.5, 13.3, 9.3, 3.9 Hz, 2H), 2.88 (dddd, J = 14.0, 10.5, 8.4, 5.4 Hz, 2H), 2.52 (ddd, J = 14.1, 9.4, 4.7 Hz, 1H), 2.42 (ddd, J = 14.0, 9.3, 4.7 Hz, 1H), 2.35 – 2.24 (m, 2H), 1.25 (t, J = 7.1 Hz, 3H).

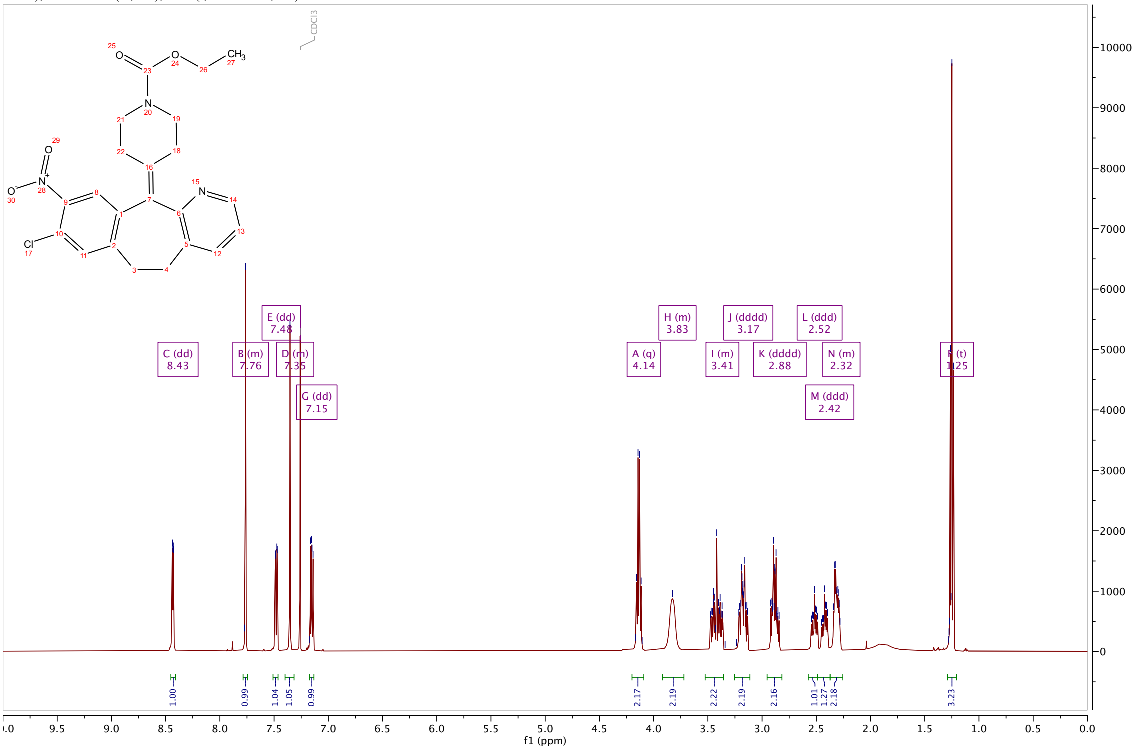

Figure S65. ^{1}H NMR spectrum of **25**.

^{13}C NMR (126 MHz, $CDCl_3$) δ 156.1, 155.6, 147.2, 145.6, 144.5, 140.0, 138.9, 137.8, 133.1, 132.5, 132.2, 126.6, 125.8, 122.9, 61.6, 44.7, 31.8, 31.0, 30.9, 30.7, 14.8.

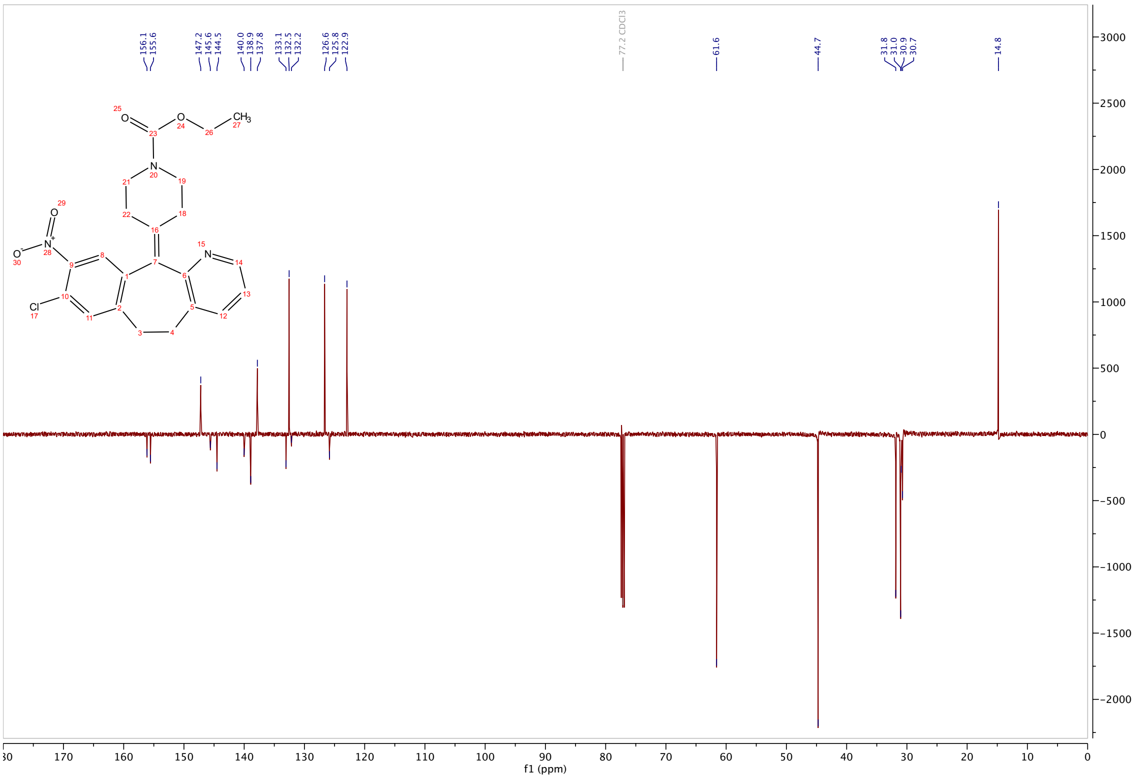

Figure S66. ^{13}C NMR spectrum of **25**.

Acquired by : Admin
Date Acquired : 6/13/2024 9:38:25 AM
Sample Name : DAVE01-018-4-major
Sample ID :
Tray# : 1
Vial# : 1
Injection Volume : 1
Data File : C:\LabSolutions\Data\2024\2024-wk24\DAVE01-018-4-major.lcd
Background File : blanco 13062024.lcd
Method File : Method SCAN ACID standard.lcm
Report Format : DefaultLCMS.lcr
Tuning File : C:\LabSolutions\Tuning File\Tuning-ESI-pos-neg01072015.lct
Processed by : Admin
Modified Date : 6/13/2024 10:02:55 AM

Chromatogram
DAVE01-018-4-major C:\LabSolutions\Data\2024\2024-wk24\DAVE01-018-4-major.lcd

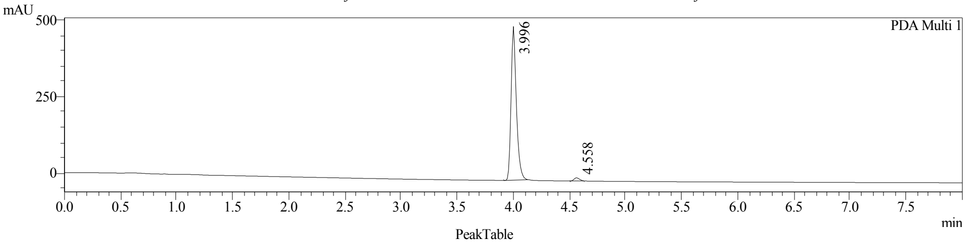

PeakTable

PDA Ch1 254nm 4nm

Peak#	Ret. Time	Area	Height	Name	Area %
1	3.996	1686084	504634		98.082
2	4.558	32975	10195		1.918
Total		1719059	514829		100.000

MS Chromatogram
DAVE01-018-4-major C:\LabSolutions\Data\2024\2024-wk24\DAVE01-018-4-major.lcd

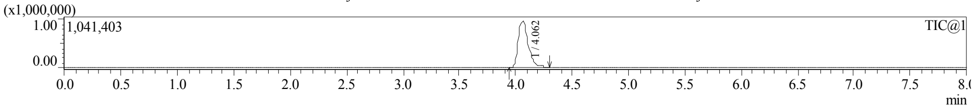

MS Spectrum Graph

#:1 Ret.Time:Averaged 4.050-4.070(Scan#:406-408)
BG Mode:Calc 3.940<->4.300(395<->431)
Mass Peaks:7 Base Peak:428.10(590100) Polarity:Pos Segment1 - Event1

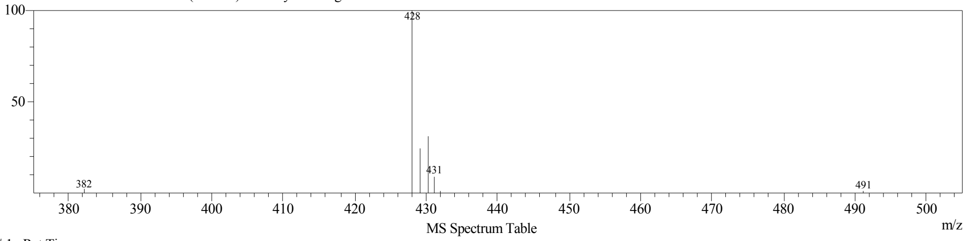

MS Spectrum Table

#:1 Ret.Time:
BG Mode:Calc 3.940<->4.300(395<->431)
Mass Peaks:7 Base Peak:428.10(590100) Polarity:Pos Segment1 - Event1

#	m/z	Abs.Inten.	Rel.Inten.	Charge	Polarity	Monoisotopic	#	m/z	Abs.Inten.	Rel.Inten.	Charge	Polarity	Monoisotopic
1	382.15	11355	1.92				5	431.15	56020	9.49			
2	428.10	590100	100.00				6	432.10	8722	1.48			
3	429.20	147130	24.93				7	491.20	8155	1.38			
4	430.15	184873	31.33										

#:2 Ret.Time:
BG Mode:None
Mass Peaks:5 Base Peak:473.15(16291) Polarity:Pos Segment1 - Event1

#	m/z	Abs.Inten.	Rel.Inten.	Charge	Polarity	Monoisotopic	#	m/z	Abs.Inten.	Rel.Inten.	Charge	Polarity	Monoisotopic
1	151.00	2340	14.36				2	473.15	16291	100.00			

Figure S67. LC-MS chromatogram of **25**.

^{1}H NMR (500 MHz, $CDCl_3$) δ 8.43 (dd, J = 4.7, 1.7 Hz, 1H), 7.47 (dΔ, J = 7.5 Hz, 1H), 7.33 (d, J = 8.3 Hz, 1H), 7.27 (d, J = 8.3 Hz, 1H), 7.16 (dd, J = 7.7, 4.8 Hz, 1H), 4.14 (q, J = 7.1 Hz, 2H), 3.89 – 3.76 (m, 2H), 3.40 – 3.28 (m, 2H), 3.18 (dddd, J = 13.1, 9.3, 6.8, 3.9 Hz, 2H), 2.96 (ddd, J = 13.8, 13.1, 5.5 Hz, 1H), 2.76 (ddd, J = 15.1, 7.6, 4.4 Hz, 1H), 2.53 (ddd, J = 14.1, 9.4, 4.6 Hz, 1H), 2.42 – 2.23 (m, 3H), 1.25 (t, J = 7.1 Hz, 4H).

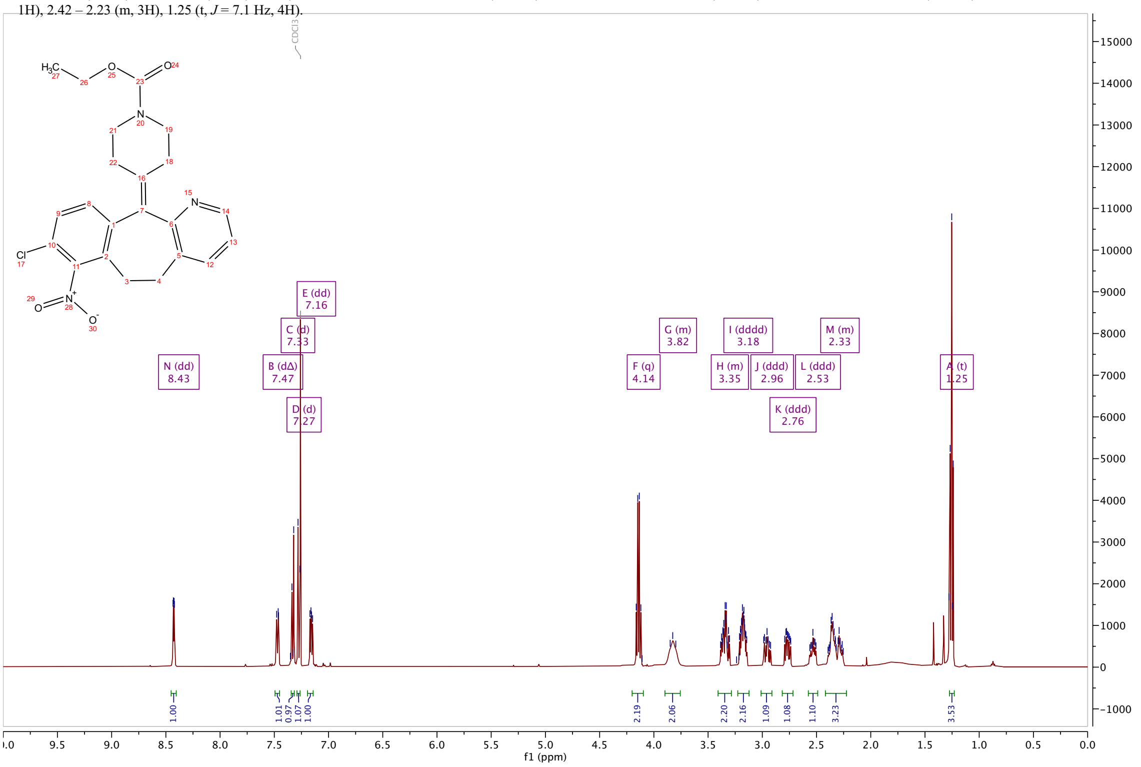

Figure S68. ^{1}H NMR spectrum of **28**.

^{13}C NMR (126 MHz, $CDCl_3$) δ 155.6, 155.4, 149.8, 147.0, 140.6, 138.4, 132.9, 131.6, 131.1, 128.3, 123.7, 122.9, 61.6, 44.8, 31.1, 30.8, 27.0, 14.8.

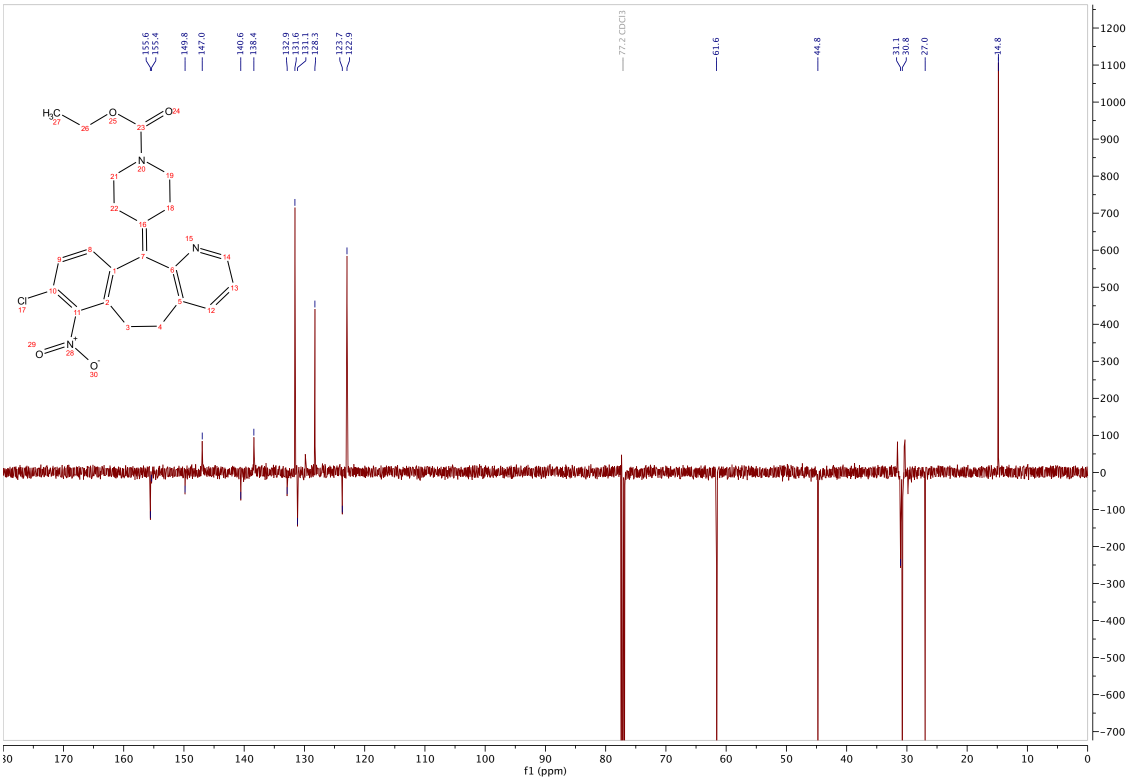

Figure S69. ^{13}C NMR spectrum of **28**.

Acquired by	: Admin
Date Acquired	: 6/13/2024 9:47:03 AM
Sample Name	: DAVE01-018-4-minor
Sample ID	:
Tray#	: 1
Vial#	: 2
Injection Volume	: 1
Data File	: C:\LabSolutions\Data\2024\2024-wk24\DAVE01-018-4-minor.lcd
Background File	: blanco 13062024.lcd
Method File	: Method SCAN ACID standard.lcm
Report Format	: DefaultLCMS.lcr
Tuning File	: C:\LabSolutions\Tuning File\Tuning-ESI-pos-neg01072015.lct
Processed by	: Admin
Modified Date	: 6/13/2024 10:04:48 AM

Chromatogram
DAVE01-018-4-minor C:\LabSolutions\Data\2024\2024-wk24\DAVE01-018-4-minor.lcd

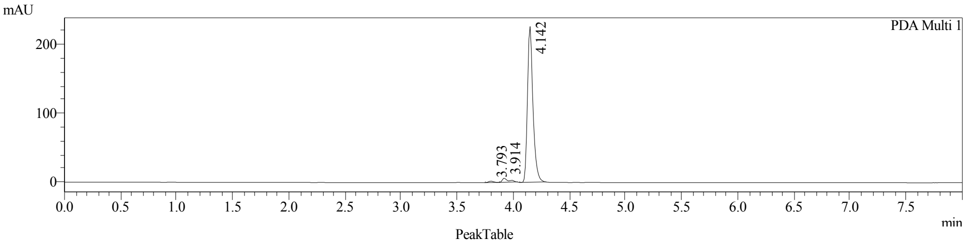

PeakTable

PDA Ch1 254nm 4nm

Peak#	Ret. Time	Area	Height	Name	Area %
1	3.793	6931	2141		0.856
2	3.914	23632	5854		2.917
3	4.142	779536	226265		96.227
Total		810098	234261		100.000

MS Chromatogram
DAVE01-018-4-minor C:\LabSolutions\Data\2024\2024-wk24\DAVE01-018-4-minor.lcd

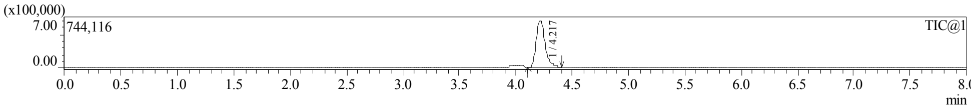

MS Spectrum Graph

#:1 Ret.Time:Averaged 4.210-4.230(Scan#:422-424)
BG Mode:Calc 4.110<->4.410(412<->442)
Mass Peaks:6 Base Peak:428.15(447266) Polarity:Pos Segment1 - Event1

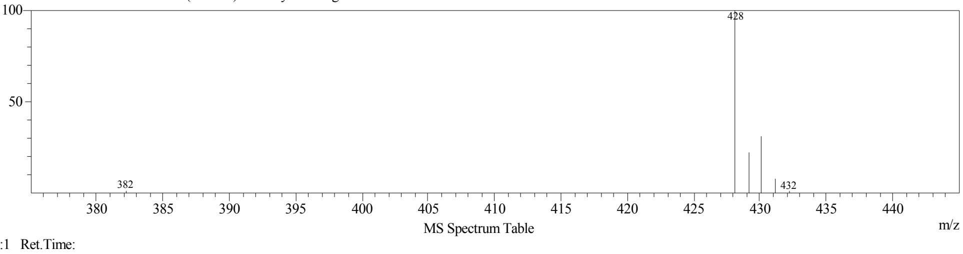

MS Spectrum Table

#:1 Ret.Time:
BG Mode:Calc 4.110<->4.410(412<->442)
Mass Peaks:6 Base Peak:428.15(447266) Polarity:Pos Segment1 - Event1

#	m/z	Abs.Inten.	Rel.Inten.	Charge	Polarity	Monoisotopic	#	m/z	Abs.Inten.	Rel.Inten.	Charge	Polarity	Monoisotopic
1	382.15	7778	1.74				4	430.15	140528	31.42			
2	428.15	447266	100.00				5	431.10	37906	8.48			
3	429.15	98336	21.99				6	432.15	5130	1.15			

#:2 Ret.Time:
BG Mode:None
Mass Peaks:8 Base Peak:428.10(6798) Polarity:Pos Segment1 - Event1

#	m/z	Abs.Inten.	Rel.Inten.	Charge	Polarity	Monoisotopic	#	m/z	Abs.Inten.	Rel.Inten.	Charge	Polarity	Monoisotopic
1	151.00	1926	28.33				3	428.10	6798	100.00			
2	223.90	1214	17.86				4	429.00	2850	41.92			

Figure S70. LC-MS chromatogram of **28**.

^{1}H NMR (500 MHz, MeOD) δ 8.36 (dd, J = 4.9, 1.6 Hz, 1H), 7.75 – 7.71 (m, 1H), 7.69 (dd, J = 7.8, 1.6 Hz, 1H), 7.53 – 7.49 (m, 1H), 7.29 (dd, J = 7.7, 4.9 Hz, 1H), 3.54 – 3.40 (m, 2H), 3.06 – 2.88 (m, 4H), 2.69 (dddd, J = 33.3, 12.7, 9.4, 3.7 Hz, 2H), 2.47 – 2.28 (m, 3H), 2.24 – 2.15 (m, 1H).

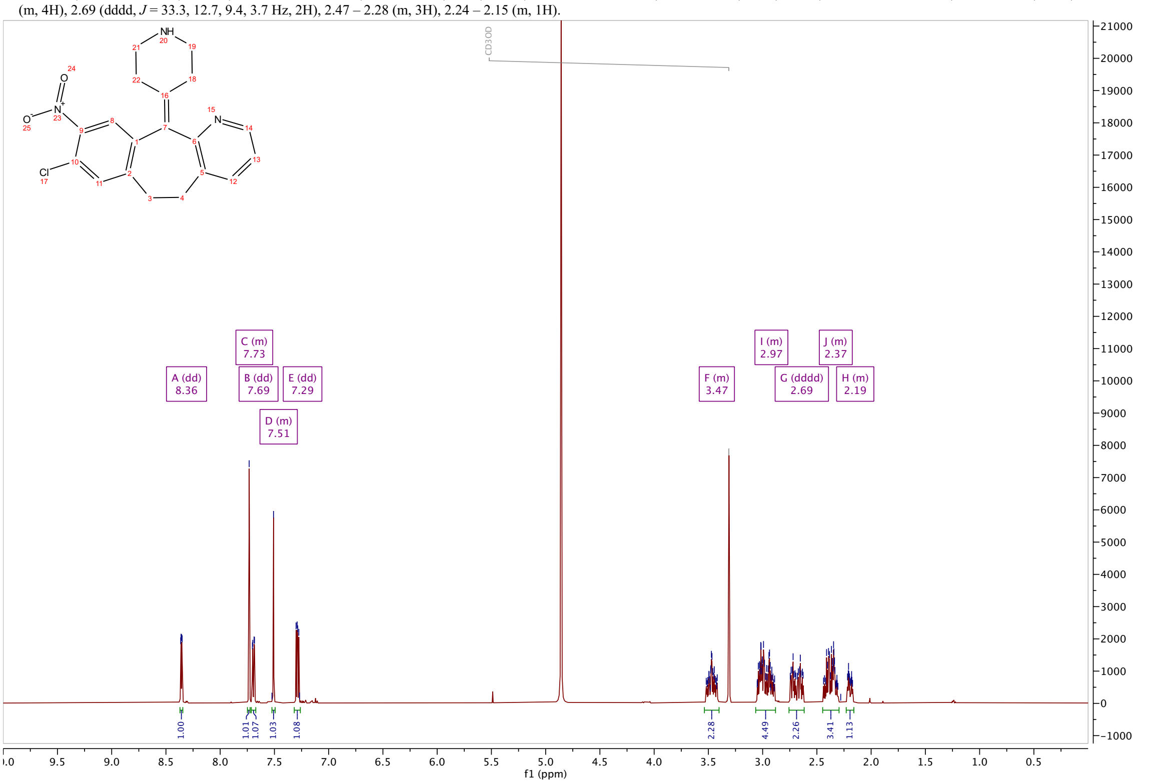

Figure S71. ^{1}H NMR spectrum of **26**.

^{13}C NMR (126 MHz, MeOD) δ 157.9, 147.5, 146.9, 146.1, 139.8, 139.6, 135.7, 133.9, 131.5, 127.3, 126.0, 124.5, 48.2, 48.0, 32.9, 32.8, 32.7, 31.3.

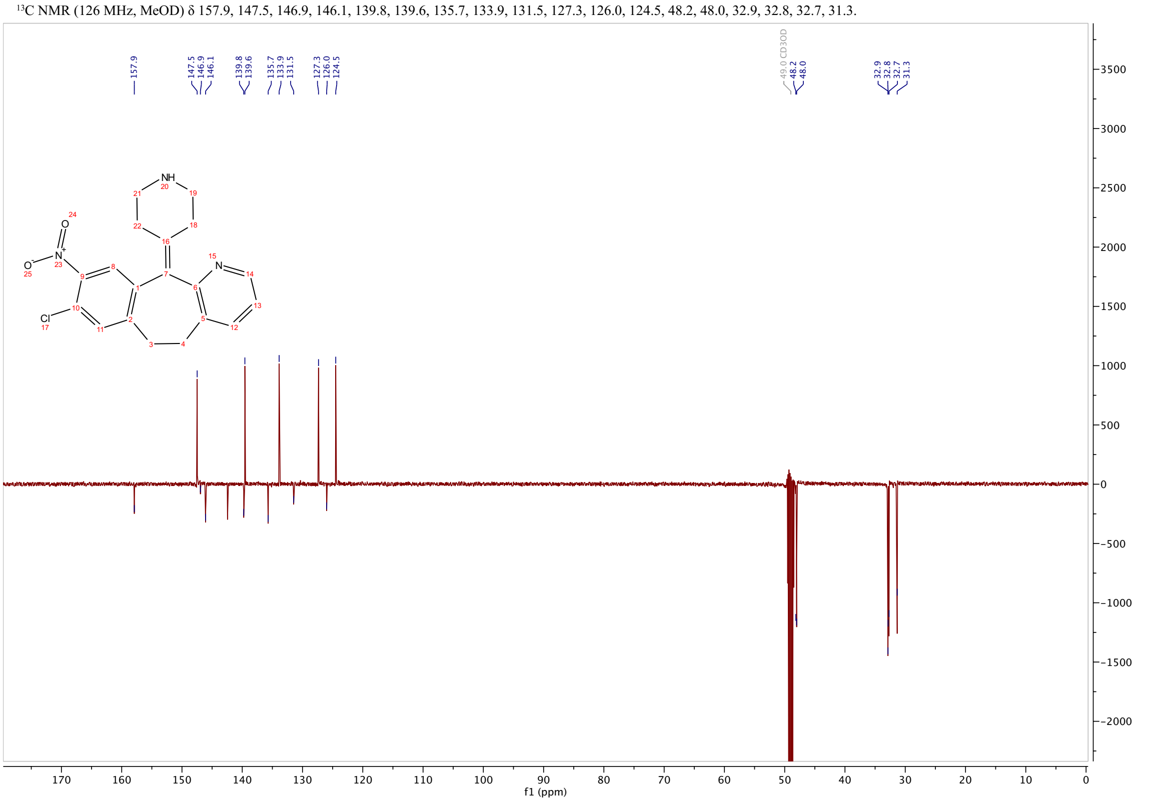

Figure S72. ^{13}C NMR spectrum of **26**.

Acquired by	: Admin
Date Acquired	: 8/29/2024 1:01:43 PM
Sample Name	: DAVE01-050-1
Sample ID	:
Tray#	: 1
Vial#	: 8
Injection Volume	: 1
Data File	: C:\LabSolutions\Data\2024\2024-wk35\DAVE01-050-1.lcd
Background File	: blanco 29082024.lcd
Method File	: Method SCAN ACID standard.lcm
Report Format	: DefaultLCMS.lcr
Tuning File	: C:\LabSolutions\Tuning File\Tuning-ESI-pos-neg01072015.lct
Processed by	: Admin
Modified Date	: 8/29/2024 1:38:48 PM

Chromatogram
DAVE01-050-1 C:\LabSolutions\Data\2024\2024-wk35\DAVE01-050-1.lcd

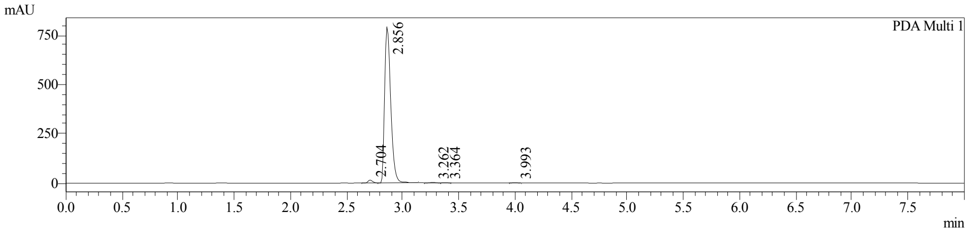

1 PDA Multi 1 / 254nm 4nm

PeakTable

PDA Ch1 254nm 4nm

Peak#	Ret. Time	Area	Height	Name	Area %
1	2.704	40823	15133		1.325
2	2.856	3024689	790130		98.202
3	3.262	6439	2070		0.209
4	3.364	4100	1480		0.133
5	3.993	4002	1171		0.130
Total		3080053	809983		100.000

MS Chromatogram
DAVE01-050-1 C:\LabSolutions\Data\2024\2024-wk35\DAVE01-050-1.lcd

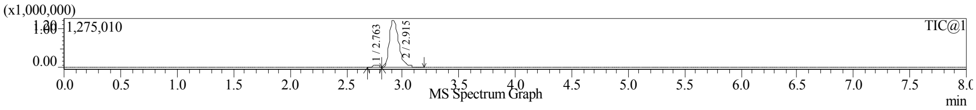

MS Spectrum Graph

#:1 Ret.Time:Averaged 2.900-2.920(Scan#:291-293)
BG Mode:Calc 2.820<->3.190(283<->320)
Mass Peaks:11 Base Peak:356.10(604520) Polarity:Pos Segment1 - Event1

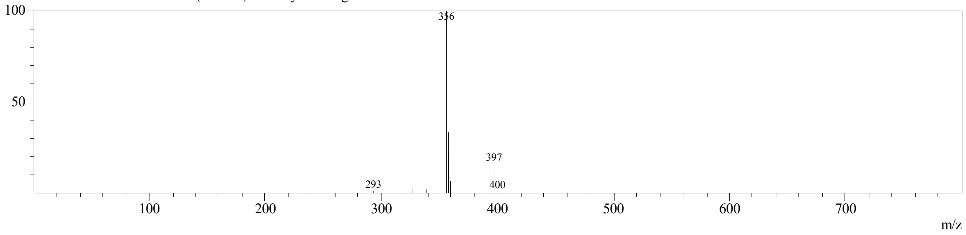

Figure S73. LC-MS chromatogram of **26**.

^{1}H NMR (500 MHz, $CDCl_3$) δ 8.43 (dd, *J* = 4.8, 1.7 Hz, 1H), 7.78 – 7.74 (m, 1H), 7.47 (dd, *J* = 7.7, 1.7 Hz, 1H), 7.37 – 7.33 (m, 1H), 7.14 (dd, *J* = 7.7, 4.8 Hz, 1H), 3.83 – 3.74 (m, 2H), 3.50 – 3.34 (m, 2H), 3.10 (dddd, *J* = 13.2, 13.2, 10.4, 3.6 Hz, 2H), 2.94 – 2.82 (m, 2H), 2.45 (dddd, *J* = 44.3, 14.0, 9.4, 4.7 Hz, 2H), 2.30 (dddd, *J* = 14.5, 5.2 Hz, 2H), 1.45 (s, 9H).

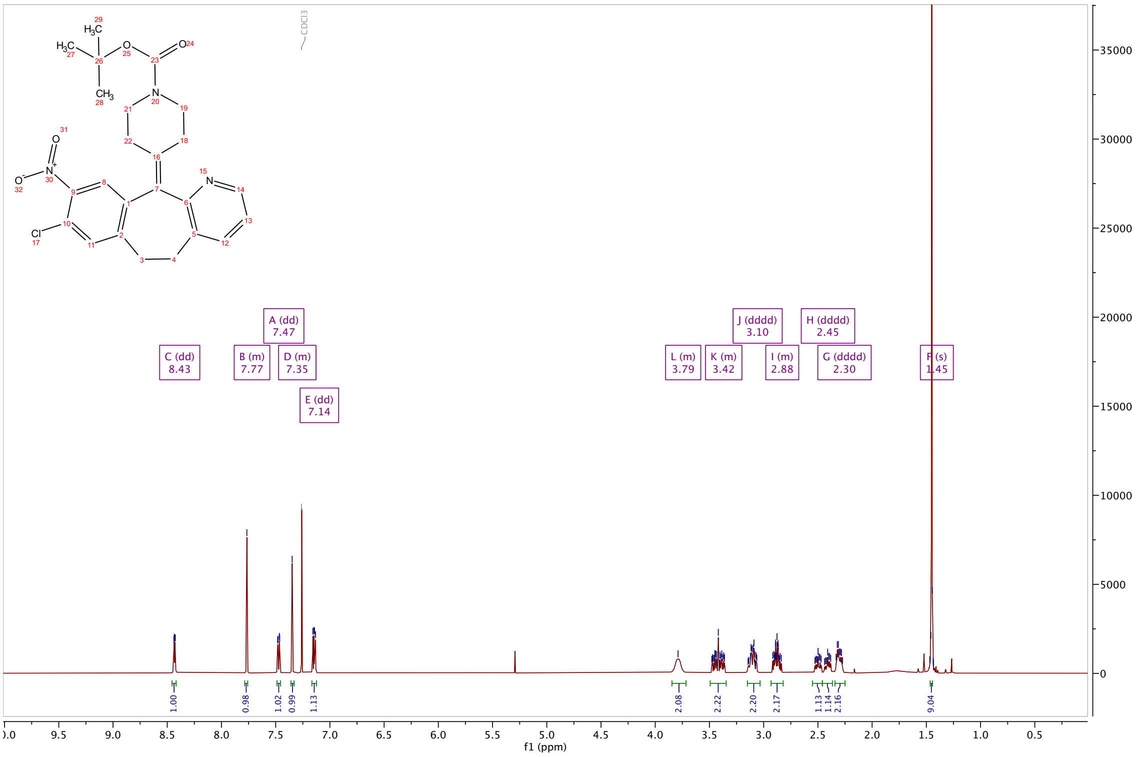

Figure S74. ^{1}H NMR spectrum of **27**.

^{13}C NMR (126 MHz, $CDCl_3$) δ 156.3, 154.8, 147.3, 145.6, 144.5, 140.3, 139.0, 137.7, 133.0, 132.5, 132.0, 126.7, 125.8, 122.9, 79.9, 45.9, 31.9, 31.0, 31.0, 30.8, 28.5.

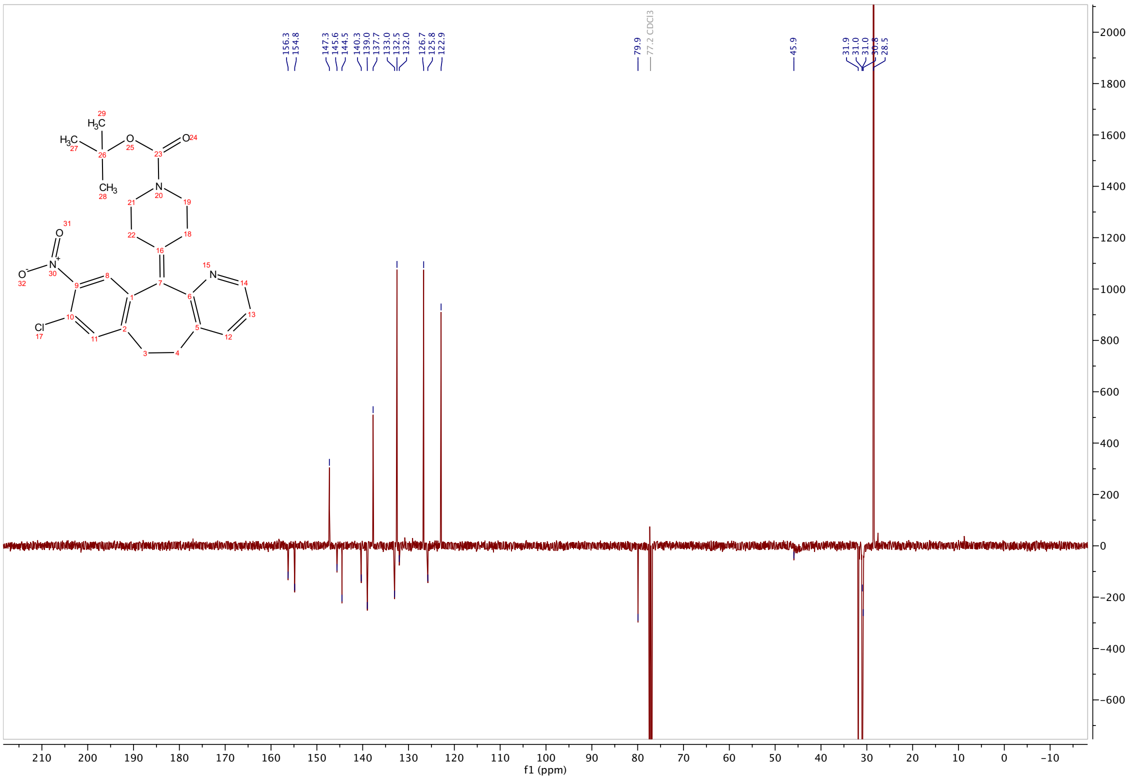

Figure S75. ^{13}C NMR spectrum of **27**.

Acquired by : Admin
Date Acquired : 8/30/2024 4:28:23 PM
Sample Name : DAVE01-058-1
Sample ID :
Tray# : 1
Vial# : 12
Injection Volume : 1
Data File : C:\LabSolutions\Data\2024\2024-wk35\DAVE01-058-1.lcd
Background File : blanco 30082024.lcd
Method File : Method SCAN ACID standard.lcm
Report Format : DefaultLCMS.lcr
Tuning File : C:\LabSolutions\Tuning File\Tuning-ESI-pos-neg01072015.lct
Processed by : Admin
Modified Date : 8/30/2024 4:40:40 PM

Chromatogram
DAVE01-058-1 C:\LabSolutions\Data\2024\2024-wk35\DAVE01-058-1.lcd

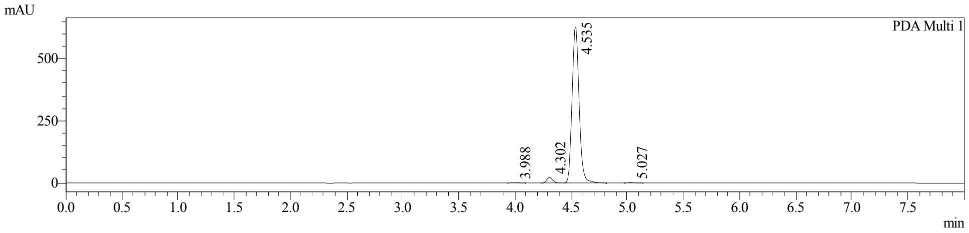

1 PDA Multi 1 / 254nm 4nm

PeakTable

PDA Ch1 254nm 4nm

Peak#	Ret. Time	Area	Height	Name	Area %
1	3.988	7276	1562		0.261
2	4.302	82577	22158		2.958
3	4.535	2694354	626768		96.530
4	5.027	6989	1724		0.250
Total		2791196	652211		100.000

MS Chromatogram
DAVE01-058-1 C:\LabSolutions\Data\2024\2024-wk35\DAVE01-058-1.lcd

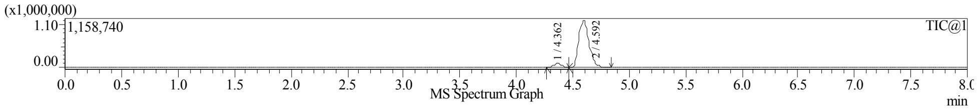

MS Spectrum Graph

#:1 Ret.Time:Averaged 4.350-4.370(Scan#:436-438)
BG Mode:Calc 4.270<->4.470(428<->448)
Mass Peaks:12 Base Peak:411.15(51451) Polarity:Pos Segment1 - Event1

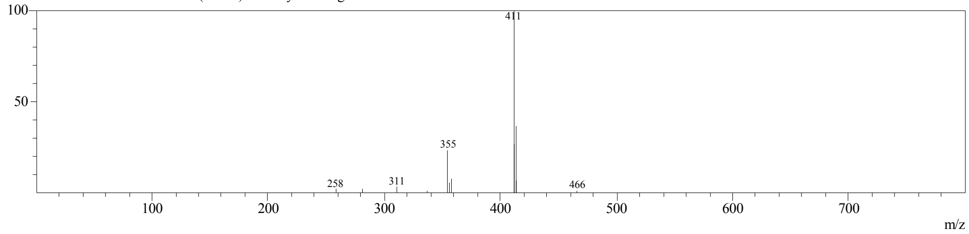

Figure S76. LC-MS chromatogram of **27**.

^{1}H NMR (500 MHz, $CDCl_3$) δ 8.38 (dd, J = 4.8, 1.7 Hz, 1H), 7.42 (dd, J = 7.7, 1.7 Hz, 1H), 7.08 (dd, J = 7.7, 4.8 Hz, 1H), 7.05 (s, 1H), 6.61 (s, 1H), 3.91 (s, 2H), 3.77 (s, 2H), 3.34 – 3.23 (m, 2H), 3.05 (dddd, J = 13.4, 9.8, 9.7, 4.0 Hz, 2H), 2.81 (ddd, J = 16.8, 9.5, 4.7 Hz, 1H), 2.73 – 2.64 (m, 1H), 2.48 – 2.30 (m, 3H), 2.26 (ddd, J = 14.2, 4.6 Hz, 1H), 1.45 (s, 9H).

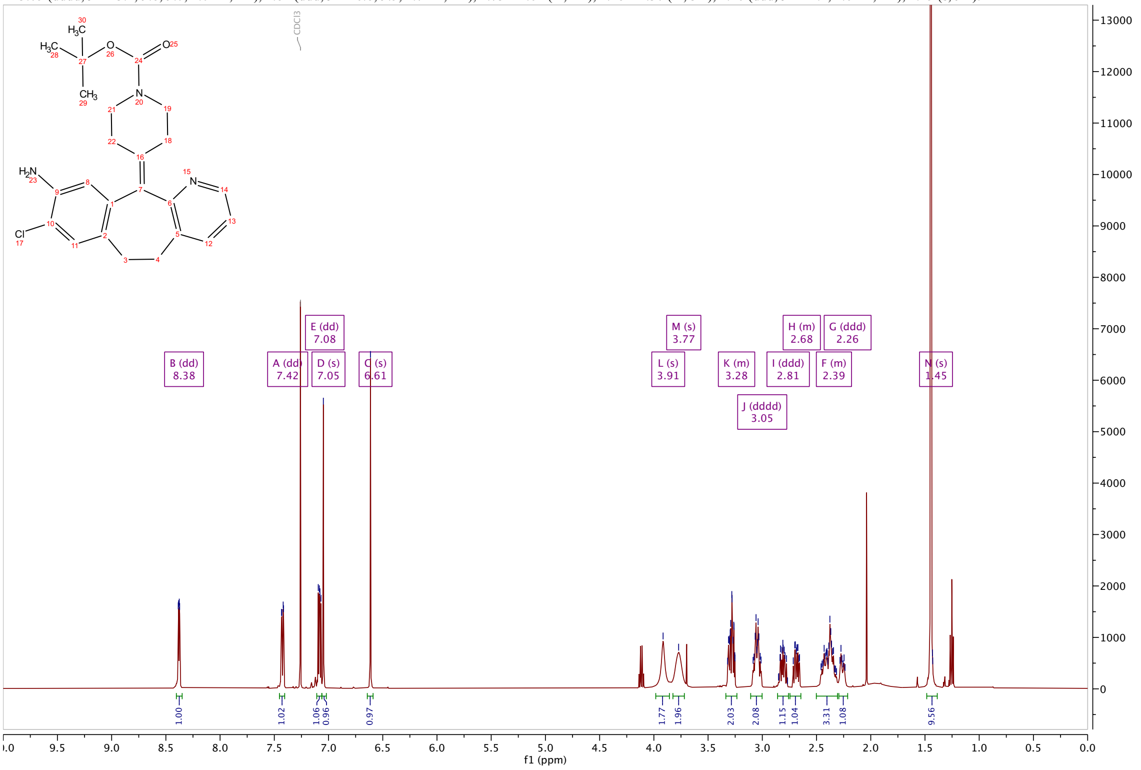

Figure S77. ^{1}H NMR spectrum of **33**.

^{13}C NMR (126 MHz, $CDCl_3$) δ 157.2, 154.9, 146.5, 140.9, 139.0, 137.9, 137.5, 134.3, 133.8, 129.6, 128.6, 122.3, 118.3, 116.4, 79.7, 45.3, 32.1, 30.9, 30.8, 30.7, 28.6.

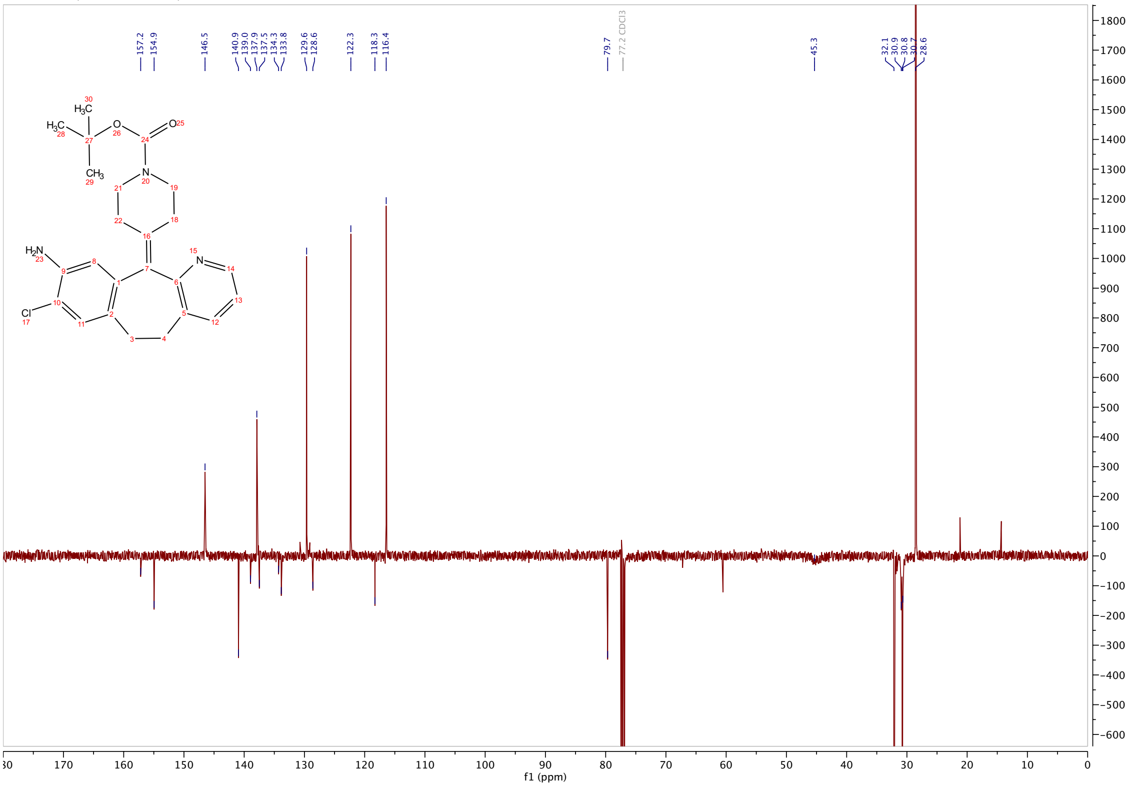

Figure S78. ^{13}C NMR spectrum of **33**.

Acquired by	: Admin
Date Acquired	: 9/3/2024 2:33:36 PM
Sample Name	: DAVE01-061-1
Sample ID	:
Tray#	: 1
Vial#	: 7
Injection Volume	: 1
Data File	: C:\LabSolutions\Data\2024\2024-wk36\DAVE01-061-1.lcd
Background File	: blanco 03092024.lcd
Method File	: Method SCAN ACID standard.lcm
Report Format	: DefaultLCMS.lcr
Tuning File	: C:\LabSolutions\Tuning File\Tuning-ESI-pos-neg01072015.lct
Processed by	: Admin
Modified Date	: 9/3/2024 2:48:28 PM

Chromatogram
DAVE01-061-1 C:\LabSolutions\Data\2024\2024-wk36\DAVE01-061-1.lcd

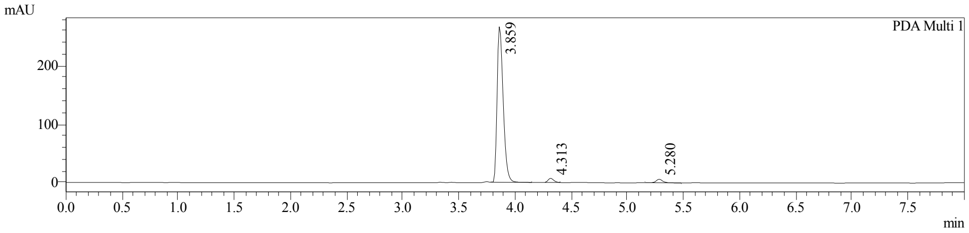

1 PDA Multi 1 / 254nm 4nm

PeakTable

PDA Ch1 254nm 4nm

Peak#	Ret. Time	Area	Height	Name	Area %
1	3.859	1016397	266170		95.203
2	4.313	25580	7285		2.396
3	5.280	25630	6094		2.401
Total		1067606	279549		100.000

MS Chromatogram
DAVE01-061-1 C:\LabSolutions\Data\2024\2024-wk36\DAVE01-061-1.lcd

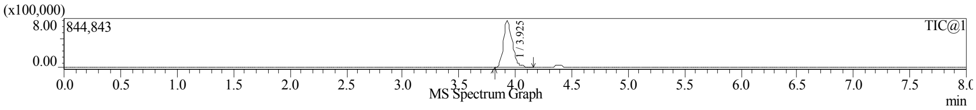

MS Spectrum Graph

#:1 Ret.Time:Averaged 3.910-3.930(Scan#:392-394)
BG Mode:Calc 3.810<->4.160(382<->417)
Mass Peaks:17 Base Peak:426.20(370072) Polarity:Pos Segment1 - Event1

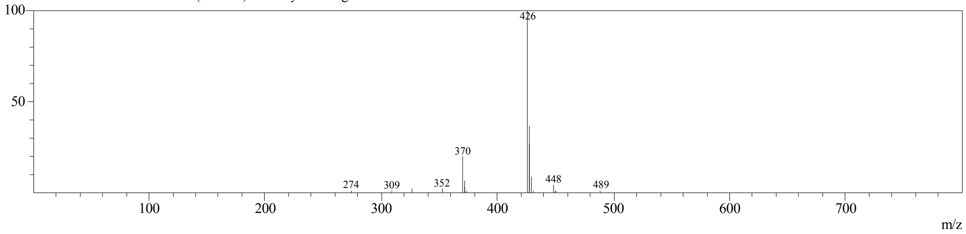

Figure S79. LC-MS chromatogram of **33**.

^{1}H NMR (500 MHz, $CDCl_3$) δ 8.43 (dd, *J* = 4.9, 1.7 Hz, 1H), 7.95 – 7.89 (m, 2H), 7.63 (m, 1H), 7.56 – 7.43 (m, 4H), 7.38 (m, 1H), 7.12 (dd, *J* = 7.7, 4.8 Hz, 1H), 3.78 (m, 2H), 3.51 – 3.35 (m, 2H), 3.12 (ddd, *J* = 13.0, 9.2, 4.0 Hz, 2H), 2.94 – 2.84 (m, 2H), 2.53 (ddd, *J* = 14.0, 9.3, 4.6 Hz, 1H), 2.44 – 2.28 (m, 3H), 1.45 (s, 9H).

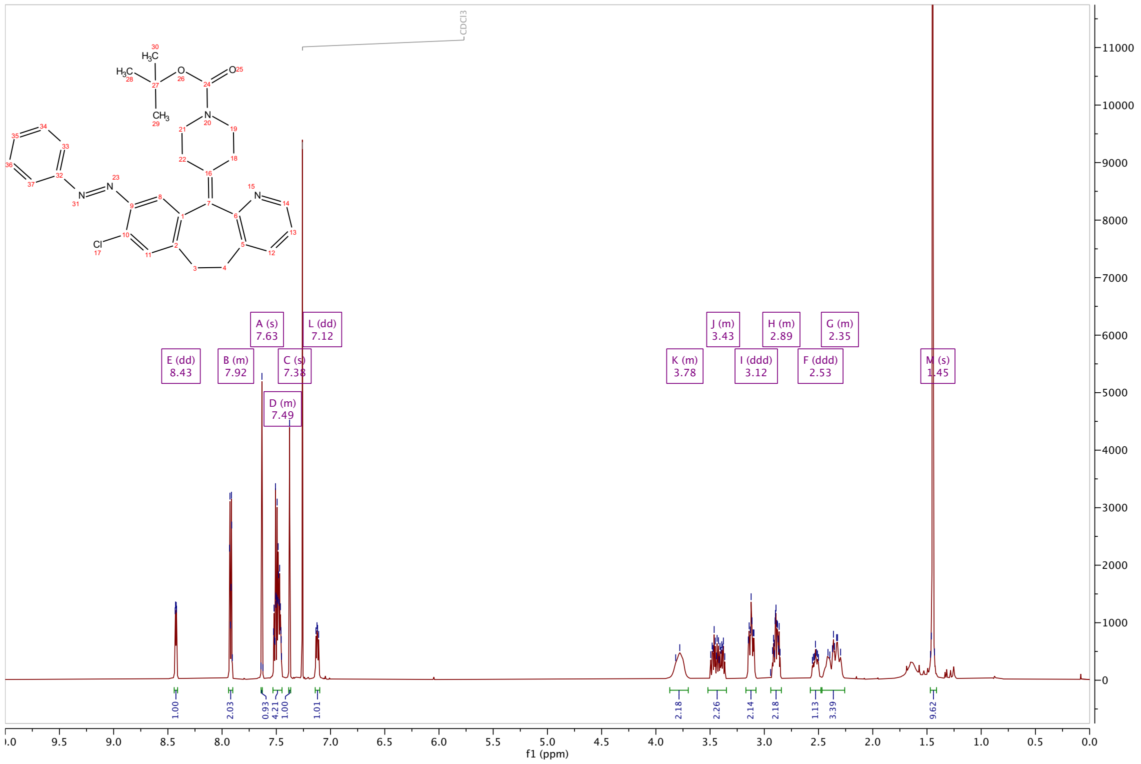

Figure S80. ^{1}H NMR spectrum of **34**.

^{13}C NMR (126 MHz, $CDCl_3$) δ 154.8, 152.7, 146.7, 146.7, 142.2, 138.5, 137.5, 134.1, 133.3, 131.4, 130.9, 129.1, 123.3, 122.4, 118.2, 79.6, 45.2, 31.6, 31.5, 30.9, 30.7, 28.4.

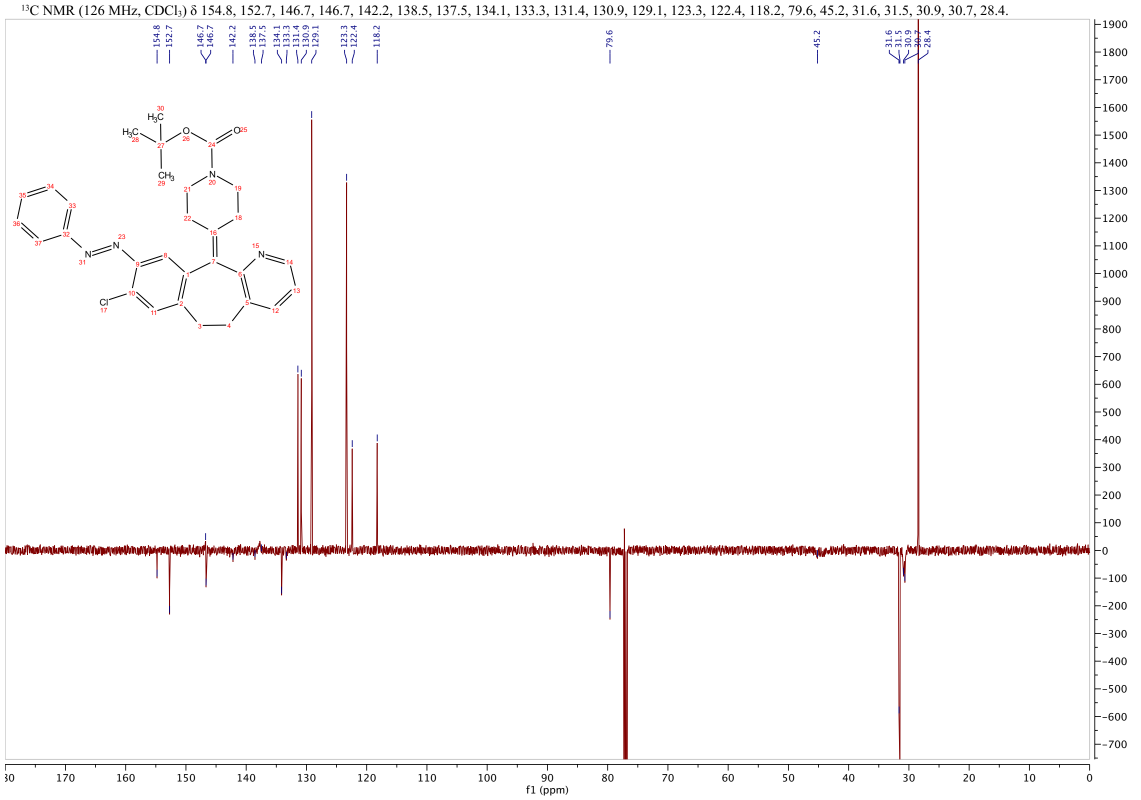

Figure S81. ^{13}C NMR spectrum of **34**.

Acquired by	: Admin
Date Acquired	: 9/16/2024 2:42:24 PM
Sample Name	: DAVE01-063-1
Sample ID	:
Tray#	: 1
Vial#	: 11
Injection Volume	: 2
Data File	: C:\LabSolutions\Data\2024\2024-wk38\DAVE01-063-1.lcd
Background File	: azoblanco 16092024.lcd
Method File	: Method SCAN ACID standard azo.lcm
Report Format	: DefaultLCMS.lcr
Tuning File	: C:\LabSolutions\Tuning File\Tuning-ESI-pos-neg01072015.lct
Processed by	: Admin
Modified Date	: 9/16/2024 3:34:49 PM

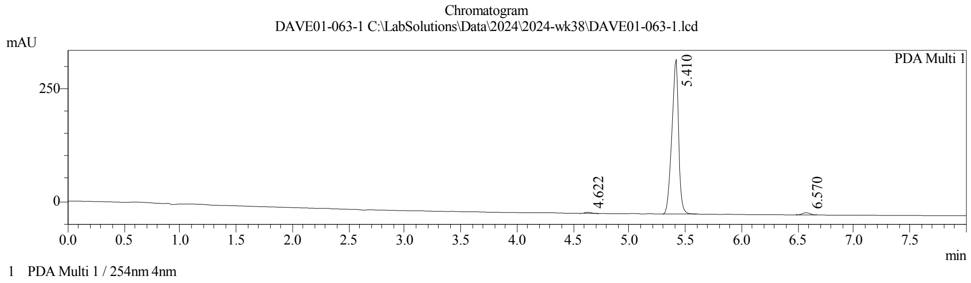

PeakTable

PDA Ch1 254nm 4nm

Peak#	Ret. Time	Area	Height	Name	Area %
1	4.622	9536	2612		0.655
2	5.410	1422749	342648		97.733
3	6.570	23468	4808		1.612
Total		1455753	350068		100.000

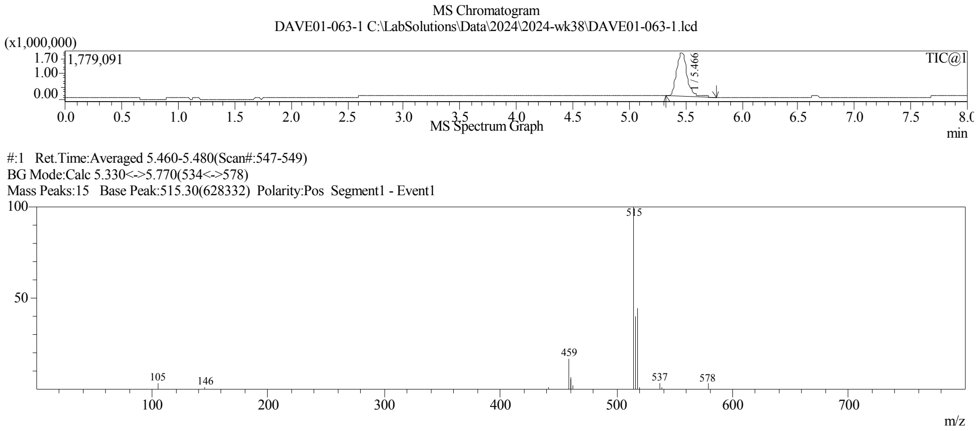

Figure S82. LC-MS chromatogram of **34**.

^{1}H NMR (600 MHz, $CDCl_3$) δ 8.41 (dd, *J* = 4.8, 1.7 Hz, 1H), 7.94 – 7.89 (m, 2H), 7.64 – 7.61 (m, 1H), 7.54 – 7.46 (m, 3H), 7.44 (dd, *J* = 7.7, 1.7 Hz, 1H), 7.39 – 7.35 (m, 1H), 7.09 (dd, *J* = 7.7, 4.7 Hz, 1H), 3.52 – 3.45 (m, 1H), 3.45 – 3.37 (m, 1H), 3.10 (dddd, *J* = 17.3, 11.8, 4.3, 4.3 Hz, 2H), 2.92 – 2.84 (m, 2H), 2.81 – 2.71 (m, 2H), 2.49 (ddd, *J* = 13.9, 9.6, 4.4 Hz, 1H), 2.43 – 2.34 (m, 3H).

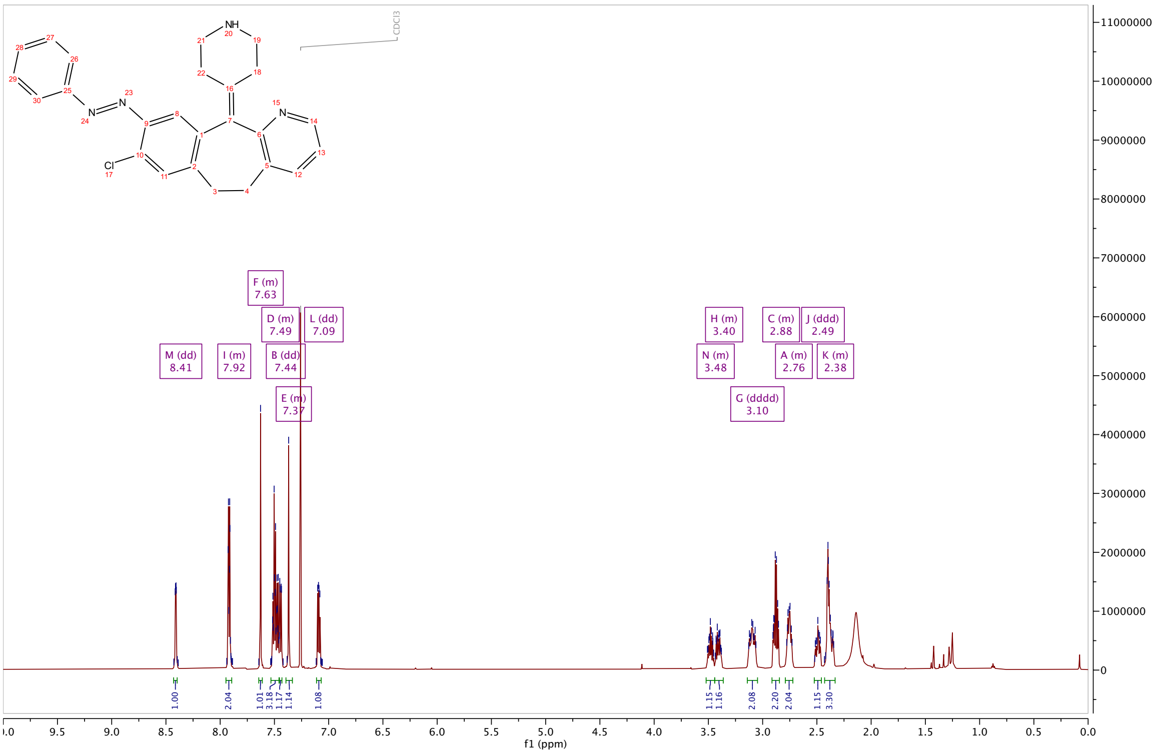

Figure S83. ^{1}H NMR spectrum of **11a**.

^{13}C NMR (151 MHz, $CDCl_3$) δ 157.2, 152.8, 147.0, 146.7, 142.5, 139.4, 138.8, 137.6, 134.1, 133.4, 132.6, 131.5, 131.0, 129.2, 123.4, 122.3, 118.4, 48.0, 47.9, 32.4, 32.2, 31.8, 31.6.

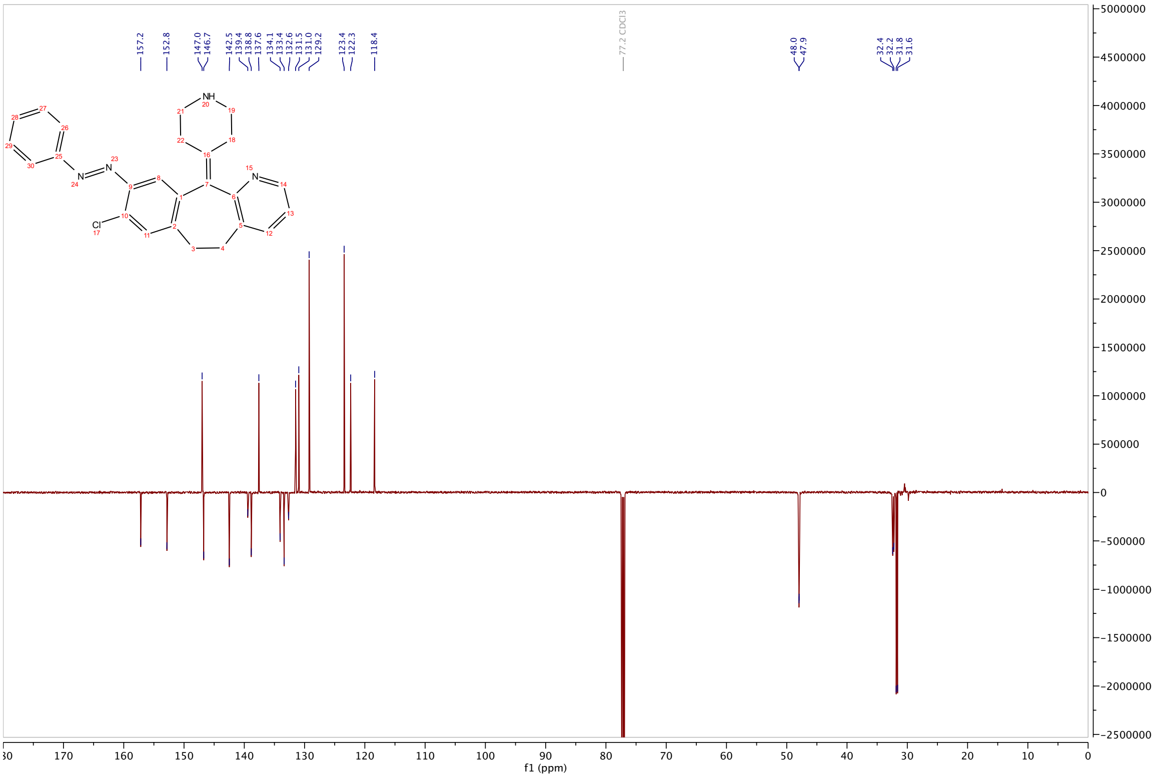

Figure S84. ^{13}C NMR spectrum of **11a**.

Acquired by	: Admin
Date Acquired	: 9/27/2024 10:18:58 AM
Sample Name	: DAVE01-079-2
Sample ID	:
Tray#	: 1
Vial#	: 5
Injection Volume	: 1
Data File	: C:\LabSolutions\Data\2024\2024-wk39\DAVE01-079-2.lcd
Background File	: azoblanco 27092024.lcd
Method File	: Method SCAN ACID standard azo.lcm
Report Format	: DefaultLCMS.lcr
Tuning File	: C:\LabSolutions\Tuning File\Tuning-ESI-pos-neg01072015.lct
Processed by	: Admin
Modified Date	: 9/27/2024 11:20:43 AM

Chromatogram
DAVE01-079-2 C:\LabSolutions\Data\2024\2024-wk39\DAVE01-079-2.lcd

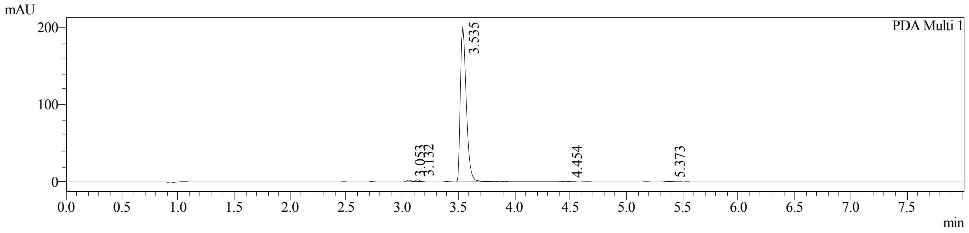

1 PDA Multi 1 / 254nm 4nm

PeakTable

PDA Ch1 254nm 4nm

Peak#	Ret. Time	Area	Height	Name	Area %
1	3.053	5469	2186		0.736
2	3.132	5474	2263		0.736
3	3.535	727745	200923		97.891
4	4.454	2767	921		0.372
5	5.373	1973	645		0.265
Total		743428	206938		100.000

MS Chromatogram
DAVE01-079-2 C:\LabSolutions\Data\2024\2024-wk39\DAVE01-079-2.lcd

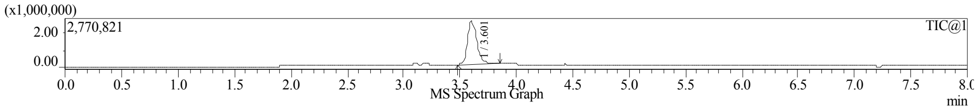

MS Spectrum Graph

#:1 Ret.Time:Averaged 3.590-3.610(Scan#:360-362)
BG Mode:Calc 3.480<->3.850(349<->386)
Mass Peaks:14 Base Peak:415.15(1161233) Polarity:Pos Segment1 - Event1

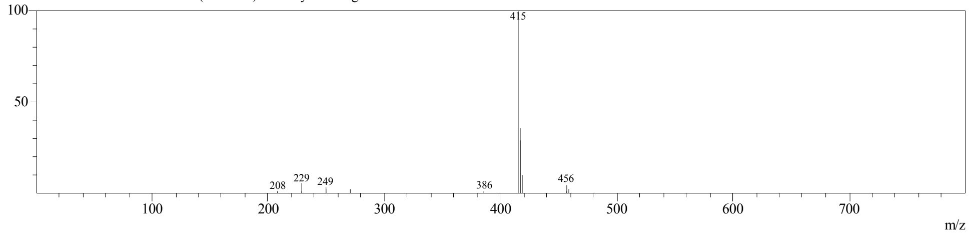

MS Spectrum Table

#:1 Ret.Time:
BG Mode:Calc 3.480<->3.850(349<->386)
Mass Peaks:14 Base Peak:415.15(1161233) Polarity:Pos Segment1 - Event1

#	m/z	Abs.Inten.	Rel.Inten.	Charge	Polarity	Monoisotopic	#	m/z	Abs.Inten.	Rel.Inten.	Charge	Polarity	Monoisotopic
1	208.30	12384	1.07				8	415.15	1161233	100.00			
2	228.25	15864	1.37				9	416.15	333486	28.72			
3	228.65	60677	5.23				10	417.15	410441	35.35			
4	249.15	39692	3.42				11	418.15	114491	9.86			
5	250.05	24304	2.09				12	456.25	57933	4.99			
6	270.00	25727	2.22				13	457.25	19699	1.70			
7	386.05	14639	1.26				14	458.20	21949	1.89			

Figure S85. LC-MS chromatogram of **11a**.

Generic Display Report

Analysis Info

Analysis Name	D:\Data\ServiceMS\Hans\2024-wk40\DAVE DAVE01-079_10-1-2024_12-15-02_ServiceMs Hystar.d	Acquisition Date	10/1/2024 12:15:54 PM
Method	ServiceMs Hystar.m	Operator	Demo User
Sample Name	DAVE DAVE01-079	Instrument	impact II
Comment			

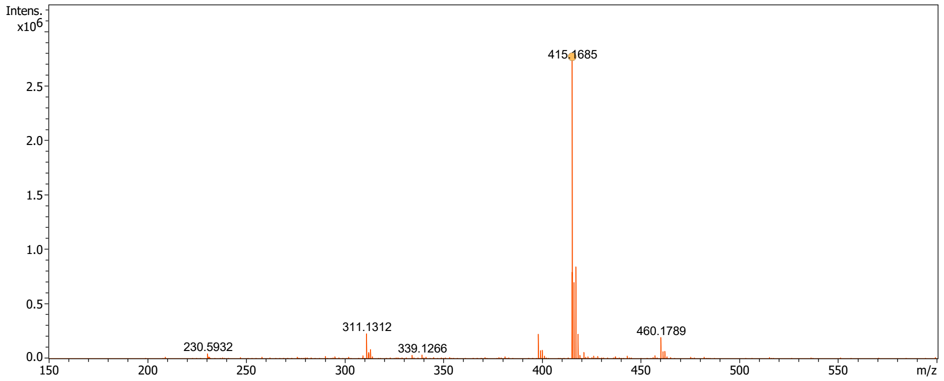

Meas. m/z	#	Ion Formula	m/z	err [ppm]	mSigma	#mSigma	Score	rdb	e⁻ Conf	N-Rule
415.1685	1	C25H24ClN4	415.1684	-0.3	27.2	1	100.00	19.0	even	ok

Bruker Compass DataAnalysis 6.0 printed: 10/1/2024 12:18:22 PM by: demo Page 1 of 1

Figure S86. HRMS spectrum of **11a**.

^{1}H NMR (500 MHz, $CDCl_3$) δ 8.42 (dd, J = 4.7, 1.7 Hz, 1H), 7.44 (dd, J = 7.8, 1.7 Hz, 1H), 7.31 (d, J = 8.3 Hz, 1H), 7.27 (d, J = 8.5 Hz, 1H), 7.12 (dd, J = 7.7, 4.7 Hz, 1H), 3.41 – 3.30 (m, 2H), 3.09 (ddd, J = 16.8, 8.5, 4.8 Hz, 2H), 3.00 – 2.87 (m, 1H), 2.75 (dddd, J = 12.3, 10.8, 9.2, 4.9 Hz, 3H), 2.49 (ddd, J = 14.1, 9.6, 4.5 Hz, 1H), 2.42 – 2.27 (m, 3H).

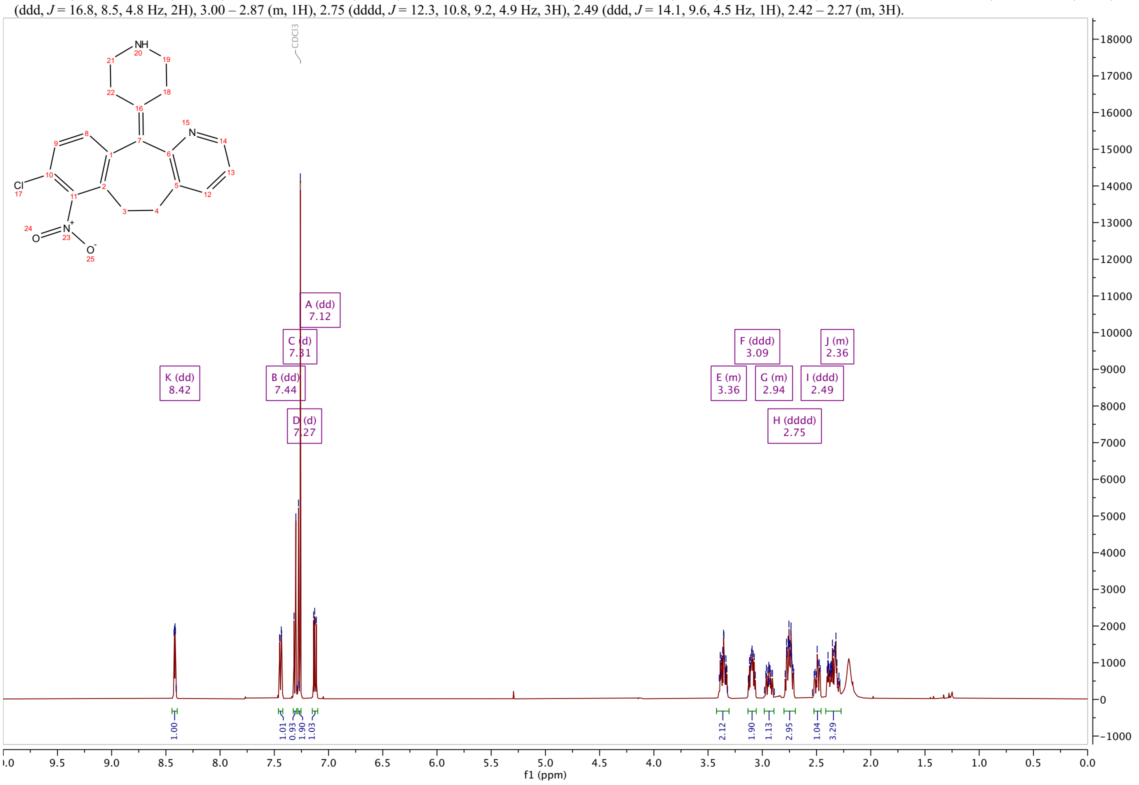

Figure S87. ^{1}H NMR spectrum of **29**.

^{13}C NMR (126 MHz, $CDCl_3$) δ 155.9, 149.8, 147.2, 141.1, 140.8, 138.0, 132.8, 131.7, 131.6, 131.2, 128.1, 123.4, 122.7, 47.9, 47.9, 32.5, 32.2, 30.7, 27.0.

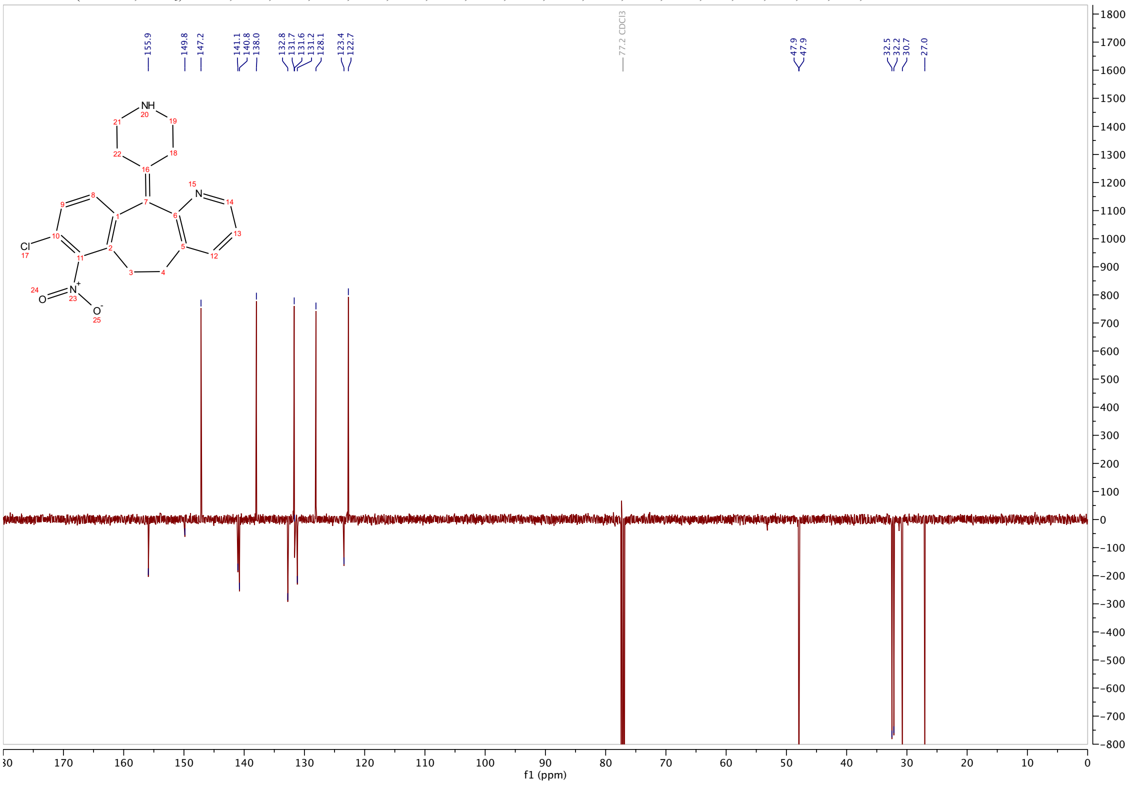

Figure S88. ^{13}C NMR spectrum of **29**.

Acquired by	: Admin
Date Acquired	: 8/29/2024 1:10:21 PM
Sample Name	: DAVE01-052-1
Sample ID	:
Tray#	: 1
Vial#	: 9
Injection Volume	: 1
Data File	: C:\LabSolutions\Data\2024\2024-wk35\DAVE01-052-1.lcd
Background File	: blanco 29082024.lcd
Method File	: Method SCAN ACID standard.lcm
Report Format	: DefaultLCMS.lcr
Tuning File	: C:\LabSolutions\Tuning File\Tuning-ESI-pos-neg01072015.lct
Processed by	: Admin
Modified Date	: 8/29/2024 1:40:04 PM

Chromatogram
DAVE01-052-1 C:\LabSolutions\Data\2024\2024-wk35\DAVE01-052-1.lcd

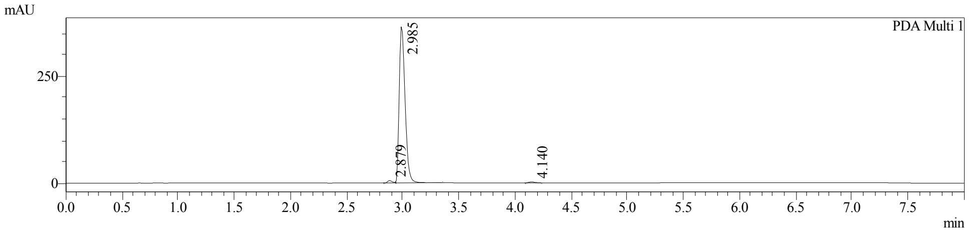

1 PDA Multi 1 / 254nm 4nm

PeakTable

PDA Ch1 254nm 4nm

Peak#	Ret. Time	Area	Height	Name	Area %
1	2.879	15875	5402		1.174
2	2.985	1326903	365647		98.150
3	4.140	9142	2236		0.676
Total		1351920	373285		100.000

MS Chromatogram
DAVE01-052-1 C:\LabSolutions\Data\2024\2024-wk35\DAVE01-052-1.lcd

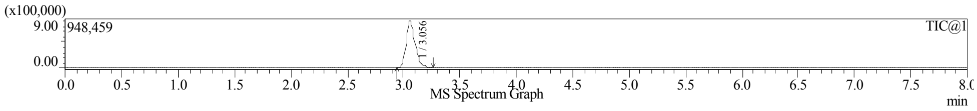

MS Spectrum Graph

#:1 Ret.Time:Averaged 3.050-3.070(Scan#:306-308)
BG Mode:Calc 2.940<->3.260(295<->327)
Mass Peaks:12 Base Peak:356.10(501630) Polarity:Pos Segment1 - Event1

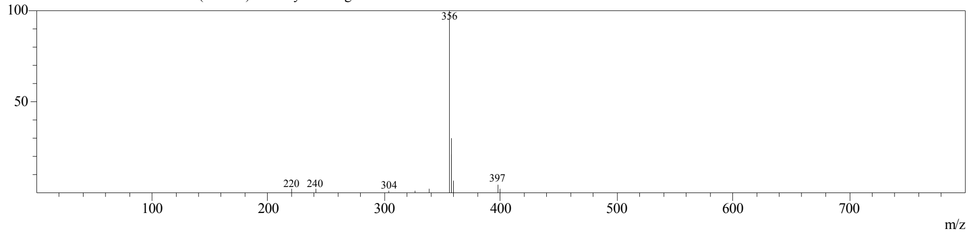

MS Spectrum Table

#:1 Ret.Time:
BG Mode:Calc 2.940<->3.260(295<->327)
Mass Peaks:12 Base Peak:356.10(501630) Polarity:Pos Segment1 - Event1

#	m/z	Abs.Inten.	Rel.Inten.	Charge	Polarity	Monoisotopic	#	m/z	Abs.Inten.	Rel.Inten.	Charge	Polarity	Monoisotopic
1	219.90	9091	1.81				7	357.10	107886	21.51			
2	240.10	9829	1.96				8	358.10	152612	30.42			
3	304.05	5477	1.09				9	359.10	32433	6.47			
4	327.05	5473	1.09				10	397.10	25102	5.00			
5	339.05	9198	1.83				11	398.20	5358	1.07			
6	356.10	501630	100.00				12	399.20	10320	2.06			

Figure S89. LC-MS chromatogram of **29**.

^{1}H NMR (500 MHz, $CDCl_3$) δ 8.42 (dd, J = 4.8, 1.7 Hz, 1H), 7.46 (dd, J = 7.8, 1.7 Hz, 1H), 7.32 (d, J = 8.2 Hz, 1H), 7.27 (d, J = 8.0 Hz, 1H), 7.14 (dd, J = 7.7, 4.8 Hz, 1H), 3.86 – 3.73 (m, 2H), 3.40 – 3.28 (m, 2H), 3.16 – 3.06 (m, 2H), 2.99 – 2.90 (m, 1H), 2.76 (ddd, J = 15.1, 7.6, 4.3 Hz, 1H), 2.51 (ddd, J = 14.2, 9.4, 4.7 Hz, 1H), 2.40 – 2.22 (m, 3H), 1.45 (s, 9H).

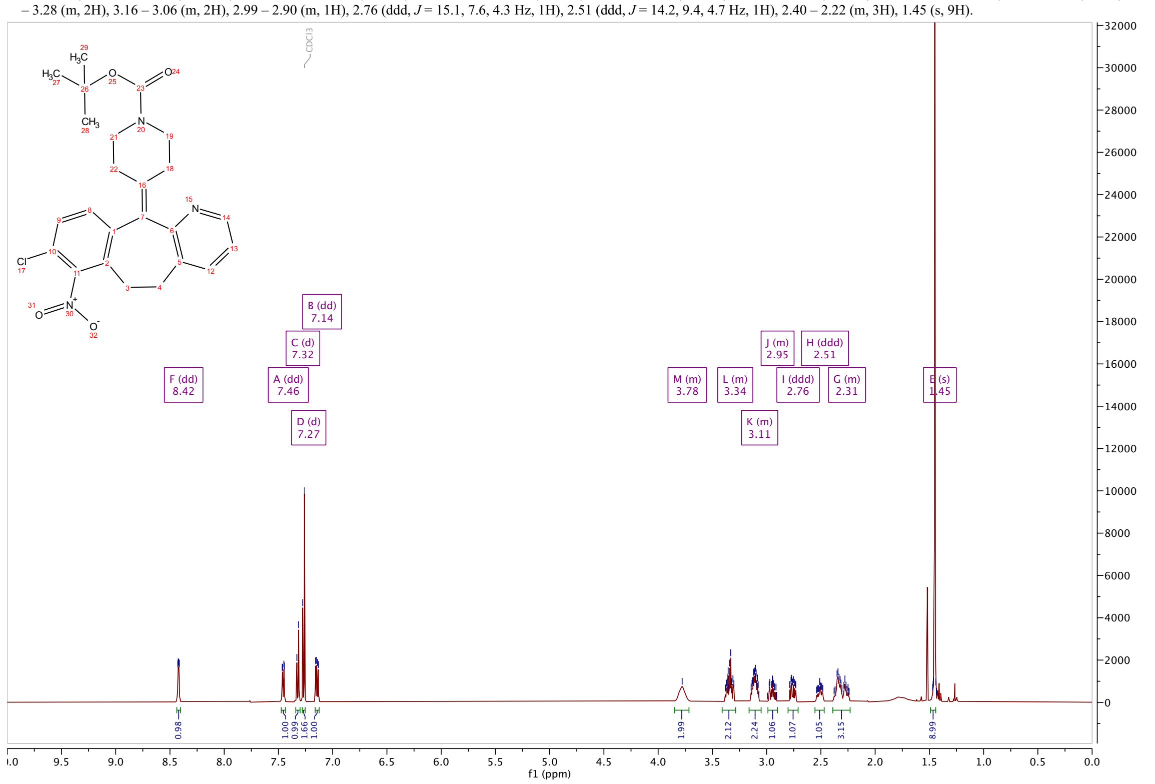

Figure S90. ^{1}H NMR spectrum of **30**.

^{13}C NMR (126 MHz, $CDCl_3$) δ 155.4, 154.7, 149.7, 146.9, 140.5, 140.2, 138.1, 132.7, 132.4, 131.5, 131.0, 128.1, 123.5, 122.7, 79.8, 45.8, 31.0, 30.6, 28.4, 26.9.

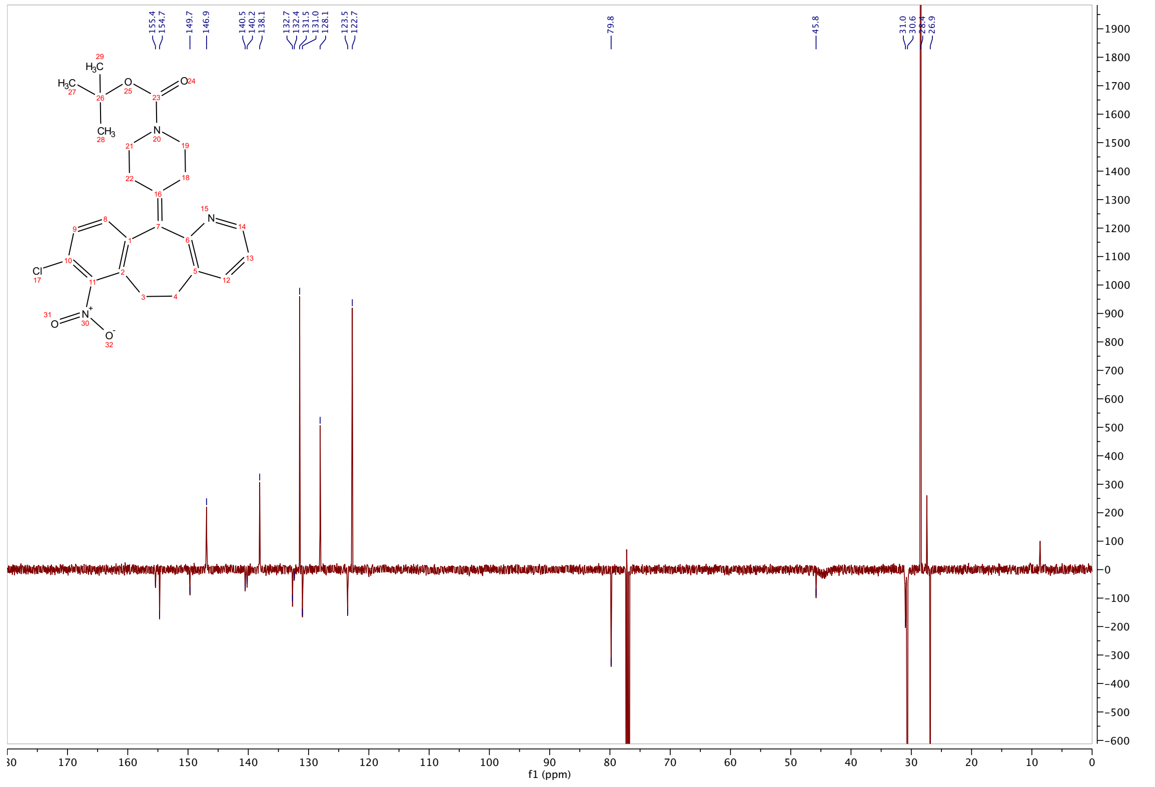

Figure S91. ^{13}C NMR spectrum of **30**.

Acquired by	: Admin
Date Acquired	: 8/30/2024 4:37:00 PM
Sample Name	: DAVE01-059-1
Sample ID	:
Tray#	: 1
Vial#	: 13
Injection Volume	: 1
Data File	: C:\LabSolutions\Data\2024\2024-wk35\DAVE01-059-1.lcd
Background File	: blanco 30082024.lcd
Method File	: Method SCAN ACID standard.lcm
Report Format	: DefaultLCMS.lcr
Tuning File	: C:\LabSolutions\Tuning File\Tuning-ESI-pos-neg01072015.lct
Processed by	: Admin
Modified Date	: 8/30/2024 4:52:55 PM

Chromatogram
DAVE01-059-1 C:\LabSolutions\Data\2024\2024-wk35\DAVE01-059-1.lcd

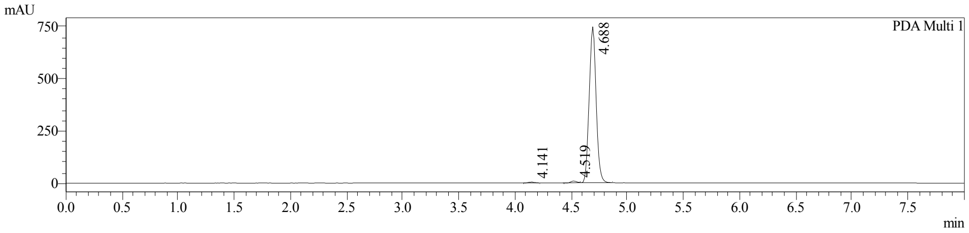

1 PDA Multi 1 / 254nm 4nm

PeakTable

PDA Ch1 254nm 4nm

Peak#	Ret. Time	Area	Height	Name	Area %
1	4.141	15888	4104		0.489
2	4.519	30231	8352		0.931
3	4.688	3200359	747306		98.579
Total		3246478	759762		100.000

MS Chromatogram
DAVE01-059-1 C:\LabSolutions\Data\2024\2024-wk35\DAVE01-059-1.lcd

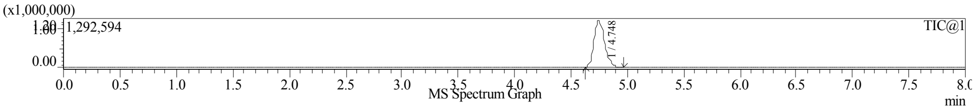

MS Spectrum Graph

#:1 Ret.Time:Averaged 4.740-4.760(Scan#:475-477)
BG Mode:Calc 4.630<->4.970(464<->498)
Mass Peaks:11 Base Peak:456.15(513725) Polarity:Pos Segment1 - Event1

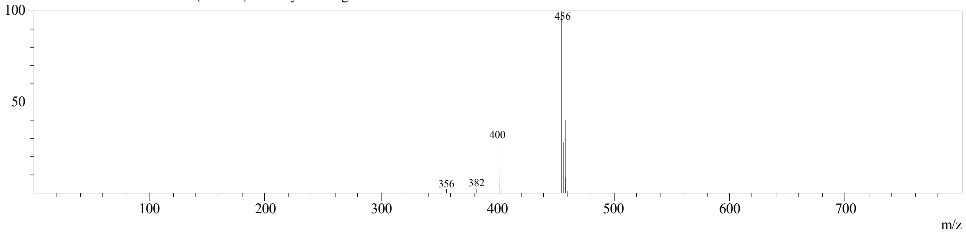

Figure S92. LC-MS chromatogram of **30**.

^{1}H NMR (500 MHz, $CDCl_3$) δ 8.42 (dd, J = 4.9, 1.7 Hz, 1H), 7.53 (dd, J = 7.6, 1.6 Hz, 1H), 7.18 – 7.10 (m, 2H), 6.64 (d, J = 8.3 Hz, 1H), 4.06 (s, 2H), 3.76 (s, 2H), 3.46 (ddd, J = 15.1, 11.3, 4.3 Hz, 1H), 3.15 (ddd, J = 13.1, 8.5, 4.7 Hz, 1H), 3.09 (ddd, J = 8.7, 3.2, 3.2 Hz, 2H), 2.89 (ddd, J = 14.6, 6.2, 4.5 Hz, 1H), 2.63 (ddd, J = 16.0, 11.3, 4.5 Hz, 1H), 2.52 – 2.35 (m, 3H), 2.22 – 2.13 (m, 1H), 1.47 (s, 9H).

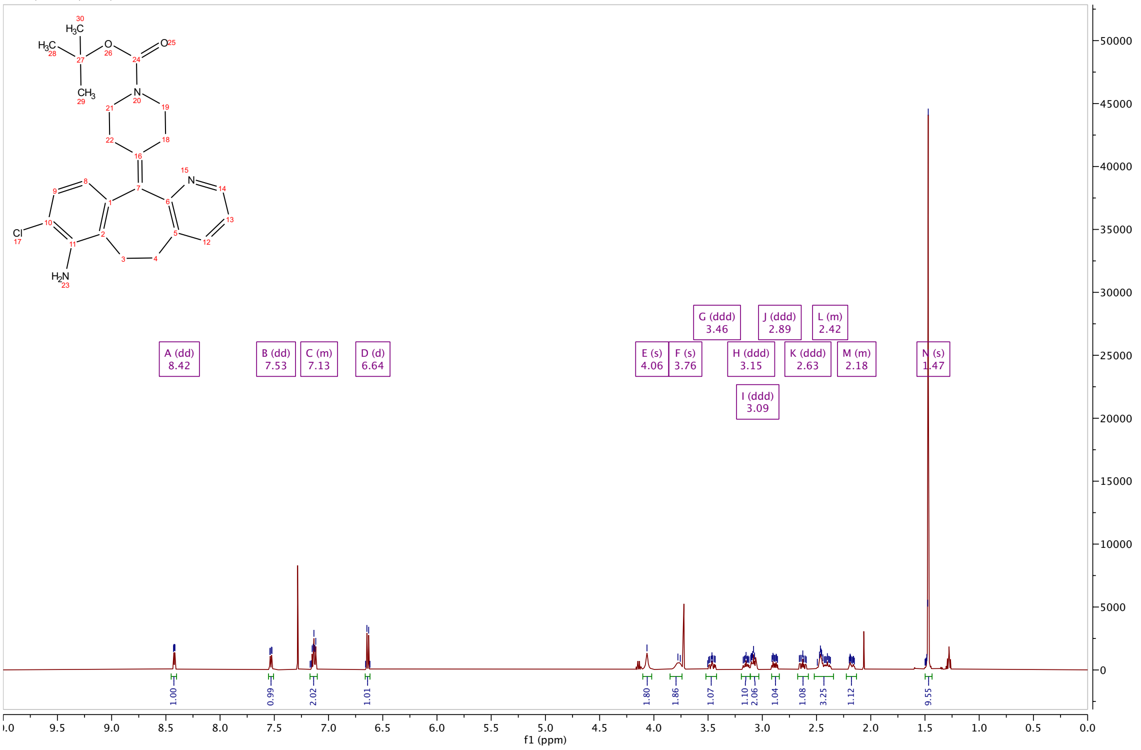

Figure S93. ^{1}H NMR spectrum of **31**.

^{13}C NMR (126 MHz, $CDCl_3$) δ 159.4, 154.9, 146.9, 141.1, 137.0, 136.4, 134.6, 133.7, 126.7, 122.5, 122.4, 120.8, 118.8, 79.7, 45.2, 30.8, 30.2, 28.6, 28.4.

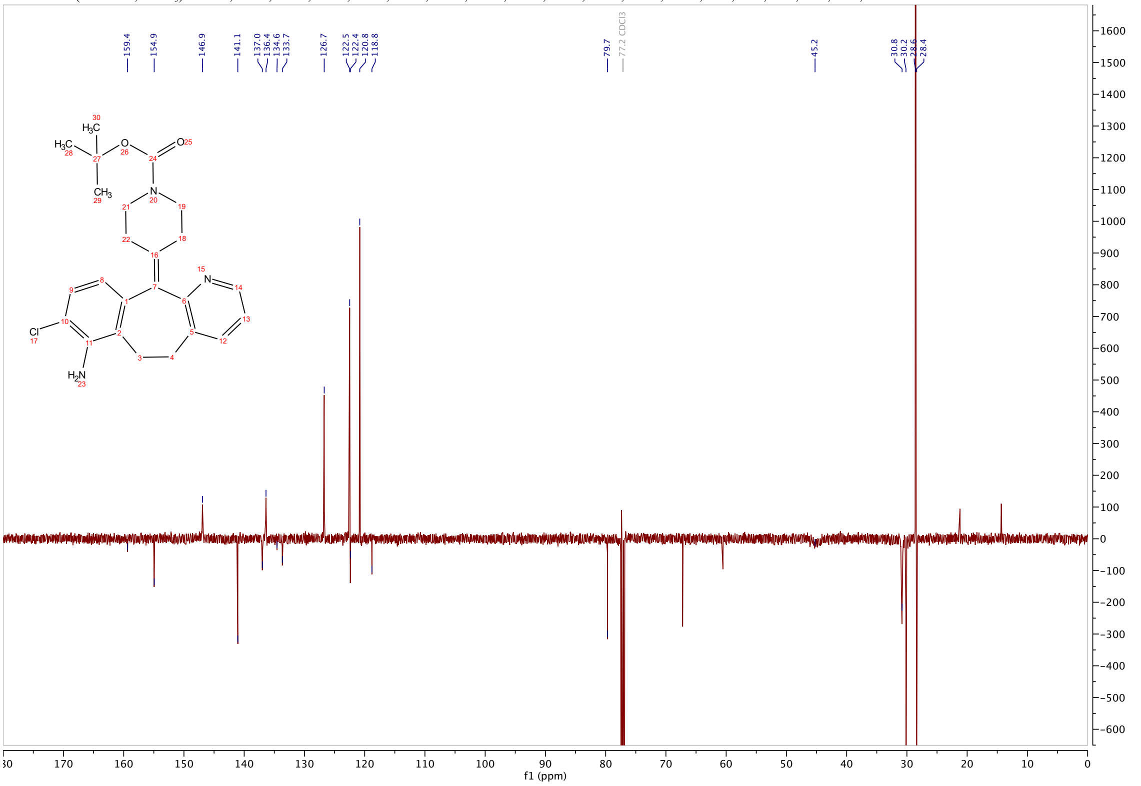

Figure S94. ^{13}C NMR spectrum of **31**.

Acquired by	: Admin
Date Acquired	: 9/3/2024 2:55:50 PM
Sample Name	: DAVE01-062-1
Sample ID	:
Tray#	: 1
Vial#	: 8
Injection Volume	: 1
Data File	: C:\LabSolutions\Data\2024\2024-wk36\DAVE01-062-1.lcd
Background File	: blanco 03092024.lcd
Method File	: Method SCAN ACID standard.lcm
Report Format	: DefaultLCMS.lcr
Tuning File	: C:\LabSolutions\Tuning File\Tuning-ESI-pos-neg01072015.lct
Processed by	: Admin
Modified Date	: 9/3/2024 3:38:21 PM

Chromatogram
DAVE01-062-1 C:\LabSolutions\Data\2024\2024-wk36\DAVE01-062-1.lcd

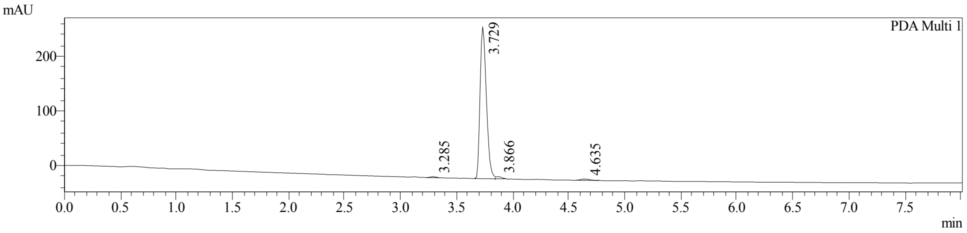

1 PDA Multi 1 / 254nm 4nm

PeakTable

PDA Ch1 254nm 4nm

Peak#	Ret. Time	Area	Height	Name	Area %
1	3.285	5288	1672		0.477
2	3.729	1079999	278706		97.361
3	3.866	11525	3688		1.039
4	4.635	12457	2305		1.123
Total		1109268	286371		100.000

MS Chromatogram
DAVE01-062-1 C:\LabSolutions\Data\2024\2024-wk36\DAVE01-062-1.lcd

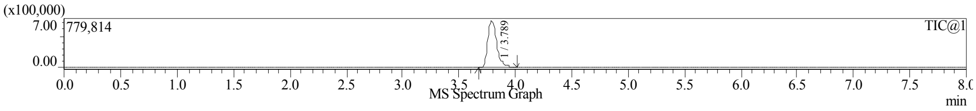

MS Spectrum Graph

#:1 Ret.Time:Averaged 3.780-3.800(Scan#:379-381)
BG Mode:Calc 3.670<->4.010(368<->402)
Mass Peaks:18 Base Peak:426.30(326540) Polarity:Pos Segment1 - Event1

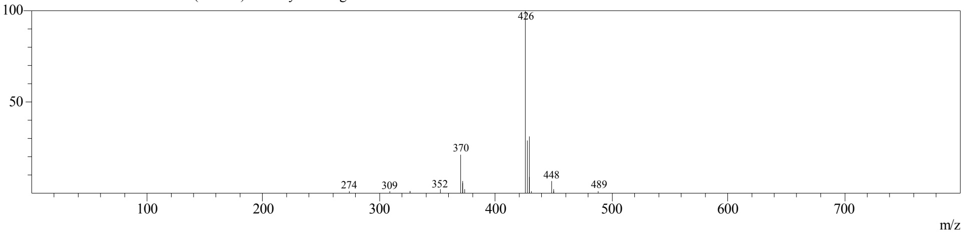

MS Spectrum Table

#:1 Ret.Time:
BG Mode:Calc 3.670<->4.010(368<->402)
Mass Peaks:18 Base Peak:426.30(326540) Polarity:Pos Segment1 - Event1

#	m/z	Abs.Inten.	Rel.Inten.	Charge	Polarity	Monoisotopic	#	m/z	Abs.Inten.	Rel.Inten.	Charge	Polarity	Monoisotopic
1	273.85	4293	1.31				8	372.15	23885	7.31			
2	308.80	3589	1.10				9	373.20	6553	2.01			
3	326.25	4730	1.45				10	426.30	326540	100.00			
4	326.75	3970	1.22				11	427.25	94912	29.07			
5	352.10	6665	2.04				12	428.30	101182	30.99			
6	370.15	70385	21.55				13	429.25	27591	8.45			
7	371.15	17863	5.47				14	430.25	3599	1.10			

Figure S95. LC-MS chromatogram of **31**.

^{1}H NMR (600 MHz, $CDCl_3$) δ 8.41 (dd, *J* = 4.8, 1.6 Hz, 1H), 7.97 – 7.92 (m, 2H), 7.62 – 7.52 (m, 3H), 7.47 – 7.40 (m, 1H), 7.31 (d, *J* = 8.2 Hz, 1H), 7.16 – 7.07 (m, 2H), 3.92 – 3.66 (m, 2H), 3.30 (ddd, *J* = 16.2, 6.8, 4.3 Hz, 1H), 3.21 – 3.06 (m, 3H), 2.96 – 2.79 (m, 2H), 2.52 (ddd, *J* = 14.1, 9.2, 4.6 Hz, 1H), 2.44 – 2.32 (m, 3H), 1.46 (s, 9H).

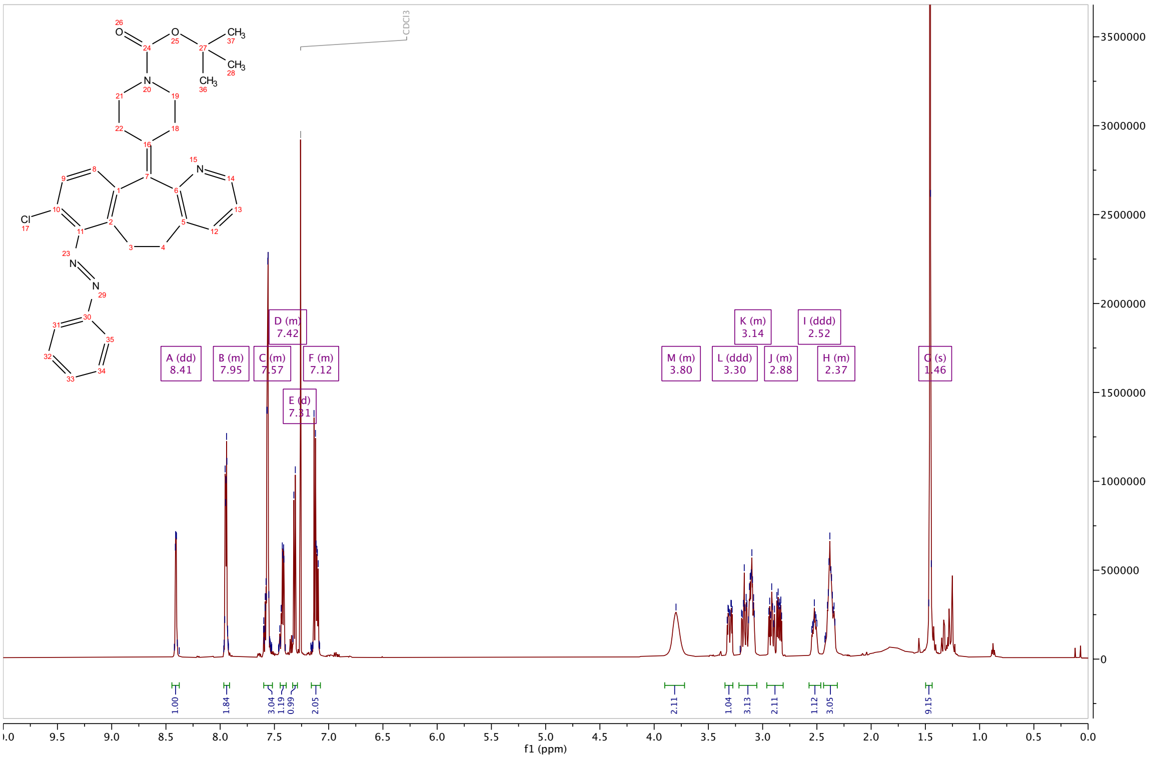

Figure S96. ^{1}H NMR spectrum of **32**.

^{13}C NMR (151 MHz, $CDCl_3$) δ 156.3, 154.9, 152.5, 150.2, 146.6, 140.6, 138.7, 138.2, 133.9, 133.6, 132.2, 130.9, 129.4, 129.1, 128.2, 123.1, 122.5, 79.8, 45.3, 44.5, 31.5, 31.1, 30.8, 28.6, 26.7.

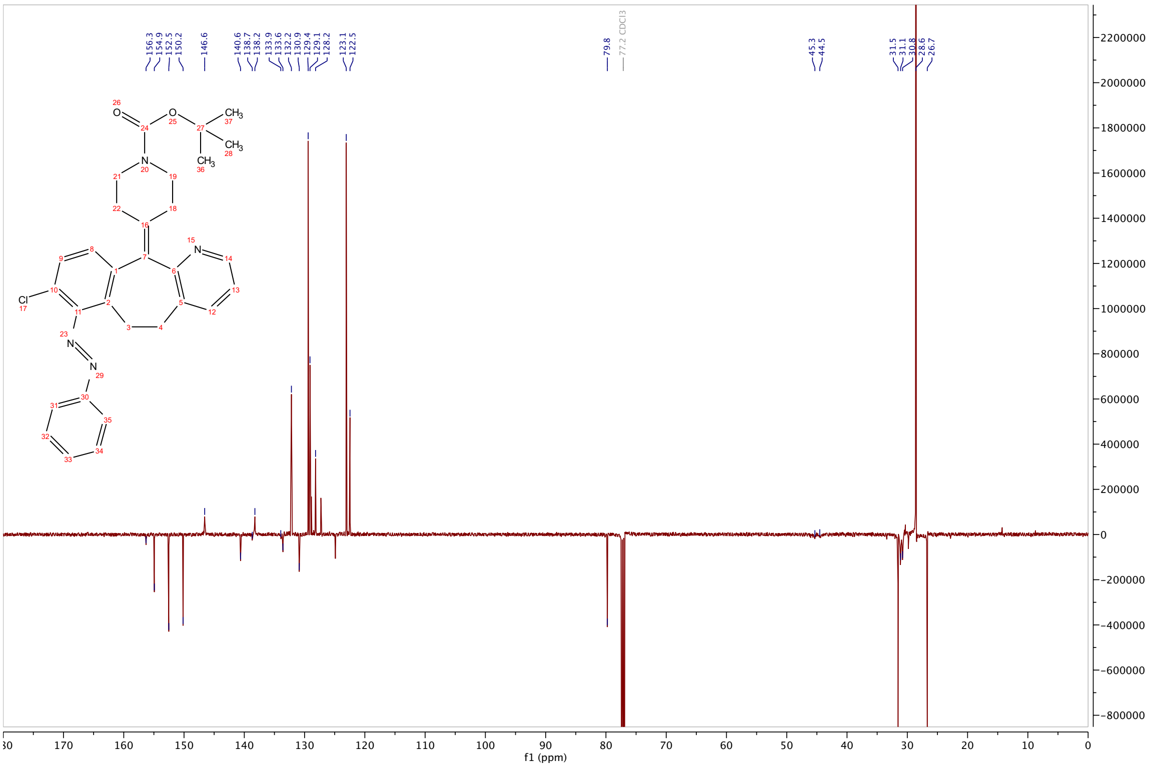

Figure S97. ^{13}C NMR spectrum of **32**.

Acquired by	: Admin
Date Acquired	: 10/21/2024 9:26:38 AM
Sample Name	: DAVE01-099-2
Sample ID	:
Tray#	: 1
Vial#	: 3
Injection Volume	: 5
Data File	: C:\LabSolutions\Data\2024\2024-wk43\DAVE01-099-2.lcd
Background File	: azoblanco 21102024.lcd
Method File	: Method SCAN ACID standard azo.lcm
Report Format	: DefaultLCMS.lcr
Tuning File	: C:\LabSolutions\Tuning File\Tuning-ESI-pos-neg01072015.lct
Processed by	: Admin
Modified Date	: 10/22/2024 1:55:06 PM

Chromatogram
DAVE01-099-2 C:\LabSolutions\Data\2024\2024-wk43\DAVE01-099-2.lcd

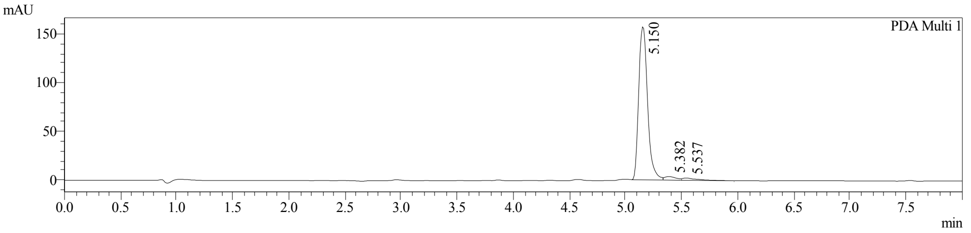

1 PDA Multi 1 / 254nm 4nm

PeakTable

PDA Ch1 254nm 4nm

Peak#	Ret. Time	Area	Height	Name	Area %
1	5.150	865595	156868		95.030
2	5.382	25170	3536		2.763
3	5.537	20096	2239		2.206
Total		910861	162643		100.000

MS Chromatogram
DAVE01-099-2 C:\LabSolutions\Data\2024\2024-wk43\DAVE01-099-2.lcd

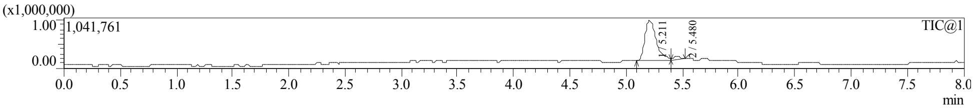

MS Spectrum Graph

#:1 Ret.Time:Averaged 5.200-5.220(Scan#:521-523)
BG Mode:Calc 5.090<->5.400(510<->541)
Mass Peaks:16 Base Peak:515.30(383881) Polarity:Pos Segment1 - Event1

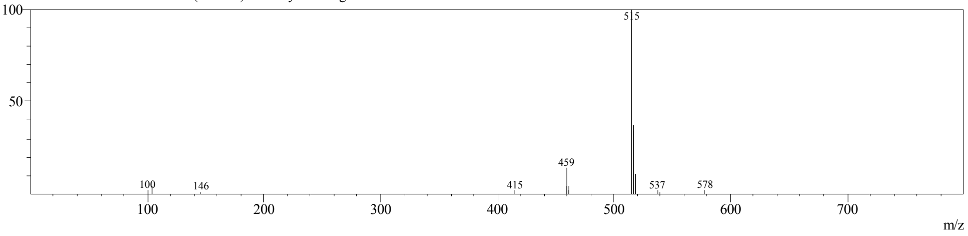

#:2 Ret.Time:Averaged 5.470-5.490(Scan#:548-550)
BG Mode:Calc 5.400<->5.530(541<->554)
Mass Peaks:83 Base Peak:100.05(18416) Polarity:Pos Segment1 - Event1

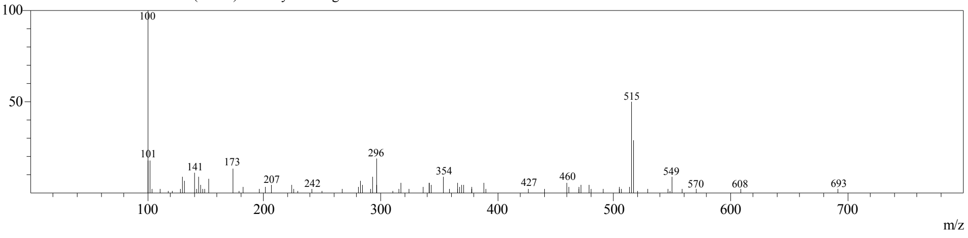

Figure S98. LC-MS chromatogram of **32**.

^{1}H NMR (600 MHz, DMSO) δ 8.53 – 8.50 (m, 1H), 8.38 – 8.32 (m, 1H), 7.91 (dd, *J* = 6.6, 3.0 Hz, 2H), 7.65 (dd, *J* = 5.2, 1.9 Hz, 3H), 7.54 (dd, *J* = 7.7, 1.7 Hz, 1H), 7.42 (d, *J* = 8.2 Hz, 1H), 7.18 (dd, *J* = 7.7, 4.7 Hz, 1H), 7.11 (d, *J* = 8.2 Hz, 1H), 3.32 – 3.24 (m, 1H), 3.11 (ddd, *J* = 14.9, 10.0, 4.5 Hz, 1H), 2.91 – 2.78 (m, 3H), 2.66 (ddd, *J* = 15.1, 7.5, 4.5 Hz, 1H), 2.58 (dddd, *J* = 31.7, 11.9, 8.2, 4.3 Hz, 2H), 2.31 – 2.09 (m, 4H).

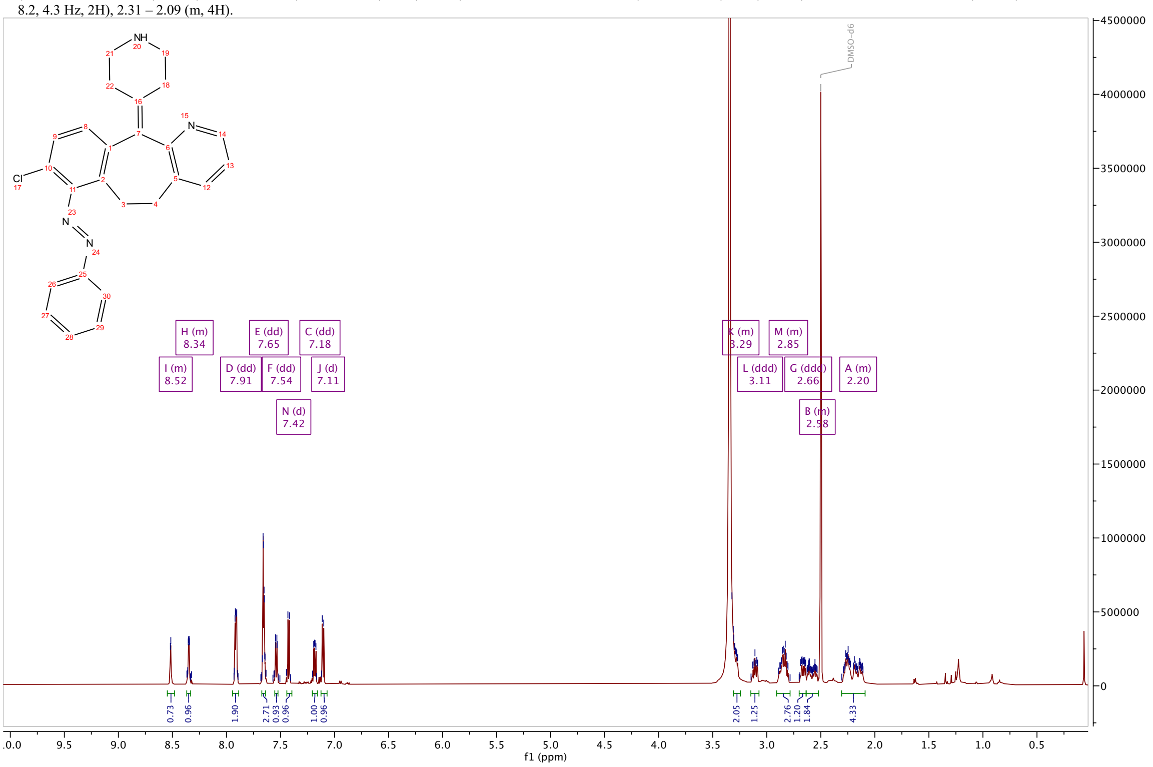

Figure S99. ^{1}H NMR spectrum of **11c**.

^{13}C NMR (151 MHz, DMSO) δ 156.7, 151.9, 149.5, 146.4, 140.8, 140.1, 137.6, 132.9, 132.6, 131.2, 130.7, 129.7, 129.4, 127.5, 122.8, 122.6, 122.2, 47.9, 47.8, 32.7, 32.4, 30.5, 26.6.

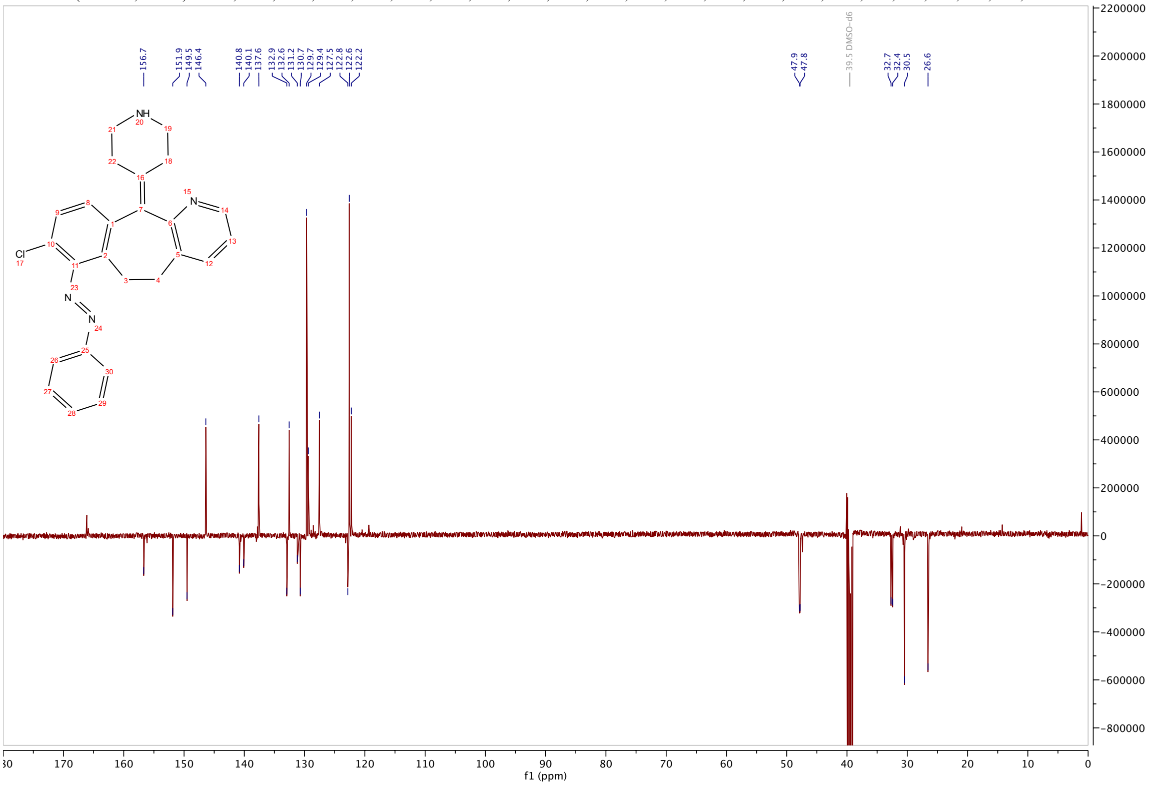

Figure S100. ^{13}C NMR spectrum of **11c**.

Acquired by	: Admin
Date Acquired	: 11/4/2024 9:18:38 AM
Sample Name	: DAVE01-100-5
Sample ID	:
Tray#	: 1
Vial#	: 1
Injection Volume	: 4
Data File	: C:\LabSolutions\Data\2024\2024-wk45\DAVE01-100-5.lcd
Background File	: azoblanco 04112024.lcd
Method File	: Method SCAN ACID standard azo.lcm
Report Format	: DefaultLCMS.lcr
Tuning File	: C:\LabSolutions\Tuning File\Tuning-ESI-pos-neg01072015.lct
Processed by	: Admin
Modified Date	: 11/4/2024 2:54:25 PM

Chromatogram
DAVE01-100-5 C:\LabSolutions\Data\2024\2024-wk45\DAVE01-100-5.lcd

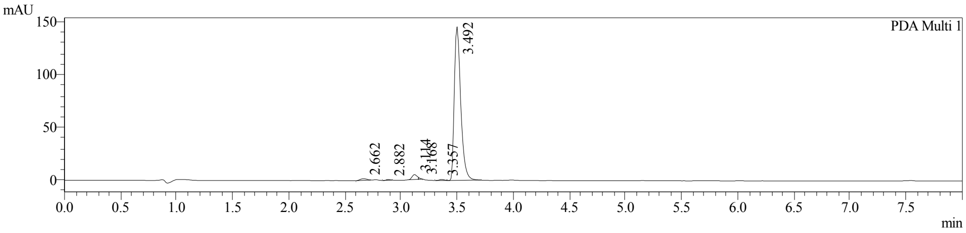

1 PDA Multi 1 / 254nm 4nm

PeakTable

PDA Ch1 254nm 4nm

Peak#	Ret. Time	Area	Height	Name	Area %
1	2.662	6596	1553		1.076
2	2.882	1771	680		0.289
3	3.114	14845	4595		2.421
4	3.168	760	787		0.124
5	3.357	2789	930		0.455
6	3.492	586517	145347		95.636
Total		613278	153892		100.000

MS Chromatogram
DAVE01-100-5 C:\LabSolutions\Data\2024\2024-wk45\DAVE01-100-5.lcd

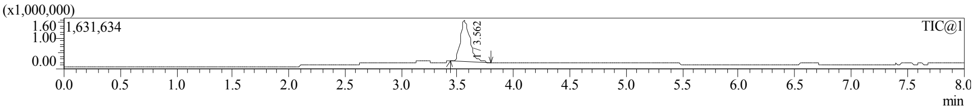

MS Spectrum Graph

#:1 Ret.Time:Averaged 3.550-3.570(Scan#:356-358)
BG Mode:Calc 3.430<->3.800(344<->381)
Mass Peaks:14 Base Peak:415.15(650040) Polarity:Pos Segment1 - Event1

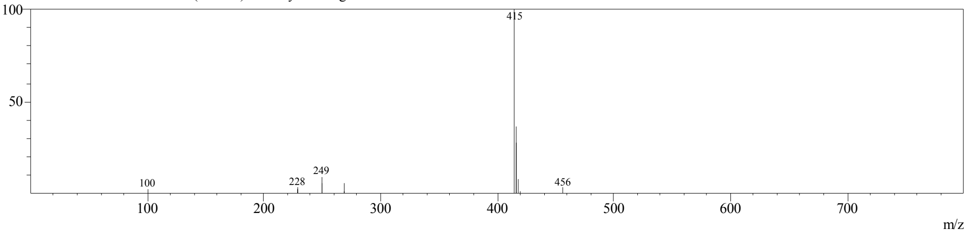

Figure S101. LC-MS chromatogram of **11c**.

HRMS MedChem

**Analysis Info**		Acquisition Date	11/5/2024 9:50:00 AM
Analysis Name	D:\Data\ServiceMS\Hans\2024-wk45\DAVE01-100_11-5-2024_09-49-02_ServiceMs Hystar.d		
Method	ServiceMs Hystar.m	Operator	Demo User
Sample Name	DAVE01-100	Instrument	impact II
Comment			

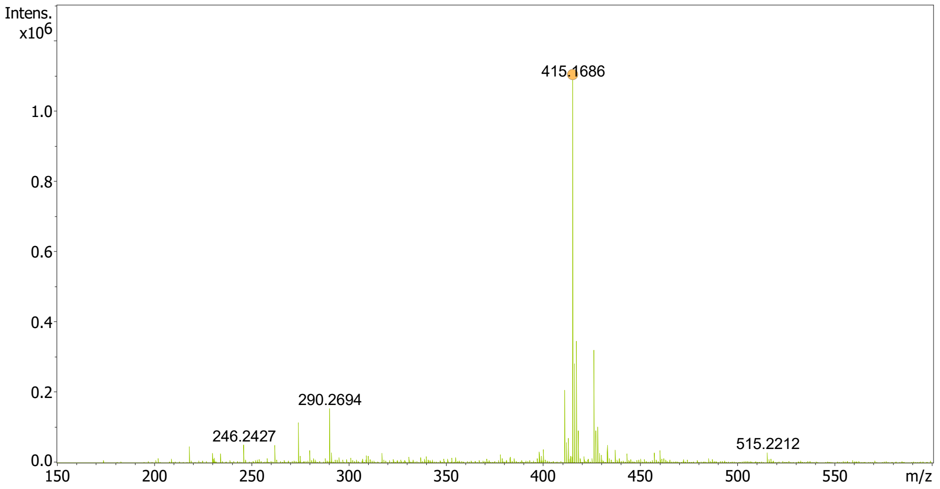

Meas. m/z	#	Ion Formula	m/z	err [ppm]	mSigma	#mSigma	Score	rdb	e⁻ Conf	N-Rule
415.1686	1	C25H24ClN4	415.1684	-0.5	23.4	1	100.00	19.0	even	ok

Figure S102. HRMS spectrum of **11c**.

^{1}H NMR (600 MHz, $CDCl_3$) δ 7.95 – 7.90 (m, 4H), 7.55 – 7.50 (m, 4H), 7.50 – 7.46 (m, 1H), 4.81 – 4.78 (m, 2H), 1.80 (t, *J* = 5.5 Hz, 1H).

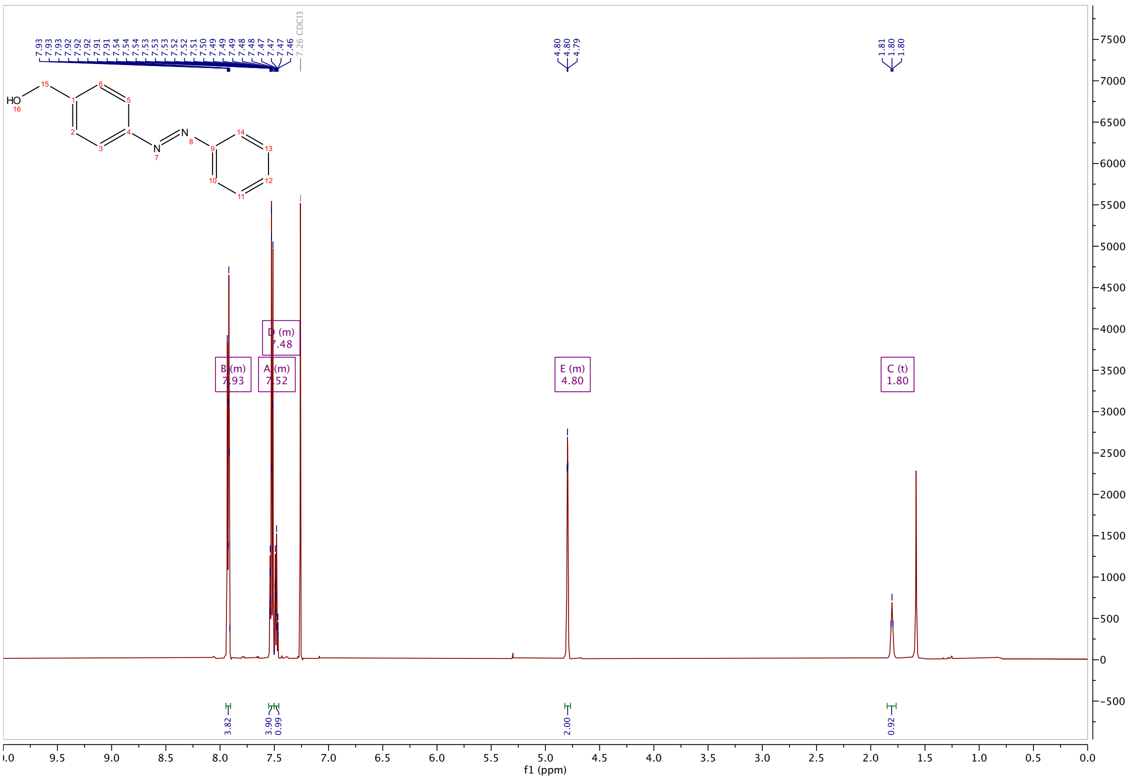

Figure S103. ^{1}H NMR spectrum of **37.**

^{13}C NMR (151 MHz, $CDCl_3$) δ 152.75, 152.23, 143.94, 131.16, 129.24, 127.58, 123.23, 122.98, 65.04.

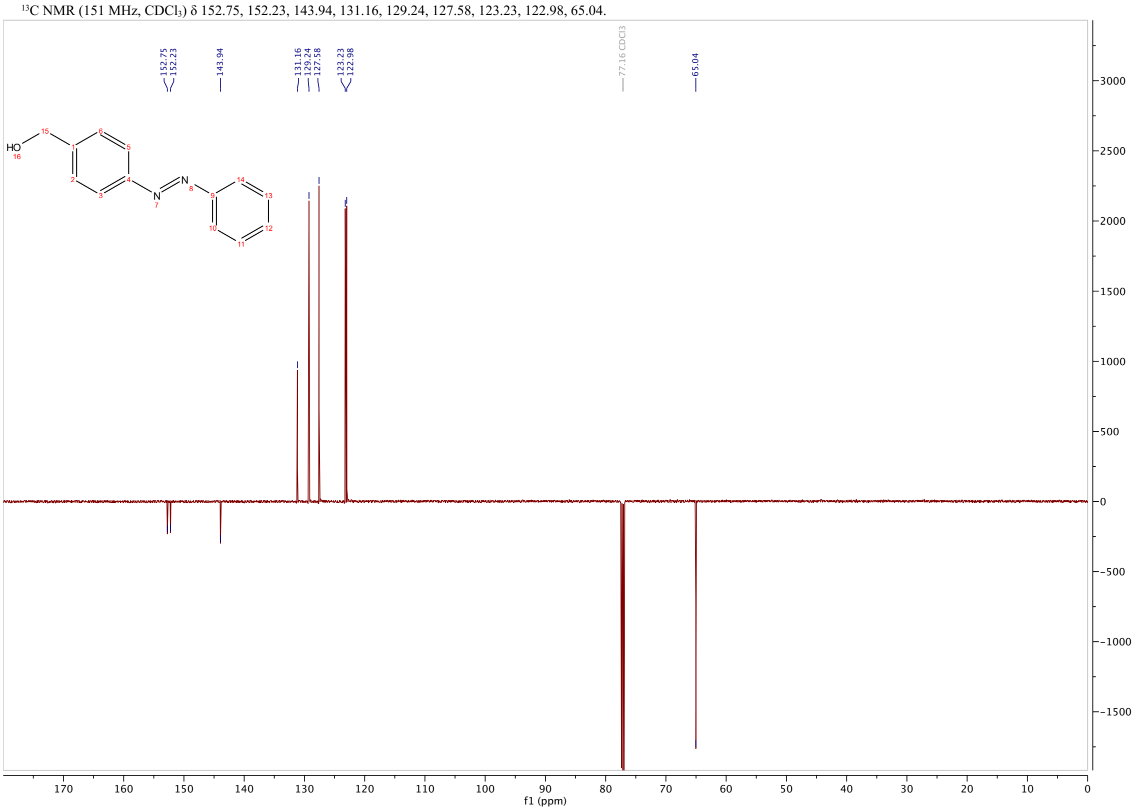

Figure S104. ^{13}C NMR spectrum of **37**.

Acquired by	: Admin
Date Acquired	: 4/18/2024 9:14:53 AM
Sample Name	: DAVE01-001-7
Sample ID	:
Tray#	: 1
Vial#	: 1
Injection Volume	: 1
Data File	: C:\LabSolutions\Data\2024\2024-wk16\DAVE01-001-7.lcd
Background File	: blanco 18042024.lcd
Method File	: Method SCAN ACID standard azo.lcm
Report Format	: DefaultLCMS.lcr
Tuning File	: C:\LabSolutions\Tuning File\Tuning-ESI-pos-neg01072015.lct
Processed by	: Admin
Modified Date	: 4/19/2024 3:44:41 PM

Chromatogram
DAVE01-001-7 C:\LabSolutions\Data\2024\2024-wk16\DAVE01-001-7.lcd

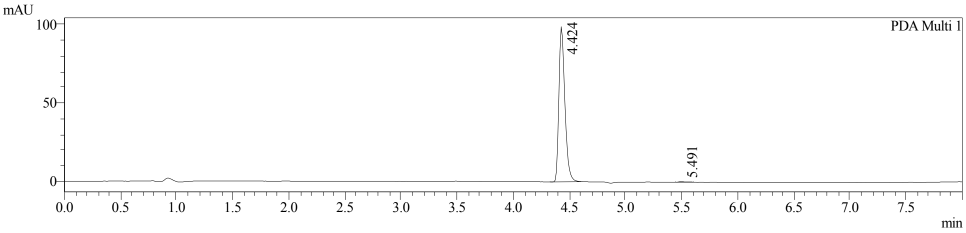

1 PDA Multi 1 / 254nm 4nm

PeakTable

PDA Ch1 254nm 4nm

Peak#	Ret. Time	Area	Height	Name	Area %
1	4.424	364575	99133		99.517
2	5.491	1769	423		0.483
Total		366344	99556		100.000

MS Chromatogram
DAVE01-001-7 C:\LabSolutions\Data\2024\2024-wk16\DAVE01-001-7.lcd

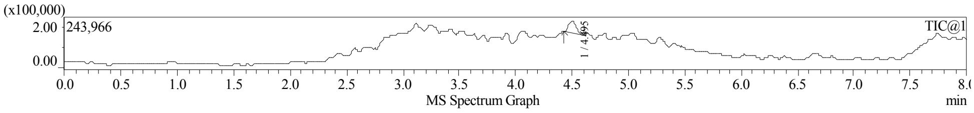

MS Spectrum Graph

#:1 Ret.Time:Averaged 4.490-4.510(Scan#:450-452)
BG Mode:Calc 4.430<->4.600(444<->461)
Mass Peaks:49 Base Peak:213.00(21993) Polarity:Pos Segment1 - Event1

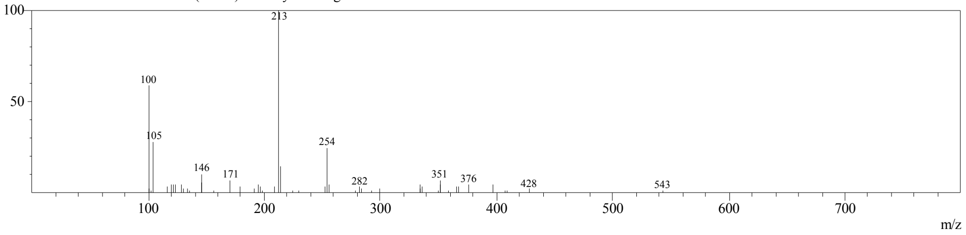

MS Spectrum Table

#:1 Ret.Time:
BG Mode:Calc 4.430<->4.600(444<->461)
Mass Peaks:49 Base Peak:213.00(21993) Polarity:Pos Segment1 - Event1

#	m/z	Abs.Inten.	Rel.Inten.	Charge	Polarity	Monoisotopic
1	100.05	12944	58.86			
2	101.00	615	2.80			
3	102.10	339	1.54			
4	104.90	6221	28.29			
5	116.05	859	3.91			
6	120.00	978	4.45			
7	120.90	984	4.47			
8	123.05	1011	4.60			

#	m/z	Abs.Inten.	Rel.Inten.	Charge	Polarity	Monoisotopic
9	128.90	922	4.19			
10	130.05	557	2.53			
11	134.10	453	2.06			
12	135.05	379	1.72			
13	145.90	2320	10.55			
14	146.80	1176	5.35			
15	156.05	351	1.60			
16	171.00	1580	7.18			

Figure S105. LC-MS chromatogram of **37**.

^{1}H NMR (600 MHz, $CDCl_3$) δ 10.11 (s, 1H), 8.06 – 8.03 (m, 4H), 7.99 – 7.95 (m, 2H), 7.58 – 7.51 (m, 3H).

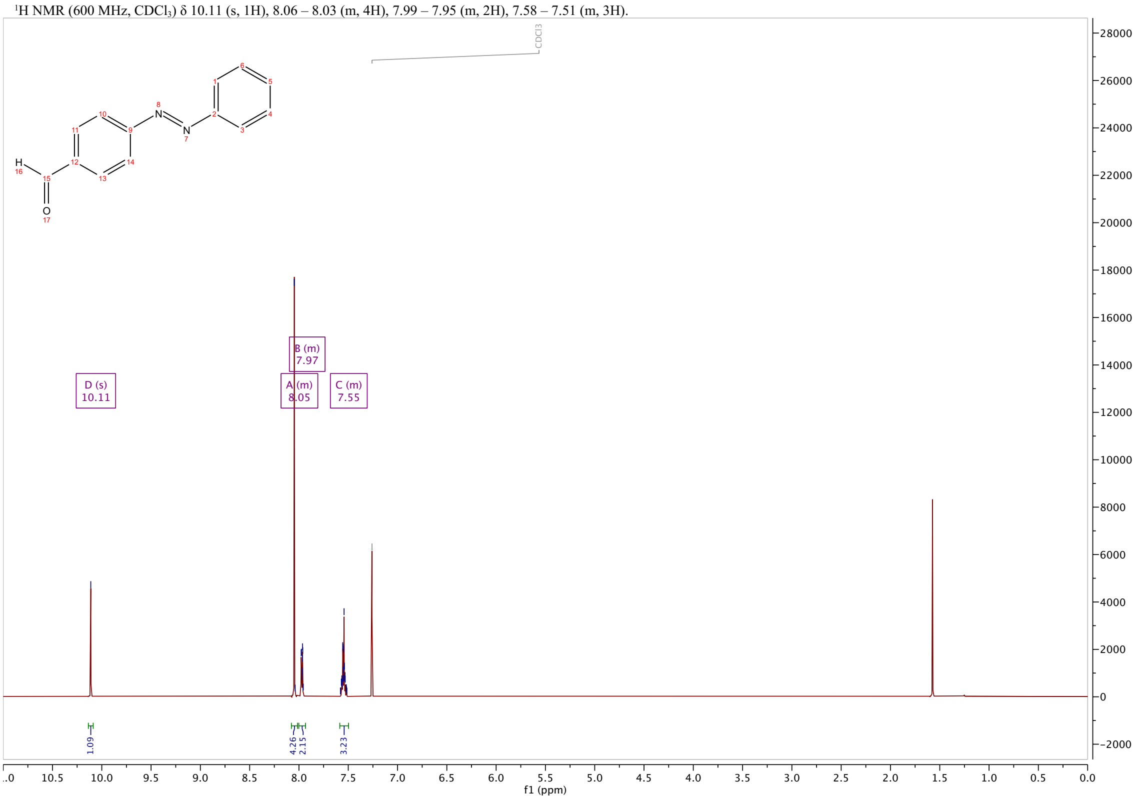

Figure S106. ^{1}H NMR spectrum of **39.**

^{13}C NMR (151 MHz, $CDCl_3$) δ 191.8, 156.0, 152.7, 137.6, 132.1, 130.9, 129.4, 123.5, 123.4.

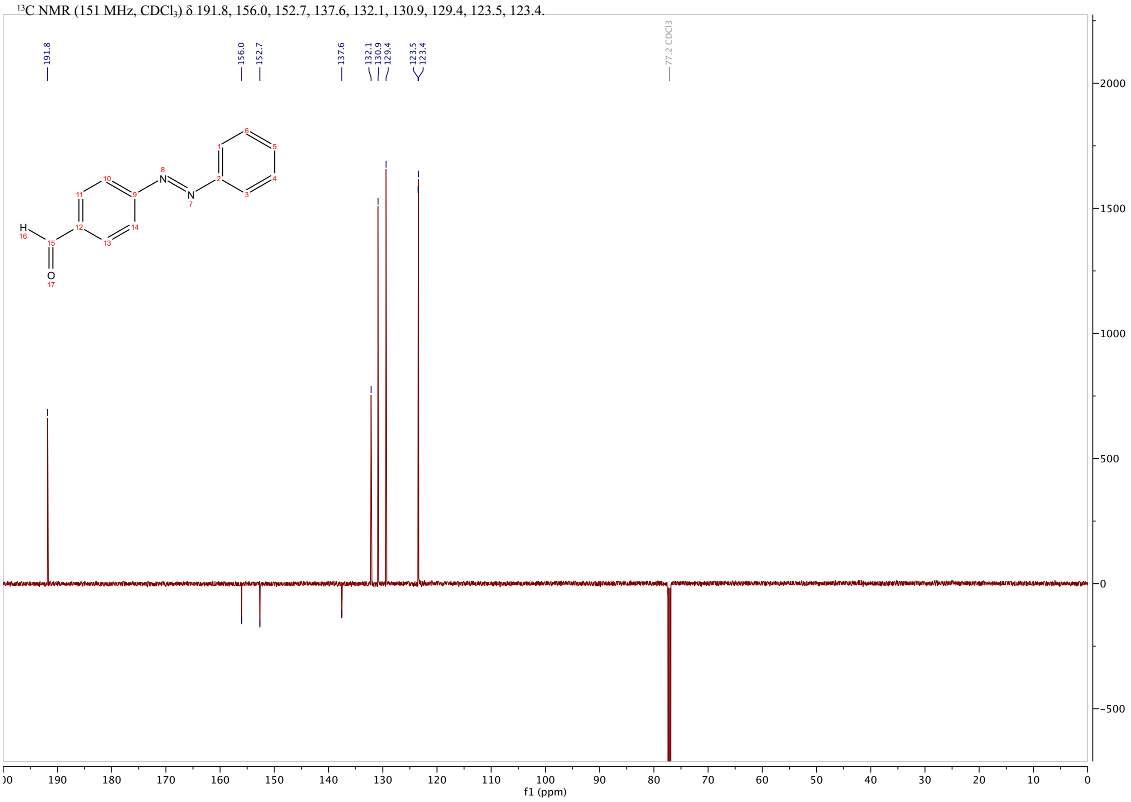

Figure S107. ^{13}C NMR spectrum of **39**.

Acquired by	: Admin
Date Acquired	: 4/22/2024 1:49:56 PM
Sample Name	: DAVE01-003-3
Sample ID	:
Tray#	: 1
Vial#	: 2
Injection Volume	: 1
Data File	: C:\LabSolutions\Data\2024\2024-wk17\DAVE01-003-3.lcd
Background File	: azoblanco 22042024.lcd
Method File	: Method SCAN ACID standard azo.lcm
Report Format	: DefaultLCMS.lcr
Tuning File	: C:\LabSolutions\Tuning File\Tuning-ESI-pos-neg01072015.lct
Processed by	: Admin
Modified Date	: 4/22/2024 2:04:39 PM

Chromatogram
DAVE01-003-3 C:\LabSolutions\Data\2024\2024-wk17\DAVE01-003-3.lcd

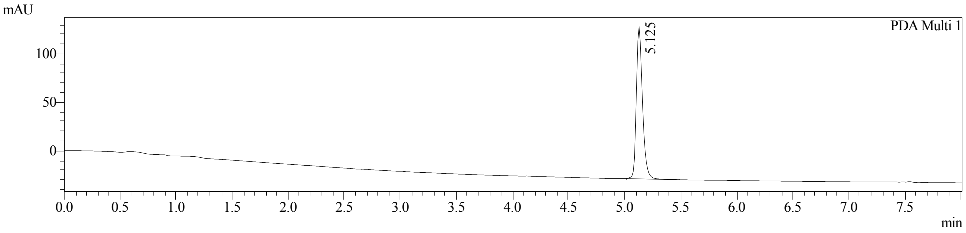

1 PDA Multi 1 / 254nm 4nm

PeakTable

PDA Ch1 254nm 4nm

Peak#	Ret. Time	Area	Height	Name	Area %
1	5.125	593193	157000		100.000
Total		593193	157000		100.000

MS Chromatogram
DAVE01-003-3 C:\LabSolutions\Data\2024\2024-wk17\DAVE01-003-3.lcd

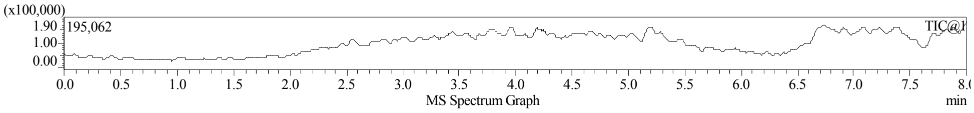

MS Spectrum Graph

#:1 Ret.Time:Averaged 5.180-5.200(Scan#:519-521)
BG Mode:None
Mass Peaks:34 Base Peak:120.00(46871) Polarity:Pos Segment1 - Event1

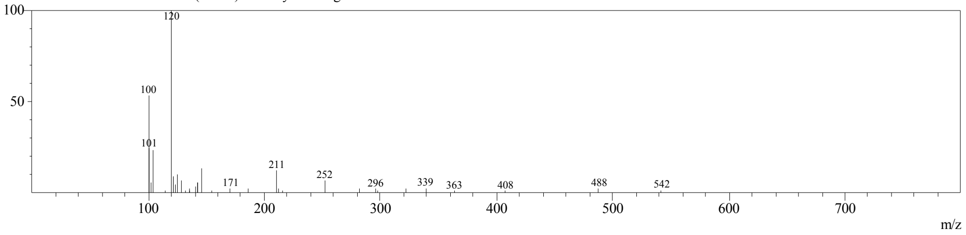

MS Spectrum Table

#:1 Ret.Time:
BG Mode:None
Mass Peaks:34 Base Peak:120.00(46871) Polarity:Pos Segment1 - Event1

#	m/z	Abs.Inten.	Rel.Inten.	Charge	Polarity	Monoisotopic
1	100.05	24918	53.16			
2	100.85	11250	24.00			
3	102.10	2470	5.27			
4	104.90	11199	23.89			
5	115.05	538	1.15			
6	120.00	46871	100.00			
7	120.85	4052	8.65			
8	122.10	4194	8.95			
9	124.10	1959	4.18			
10	125.85	4566	9.74			
11	128.10	3422	7.30			
12	132.10	857	1.83			
13	134.85	899	1.92			
14	135.85	1199	2.56			
15	141.00	1838	3.92			
16	141.80	2753	5.87			

Figure S108. LC-MS chromatogram of **39**.

^{1}H NMR (600 MHz, $CDCl_3$) δ 8.4 (dd, J = 4.9, 1.7 Hz, 1H), 7.9 – 7.9 (m, 2H), 7.9 – 7.8 (m, 2H), 7.5 – 7.5 (m, 2H), 7.5 – 7.5 (m, 2H), 7.5 – 7.4 (m, 1H), 7.4 (dd, J = 7.7, 1.7 Hz, 1H), 7.2 – 7.1 (m, 2H), 7.1 (dd, J = 8.2, 2.0 Hz, 1H), 7.1 (dd, J = 7.6, 4.8 Hz, 1H), 3.6 (s, 2H), 3.5 – 3.3 (m, 2H), 2.9 – 2.8 (m, 2H), 2.8 – 2.7 (m, 2H), 2.6 (ddd, J = 14.1, 9.9, 4.2 Hz, 1H), 2.5 (ddd, J = 13.9, 9.7, 4.2 Hz, 1H), 2.4 – 2.3 (m, 2H), 2.2 – 2.1 (m, 2H).

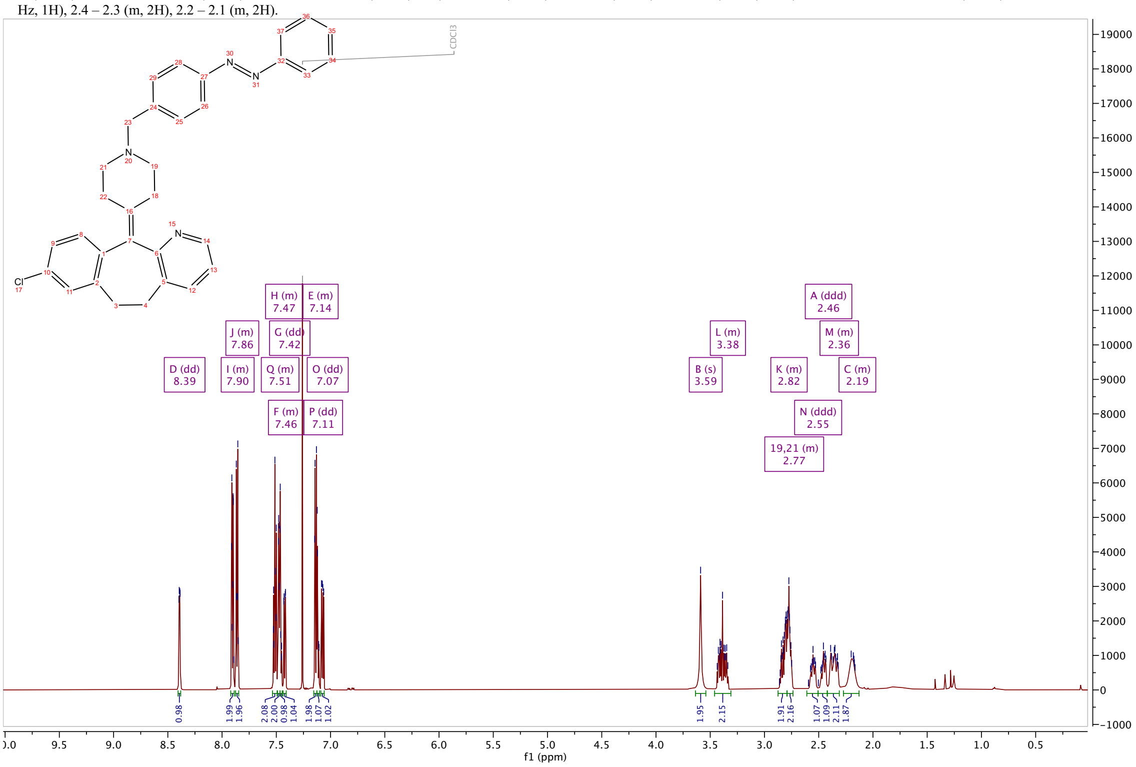

Figure S109. ^{1}H NMR spectrum of **12a.**

^{13}C NMR (151 MHz, $CDCl_3$) δ 157.7, 152.8, 152.0, 146.8, 141.9, 139.6, 139.0, 138.0, 137.4, 133.5, 132.8, 131.0, 131.0, 129.9, 129.2, 129.1, 126.1, 122.9, 122.9, 122.2, 62.6, 55.0, 54.9, 32.0, 31.6, 31.1, 30.9. One missing carbon (5)

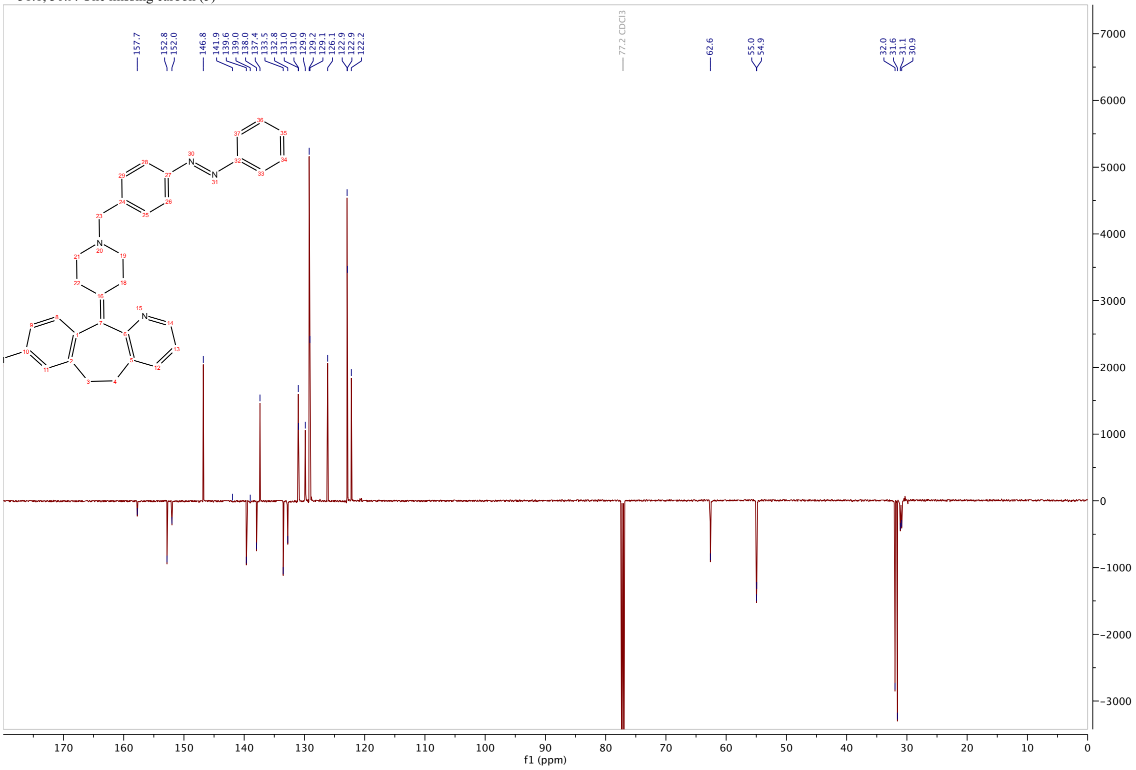

Figure S110. ^{13}C NMR spectrum of **12a**.

Acquired by	: Admin
Date Acquired	: 5/28/2024 9:57:32 AM
Sample Name	: DAVE01-006-11
Sample ID	:
Tray#	: 1
Vial#	: 4
Injection Volume	: 3
Data File	: C:\LabSolutions\Data\2024\2024-wk22\DAVE01-006-11.lcd
Background File	: azoblanco 28052024.lcd
Method File	: Method SCAN ACID standard azo.lcm
Report Format	: DefaultLCMS.lcr
Tuning File	: C:\LabSolutions\Tuning File\Tuning-ESI-pos-neg01072015.lct
Processed by	: Admin
Modified Date	: 5/28/2024 10:49:06 AM

Chromatogram
DAVE01-006-11 C:\LabSolutions\Data\2024\2024-wk22\DAVE01-006-11.lcd

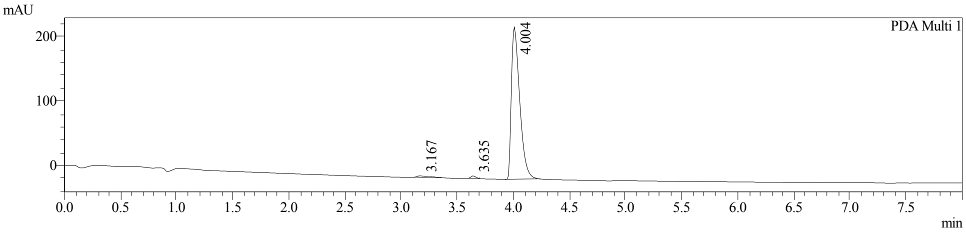

1 PDA Multi 1 / 254nm 4nm

PeakTable

PDA Ch1 254nm 4nm

Peak#	Ret. Time	Area	Height	Name	Area %
1	3.167	13648	1905		1.084
2	3.635	11245	3987		0.893
3	4.004	1234685	236398		98.024
Total		1259578	242290		100.000

MS Chromatogram
DAVE01-006-11 C:\LabSolutions\Data\2024\2024-wk22\DAVE01-006-11.lcd

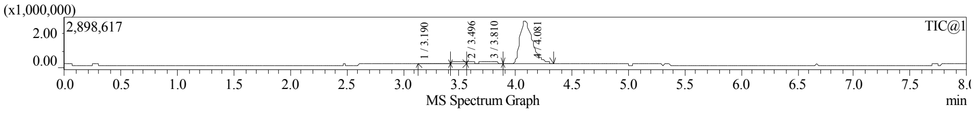

MS Spectrum Graph

#:1 Ret.Time:Averaged 4.070-4.100(Scan#:408-411)
BG Mode:None
Mass Peaks:31 Base Peak:505.20(702096) Polarity:Pos Segment1 - Event1

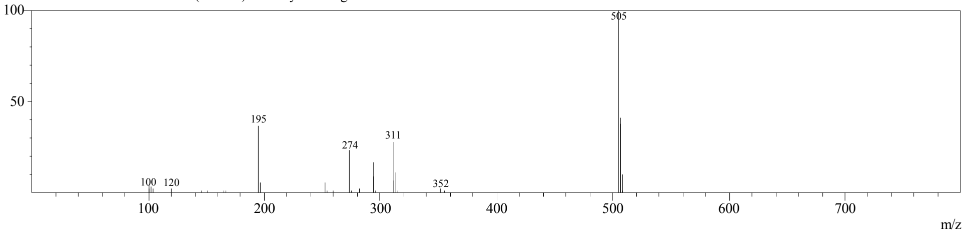

MS Spectrum Table

#:1 Ret.Time:
BG Mode:None
Mass Peaks:31 Base Peak:505.20(702096) Polarity:Pos Segment1 - Event1

#	m/z	Abs.Inten.	Rel.Inten.	Charge	Polarity	Monoisotopic	#	m/z	Abs.Inten.	Rel.Inten.	Charge	Polarity	Monoisotopic
1	100.05	19017	2.71				9	167.00	7219	1.03			
2	100.95	33315	4.75				10	195.00	260300	37.07			
3	102.00	22040	3.14				11	196.00	38080	5.42			
4	105.00	13900	1.98				12	253.25	39648	5.65			
5	120.00	16706	2.38				13	254.20	13208	1.88			
6	146.00	7091	1.01				14	259.05	13388	1.91			
7	152.00	7207	1.03				15	273.75	161592	23.02			
8	164.80	10674	1.52				16	275.40	7021	1.00			

Figure S111. LC-MS chromatogram of **2a**.

HRMS MedChem

Analysis Info

Analysis Name	D:\Data\ServiceMS\Hans\2024-wk31\DAVE VUF26783_7-31-2024_09-43-07_ServiceMs Hystar.d	Acquisition Date	7/31/2024 9:44:00 AM
Method	ServiceMs Hystar.m	Operator	Demo User
Sample Name	DAVE VUF26783	Instrument	impact II
Comment			

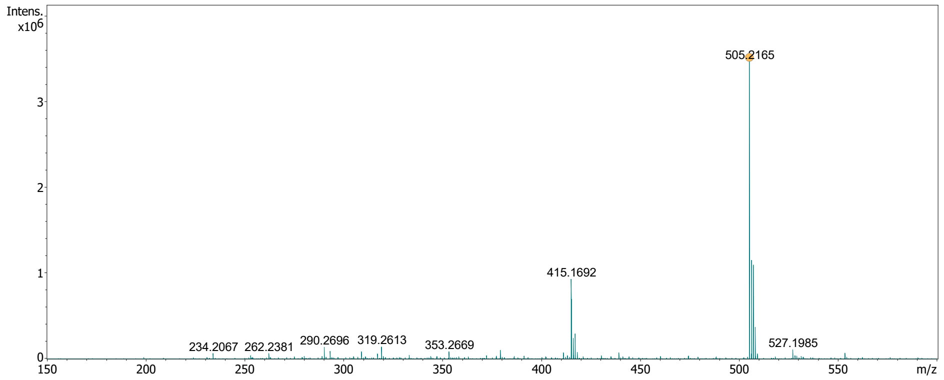

Meas. m/z	#	Ion Formula	m/z	err [ppm]	mSigma	#mSigma	Score	rdb	e⁻ Conf	N-Rule
505.2165	1	C32H30ClN4	505.2154	-2.3	34.0	1	100.00	23.0	even	ok

Bruker Compass DataAnalysis 6.0 printed: 7/31/2024 10:04:42 AM by: demo Page 1 of 1

Figure S112. HRMS spectrum of **12a**.

^{1}H NMR (500 MHz, $CDCl_3$) δ 7.95 – 7.89 (m, 3H), 7.86 (ddd, *J* = 7.5, 1.8, 1.8 Hz, 1H), 7.57 – 7.45 (m, 5H), 4.89 – 4.76 (m, 2H), 1.98 (d, *J* = 5.1 Hz, 1H).

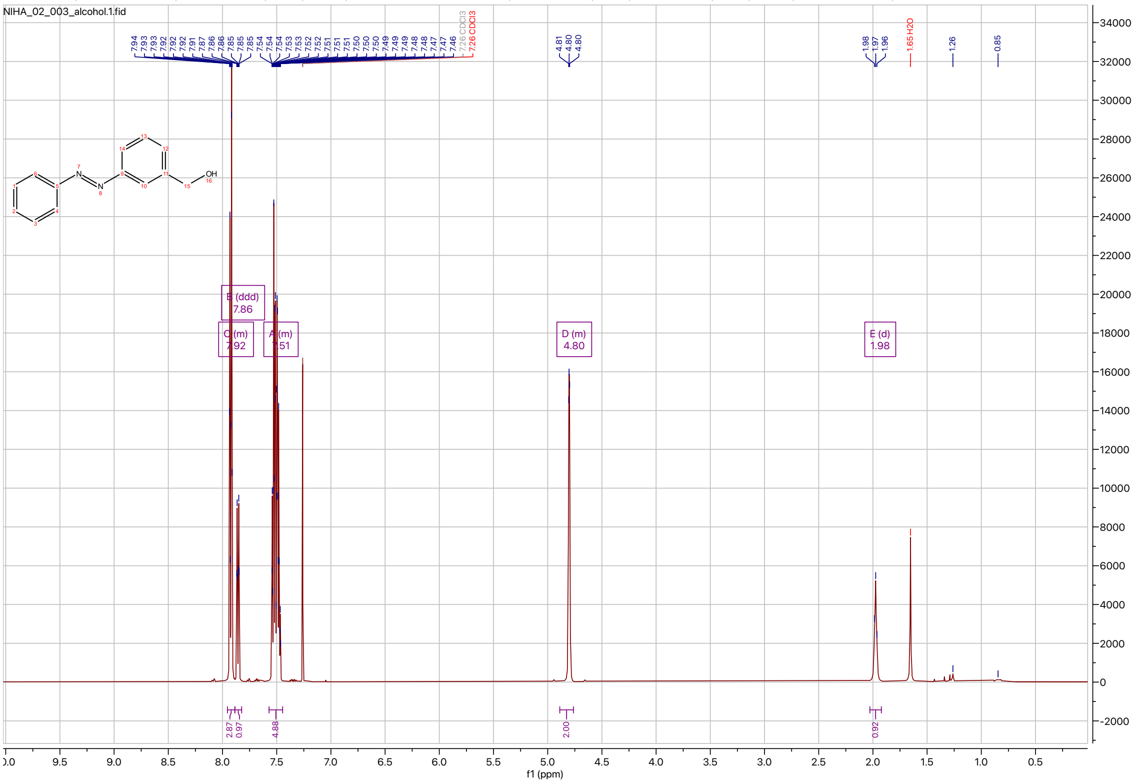

Figure S113. ^{1}H NMR spectrum of **38.**

^{13}C NMR (126 MHz, $CDCl_3$) δ 153.0, 152.7, 142.2, 131.2, 129.5, 129.4, 129.2, 123.0, 122.8, 120.7, 65.1.

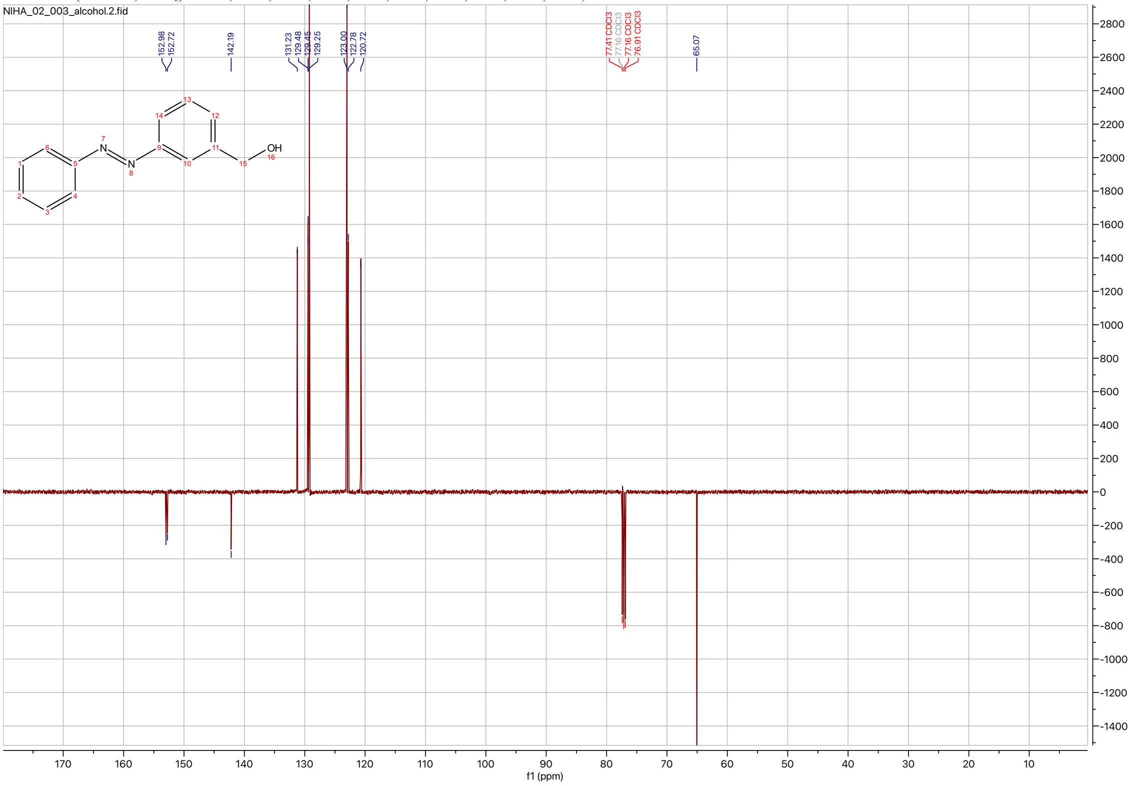

Figure S114. ^{13}C NMR spectrum of **38**.

Acquired by	: Admin
Date Acquired	: 4/14/2025 10:19:18 AM
Sample Name	: NIHA_02_003_alcohol
Sample ID	:
Tray#	: 1
Vial#	: 6
Injection Volume	: 1
Data File	: C:\LabSolutions\Data\2025\2025-wk16\NIHA_02_003_alcohol.lcd
Background File	: blanco 14042025.lcd
Method File	: Method SCAN ACID standard azo.lcm
Report Format	: DefaultLCMS.lcr
Tuning File	: C:\LabSolutions\Tuning File\Tuning-ESI-pos-neg01072015.lct
Processed by	: Admin
Modified Date	: 4/14/2025 3:28:40 PM

Chromatogram
NIHA_02_003_alcohol C:\LabSolutions\Data\2025\2025-wk16\NIHA_02_003_alcohol.lcd

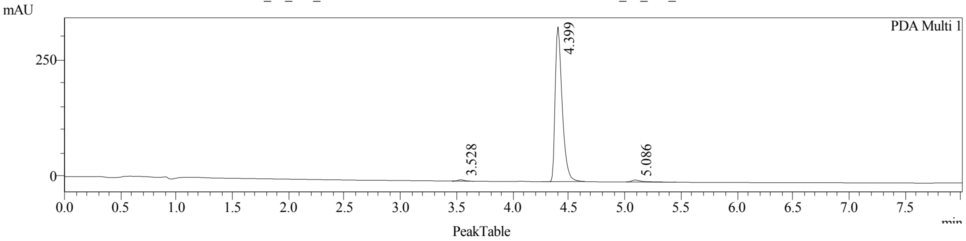

PeakTable

PDA Ch1 254nm 4nm

Peak#	Ret. Time	Area	Height	Name	Area %
1	3.528	10540	2792		0.717
2	4.399	1433210	331952		97.481
3	5.086	26503	4396		1.803
Total		1470253	339140		100.000

MS Chromatogram
NIHA_02_003_alcohol C:\LabSolutions\Data\2025\2025-wk16\NIHA_02_003_alcohol.lcd

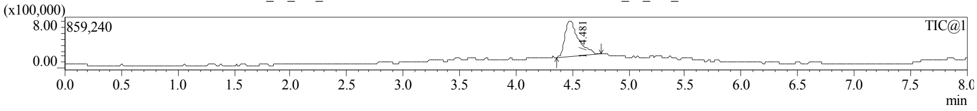

MS Spectrum Graph

#:1 Ret.Time:Averaged 4.470-4.490(Scan#:448-450)
BG Mode:Calc 4.360<->4.760(437<->477)
Mass Peaks:5 Base Peak:213.00(470615) Polarity:Pos Segment1 - Event1

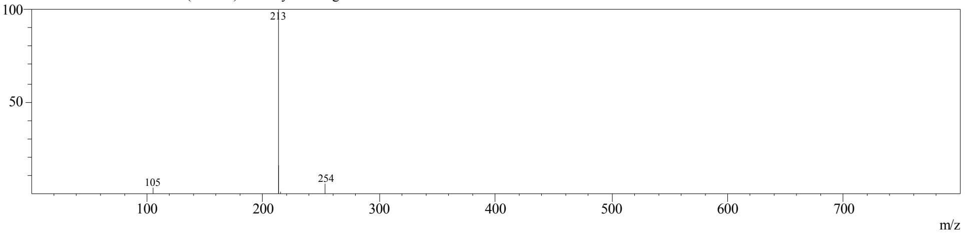

MS Spectrum Table

#:1 Ret.Time:
BG Mode:Calc 4.360<->4.760(437<->477)
Mass Peaks:5 Base Peak:213.00(470615) Polarity:Pos Segment1 - Event1

#	m/z	Abs.Inten.	Rel.Inten.	Charge	Polarity	Monoisotopic
1	105.00	12723	2.70			
2	213.00	470615	100.00			
3	214.00	71173	15.12			
4	215.10	4917	1.04			
5	254.10	23481	4.99			

Figure S115. LC-MS chromatogram of **38**.

^{1}H NMR (500 MHz, $CDCl_3$) δ 10.14 (s, 1H), 8.41 (dd, *J* = 1.8, 1.8 Hz, 1H), 8.19 (ddd, *J* = 7.9, 2.1, 1.3 Hz, 1H), 8.01 (ddd, *J* = 7.6, 1.4, 1.4 Hz, 1H), 7.99 – 7.93 (m, 2H), 7.70 (dd, *J* = 7.7, 7.7 Hz, 1H), 7.59 – 7.49 (m, 3H).

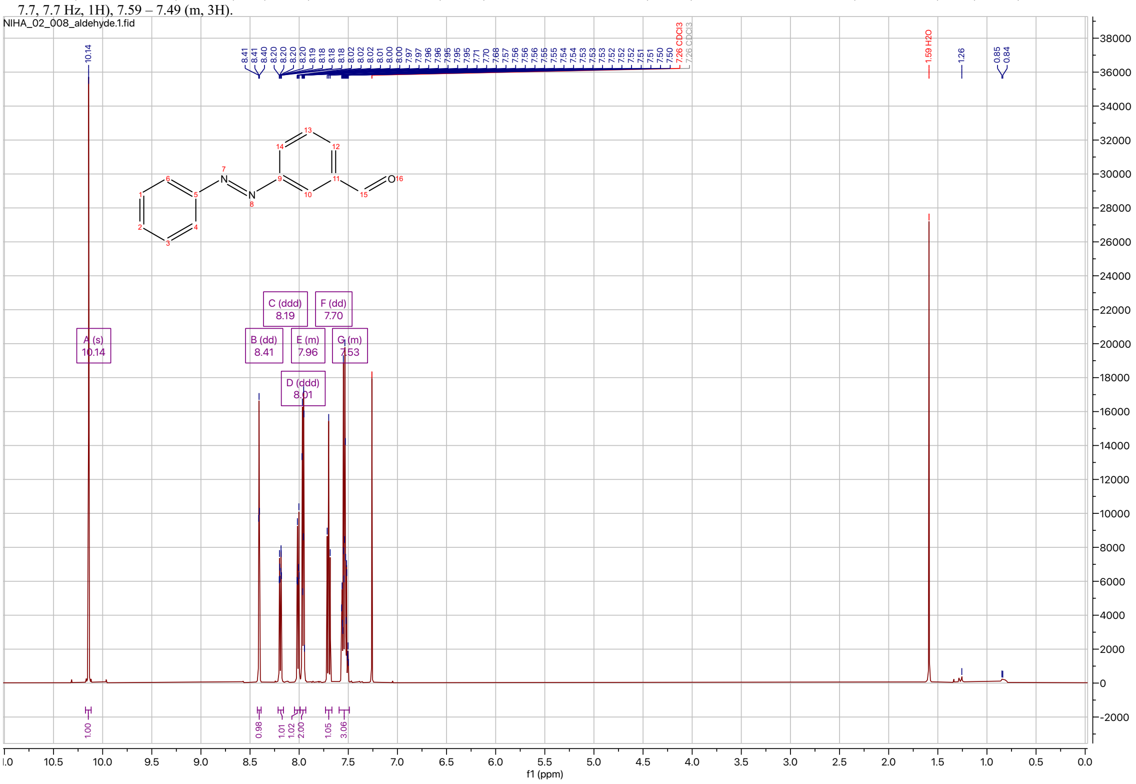

Figure S116. ^{1}H NMR spectrum of **40.**

^{13}C NMR (126 MHz, $CDCl_3$) δ 191.9, 153.1, 152.5, 137.5, 131.8, 131.2, 130.0, 129.4, 128.9, 124.0, 123.2.

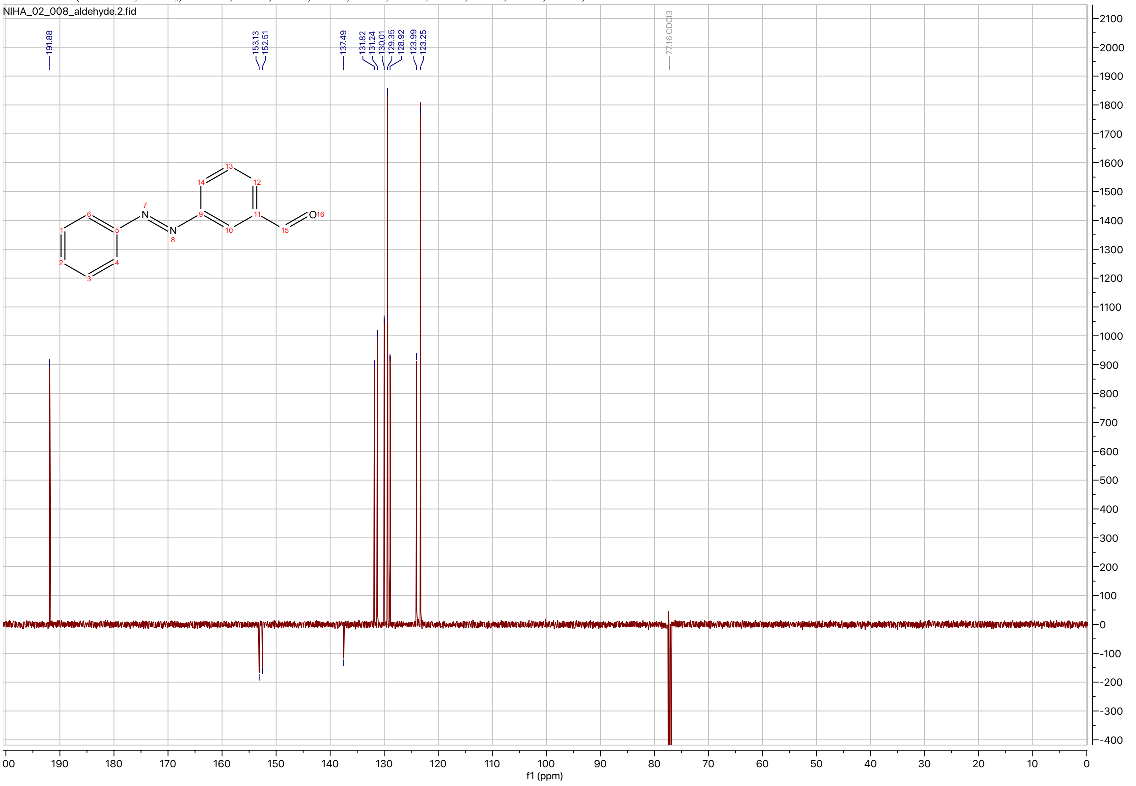

Figure S117. ^{13}C NMR spectrum of **40**.

Acquired by	: Admin
Date Acquired	: 4/14/2025 10:27:56 AM
Sample Name	: NIHA_02_008_aldehyde
Sample ID	:
Tray#	: 1
Vial#	: 5
Injection Volume	: 1
Data File	: C:\LabSolutions\Data\2025\2025-wk16\NIHA_02_008_aldehyde.lcd
Background File	: blanco 14042025.lcd
Method File	: Method SCAN ACID standard azo.lcm
Report Format	: DefaultLCMS.lcr
Tuning File	: C:\LabSolutions\Tuning File\Tuning-ESI-pos-neg01072015.lct
Processed by	: Admin
Modified Date	: 4/14/2025 3:29:21 PM

Chromatogram
NIHA_02_008_aldehyde C:\LabSolutions\Data\2025\2025-wk16\NIHA_02_008_aldehyde.lcd

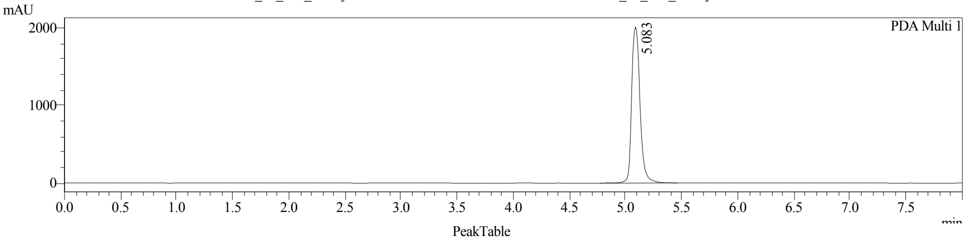

PeakTable

PDA Ch1 254nm 4nm

Peak#	Ret. Time	Area	Height	Name	Area %
1	5.083	10433187	2010215		100.000
Total		10433187	2010215		100.000

MS Chromatogram
NIHA_02_008_aldehyde C:\LabSolutions\Data\2025\2025-wk16\NIHA_02_008_aldehyde.lcd

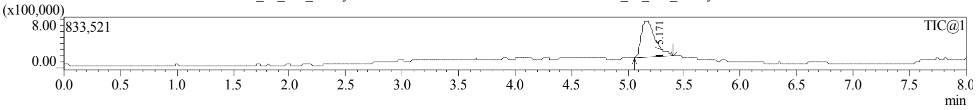

MS Spectrum Graph

#:1 Ret.Time:Averaged 5.160-5.180(Scan#:517-519)
BG Mode:Calc 5.060<->5.410(507<->542)
Mass Peaks:10 Base Peak:211.00(366551) Polarity:Pos Segment1 - Event1

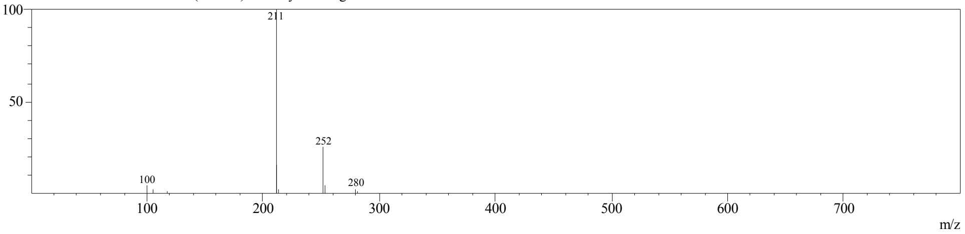

MS Spectrum Table

#:1 Ret.Time:
BG Mode:Calc 5.060<->5.410(507<->542)
Mass Peaks:10 Base Peak:211.00(366551) Polarity:Pos Segment1 - Event1

#	m/z	Abs.Inten.	Rel.Inten.	Charge	Polarity	Monoisotopic
1	100.10	14842	4.05			
2	105.05	6287	1.72			
3	118.05	4159	1.13			
4	211.00	366551	100.00			
5	212.05	56476	15.41			
6	213.10	7329	2.00			
7	252.10	92223	25.16			
8	253.10	17137	4.68			
9	280.10	8952	2.44			
10	282.10	3788	1.03			

Figure S118. LC-MS chromatogram of **40**.

^{1}H NMR (600 MHz, $CDCl_3$) δ 8.38 (dd, *J* = 4.8, 1.6 Hz, 1H), 7.93 – 7.89 (m, 2H), 7.87 – 7.84 (m, 1H), 7.82 – 7.77 (m, 1H), 7.54 – 7.49 (m, 2H), 7.49 – 7.44 (m, 3H), 7.42 (dd, *J* = 7.6, 1.7 Hz, 1H), 7.15 – 7.09 (m, 3H), 7.07 (dd, *J* = 7.6, 4.8 Hz, 1H), 3.61 (s, 2H), 3.45 – 3.33 (m, 2H), 2.87 – 2.73 (m, 4H), 2.55 (ddd, *J* = 14.2, 10.0, 4.3 Hz, 1H), 2.45 (ddd, *J* = 14.0, 9.9, 4.3 Hz, 1H), 2.40 – 2.30 (m, 2H), 2.25 – 2.13 (m, 2H).

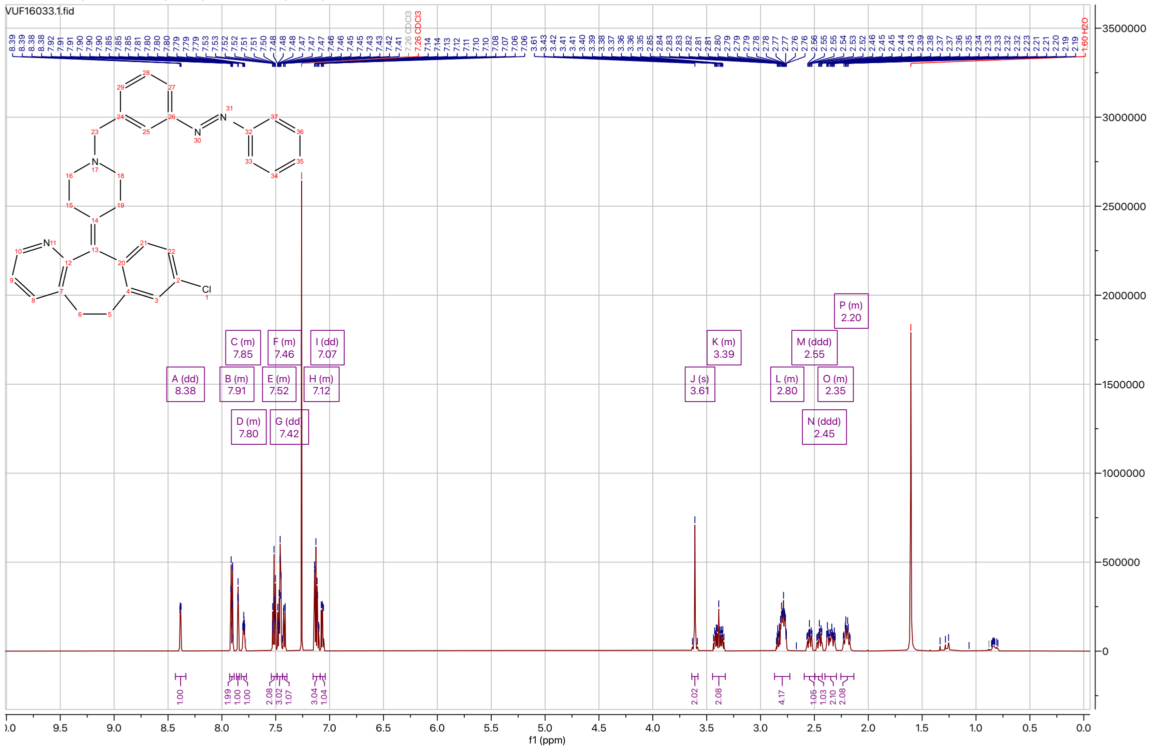

Figure S119. ^{1}H NMR spectrum of **12b.**

^{13}C NMR (151 MHz, $CDCl_3$) δ 157.8, 152.8, 152.8, 146.8, 139.8, 139.6, 139.2, 138.0, 137.3, 133.5, 132.8, 131.8, 131.1, 131.0, 129.2, 129.1, 126.1, 123.6, 123.0, 122.2, 121.8, 62.7, 55.0, 54.9, 32.0, 31.6, 31.2, 30.9.

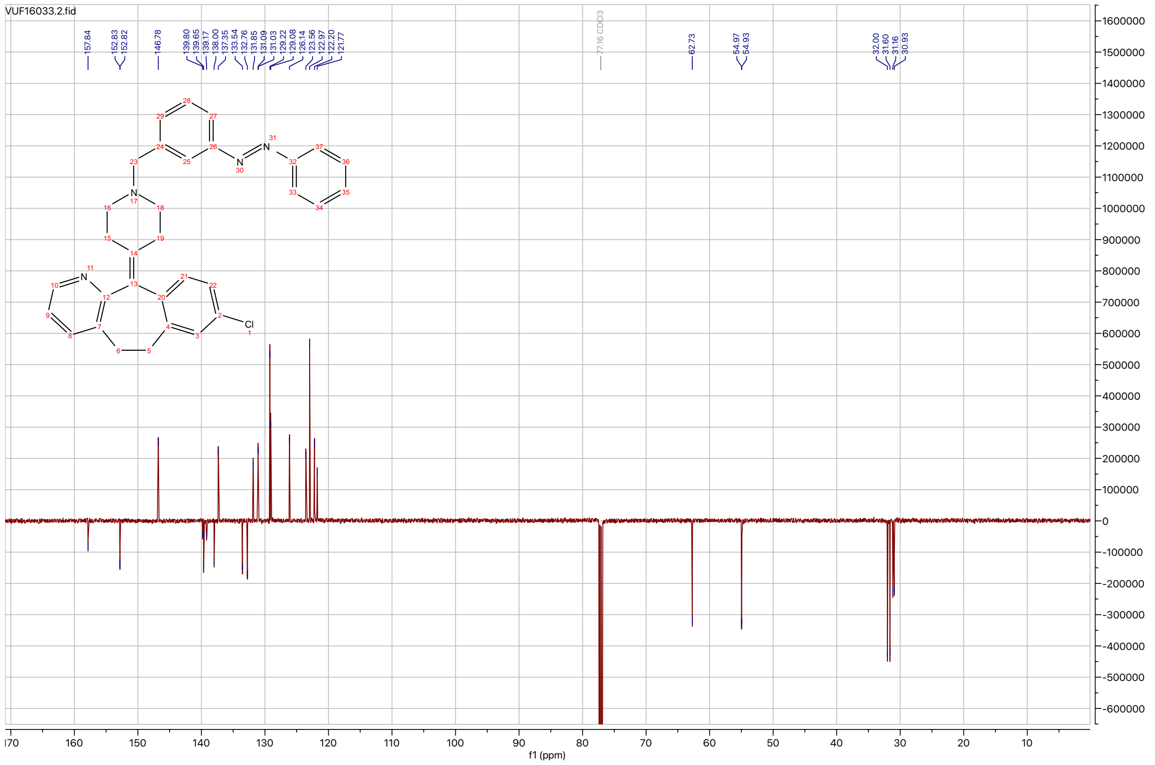

Figure S120. ^{13}C NMR spectrum of **12b**.

Acquired by : Admin
Date Acquired : 5/1/2025 1:41:09 PM
Sample Name : NIHA_02_011
Sample ID :
Tray# : 1
Vial# : 3
Injection Volume : 5
Data File : C:\LabSolutions\Data\2025\2025-wk18\NIHA_02_011.lcd
Background File : azoblanco 01052025.lcd
Method File : Method SCAN ACID standard azo.lcm
Report Format : DefaultLCMS.lcr
Tuning File : C:\LabSolutions\Tuning File\Tuning-ESI-pos-neg01072015.lct
Processed by : Admin
Modified Date : 5/1/2025 2:07:27 PM

Chromatogram
NIHA_02_011 C:\LabSolutions\Data\2025\2025-wk18\NIHA_02_011.lcd

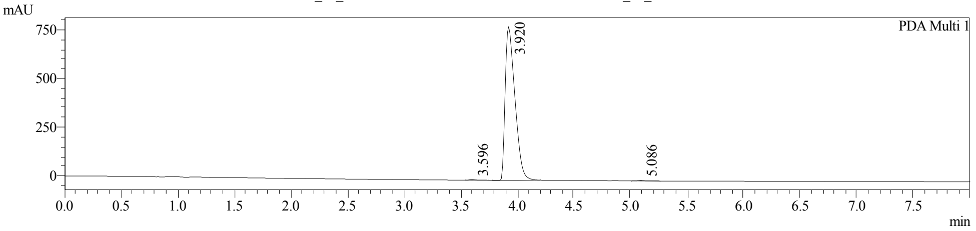

1 PDA Multi 1 / 254nm 4nm

PeakTable

PDA Ch1 254nm 4nm

Peak#	Ret. Time	Area	Height	Name	Area %
1	3.596	10839	3371		0.231
2	3.920	4670123	787775		99.579
3	5.086	8927	2250		0.190
Total		4689888	793396		100.000

MS Chromatogram
NIHA_02_011 C:\LabSolutions\Data\2025\2025-wk18\NIHA_02_011.lcd

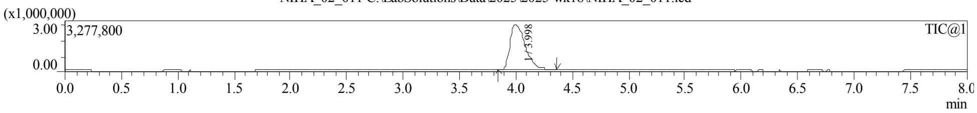

MS Spectrum Graph

#:1 Ret.Time:Averaged 3.990-4.010(Scan#:400-402)
BG Mode:Calc 3.850<->4.370(386<->438)
Mass Peaks:16 Base Peak:505.25(1300741) Polarity:Pos Segment1 - Event1

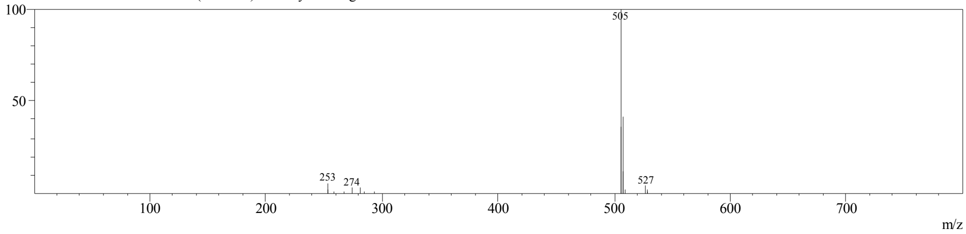

MS Spectrum Table

#:1 Ret.Time:
BG Mode:Calc 3.850<->4.370(386<->438)
Mass Peaks:16 Base Peak:505.25(1300741) Polarity:Pos Segment1 - Event1

#	m/z	Abs.Inten.	Rel.Inten.	Charge	Polarity	Monoisotopic
1	253.15	72082	5.54			
2	254.20	26114	2.01			
3	259.10	13285	1.02			
4	267.15	13487	1.04			
5	273.75	34786	2.67			
6	282.05	34816	2.68			
7	284.05	14476	1.11			
8	294.20	15062	1.16			

#	m/z	Abs.Inten.	Rel.Inten.	Charge	Polarity	Monoisotopic
9	505.25	1300741	100.00			
10	506.30	468635	36.03			
11	507.25	549348	42.23			
12	508.30	154293	11.86			
13	509.30	31648	2.43			
14	527.30	50539	3.89			
15	528.35	15503	1.19			
16	529.35	18608	1.43			

Figure S121. LC-MS chromatogram of **12b**.

Generic Display Report

Analysis Info		Acquisition Date	4/23/2025 9:03:01 AM
Analysis Name	D:\Data\ServiceMS\Hans\2025-wk17\LBI VUF16033_4-23-2025_09-02-18_100-1200mz range1.d		
Method	100-1200mz range1.m	Operator	Demo User
Sample Name	LBI VUF16033	Instrument	impact II
Comment			

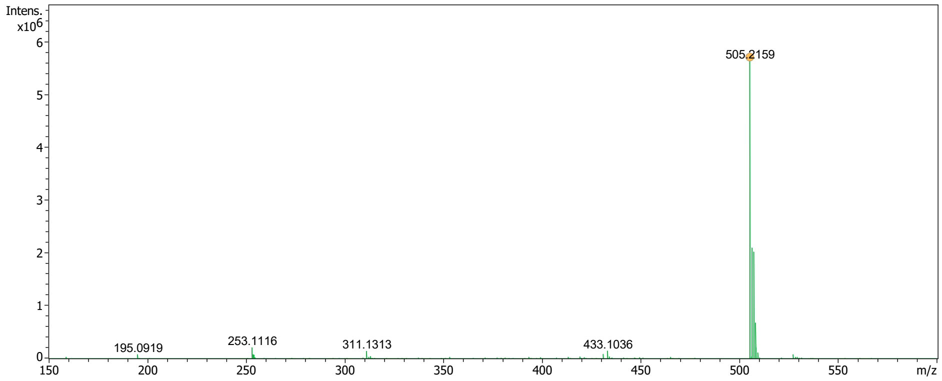

Meas. m/z	#	Ion Formula	m/z	err [ppm]	mSigma	#mSigma	Score	rdb	e⁻ Conf	N-Rule
505.2159	1	C32H30ClN4	505.2154	-1.0	11.8	1	100.00	23.0	even	ok

Bruker Compass DataAnalysis 6.0 printed: 4/23/2025 10:53:30 AM by: demo Page 1 of 1

Figure S122. HRMS spectrum of **12b**.

^{1}H NMR (600 MHz, $CDCl_3$) δ 8.38 (dd, *J* = 4.8, 1.7 Hz, 1H), 7.81 (dd, *J* = 8.1, 1.3 Hz, 1H), 7.68 (d, *J* = 7.7 Hz, 1H), 7.53 (ddd, *J* = 7.6, 7.6, 1.3 Hz, 1H), 7.42 (dd, *J* = 7.6, 1.7 Hz, 1H), 7.40 – 7.34 (m, 1H), 7.18 – 7.09 (m, 3H), 7.07 (dd, *J* = 7.6, 4.8 Hz, 1H), 3.78 (s, 2H), 3.44 – 3.30 (m, 2H), 2.88 – 2.74 (m, 3H), 2.73 – 2.66 (m, 2H), 2.49 (ddd, *J* = 14.1, 9.9, 4.3 Hz, 1H), 2.40 (ddd, *J* = 14.0, 9.8, 4.3 Hz, 1H), 2.36 – 2.25 (m, 2H), 2.25 – 2.12 (m, 2H).

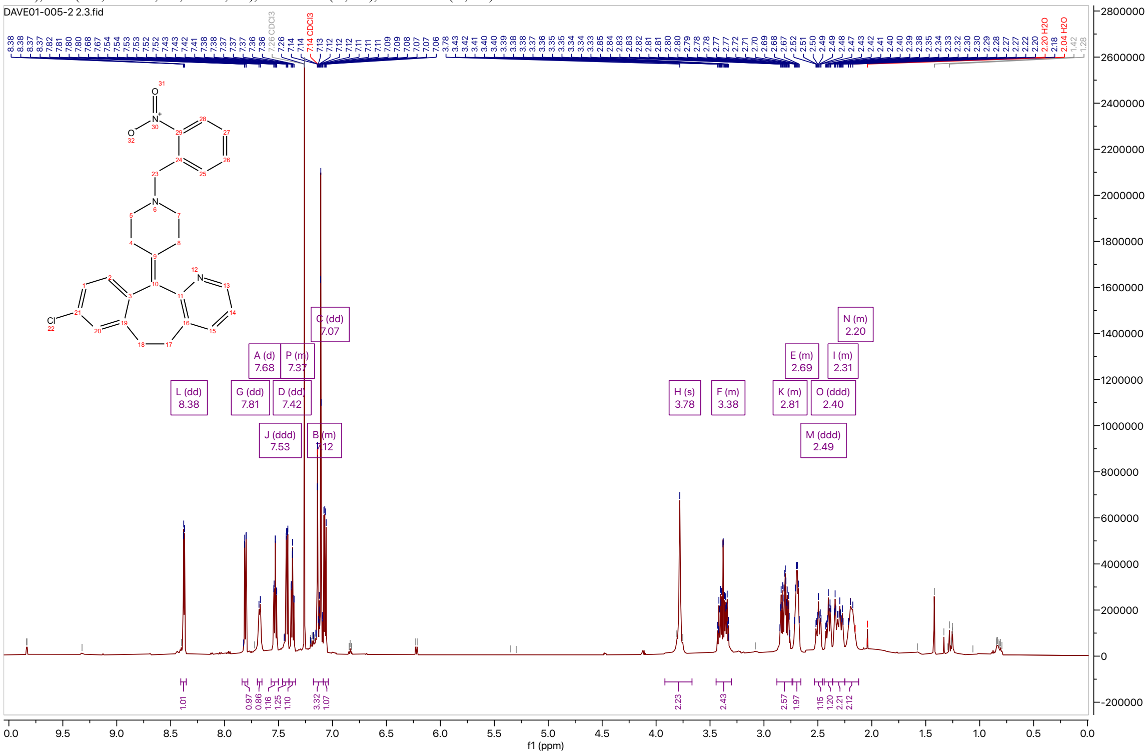

Figure S123. ^{1}H NMR spectrum of **42.**

^{13}C NMR (151 MHz, $CDCl_3$) δ 157.7 , 149.9, 146.7, 139.7, 138.7$^{\#}$, 137.9, 137.4, 134.3$^{\#}$, 133.6, 132.9, 132.8, 132.6, 131.0, 130.9, 129.1, 127.9, 126.1, 124.5, 122.2, 58.8, 55.0, 54.9, 31.9, 31.6, 31.1, 30.9.

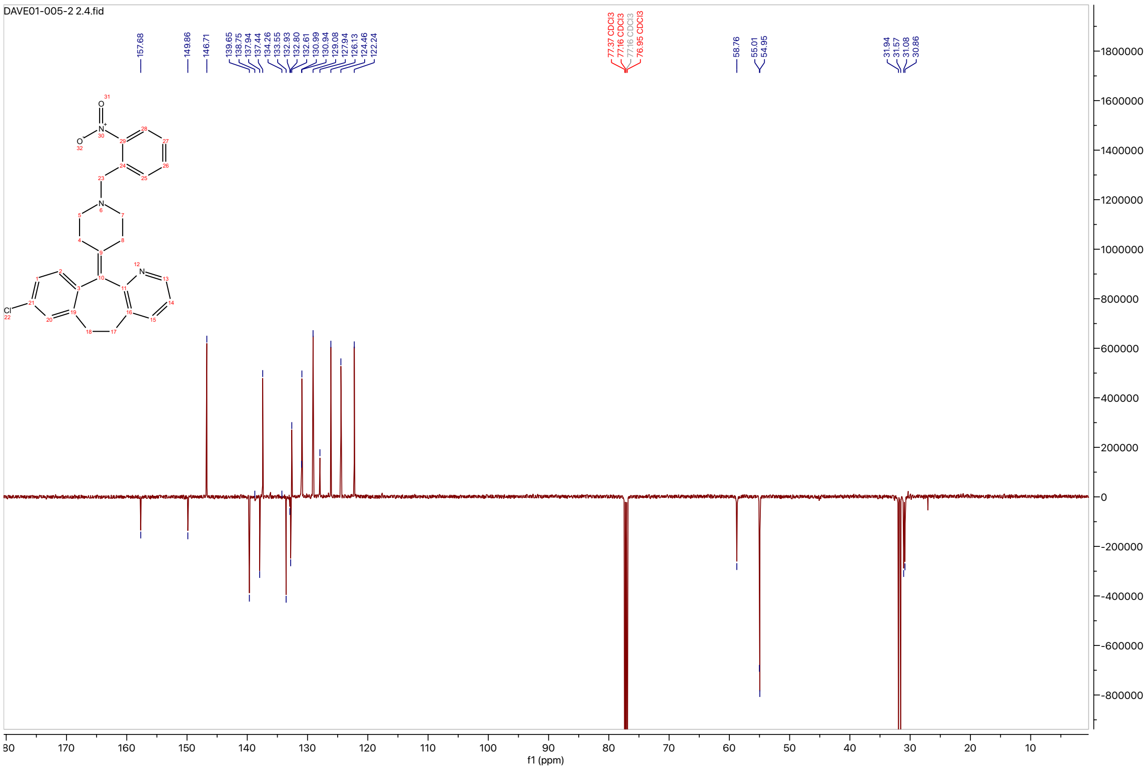

Figure S124. ^{13}C NMR spectrum of **42**.

Acquired by	: Admin
Date Acquired	: 4/23/2024 4:56:54 PM
Sample Name	: DAVE01-005-4
Sample ID	:
Tray#	: 1
Vial#	: 20
Injection Volume	: 1
Data File	: C:\LabSolutions\Data\2024\2024-wk17\DAVE01-005-4.lcd
Background File	: blanco 23042024.lcd
Method File	: Method SCAN ACID standard.lcm
Report Format	: DefaultLCMS.lcr
Tuning File	: C:\LabSolutions\Tuning File\Tuning-ESI-pos-neg01072015.lct
Processed by	: Admin
Modified Date	: 4/24/2024 9:10:37 AM

Chromatogram
DAVE01-005-4 C:\LabSolutions\Data\2024\2024-wk17\DAVE01-005-4.lcd

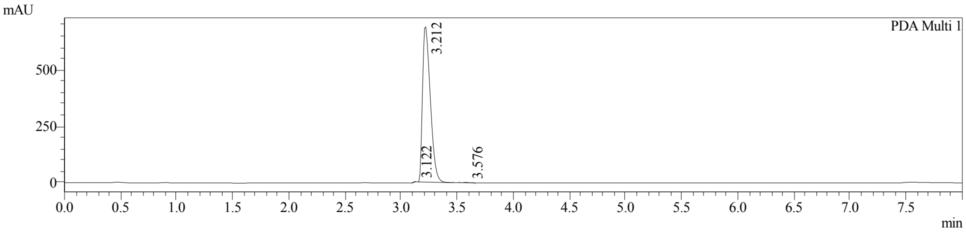

1 PDA Multi 1 / 230nm 4nm

PeakTable

PDA Ch1 230nm 4nm

Peak#	Ret. Time	Area	Height	Name	Area %
1	3.122	4388	2352		0.130
2	3.212	3366376	687518		99.715
3	3.576	5217	1440		0.155
Total		3375981	691310		100.000

MS Chromatogram
DAVE01-005-4 C:\LabSolutions\Data\2024\2024-wk17\DAVE01-005-4.lcd

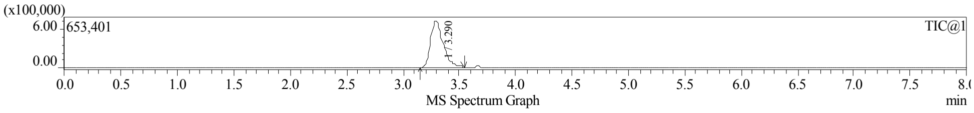

MS Spectrum Graph

#:1 Ret.Time:Averaged 3.260-3.310(Scan#:327-332)
BG Mode:None
Mass Peaks:15 Base Peak:446.15(272960) Polarity:Pos Segment1 - Event1

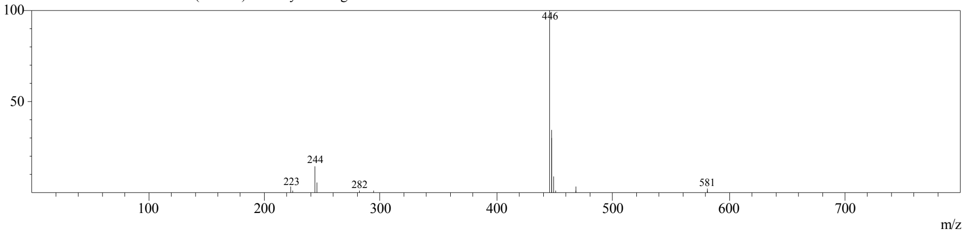

MS Spectrum Table

#:1 Ret.Time:
BG Mode:None
Mass Peaks:15 Base Peak:446.15(272960) Polarity:Pos Segment1 - Event1

#	m/z	Abs.Inten.	Rel.Inten.	Charge	Polarity	Monoisotopic
1	223.50	8352	3.06			
2	224.45	4720	1.73			
3	244.05	41147	15.07			
4	245.10	15116	5.54			
5	282.10	3842	1.41			
6	294.00	3057	1.12			
7	446.15	272960	100.00			
8	447.10	81416	29.83			
9	448.15	94993	34.80			
10	449.15	24117	8.84			
11	450.20	3859	1.41			
12	468.20	8298	3.04			
13	469.10	3085	1.13			
14	581.25	6437	2.36			
15	582.30	3367	1.23			

Figure S125. LC-MS chromatogram of **42**.

^{1}H NMR (500 MHz, $CDCl_3$) δ 8.37 (dd, *J* = 4.8, 1.7 Hz, 1H), 7.41 (dd, *J* = 7.6, 1.7 Hz, 1H), 7.15 (d, *J* = 1.3 Hz, 1H), 7.11 (d, *J* = 1.3 Hz, 2H), 7.09 – 7.05 (m, 2H), 6.96 – 6.91 (m, 1H), 6.67 – 6.60 (m, 2H), 4.98 (br s, 2H), 3.56 (s, 2H), 3.46 – 3.28 (m, 2H), 2.92 – 2.71 (m, 4H), 2.61 – 2.09 (m, 6H).

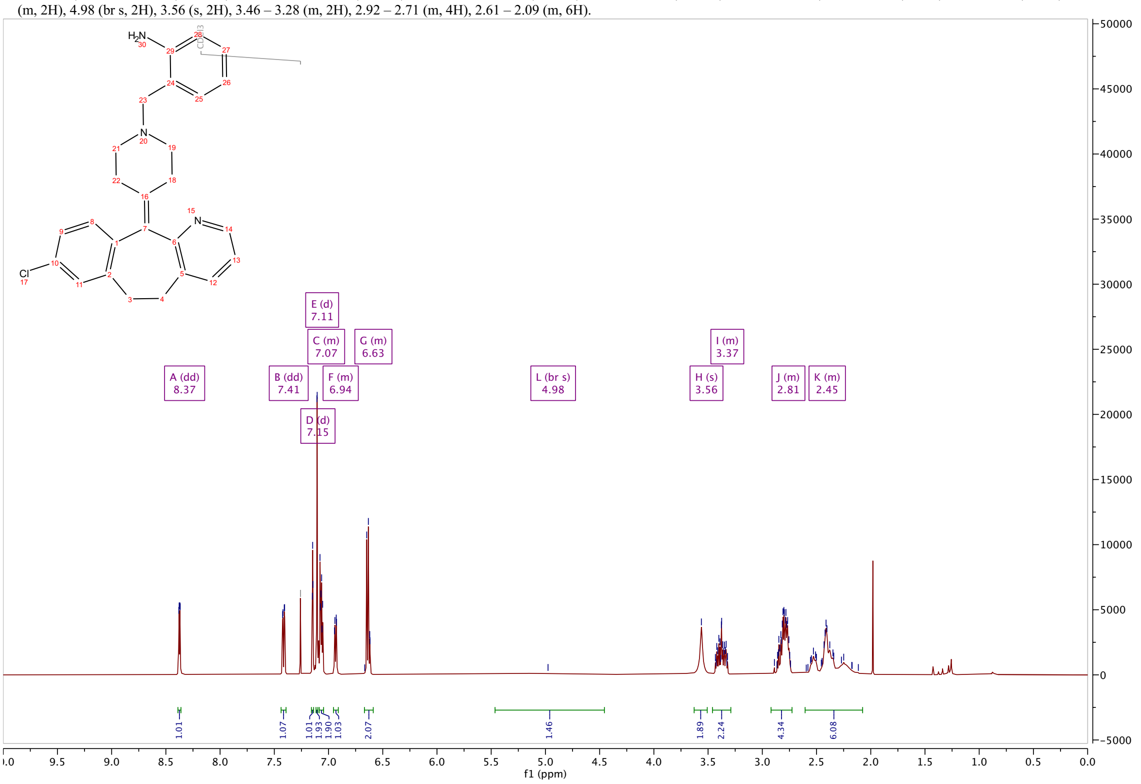

Figure S126. ^{1}H NMR spectrum of **43.**

^{13}C NMR (126 MHz, $CDCl_3$) δ 157.4, 147.2, 146.7, 139.6, 137.8, 137.5, 133.5, 132.9, 130.9, 130.7, 129.1, 128.8, 126.1, 122.3, 117.7, 115.9, 61.5, 54.5, 54.4, 31.8, 31.5, 30.7, 30.6.

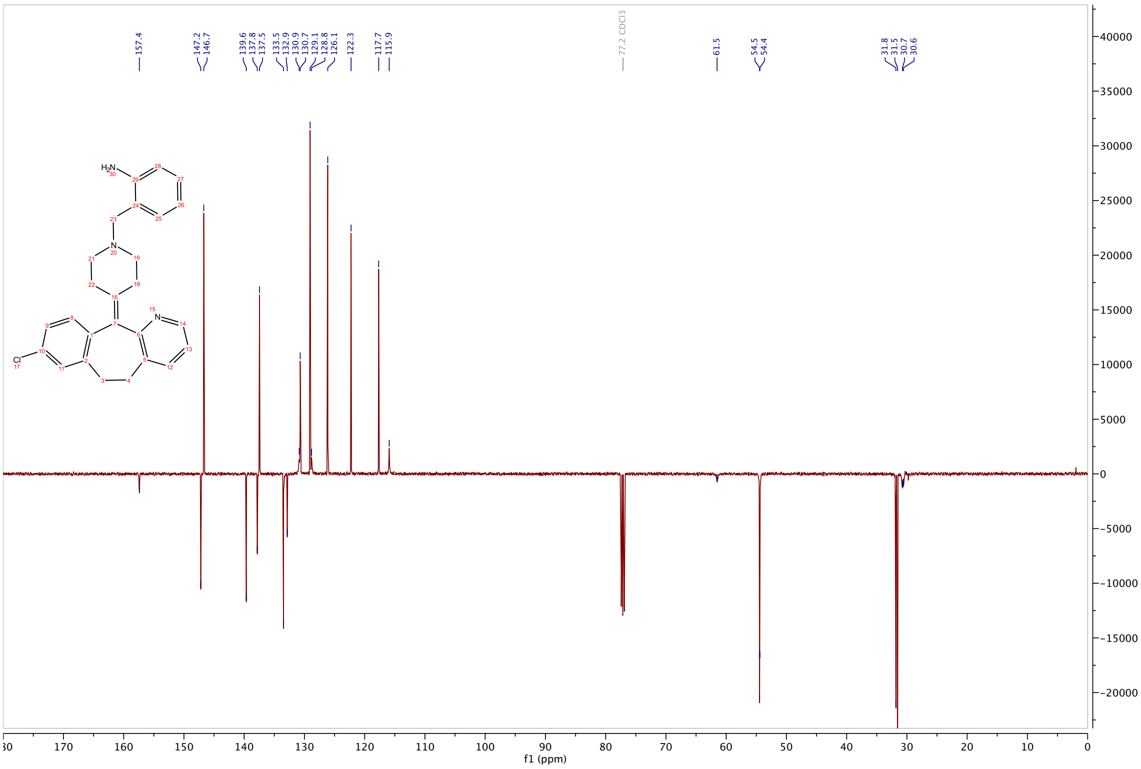

Figure S127. ^{13}C NMR spectrum of **43**.

Date Acquired : 25/04/2024 10:31:46
Sample Name : DAVE01-007-2
Sample ID :
Tray# : 1
Vial# : 44
Injection Volume : 0.3
Data File : C:\LabSolutions\Data\2024\2024-wk17\DAVE01-007-2.lcd
Background File : blanco 25042024.lcd
Method File : Standard acid posneg.lcm
Report Format : DEFAULT.lsr
Tuning File : 01072022_Tunefile.lct
Modified Date : 08/05/2024 08:59:22

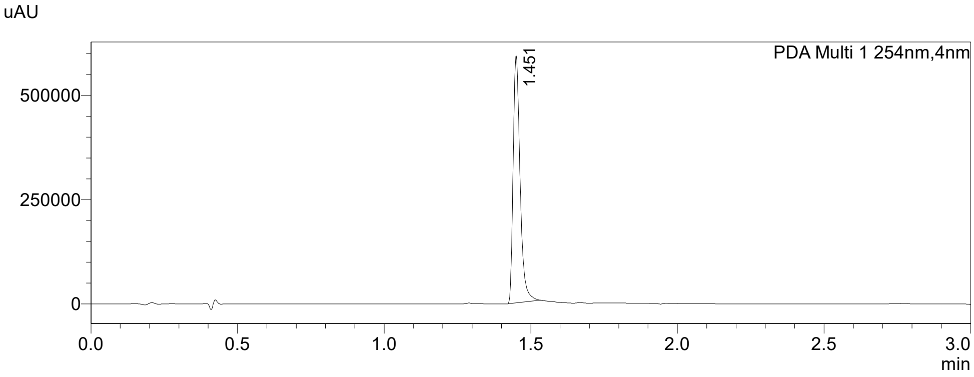

PDA Peak Table

PDA Ch1 254nm

Peak#	Ret. Time	Area	Area%
1	1.451	976456	100.000
Total		976456	100.000

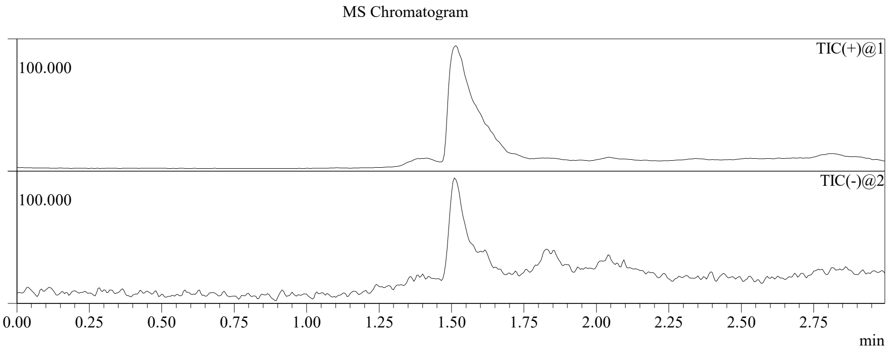

Figure S128. LC-MS chromatogram of **43**.

^{1}H NMR (600 MHz, $CDCl_3$) δ 8.37 (dd, J = 4.8, 1.6 Hz, 1H), 7.92 – 7.86 (m, 2H), 7.66 – 7.60 (m, 2H), 7.52 (dd, J = 8.3, 6.5 Hz, 2H), 7.50 – 7.47 (m, 1H), 7.45 (ddd, J = 8.8, 7.6, 2.0 Hz, 1H), 7.41 (dd, J = 7.7, 1.7 Hz, 1H), 7.34 (ddΔ, J = 7.5 Hz, 1H), 7.13 – 7.10 (m, 2H), 7.09 (dd, J = 8.1, 2.0 Hz, 1H), 7.06 (dd, J = 7.7, 4.7 Hz, 1H), 4.14 (s, 2H), 3.43 – 3.28 (m, 2H), 2.89 – 2.81 (m, 2H), 2.81 – 2.73 (m, 2H), 2.53 (ddd, J = 14.2, 10.0, 4.3 Hz, 1H), 2.43 (ddd, J = 14.0, 9.8, 4.2 Hz, 1H), 2.37 – 2.29 (m, 2H), 2.29 – 2.18 (m, 2H).

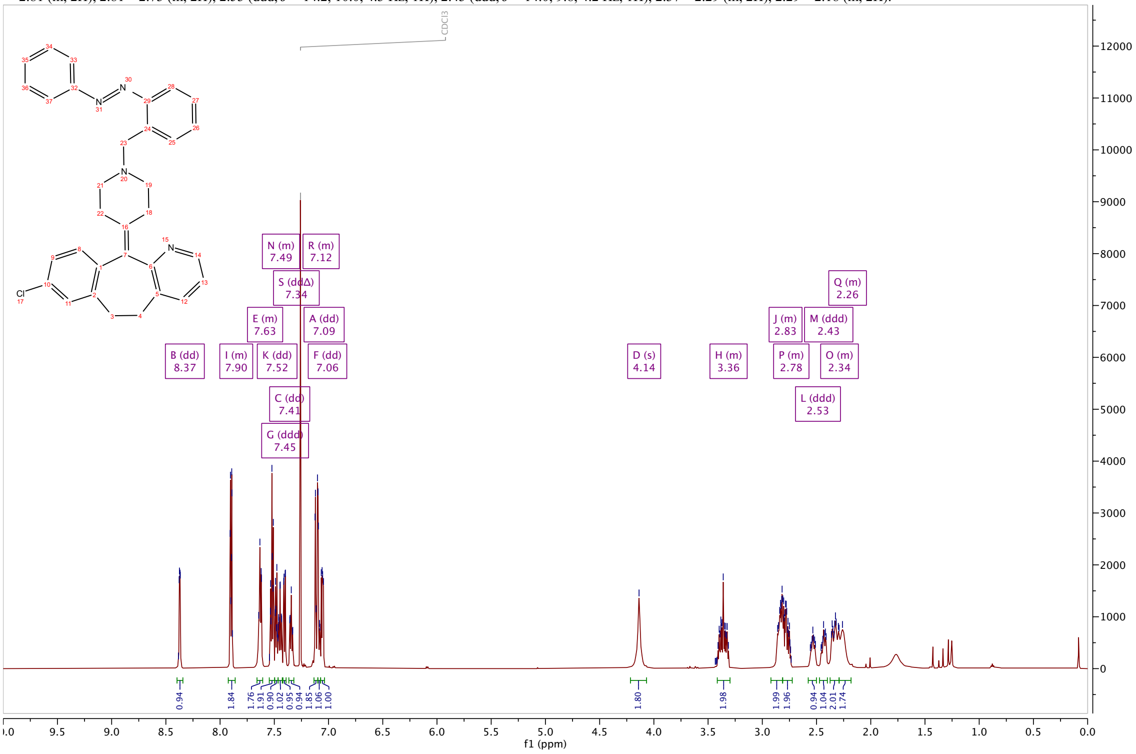

Figure S129. ^{1}H NMR spectrum of **12c.**

^{13}C NMR (151 MHz, $CDCl_3$) δ 157.8, 153.0, 151.0, 146.7, 139.6, 139.0, 138.0, 137.4, 137.3, 133.5, 132.7, 132.5, 131.1, 131.0, 131.0, 130.9, 129.2, 129.1, 127.8, 126.1, 123.1, 122.2, 115.4, 56.6, 54.8, 54.7, 32.0, 31.6, 31.2, 31.0.

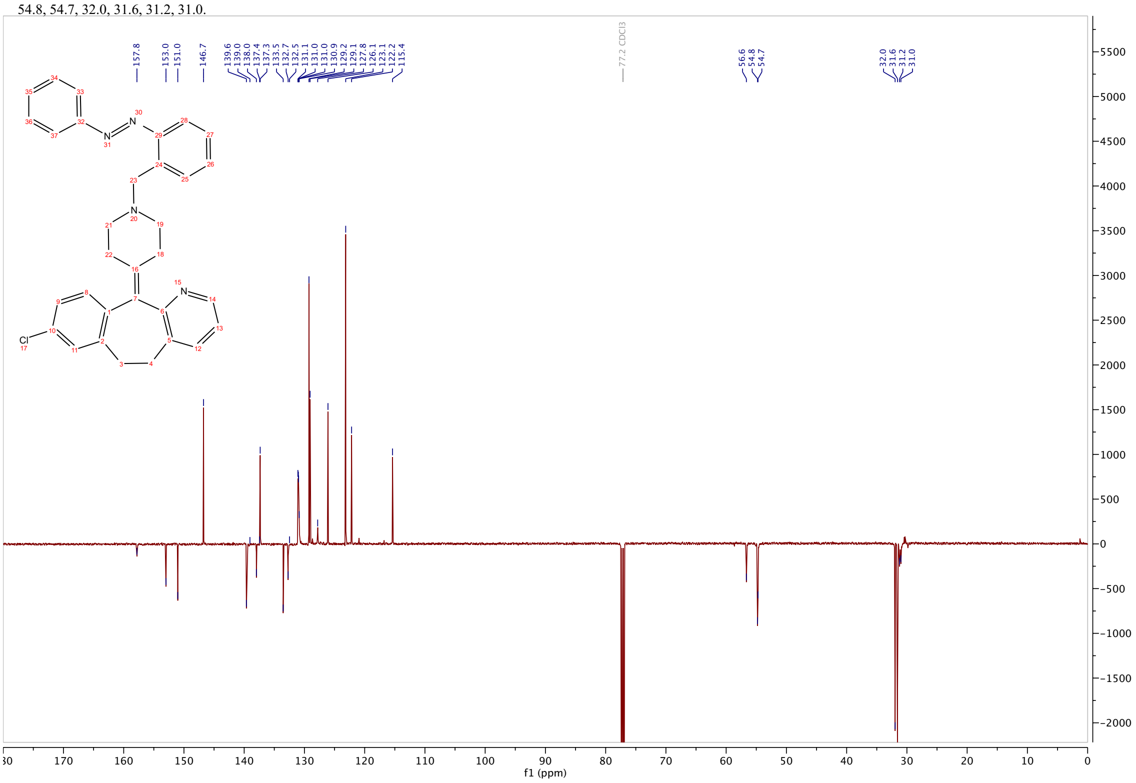

Figure S130. ^{13}C NMR spectrum of **12c**.

Acquired by	: Admin
Date Acquired	: 5/29/2024 11:00:27 AM
Sample Name	: DAVE01-010-8
Sample ID	:
Tray#	: 1
Vial#	: 3
Injection Volume	: 3
Data File	: C:\LabSolutions\Data\2024\2024-wk22\DAVE01-010-8.lcd
Background File	: azoblanco 29052024.lcd
Method File	: Method SCAN ACID standard azo.lcm
Report Format	: DefaultLCMS.lcr
Tuning File	: C:\LabSolutions\Tuning File\Tuning-ESI-pos-neg01072015.lct
Processed by	: Admin
Modified Date	: 5/29/2024 12:39:28 PM

Chromatogram
DAVE01-010-8 C:\LabSolutions\Data\2024\2024-wk22\DAVE01-010-8.lcd

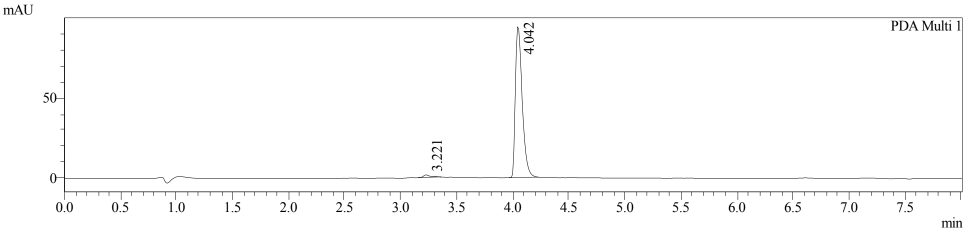

1 PDA Multi 1 / 254nm 4nm

PeakTable

PDA Ch1 254nm 4nm

Peak#	Ret. Time	Area	Height	Name	Area %
1	3.221	7332	1519		1.800
2	4.042	399990	93655		98.200
Total		407322	95175		100.000

MS Chromatogram
DAVE01-010-8 C:\LabSolutions\Data\2024\2024-wk22\DAVE01-010-8.lcd

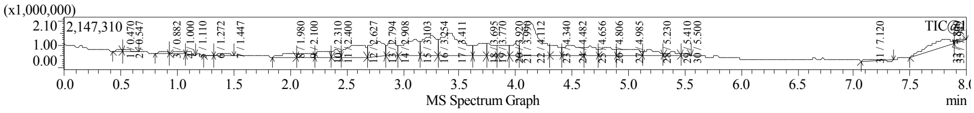

MS Spectrum Graph

#:1 Ret.Time:Averaged 4.100-4.140(Scan#:411-415)
BG Mode:None
Mass Peaks:26 Base Peak:505.20(415131) Polarity:Pos Segment1 - Event1

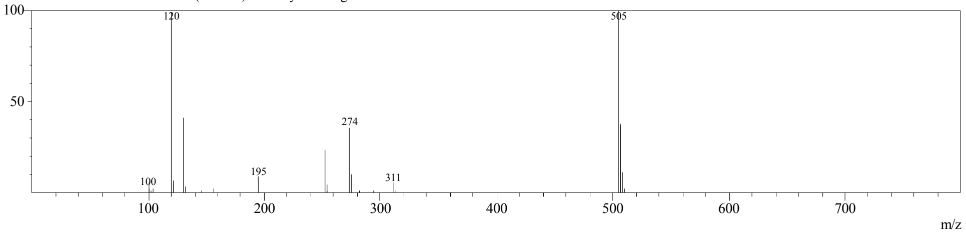

MS Spectrum Table

#:1 Ret.Time:
BG Mode:None
Mass Peaks:26 Base Peak:505.20(415131) Polarity:Pos Segment1 - Event1

#	m/z	Abs.Inten.	Rel.Inten.	Charge	Polarity	Monoisotopic
1	100.00	11935	2.87			
2	101.00	22850	5.50			
3	102.00	6688	1.61			
4	105.00	10742	2.59			
5	120.00	408173	98.32			
6	120.95	27676	6.67			
7	122.00	17269	4.16			
8	130.05	171165	41.23			

#	m/z	Abs.Inten.	Rel.Inten.	Charge	Polarity	Monoisotopic
9	131.20	13563	3.27			
10	146.00	4843	1.17			
11	156.85	10421	2.51			
12	195.00	35577	8.57			
13	253.25	96936	23.35			
14	253.75	21097	5.08			
15	254.75	7244	1.74			
16	273.65	149247	35.95			

Figure S131. LC-MS chromatogram of **12c**.

HRMS MedChem

**Analysis Info**		Acquisition Date	7/31/2024 9:49:58 AM
Analysis Name	D:\Data\ServiceMS\Hans\2024-wk31\DAVE VUF26784_7-31-2024_09-49-06_ServiceMs Hystar.d		
Method	ServiceMs Hystar.m	Operator	Demo User
Sample Name	DAVE VUF26784	Instrument	impact II
Comment			

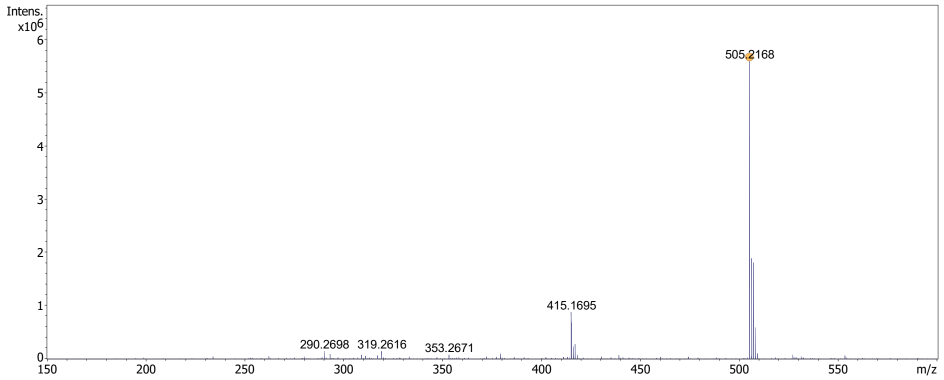

Meas. m/z	#	Ion Formula	m/z	err [ppm]	mSigma	#mSigma	Score	rdb	e⁻ Conf	N-Rule
505.2168	1	C32H30ClN4	505.2154	-2.9	30.8	1	100.00	23.0	even	ok

Figure S132. HRMS spectrum of **12c**.

References

(1) Wang, D.; Guo, Q.; Wu, Z.; Li, M.; He, B.; Du, Y.; Zhang, K.; Tao, Y. Molecular Mechanism of Antihistamines Recognition and Regulation of the Histamine H1 Receptor. *Nature Communications 2024 15:1* **2024**, *15* (1), 1–10. https://doi.org/10.1038/s41467-023-44477-4.

(2) Bosma, R.; Moritani, R.; Leurs, R.; Vischer, H. F. BRET-Based β-Arrestin2 Recruitment to the Histamine H1 Receptor for Investigating Antihistamine Binding Kinetics. *Pharmacol Res* **2016**, *111*, 679–687. https://doi.org/10.1016/J.PHRS.2016.07.034.

(3) Bosma, R.; Wang, Z.; Kooistra, A. J.; Bushby, N.; Kuhne, S.; Van Den Bor, J.; Waring, M. J.; De Graaf, C.; De Esch, I. J.; Vischer, H. F.; Sheppard, R. J.; Wijtmans, M.; Leurs, R. Route to Prolonged Residence Time at the Histamine H1 Receptor: Growing from Desloratadine to Rupatadine. *J Med Chem* **2019**, *62* (14), 6630–6644. https://doi.org/10.1021/acs.jmedchem.9b00447.

(4) Yung-Chi, C.; Prusoff, W. H. Relationship between the Inhibition Constant (KI) and the Concentration of Inhibitor Which Causes 50 per Cent Inhibition (I50) of an Enzymatic Reaction. *Biochem Pharmacol* **1973**, *22* (23), 3099–3108. https://doi.org/10.1016/0006-2952(73)90196-2.

(5) Ahmed, Z.; Siiskonen, A.; Virkki, M.; Priimagi, A. Controlling Azobenzene Photoswitching through Combined Ortho-Fluorination and -Amination. *Chemical Communications* **2017**, *53* (93), 12520–12523. https://doi.org/10.1039/C7CC07308A.
